# Development of a consensus statement on the role of the family in the physical activity, sedentary, and sleep behaviours of children and youth

**DOI:** 10.1186/s12966-020-00973-0

**Published:** 2020-06-16

**Authors:** Ryan E. Rhodes, Michelle D. Guerrero, Leigh M. Vanderloo, Kheana Barbeau, Catherine S. Birken, Jean-Philippe Chaput, Guy Faulkner, Ian Janssen, Sheri Madigan, Louise C. Mâsse, Tara-Leigh McHugh, Megan Perdew, Kelly Stone, Jacob Shelley, Nora Spinks, Katherine A. Tamminen, Jennifer R. Tomasone, Helen Ward, Frank Welsh, Mark S. Tremblay

**Affiliations:** 1 Behavioural Medicine Laboratory, Faculty of Education, University of Victoria, PO Box 3010 STN CSC, Victoria, BC V8W 3N4 Canada; 2 Healthy Active Living and Obesity Research Group, Children’s Hospital of Eastern Ontario Research Institute, 401 Smyth Road, Ottawa, ON K1H 8L1 Canada; 3 ParticipACTION, Toronto, ON M5S 1M2 Canada; 4 School of Psychology, University of Ottawa, Ottawa, ON K1N 9A8 Canada; 5 SickKids Research Institute and the Department of Pediatrics, University of Toronto, Toronto, ON M5G 1X8 Canada; 6 School of Kinesiology, University of British Columbia, Vancouver, BC V6T 1Z4 Canada; 7 School of Kinesiology and Health Studies, Queen’s University, Kingston, ON K7L 3N6 Canada; 8 Department of Psychology, Faculty of Arts, University of Calgary, Calgary, AB T2N 1N4 Canada; 9 BC Children’s Hospital Research Institute / School of Population and Public Health, Faculty of Medicine, University of British Columbia, Vancouver, BC V6H 3N1 Canada; 10 Faculty of Kinesiology, Sport and Recreation, University of Alberta, Edmonton, Alberta T6G 2H9 Canada; 11 Families Canada, Ottawa, ON K1S 1V7 Canada; 12 Faculty of Law & School of Health Studies, Faculty of Health Sciences, Western University, London, ON N6A 3K7 UK; 13 The Vanier Institute of the Family, Ottawa, ON K2G 6B1 Canada; 14 Faculty of Kinesiology and Physical Education, University of Toronto, Toronto, ON M5S 2W6 Canada; 15 Kids First Parents Association of Canada, Burnaby, BC V5C 2H2 Canada; 16 Canadian Public Health Association, Ottawa, ON K1G 3Y6 Canada

**Keywords:** Parent, Guardian, Sibling, Role model, Support, Physical activity, Sleep, Sedentary behaviours, Rules, Parenting practices, Structure, Health

## Abstract

**Background:**

Children and youth who meet the physical activity, sedentary, and sleep behaviour recommendations in the Canadian 24-Hour Movement Guidelines are more likely to have desirable physical and psychosocial health outcomes. Yet, few children and youth actually meet the recommendations. The family is a key source of influence that can affect lifestyle behaviours. The purpose of this paper is to describe the process used to develop the *Consensus Statement on the Role of the Family in the Physical Activity, Sedentary, and Sleep Behaviours of Children and Youth* (0–17 years) and present, explain, substantiate, and discuss the final *Consensus Statement.*

**Methods:**

The development of the *Consensus Statement* included the establishment of a multidisciplinary Expert Panel, completion of six reviews (three literature, two scoping, one systematic review of reviews), custom data analyses of Statistics Canada’s Canadian Health Measures Survey, integration of related research identified by Expert Panel members, a stakeholder consultation, establishment of consensus, and the development of a media, public relations, communications and launch plan.

**Results:**

Evidence from the literature reviews provided substantial support for the importance of family on children’s movement behaviours and highlighted the importance of inclusion of the entire family system as a source of influence and promotion of healthy child and youth movement behaviours. The Expert Panel incorporated the collective evidence from all reviews, the custom analyses, other related research identified, and stakeholder survey feedback, to develop a conceptual model and arrive at the *Consensus Statement: Families can support children and youth in achieving healthy physical activity, sedentary and sleep behaviours by encouraging, facilitating, modelling, setting expectations and engaging in healthy movement behaviours with them. Other sources of influence are important (*e.g.*, child care, school, health care, community, governments) and can support families in this pursuit*.

**Conclusion:**

Family is important for the support and promotion of healthy movement behaviours of children and youth. This *Consensus Statement* serves as a comprehensive, credible, and current synopsis of related evidence, recommendations, and resources for multiple stakeholders.

## Background

The Canadian 24-Hour Movement Guidelines were recently developed to provide public health guidelines integrating recommendations for physical activity, sedentary, and sleep behaviours for the pediatric population ranging from 0 to 4 years and 5 to 17 years [1, 2]. Children and youth who adhere to these guidelines are more likely to display healthy growth, body composition, cardiorespiratory and musculoskeletal fitness, cardiovascular and metabolic health, motor development, cognitive development, academic achievement, emotional regulation, pro-social behaviours, and overall quality of life [3–12]. Furthermore, healthy movement behaviours in childhood are associated with a higher likelihood of healthy physical activity [13], sedentary [14], and sleep [15] behaviours in later adolescence or adulthood and their commensurate positive health outcomes [16, 17].

Because of the unequivocal holistic health benefits of healthy movement behaviours during childhood and adolescence, several authoritative groups around the world have released, endorsed or promoted evidence-based movement guidelines and recommendations to inform the public [1, 2, 18–31]. A sample of these guidelines, resources and links are summarized in Table 1 to demonstrate the consistency of recommendations and the rapid global adoption of the 24-h movement paradigm. In essence, these guidelines from many countries and jurisdictions recommend that children move more, sit less, limit recreational screen-time, and preserve a good night’s sleep every day. The Canadian 24-h movement guidelines [1, 2] were the first such guidelines and are highlighted in bold in Table 1.

**Table 1:** Movement Behaviour Guidelines for Children and Youth (ages 0–17 years) from different authoritative organizations

Authoritative Organization and Reference	Country	Age Group	Recommendation PHYSICAL ACTIVITY	Recommendation SCREENTIME/ SEDENTARY TIME	Recommendation SLEEP	Additional Notes
American Academy of Pediatrics Council on Communications and Media. 2016 [24]	USA	0–1.5 yrs		Avoid digital media use (except video chatting) in children younger than 18 to 24 months.		
		1.5–2 yrs		Avoid solo media use in this age group.		For children ages 18 to 24 months of age, if you want to introduce digital media, choose high-quality programming and use media together with your child. Avoid solo media use in this age group. Use the Family Media Use Plan (www.healthychildren.org/MediaUsePlan) with specific guidelines for each child and parent.
		2–5 yrs		For children 2 to 5 yrs. of age, limit screen use to 1 h per day of high-quality programming.		Create unplugged spaces and times in homes. Use new technologies in social and creative ways. Stress the importance of not displacing sleep, exercise, play, reading aloud, and social interaction with screen use. Recommend no screens during meals and for 1 h before bedtime. Remove devices from bedrooms before bed. For children 2–5 yrs. of age, limit screen use to 1 h per day of high-quality programming, co-view with your children, help children understand what they are seeing, and help them apply what they learn. Monitor children’s media content and what apps are used or downloaded. Test apps before the child uses them, play together, and ask the child what they think about the app. Keep bedrooms, mealtimes, and parent-child playtimes screen-free for children and parents. Parents can set a “do not disturb” option on their phones during these times. Avoid fast-paced programs (young children do not understand them as well), apps with lots of distracting content, and any violent content. Turn off TVs and other devices when not in use.
American Academy of Sleep Medicine. 2016 [20]	USA	4 months – 1 yr			Infants* 4 months to 12 months should sleep 12 to 16 h per 24 h (including naps).	*Recommendations for infants younger than 4 months are not included due to the wide range of normal variation in duration and patterns of sleep, and insufficient evidence for associations with health outcomes.
		1–2 yrs			11 to 14 h per 24 h (including naps)	
		3–5 yrs			10 to 13 h per 24 h (including naps)	
		6–12 yrs			9 to 12 h per 24 h	
		13–18 yrs			8 to 10 h per 24 h	
Australian 24-Hour Movement Guidelines for the Early Years (Birth to 5 years): An Integration of Physical Activity, Sedentary Behaviour, and Sleep. 2017 [23]	Australia	0–1 yr	Being physically active several times in a variety of ways, particularly through interactive floor-based play; more is better. For those not yet mobile, this includes at least 30 min of tummy time spread throughout the day while awake.	Not being restrained for more than 1 h at a time (e.g., in a stroller, car seat or high chair). When sedentary, engaging in pursuits such as reading and storytelling with a caregiver is encouraged. Screen-time is not recommended.	14 to 17 h (for those aged 0–3 months) of good quality sleep, including naps. 12 to 16 h (for those aged 4–11 months) of good quality sleep, including naps.	
		1–2 yrs	At least 180 min spent in a variety of physical activities at any intensity, spread throughout the day; more is better. Including energetic play.	Not being restrained for more than 1 h at a time (e.g., in a stroller, car seat or high chair) or sitting for extended periods. When sedentary, engaging in pursuits such as reading and storytelling with a caregiver is encouraged. For those younger than 2 yrs., sedentary screen-time is not recommended. For those aged 2 yrs., sedentary screen-time should be no more than 1 h per day; less is better.	11 to 14 h of good quality sleep, including naps, with consistent sleep and wakeup times.	
		3–5 yrs	At least 180 min spent in a variety of physical activities spread throughout the day, of which at least 60 min is energetic play; more is better.	Not being restrained for more than 1 h at a time (e.g., in a stroller or car seat). Avoid sitting for extended periods. When sedentary, engaging in pursuits such as reading and storytelling with a caregiver is encouraged. Sedentary screen-time should be no more than 1 h per day; less is better.	10 to 13 h of good quality sleep, which may include a nap, with consistent sleep and wakeup times.	
Australian Department of Health. Australian 24-Hour Movement Guidelines for Children and Young People (5–17 years) - An Integration of Physical Activity, Sedentary Behaviour and Sleep. 2019. Available from: https://www1.health.gov.au/internet/main/publishing.nsf/Content/health-24-hours-phys-act-guidelines [25]	Australia	5–13 yrs	Accumulating 60 min or more of MVPA per day involving mainly aerobic activities. Activities that are vigorous, as well as those that strengthen muscle and bone should be incorporated at least 3 days per week. Several hrs of a variety of light physical activities.	Limiting sedentary recreational screen-time to no more than 2 h per day. Breaking up long periods of sitting as often as possible.	An uninterrupted 9 to 11 h of sleep per night for those aged 5–13 yrs. Consistent bed and wake-up times.	For greater health benefits, replace sedentary time with additional MVPA, while preserving sufficient sleep.
		14–17 yrs			8 to 10 h per night for those aged 14–17 yrs. Consistent bed and wake-up times.	
**Canadian 24-Hour Movement Guidelines for the Early Years (0–4 years): An Integration of Physical Activity, Sedentary Behaviour, and Sleep. 2017** [2]	**Canada**	**0–1 yr**	**Being physically active several times in a variety of ways, particularly through interactive floor-based play; more is better.** **For those not yet mobile, this includes at least 30 min of tummy time spread throughout the day while awake.**	**Not being restrained for more than 1 h at a time (e.g., in a stroller or high chair). Screen-time is not recommended.** **When sedentary, engaging in pursuits such as reading and storytelling with a caregiver is encouraged.**	**14–17 h (for those aged 0–3 months) or 12–16 h (for those aged 4–11 months) of good-quality sleep, including naps.**	
		**1–2 yrs**	**At least 180 min spent in a variety of physical activities at any intensity, including energetic play, spread throughout the day — more is better.**	**Not being restrained for more than 1 h at a time (e.g., in a stroller or high chair) or sitting for extended periods.** **For those younger than 2 yrs, sedentary screen-time is not recommended. For those aged 2 yrs, sedentary screen-time should be no more than 1 h; less is better.** **When sedentary, engaging in pursuits such as reading and storytelling with a caregiver is encouraged.**	**11–14 h of good-quality sleep, including naps, with consistent bedtimes and wake-up times.**	
		**3–4 yrs**	**At least 180 min spent in a variety of physical activities spread throughout the day, of which at least 60 min is energetic play —more is better.**	**Not being restrained for more than 1 h at a time (e.g., in a stroller or car seat) or sitting for extended periods.** **Sedentary screen-time should be no more than 1 h; less is better.** **When sedentary, engaging in pursuits such as reading and storytelling with a caregiver is encouraged.**	**10–13 h of good-quality sleep, which may include a nap, with consistent bedtimes and wake-up times.**	
**Canadian 24-Hour Movement Guidelines for Children and Youth: An Integration of Physical Activity, Sedentary Behaviour, and Sleep. 2016** [1]	**Canada**	**5–13 yrs**	**An accumulation of at least 60 min/day of MVPA involving a variety of aerobic activities.** **Vigorous physical activity and muscle and bone strengthening activities should each be incorporated at least 3 days/wk.** **Several hours of a variety of structured and unstructured light physical activities.**	**No more than 2 h/day of recreational screen-time.** **Limited sitting for extended periods.**	**9–11 h**	**Preserving sufficient sleep, trading indoor for outdoor time, and replacing sedentary behaviours and light physical activity with additional MVPA can provide greater health benefits.**
		**14–17 yrs**			**8–10 h**	
Canadian Pediatric Society. Screen-time and Young Children: Promoting Health and Development in a Digital World. 2017 [26]	Canada	0 - < 2 yrs		Screen-time for children younger than 2 yrs. is not recommended.		Ensure that sedentary screen-time is not a routine part of childcare for children younger than 5 yrs. Maintain daily ‘screen-free’ times, especially for family meals and book-sharing. Avoid screens for at least 1 h before bedtime, given the potential for melatonin-suppressing effects. Be present and engaged when screens are used and, whenever possible, co-view with children. Be aware of content and prioritize educational, age-appropriate and interactive programming. Use parenting strategies that teach self-regulation, calming and limit-setting. Choose healthy alternatives, such as reading, outdoor play, and creative, hands-on activities. Turn off devices at home during family time. Turn off screens when not in use and avoid background TV.
		2–5 yrs		For children 2 to 5 yrs., limit routine or regular screen-time to less than 1 h per day.		
Children and Screens: Institute of Digital Media and Child Development, 2017 https://www.childrenandscreens.com/findings/ [27]	USA			Limiting TV exposure (especially background TV) before the age of 2 yrs.		Plan a bedtime that allows for adequate sleep. Use a bedtime routine that includes calming activities and avoids electronic media use. Limit media use in the hour or two before bedtime. Turn off electronic media devices in the evening and charge them in a central location outside bedrooms. Be a healthy sleep and media role model for your child or adolescent. Remove all electronic media from your child or teen’s bedroom, including TVs, video games, computers, tablets, and cell phones. Turn mobile devices off during class and other learning activities. Turn TV off during schoolwork time. Turn TV off when no one is watching.
		4–13 yrs		Limit total screen-time, TV watching, video game playing, and computer use (excluding computer use for school homework, when applicable) no more than 7 h per week.		
		Newborns 0–3 months			14–17 h	
National Sleep Foundation’s updated sleep duration recommendations: final report. 2015 [21]	USA	Infants (4–11 months)			12–15 h	
		Toddlers (1–2 yrs)			11–14 h	
		Preschoolers (3–5 yrs)			10–13 h	
		School-aged children (6–13 yrs)			9–11 h	
		Teenagers (14–17 yrs)			8–10 h	
New Zealand Ministry of Health. Sit Less, Move More, Sleep Well: Active Play Guidelines for Under-Fives. 2017 [29]	New Zealand	0–1 yr		Discourage screen-time	Babies (birth to 3 months) should have 14 to 17 h good-quality sleep every day, including daytime sleeps centred around their physical and emotional needs. Infants (4–12 months) should have 12 to 15 h good-quality sleep every day, including daytime sleeps, which will tend to decrease as they get closer to 1 yr of age.	Provide regular activity breaks to limit the amount of time a child spends sitting. Discourage screen-time for under-2-yr-olds and limit screen-time to less than 1 h. every day for children aged 2 yrs. or older – less is best! Limit time in equipment that restricts free movement. From birth, encourage regular, unrestricted floor-based play (tummy time), on a safe surface. Be a role model: reduce your own screen use. Replace TV time with reading time, story time or doing jigsaw puzzles together. Avoid having the TV playing in the background. Remove the TV completely or limit having it on until the children have gone to bed. Do not have screens in (any) bedrooms. Set limited viewing times for all screens. Store DVDs, consoles, tablets and electronic games out of sight. Break up long car journeys with regular stops (preferably at least once an hr), removing under-fives from their capsule/car seat at each stop. Encourage toddlers and preschoolers to walk instead of being in a pushchair.
		1–2 yrs	At least 3 h every day for toddlers and preschoolers, spread throughout the day.	Discourage screen-time.	Toddlers (1 to 2 yrs. inclusive) should have 11 to 14 h of good-quality sleep every day, including at least one daytime sleep.	
		2 yrs. and older	At least 3 h every day for toddlers and preschoolers, spread throughout the day.	Less than 1 h every day	Preschoolers (3 to 4 yrs. inclusive) should have 10 to 13 h of good-quality sleep every day, with consistent bedtimes and wake-up times.	
U.K. Chief Medical Officers’ Physical Activity Guidelines, 2019 [22, 30].	UK	0–1 yr	Infants should be physically active several times every day in a variety of ways, including interactive floor-based activity, e.g. crawling. For infants not yet mobile, this includes at least 30 min of tummy time spread throughout the day while awake (and other movements such as reaching and grasping, pushing and pulling themselves independently, or rolling over); more is better. Tummy time may be unfamiliar to babies at first, but can be increased gradually, starting from a min or two at a time, as the baby becomes used to it. Babies should not sleep on their tummies.			
		1–2 yrs	Toddlers should spend at least 180 min (3 h) per day in a variety of physical activities at any intensity, including active and outdoor play, spread throughout the day; more is better.			
		3–4 yrs	Preschoolers should spend at least 180 min (3 h) per day in a variety of physical activities spread throughout the day, including active and outdoor play. More is better; the 180 min should include at least 60 min of MVPA.			
		5–18 yrs	Children and young people should engage in MVPA for an average of at least 60 min per day across the week. This can include all forms of activity such as physical education, active travel, after-school activities, play and sports.			Children and young people should engage in a variety of types and intensities of physical activity across the week to develop movement skills, muscular fitness, and bone strength. Children and young people should aim to minimize the amount of time spent being sedentary, and when physically possible should break up long periods of not moving with at least light physical activity.
U.S. Department of Health and Human Services. Physical Activity Guidelines for Americans. 2nd ed. 2018 [31]	USA	3–5 yrs	Although the specific amount of activity needed to improve bone health and avoid excess fat in young children is not well defined, a reasonable target may be 3 h per day of activity of all intensities: light, moderate, or vigorous intensity.			The Advisory Committee did not review evidence for children younger than age 3 yrs.
		6–17 yrs	Children and adolescents ages 6 through 17 yrs. should do 60 min (1 h) or more of MVPA daily. Aerobic: Most of the 60 min or more per day should be either moderate- or vigorous-intensity aerobic physical activity and should include vigorous-intensity physical activity on at least 3 days a week. Muscle-strengthening: As part of their 60 min or more of daily physical activity, children and adolescents should include muscle-strengthening physical activity on at least 3 days a week. Bone-strengthening: As part of their 60 min or more of daily physical activity, children and adolescents should include bone-strengthening physical activity on at least 3 days a week.			
World Health Organization. WHO Guidelines on Physical Activity, Sedentary Behaviour and Sleep for Children Under 5 Years of Age. 2019 [18]	Global	0–1 yr	Be physically active several times a day in a variety of ways, particularly through interactive floor-based play; more is better. For those not yet mobile, this includes at least 30 min in prone position (tummy time) spread throughout the day while awake.	Not be restrained for more than 1 h at a time (e.g. prams/ strollers, highchairs, or strapped on a caregiver’s back). Screen-time is not recommended. When sedentary, engaging in reading and storytelling with a caregiver is encouraged.	Have 14–17 h (0–3 months of age) or 12–16 h (4–11 months of age) of good quality sleep, including naps.	
		1–2 yrs	Spend at least 180 min in a variety of types of physical activities at any intensity, including MVPA, spread throughout the day; more is better.	Not be restrained for more than 1 h at a time (e.g., prams/ strollers, highchairs, or strapped on a caregiver’s back) or sit for extended periods of time. For 1-yr-olds, sedentary screen-time (such as watching TV or videos, playing computer games) is not recommended. For those aged 2 yrs., sedentary screen-time should be no more than 1 h; less is better. When sedentary, engaging in reading and storytelling with a caregiver is encouraged.	Have 11–14 h of good quality sleep, including naps, with regular sleep and wake-up times.	
		3–4 yrs	Spend at least 180 min in a variety of types of physical activities at any intensity, of which at least 60 min is MVPA, spread throughout the day; more in better.	Not be restrained for more than 1 h at a time (e.g. prams/ strollers) or sit for extended periods of time. Sedentary screen-time should be no more than 1 h; less is better. When sedentary, engaging in reading and storytelling with a caregiver is encouraged.	Have 10–13 h of good quality sleep, which may include a nap, with regular sleep and wake-up times.	
World Health Organization. Global Recommendation on Physical Activity for Health. 2010 [19]	Global	5–17 yrs	At least 60 min of MVPA daily. Most of daily physical activity should be aerobic. Vigorous-intensity activities should be incorporated, including those that strengthen muscle and bone, at least 3 times per week.			

Note: *Min* minutes; *Hrs* hours; *Yrs* years; *MVPA* moderate-to-vigorous intensity physical activity

Many studies have shown that health benefits accumulate with each additional movement guideline met (i.e., physical activity, sedentary behaviour/screen-time, sleep) [9, 32–37]. Unfortunately, among Canadian children, only 13% of 3–4-year-olds [9], 17% of 5–17 year-olds [9, 38], and 3% of 11–15 year-olds [34] adhere to the Canadian 24-Hour Movement Guidelines [1, 2]. Similar low adherence to healthy movement behaviour recommendations among children and youth have been reported in samples from Australia [39], Belgium [40], Mozambique [41], New Zealand [42], Sweden [43], the United Kingdom [44], the United States [37, 45, 46], and even lower adherences in China [35], Singapore [47] and South Korea [36]. Clearly, the need to promote healthy movement behaviours among children and youth is a world-wide public health priority.

The promotion of increased physical activity, decreased sedentary behaviour, and good sleep hygiene (e.g., routines and practices that are conducive to sleeping well) can occur in multiple settings, such as in childcare [48], at school [49], and within the community [50, 51]. Children and youth also spend considerable time with siblings and in the care of their parents or legal guardians, suggesting family-based initiatives are likely central to improving movement behaviours at the population level. Family influence (e.g., parents, guardians, siblings) on physical activity [52], sedentary behaviour [53], and sleep [54] has received considerable research attention. Family influence on a child can take many forms, such as modelling [55], encouragement [56], logistical support [57], rules and restrictions [54], co-participation [58], and spectating/supervision [59]. Parenting practices that influence child and youth health behaviours include components of responsiveness (providing encouragement and autonomy), structure (providing social and physical environments) and demandingness (restrictive and punitive practices) [60, 61]. Family systems and behaviours are complex; the family unit is in a constant cycle of interactions among members, with evolution and change being inherent [62–64]. Family membership can vary considerably, as can roles and responsibilities; however, parents (or legal guardians) have unique legal responsibilities, liabilities, and decision-making authority.

With constantly changing environments (including practices, policies, social norms, built features, technology) at home, childcare centres, schools and in communities, coupled with the new paradigm of integrated movement behaviours [1, 2], the challenges for achieving healthy movement behaviours can be overwhelming for families and those who support them (e.g., public health professionals, health care providers, teachers, policymakers). Accordingly, a comprehensive process was initiated in Canada to develop and release an *Consensus Statement on the Role of the Family in the Physical Activity, Sedentary, and Sleep Behaviours of Children and Youth* (0–17 years) to consolidate related evidence, highlight key facts, and provide guidance to families and those who support families on how to promote regular healthy movement behaviours of children and youth. The process and this manuscript were modelled after similar initatives that proved to be successful and well-received by the academic community (all cited > 100 times according to Scopus [1, 2, 65–69]). The purpose of this manuscript is to describe the process used to develop the *Consensus Statement* and present, explain, substantiate, and discuss the final *Consensus Statement.*

## Methods

Active Healthy Kids Canada and ParticipACTION (Canadian not-for-profit organizations) have been producing Canadian Report Cards on Physical Activity for Children and Youth since 2005 [70–74]. These Report Cards provide a public-facing synthesis of the best available evidence of how Canada is doing across a range of indicators related to movement behaviours, and the settings and sources of influence that serve as barriers or enablers of these behaviours. In recent years, the Report Card has also served as an effective dissemination and distribution vehicle for additional “knowledge products”. These knowledge products serve as complementary adjuncts to the Report Card but are also stand-alone resources. Previous knowledge products have included the Global Matrix in 2014 [75], the *Active Outdoor Play Position Statement* in 2015 [76], the Canadian 24-Hour Movement Guidelines for Children and Youth in 2016 [1], and the *Expert Statement on Physical Activity and Brain Health in Children and Youth* in 2018 (https://www.participaction.com/en-ca/resources/report-card). This *Consensus Statement on the Role of the Family in the Physical Activity, Sedentary, and Sleep Behaviours of Children and Youth* is the latest in this list of knowledge products and is contained within the *2020 ParticipACTION Report Card on Physical Activity for Children and Youth* [77]*.*

This project was initiated in partnership by ParticipACTION and the Healthy Active Living and Obesity Research Group (HALO) at the Children’s Hospital of Eastern Ontario (CHEO) Research Institute. The development and release of the *2020 Consensus Statement* included the establishment of a multi-disciplinary Expert Panel, completion of six reviews (three literature, two scoping, and one review of reviews), custom data analyses from Statistics Canada’s Canadian Health Measures Survey (CHMS), integration of related research identified by Expert Panel members, a stakeholder consultation process, establishment of consensus, and the development of a media, public relations, communications and launch plan.

To establish the Expert Panel, the project leaders (MST, LMV, RER) created a list of experts in the field of children’s movement behaviours, family health research, advocates and thought leaders in these areas. Email invitations were used to recruit Expert Panel members. While the project was Canadian led and Canadian focussed, two international candidates with prominent research profile in this area were also invited to join the Expert Panel and provide an international perspective.

The six reviews were undertaken to help inform the *Consensus Statement* after initial consultation with the multi-disciplinary Expert Panel. The first three reviews (reviews #1–3) were conducted 9 months prior to the last three reviews (reviews #4–6). Findings from reviews #1–3 were used to determine whether there was sufficient evidence on family and children’s movement behaviours, and whether the Expert Panel was in a position to write a consensus statement on this topic. Members of the Expert Panel reviewed the findings from reviews #1–3 and agreed to proceed with reviews #4–6. Team members responsible for conducting reviews #1–3 differed from those responsible for conducting reviews #4–6. This approach was taken in order to draw on the different expertise and experience of team members and to share workload burden. Nevertheless, all reviews followed the basic PRISMA statements for reporting reviews [78, 79]. The overall purposes of the systematic literature reviews were to: i) examine the breadth of existing research on family and children and youth’s physical activity, sedentary, and sleep behaviours, and categorize articles by type of familial influence, ii) synthesize findings of frequently researched themes within each movement behaviour, iii) provide an overview of family-systems based theories used in the disciplines of psychology, sociology, and/or social work; iv) provide an overview of family-based theories or family behavioural models used to understand child health behaviour change, v) identify significant correlates of parental support of child and youth healthy 24-h movement behaviours, and vi) evaluate the efficacy of child and youth interventions focused on changes in physical activity, sedentary and/or sleep behaviours, where at least one parent was involved in the intervention. Therefore, the six reviews investigated the evidence on: i) family influences and characteristics and children and youth’s physical activity behaviours (review #1); ii) family characteristics and children and youth’s sedentary behaviours and screen-time behaviours (review #2); iii) family characteristics and children and youth’s sleep behaviours (review #3); iv) family systems approaches applied to understand 24-h movement behaviours among children and youth (review #4); v) correlates of parental support of child and youth 24-h movement behaviours (review #5); and, vi) family-based interventions on children and youth 24-h movement behaviours (review #6).

To better understand the relationships between family structure and child movement behaviours in Canada, custom analyses from Statistics Canada’s CHMS [80] were requested. The CHMS is a large, nationally representative repeated cross-sectional survey of Canadians aged 3–79 years that gathers directly measured health data on Canadians in two-year cycles. Data are gathered through a detailed health interview and a visit to a mobile examination centre where several direct measures of health are taken (e.g., anthropometry, physical fitness, accelerometry-measured physical activity and sedentary behaviour, blood pressure; and biospecimen collection for analysis of chronic and infectious disease biomarkers, nutritional status, and environmental exposures). Complete details of the CHMS are available at https://www23.statcan.gc.ca/imdb/p2SV.pl?Function=getSurvey&SDDS=5071). Analyses obtained included merged data from cycles 1–5 (2007–2017) that described relationships between accelerometer-measured variables (including average minutes per day of moderate-to-vigorous physical activity [MVPA], light physical activity, sedentary time, and step counts) by number of children in the household and single- versus two-parent households, by age groups (5–11 [cycle 2–5 only] and 12–17 years) and sex. Similar analyses were also obtained for self-reported sleep duration, screen-time and physical activity domains (active transportation, recreational physical activity, occupational/household physical activity); however, adult and youth results are from cycles 4–5 combined only, and children and preschoolers results are from cycles 2, 3, 4, and 5 combined. Screen-time results included cycles 2, 3, 4 only due to a response option change in cycle 5 that makes it non-comparable to previous cycles. The CHMS also samples same-family parent-child dyads in approximately one fifth of the sample in each cycle. Recently, findings from the dyad file comparing parent-child physical activity, screen-time and fitness were also gathered.

A small Steering Committee with four representatives (RER, MDG, LMV, MST) from the larger Expert Panel was formed to organize the work plan, complete the reviews, draft the *Consensus Statement* and related manuscript, and report back to the multi-disciplinary Expert Panel. Expert Panel members were selected based on their research, practice, and/or professional experience related to the 24-Hour Movement Guidelines [1, 2] and/or family healthy movement behaviours and/or involvement with organizations interested in children and youth in the context of the family, with stratification across movement behaviours (i.e., physical activity, sedentary behaviours, sleep) and attention to geography, gender and career-stage diversity. The Expert Panel first met on May 16–17, 2019 in Toronto, Canada. The objectives of this first meeting were to meet and network; explain the history and purpose of the ParticipACTION Report Card and how this *Consensus Statement* relates to the Report Card; present background efforts and research; brainstorm content/focus; and discuss the form, format, timelines and logistics of the *Consensus Statement* development. The Expert Panel was also invited to provide suggestions for additional panel members to fill any knowledge or expertise gaps. Four additional members were invited and all accepted invitations to be involved in the subsequent meeting and related project tasks.

The group met for a follow-up meeting in January 23–24, 2020 in Toronto, Canada, where results of the reviews and custom analyses were presented, other research evidence was discussed, a glossary and a conceptual model were developed, initial communication and dissemination strategies were planned, and organization for a stakeholder consultation was initiated. In addition, a first draft of the *Consensus Statement* was created by the Expert Panel and finalized after the meeting through an iterative process to achieve consensus.

**Fig. 1:**
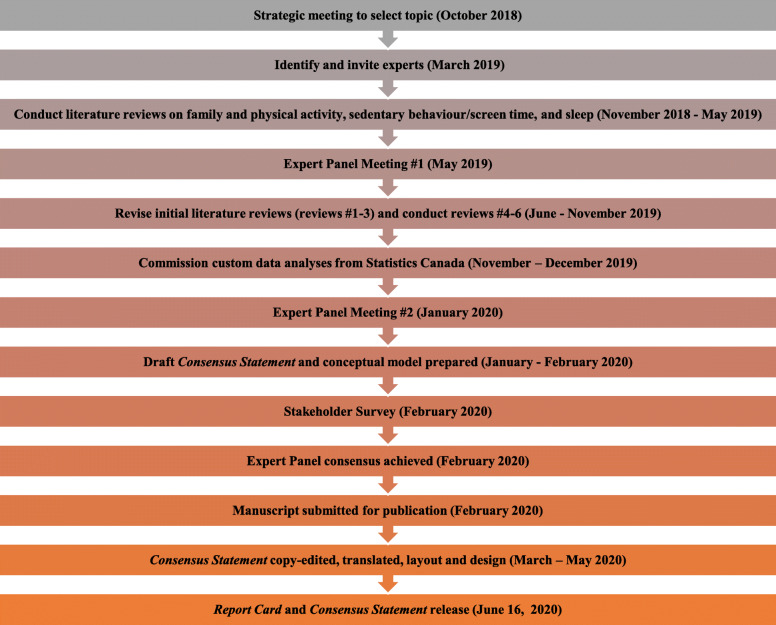
Overview of timelines and key components of the Consensus Statement development

An online survey was developed and administered to solicit assessments and comments from stakeholders on the draft *Consensus Statement*. The survey was designed to seek assessments of the clarity and level of support for the various components of the *Consensus Statement* (title, summary, evidence, conceptual model, recommendations) separately. A copy of the survey is provided as Additional File Survey. Quantitative data were gathered from five-point rating scales that went from strongly disagree to strongly agree and qualitative data were gathered through open comment boxes for each component. The survey was disseminated through the networks of Expert Panel members. The survey was only live for 4 days because of time restrictions related to a set release date. The Steering Committee collated the responses and thematic analysis was undertaken to identify prominent or emerging themes to help better shape the wording, content and utility of the *Consensus Statement*. The *Consensus Statement* was revised accordingly. Finally, the revised *Consensus Statement* was circulated to the entire Expert Panel for final comment, revisions, and sign-off. The final *Consensus Statement* was translated into French and both the English and French versions went through a creative design process by ParticipACTION for inclusion in the 2020 ParticipACTION Report Card. A summary overview of the timelines and key components of the *Consensus Statement* is provided in Fig. 1.

## Results

### Establishment of expert panel

Twenty-one candidates were initially invited to join the Expert Panel, including two international delegates, and information was provided on the commitement required. Fourteen of the invitations were accepted. Reasons for invitation decline were either too many concurrent commitments (*n* = 4) or no response received (*n* = 4). One international delegate declined and the other did not respond. At the first Consensus Panel meeting four additional candidates were identified and invited to join. Invitations were sent and all four accepted. The final Expert Panel composition included 17 experts. Two research staff (KB, MP) provided significant support to the Expert Panel.

An overview of the evidence obtained from the six reviews, analyses from Statistics Canada’s Canadian Health Measures Survey, results of the stakeholder consultation, final conceptual model, presentation of final *Consensus Statement*, and release and evaluation plans are provided below.

### Family and children and Youth’s movement Behaviours (reviews #1–3)

Inclusion and exclusion criteria for reviews #1–3 are summarized in Additional File Table S1. Article titles and abstracts for each review were screened and categorized into themes. A summary of the search process, the findings organized by theme, and all references are provided in Additional Files Review #1, Review #2, and Review #3, respectively. Search details and key findings of the most frequently researched family characteristics related to physical activity (*physical activity modelling*, *parental emotional support*, *sociodemographic factors*, and *parental beliefs, attitudes and knowledge*), sedentary behaviour (*parental behaviours*, *sociodemographic factors,* and *household practices*) and sleep (*household practices*, *sociodemographic factors*, and *family environment*) are outlined in Table 2.

**Table 2:** Search details and key findings of reviews #1–3

	Timeline	Databases Used	Search Terms	Number of reviewers	Number of articles included
**Review #1 – Physical activity**	Nov. 2018 – Nov. 2019	Ovid MEDLINE, Embase	**Family:** Family characteristics: family relations; parent-child relations; father-child relations; mother-child relations; parenting; parenting styles; parental behaviour; parents; fathers; mothers; siblings; sibling relations; bother; sister; attitudes; perception; attitude to health; health behaviours. **Physical activity:** Physical activity; exercise; sport; physical exertion; aerobic; active transportation; active neighbourhood	2	739
Key findings	**Physical activity modelling (*n*** **= 359):** • A review of reviews (*k* = 18; 375 quantitative studies) demonstrated that family behaviours, such as modelling or co-participation, had a stronger positive relationship with children’s physical activity than more general family household practices and beliefs [81]. • Parental physical activity was positively associated with their children’s physical activity levels (*k* = 10) [53]. • Longitudinal studies that followed families over a period of 5 years (from the time children were 5–6-year-olds to 10–12-year-olds) demonstrated that maternal physical activity [82], paternal reinforcement [82], and sibling co-participation [83] were positively associated with MVPA among pre-adolescent and adolescent boys and girls; however, only girls’ MVPA was negatively associated with maternal sedentary behavior [83]. • Findings from a systematic review of cross-sectional studies (*k* = 10) demonstrated that both parents’ modelling behaviours were positively associated with their children’s (3–19 years) physical activity behaviors [84]. • Parental modelling of active transportation was also important, such that it reduced the negative association between perceived safety and youth’s active transport [85], which is a significant barrier for active transportation for children and adolescents [86–89]. **Parental emotional support (*n*** **= 234):** • Systematic reviews on children ([90] *k* = 3, [79]*k* = 11) and adolescents ([90] *k* = 4, [91]*k* = 75, [92] *k* = 52) found that familial and parental social support were consistently positively associated with increased physical activity in cross-sectional and longitudinal studies. For adolescent girls, this effect was slightly stronger when support came from the mother (*r* = .22) compared to support from both parents (*r* = .19) [92]. When examining studies that included both genders, more consistent positive associations were found when support came from the entire family or from both parents rather than each family member separately (e.g., mother, father, siblings) [91]. • Mixed results were found when examining the relationship between parental encouragement/support and outdoor play in children, with studies showing either positive or null relationships (*k* = 11) [93]. **Sociodemographic factors (*n*** **= 172):** • Parental education and income, but not employment status, were associated with children’s physical activity [93]. • A systematic review (*k* = 12) indicated that children of higher educated parents engaged in less outdoor play [93]. This result was most consistent when maternal education was high (*k* = 6) [93]. This finding is also supported by independent studies and other systematic reviews, whereby children of mothers with a graduate or professional degree were more likely to be inactive [94] and less likely to use active transportation (*k* = 9) [95]. • Association between household income and physical activity varied depending on the type of physical activity. For outdoor play, a systematic review (*k* = 5) showed that there was no association between household income and time spent in outdoor play [93]. • Findings from independent studies show that higher household income was positively associated with children’s leisure physical activity [94] and engagement in sports [96, 97]; however, evidence from a systematic review (*k* = 21) showed that higher household income was negatively associated with use of active transportation [95]. **Parental beliefs, attitudes, and knowledge (*n*** **= 134):** • Parental beliefs about safety of physical activity-related activities and their neighbourhood were shown to be important for outdoor play [88, 98], leisure [86, 99], and organized physical activity [99]. • Parental attitudes toward physical activity have been shown to be associated with youth’s physical activity; children of parents who believe physical activity is important are more likely to engage in organized [99, 100] and leisure [99, 101] physical activities. • Parental modelling [102–104] and child stimulation of healthy dietary intake [104] were positively associated with children and youth physical activity levels.				
**Review #2 – Sedentary behaviour**	Nov. 2018 – Nov. 2019	Ovid MEDLINE, Embase	**Family:** Same search terms outlined in review #1 **Sedentary behaviour:** Sedentary lifestyle; sedentary behaviour; inactivity; physical inactivity; sitting; laying; TV; video games; Internet; computer; screen; smartphone; iPad; apps; mobile applications; social media; Facebook; YouTube; Twitter; Snapchat; Instagram; Pinterest; screen viewing; screen-time.	2	313
Key Findings	**Parental behaviours (*n*** **= 104)** • Substantial evidence showed that parents’ sedentary behaviour and screen-time were positive correlates of children’s sedentary behaviour and screen-time [105–119]. • Studies that specifically examined mothers’ screen-based behaviours showed a positive correlation between mother and child’s screen-time [108, 120, 121]. • Some evidence showed that co-viewing with parents was associated with increased screen-time in children [121, 122]. **Sociodemographic factors (*n*** **= 92)** • Parental education specifically and socioeconomic status more generally were negatively associated with sedentary behaviour and screen-time [123–134]. • Children and youth from families with higher socioeconomic status, but living in low- and middle-income countries, reported higher screen-time use than those from families with lower socioeconomic status [135]. • Mixed findings regarding how the presence, number, or type (younger vs., older) of sibling are associated with children’s sedentary behaviour and/or screen-time [121, 136–138]. **Household practices (*n*** **= 84)** • Children who lived in a family with screen-time restrictions spent less time engaged in screen-based behaviours [126, 133, 139–149]. • Electronics in children and youth’s bedrooms were associated with increased time spent in screen viewing [110, 111, 134, 150–156]. • Number of electronics in the household was a positive predictor of screen viewing [144, 157]. • Eating meals in front of the TV was linked with greater sedentary behaviour and screen viewing [134, 144, 157–159].				
**Review #3 – Sleep**	Nov. 2018 – Nov. 2019	Ovid MEDLINE, Embase	**Family:** Same search terms outlined in review #1 **Sleep:** Sleep duration; sleep quality; sleep timing; sleep routine; sleep hygiene; sleep habits; sleep patterns; sleep efficiency; sleep behaviour; sleep interruption; sleep schedule.	2	189
Key Findings	**Household practices (*n*** **= 81):** • Evidence showed that good sleep hygiene (e.g., regular bedtimes, read at bedtime, or fall asleep in bed) was positively associated with sleep duration [160–166], sleep quality [167], and negatively associated with sleep latency (the amount of time it takes to go from being fully awake to sleeping) [168]. • Implementing consistent bedtime routines (e.g., bath, massage, brushing teeth, and quiet activities) has been shown to be beneficial for sleep onset latency, frequency and duration of nighttime awakenings, and sleep consolidation [169]. • Electronic use was associated with shorter sleep duration [162, 170–172], and accessibility to electronics in the child’s bedroom was adversely associated with sleep outcomes (e.g., shorter sleep duration, delayed bedtime, increased daytime sleepiness) [164, 173–177]. • Parental presence until sleep onset (e.g., holding, rocking, feeding) was consistently associated with shorter sleep duration [170, 176, 178, 179]. • Sharing a bedroom or bed with siblings/parents was linked with adverse sleep outcomes such as shorter sleep duration, poor sleep quality, night awakenings, and daytime sleepiness [163, 180–183]. • Presence of positive parenting practices (e.g., eating dinner together, limiting screen-time, encouraging social maturity) was associated with longer sleep durations [184–187]. **Sociodemographic factors (*n*** **= 54):** • Higher maternal education [172, 177, 188] and maternal employment [189–191] were associated with poor sleep outcomes (e.g., shorter sleep duration, sleep problems). • Less optimal sleep outcomes (e.g., decreased sleep duration, delayed sleep onset) were evident in low socioeconomic families [192–196], and better sleep hygiene was more common in high socioeconomic families [177, 197]. **Family environment (*n*** **= 29):** • More chaotic, disorganized, and irregular family environments were associated with negative sleep outcomes, including lower levels of sleep quality [160, 198], bedtime resistance and inconsistency [199], sleep problems (e.g., sleep assistance, daytime sleepiness) [200, 201] and delayed sleep onset [202]. • Good family relationships were associated with better sleep quality [167], whereas poor family relationships (e.g., emotional insecurity, low parental hardiness, marital insecurity) were associated with short sleep duration, poor sleep quality, and greater sleep problems [175, 203–205].				

### Family systems theory, correlates of parental support for child and youth movement Behaviours, and family-based interventions (reviews #4–6)

Inclusion and exclusion criteria for reviews #4–6 are summarized in Additional File Table S2. A summary of the search process for reviews 4–5, and all references can be found in Additional Files Review #4, and Review #5, respectively. The search process for review #6 can be found in Additional File Figure S1 (all references for review #6 are cited directly in the manuscript). Key findings are highlighted in Table 3. Review #4 highlights the dynamic and fluid nature of the family system. While family systems have been incorporated into many schematics applied to child movement behaviours, the evaluation of these family systems approaches is scarce in the empirical literature. Review #5 found few sociodemographic correlates of parental support for movement behaviours. Almost all of this research has been conducted on physical activity and not sedentary and sleep behaviours. Age was negatively associated with support. There is evidence that parenting cognitions about support (attitudes, perceived control, intentions) are key correlates of child and youth physical activity, while planning to support one’s child is important for physical activity and sleep. Finally, review #6 showed that considerable family intervention research is accumulating for child and youth physical activity promotion, followed by family involvement in screen-time interventions and less research on family involvement in child and youth sleep. Physical activity research has noted general effectiveness of family interventions, while the efficacy of family-based screen-time interventions is less conclusive and the efficacy of family-based sleep interventions unknown. There are few moderators that have been identified for these interventions, but merely providing educational material for families may be ineffective in changing child and youth physical activity; instead, the current evidence shows that implementing behavioural approaches with families, including planning and setting goals, may be successful.

**Table 3:** Search details and key findings of reviews #4–6

	Timeline	Databases Used	Search Terms	Number of reviewers	Number of articles included
**Review #4 – Family systems approaches applied to understand 24-h movement behaviours among children and youth**	Sept. – Nov. 2019	Academic search premier, CINAHL Complete, Cochrane Central Register of Controlled Trials, Health Source: Nursing/Academic edition, Humanities and Social Sciences Index (H.W. Wilson), MEDLINE with Full text, PsychARTICLES, and PsycINFO	Family systems theory; family-based interventions; childhood obesity; 24-h movement behaviors; family behaviour change; child behaviour change; family and child physical activity, sedentary and sleep behaviours; Family Ecological Model; family characteristics; family relations; parent-child relations; father-child relations; mother-child relations; parenting; parenting styles; parental behaviour; parents; fathers; mothers; siblings; sibling relations; bother; sister; attitudes; perception; attitude to health; health behaviours.	2	20
	**Origin and development of family systems theory:** • Family systems theory is the primary theoretical approach to understanding and intervening upon family behaviour [206]. The approach has considerable overlap with Attachment Theory from developmental psychology [207] and highlights that the family is in a constant cycle of interactions with inherent evolution and change. Recurring challenges, reorganization, and changes are inevitable parts of the family life cycle that must be considered [62, 63]. Interventions should likely target the family unit rather than the child in isolation. • Barnhill [208] advanced the Family Systems Theory by specifying eight dimensions: individuation vs. enmeshment, mutuality vs. isolation, flexibility vs. rigidity, stability vs. disorganization, clear vs. unclear/distorted perception, clear vs. unclear/distorted communication, role reciprocity vs. unclear roles of role conflict and clear vs. diffuse or breached generational boundaries. These eight dimensions of healthy family functioning can be grouped into four mutually causal constructs that include identity processes, change, information processing, and role structuring. • Bronfenbrenner [209] aextended family systems to a larger environment and proposed three systems that impact child development: mesosystems, exosystems, and chronosystems. Mesosystems incorporate the developmental processes that occur outside of the family home, such as experiences at school. Exosystems include parents’ social networks, the workplace, and friend circles. The chronosystem describes life transitions. Bronfenbrenner highlights that these contexts do not operate independently of one another and instead interact with each other. **Family systems theory adapted for health behaviour changes:** • Davison and colleagues [210] presented the revised family systems ecological model, which incorporates factors that affect parenting cognitions and behaviours such as intra-familial (educational and cultural) backgrounds in addition to community factors (social connectedness to neighbours and friends). The model suggests that parenting for child health behaviour includes: i) knowledge and beliefs about health behaviour; ii) modelling of health behaviours; iii) shaping of child behaviour through rewards and punishments; and, iv) provisions for the child to engage in the health behaviour. • Myoungock and Whittemore [211] proposed the family management style framework for childhood obesity interventions. It focuses on family functioning and parental perspectives associated with the management of children’s health behaviours. • Kitzman-Ulrich and colleagues [212] proposed a Family Systems Theory framework for evaluating family-level variables and positive (authoritative) parenting styles that lead to improvements in youth health behaviours (physical activity, dietary and weight-loss behaviours). • Nowicka and Flodmark [213] presented a family therapy model for treating childhood obesity known as Standardized Obesity Family Therapy (SOFT). The SOFT model integrates Family Systems Theory and elements used in focused solution development such as creating expectations for change, establishing goals for therapy and defining potential solutions. • The Levels of Interacting Family Environmental Subsystems (LIFES) framework incorporates theoretical concepts from ecological systems and Family Systems Theory [214], highlighting that child health behaviours are influenced by both child factors and factors existing within the family (i.e., parents’ behaviours, parenting practices and family functioning). **Studies that have tested family systems approaches with interventions (*n*** **= 2):** • Both studies applied the SOFT model [213] to examine integrating Family Systems Theory and solution-focused theory (i.e., focusing on solutions, preferred future and goals as opposed to the problem and its cause) into family therapy sessions for adolescents and their families. • This treatment model focused n engaging the whole family in the obesity treatment process with an emphasis on encouraging physical activity (60 min per day) and reducing sedentary screen-time (less than 2 h per day). Both interventions were effective in decreasing child body mass index in comparison to control conditions; unfortunately, no behavioural outcomes were assessed in these trials so the complete understanding of the effectiveness of the intervention is limited [215, 216].				
	**Timeline**	**Databases Used**	**Search Terms**	**Number of reviewers**	**Number of articles included**
**Review #5 – Correlates of parental support of child and youth 24-h movement behaviours**^a^	Sept. – Nov. 2019	Academic search premier, CINAHL Complete, Cochrane Central Register of Controlled Trials, Health Source: Nursing/Academic edition, Humanities and Social Sciences Index (H.W. Wilson), MEDLINE with Full text, PsychARTICLES, PsycINFO, SPORTDiscuss, and ScienceDirect	Parent; caregiver; mother; father; parental support; parenting practices; parenting strategies; parenting behaviours; parental correlates; child; adolescent; physical activity; exercise; sport; physical exertion; aerobic; active transportation; active commute; park; outdoor; outdoor play; active lifestyle; active neighbourhood. Prospero# CRD42020154439	3	25 (22 unique datasets)
Key Findings	**Composition of studies and characteristics** • Sixteen studies focused on physical activity support (76%), with two studies focused on sleep support [54, 217] and four on sedentary behaviour or screen-time restriction [14, 218–220]. The majority of the studies used a cross-sectional design, and the targets of the support focused on both children and youth; only 27% focused on a single age group (i.e., children or adolescents). • Overall, parental support measurement was extremely varied, with most studies employing different instruments. However, the assessment of frequency of encouragement, logistical support, and co-participation activity were common elements across most of the measures and all measures reported generally sound indicators of reliability. **Correlates of parental support of child and youth physical activity** • Among potential demographic correlates of physical activity, only child age showed a reliable negative association (median *r* = −.13) [59, 218, 221–228], suggesting that older children and youth received less support than younger children and youth. • All social-cognitive factors assessed (attitudes, perceived control) were significant correlates of parental support in the medium effect size range [229]. Intention and planning to support were correlated with support behaviours in the large effect size range [229]. • Parenting style, in the form of authoritative parenting, had mixed results as a correlate of support for child/youth physical activity [225, 230]. • Neighbourhood safety [225] was a correlate of parental support in the small effect size range (median *r* = .16) [229]. **Correlates of parental restriction of child and youth sedentary behaviour** • Sedentary behaviour restriction studies included only two correlates. Child body mass index and parent gender had no association with sedentary behaviour measured as screen-time) restriction [219, 220]. **Correlates of parental support of child and youth sleep** • Studies on parental support of child and youth sleep found child age had a negative relationship with sleep support [54, 219]. Planning was the only cognitive and behavioural construct present in current studies and it showed a medium sized association (r = 0.24–0.50) with sleep support [54, 217, 218].				
	**Timeline**	**Databases Used**	**Search Terms**	**Number of reviewers**	**Number of articles included**
**Review #6** Family-based interventions on children and youth 24-h movement behaviours	Sept. – Nov. 2019	Academic search premier, CINAHL Complete, Cochrane Central Register of Controlled Trials, Health Source: Nursing/Academic edition, Humanities and Social Sciences Index (H.W. Wilson), MEDLINE with Full text, PsychARTICLES, PsycINFO, SPORTDiscuss, and ScienceDirect	Family-based; family mediators; family interventions; family moderators; behavior change strategies; children; youth; sleep; bedtime; sports; exercise; physical activity; sedentary lifestyle; sedentary behaviour; inactivity; physical inactivity; sitting; laying; TV; TV viewing; video games; Internet; computer; screen; smartphone; iPad; apps; mobile applications; social media; Facebook; YouTube; Twitter; Snapchat; Instagram; Pinterest; screen viewing; screen-time.	3	10
Key Findings	**Composition of studies and characteristics** • Eleven review articles met the inclusion criteria [49–51, 231–238]. Two-hundred and fifty studies (not independent) from the 11 review articles targeted child and adolescent physical activity behaviour. Four review articles incorporated 83 studies (not independent) that targeted screen-time and/or sedentary behaviours in addition to physical activity [49, 51, 234, 235]. Only one review that included 24 studies addressed child sleep behaviours [49], but this did not include the efficacy of these interventions. • The majority of reviews included studies published between 1980 and 2015. All reviews targeted families with school-aged children (5–12 years old) and eight of these also included adolescents (12–18 years old). Overall, the reviews targeted diverse samples whereby four reviews included families of low socioeconomic status [49, 231, 234, 235], and six reviews targeted ethnic minority populations [49, 50, 231, 232, 235, 238]. Four reviews included randomized controlled or quasi-experimental trials only [49–51, 231] and the other reviews also incorporated non-controlled trials, pilot or feasibility studies, and prospective cohort studies [232–235, 237, 238]. **Family-based physical activity intervention effectiveness** • Overall, nine reviews provided evidence of intervention effectiveness in physical activity [50, 51, 231–236, 238]. Two of these reviews provided a point estimate of intervention effectiveness using meta-analysis [50, 233]. There was considerable heterogeneity between the reviews (*d* = .29 with an average confidence interval between 0.17 and 0.42). This heterogeneity was further highlighted in the seven narrative reviews, where four concluded outcomes were inconclusive for family interventions [51, 231, 232, 238], one considered the evidence for behaviour change to be convincing [234], and the other two concluded that family interventions have been ineffective at changing child/youth physical activity [235, 237]. **Moderators of physical activity effectiveness** • Whether certain demographic profiles have been more responsive to intervention is currently mixed. The exception to this finding appears to be low socioeconomic groups, who have been successfully targeted in such interventions with positive physical activity outcomes [231, 234]. There is mixed evidence on whether following a formal theoretical framework has improved outcomes. Similarly, behaviour change strategies based on family-level support sessions to promote physical activity, and coordinated parent-child physical activity behaviour, have yielded mixed effects across reviews [235, 237, 238]. By contrast, there is consistent evidence that mere information-based education on the benefits of physical activity administered to children and their parents is an ineffective strategy to produce changes in physical activity. The largest and most recent review [233] found that behavioural strategies (goal setting, reinforcement) have been successful in invoking positive changes in physical activity. The best setting in which to promote family-based physical activity was inconclusive, but one review [233] found very limited evidence that primary care settings were a useful context in which to intervene. Similarly, delivery modes for the intervention were appraised as both successful and with mixed findings across face-to-face, distance-based (e.g., telephone), and larger group-based settings. **Family-based screen-time intervention effectiveness** • Two of the reviews found the current evidence inconclusive [51, 235] and the third concluded that family-based interventions have been ineffective [236]. The current data set is too limited to address moderators of intervention effectiveness with any certainty. There is preliminary evidence that families of low socioeconomic status may be responsive to intervention [231], and that merely providing information on the harms of sedentary behaviour may be ineffective [231, 235]. There appears to be equally mixed findings on how best to deliver these interventions with heterogeneity across face-to-face, distance, and group interventions. **Family-based sleep intervention effectiveness** • No reviews overviewed the effectiveness of sleep interventions for children and youth.				

^a^Full details on the physical activity portion of this review can be found in Rhodes, R.E., Perdew, M. & Malli, S. (in press). Correlates of Parental Support of Child and Youth Physical Activity: A Systematic Review. International Journal of Behavioral Medicine

### Evidence from the Canadian health measures survey (CHMS)

The results from the custom CHMS accelerometer analyses are presented below. In essence they show no significant differences in daily MVPA for boys or girls, of any age, based on number of siblings in the household. One exception is that for boys 12–17 years of age, having siblings had a positive association with physical activity. No differences in daily MVPA in 5-to-17 year-old children and youth according to a single- versus two-parent household structure; however, for 3-to-4 year-olds there is an effect whereby those living in households with two parents are more active than those living in households with one parent. There were no significant differences in the proportion of children aged 3 to 11 years meeting the daily MVPA recommendation of ≥60 min according to number of siblings in the household or single- versus two-parent household.

CHMS data on self- or parental-reported movement behaviours were also available for active transportation, recreational physical activity, occupational/household physical activity, screen-time and sleep duration by number of siblings in the household and by single- versus two-parent household. For children aged 3 to 4 years and for youth aged 12 to 17 years, there were no significant differences for any variables by number of siblings or parents. For children aged 5 to 11 years, the following significant differences were observed:

- Girls with no siblings or one sibling participated in more organized sports, lessons, and leagues compared to girls with two or more siblings;
- Boys with two or more siblings participated in more unstructured play outside of school compared to boys with one sibling or less;
- Girls in two-parent households participated in more organized sports, lessons, and leagues compared to girls in one-parent households;
- Girls and boys from two-parent households had less screen-time compared to single-parent households; and,
- Boys from two-parent households had more sleep compared to boys from single parent households.

In addition to the custom analyses described above, recently published findings from the CHMS examined parent and child sedentary behaviour and physical activity in early childhood [239]. This study found that higher parental screen-time, sedentary time, light physical activity and MVPA were significantly associated with higher screen-time, sedentary time, light physical activity, and MVPA, respectively, of their children in this large representative sample of Canadian 3–5-year-olds. The strength of relationships did not differ between weekdays and weekend days, sons and daughters or mothers and fathers [239].

Recently published findings from the same family parent-child dyad file explored parent-child associations for physical activity and screen-time [112]. These findings demonstrated the parents’ MVPA (parental role modelling) was associated with that of the child, with each additional 20 min of parental MVPA being associated with an extra 5 min of child MVPA [112]. Parents’ sedentary time was also associated with daughters’ sedentary time on weekends, and sons’ after school. Supporting children to be active through enrolment in organized sports, leagues or lessons was also positively associated with a child’s MVPA. Parental role modelling and supporting children to be active had independent effects. This is important because in some cases, parental modelling may be an insufficient influence on the physical activity of their children because parental participation in physical activity by itself does not remove potential barriers to their child being active (e.g., providing transportation to an activity, learning new skills).

Custom dyad analyses using accelerometer data generally showed significant associations for MVPA and sedentary behaviour among mother-daughter dyads, and for MVPA among father-son dyads. Custom dyad analyses on measures of fitness also show significant associations between parents and children for cardiorespiratory fitness, muscular strength, and flexibility. Correlation analyses indicate that the relationship is stronger in:

- Father-daughter pairs for cardiorespiratory fitness and muscular strength;
- Father-son pairs for muscular strength and flexibility; and.
- Mother-son pairs for muscular strength and flexibility.

Collectively the results for the CHMS show that while number of siblings and single- versus two-parent households are not generally associated with 24-h movement behaviours, the behaviours (physical activity and screen-time) and characteritsics (physical fitness) of parents are associated with the behaviours and characteristics of their children. These findings align with those found in the various reviews described above where parental role modelling was consistently found to be an important correlate of children’s 24-h movement behaviours.

### Stakeholder survey results and subsequent modifications

Sixty-seven stakeholders responded to the survey in the 4 days it was live. The sample consisted of a wide mix of sectors, yet a particularly large concentration of researchers (6 nongovernment organizations, 1 family support, 10 education, 1 recreation, 2 childcare, 2 healthcare, 5 public health, 32 research, 5 government, 3 other). Further, stakeholders were from Alberta (*n* = 9), British Columbia (*n* = 18), Nunavut (n = 1), Ontario (*n* = 18), Prince Edward Island (*n* = 1), Quebec (*n* = 4), Saskatchewan (*n* = 1), and outside of Canada (*n* = 18).

Overall, stakeholders agreed with the *Consensus Statement*. Specifically, 78% of the sample thought the title was clearly stated, 96% indicated the summary was clearly stated, 82% of the sample reported they would use the *Consensus Statement*, 99% of the sample thought the evidence for the statement was clearly stated, 76% indicated the conceptual model was clearly presented, and 95% of the sample reported the recommendations were clearly stated. Overall, 88% of the sample reported that the *Consensus Statement* would be (very to moderately) important to their work, while only one stakeholder reported that it would not be important.

The survey respondents were engaged and provided many comments and suggestions. Most comments were minor editorial suggestions (which were made) or compliments. As noted above, the level of agreement was very high. Nevertheless, a few clusters of suggestions emerged, albeit from a small minority of respondents. First, there was some concern about the title – that it was unclear (*n* = 6) or confusing (*n* = 3). Changes were made to address these concerns. Second, there were several small suggestions about the conceptual model, and these were also incorporated in revisions. Third, four respondents commented that there was too much responsibility placed on the family, however this was before they reached the portion of the survey that dealt with the other sources of influence on the family (e.g., schools, community, health care, government). Nevertheless, additional clarification was added to improve clarity that although the *Consensus Statement* is focussed on the role of the family, the *Statement* also speaks to the importance of those who influence and can support families. Finally, two respondents suggested that a review be completed to ensure inclusive language, particularly with respect to children with a disability. This was also completed and revisions made accordingly.

### Final *Consensus Statement*, conceptual model, release, and evaluation plans

**Fig. 2:**
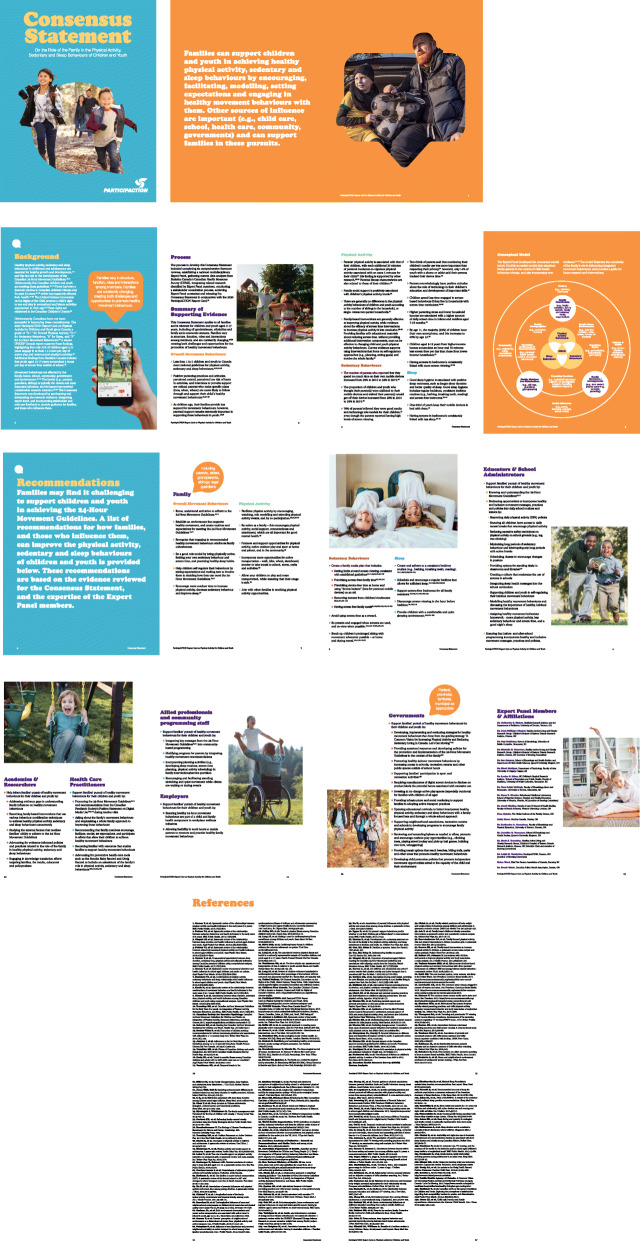
Consensus Statement on the Role of the Family in the Physical Activity, Sedentary, and Sleep Behaviours of Children and Youth. Document can be found at: https://participaction.com/consensus-statement

After meeting to discuss the findings of the six reviews and the custom analyses, performing multiple rounds of reviews and revisions, and incorporation of feedback through the stakeholder survey, the Expert Panel reached consensus on the final *Consensus Statement on the Role of the Family in the Physical Activity, Sedentary, and Sleep Behaviours of Children and Youth*. The *Consensus Statement* itself is a concise, public-facing, authoritative consolidation of research evidence. The statement includes brief background text, summary of the process, key summary points and sources of the aggregated evidence, a conceptual model consolidating the scope of the findings, followed by concise recommendations to anticipated audiences (e.g., family members, educators, health professionals, government, researchers). The *Consensus Statement* concludes with key support resources, acknowledgements and references. It was designed to be a foundation of credible evidence from which programs, strategies, campaigns, policies and practices can be initiated and supported. The *Consensus Statement* as a knowledge product within the 2020 ParticipACTION Report Card on Physical Activity for Children and Youth [85] was released on June 16, 2020. The final *Consensus Statement* can be found in Fig. 2 (see also: https://participaction.com/consensus-statement). The *Consensus Statement* is also available in French at https://www.participaction.com/fr-ca/ressources/bulletin-de-participaction.

To maximize the impact of the *Consensus Statement,* a public relations and dissemination plan was developed and led by ParticipACTION. The release of the *Consensus Statement* benefited greatly from the proactive distribution, public relations and media strategy prepared to maximize and optimize the reach and impact of the release of the ParticipACTION Report Card. Through the Report Card, the *Consensus Statement* reached relevant stakeholders (approximately 50,000 educators, health professionals, government, media, and researchers) across Canada. The general public (expectation of more than 300,000 Canadians) were reached directly through public-facing communications and social channels and indirectly through the media.

To ensure targeted reach to the specific audiences highlighted in the *Consensus Statement,* it was further shared by the networks of the Expert Panel members and their affiliated organizations. Specific findings were shared via ParticipACTION’s and other partnering individuals’/organizations’ social media channels (e.g., Twitter, Facebook, Instagram, blogs). Press releases and communications with partnering organizations’ networks also took place on the day of release. An evaluation of the *Consensus Statement* release will occur as part of the larger, more comprehensive Report Card evaluation plan, including tracking of distribution, media impressions, hits and quality, website traffic and site visits, social media reach, and engagement among users. Surveys with Report Card users and debrief meetings with ParticipACTION’s strategic partners and Expert Panel members will provide further important insights.

## Discussion

A comprehensive process was initiated in Canada to develop and release a *Consensus Statement on the Role of the Family in the Physical Activity, Sedentary, and Sleep Behaviours of Children and Youth* to consolidate related evidence, highlight key considerations, and provide recommendations to families and other related networks on how to support regular healthy movement behaviours as outlined by the Canadian 24-Hour Movement Guidelines for Children and Youth [1, 2]. Triangulated evidence was obtained from six reviews, analyses from Statistics Canada’s CHMS, and feedback and insights by Expert Panel members and those who responded to the stakeholder survey. This process culminated in the *Consensus Statement* (Fig. 2), the development of a conceptual model, a discussion of the key take-away messages, and concluded with the identification of critical gaps in current knowledge that form recommendations for future research.

An explanation and substantiation for the final content of the *Consensus Statement* is provided below. First, the summary statement deliberately situates the family as the proximal micro-system of child influence, following the work of Bronfenbrenner [209] that was outlined in the family systems review (review #4). It subsequently provides several descriptors of how a family may support child and youth movement behaviours to acknowledge the complex assortment of behaviours and practices found within the reviews on the correlates of parental support, and family characteristics and children and youth’s physical activity, sedentary, and sleep behaviours. This information was directly informed by the review of family support correlates (review #5) and indirectly based on reviews #1–3 and review #6.

The Background section of the *Consensus Statement* highlights the importance of child and youth movement behaviours, noted from past evidence that formed the Canadian 24-Hour Movement Guidelines for Children and Youth [1, 2], augmented by contemporary epidemiological evidence from our custom analyses from Statistics Canada, and supported by the United Nations Convention on the Rights of the Child [240] and the Canadian Children’s Charter [241]. Given that movement behaviours are socioecological in scope [242], the background section concludes with a more fulsome statement about the multiple sources of influence that shape child and youth movement behaviours to inform readers that family is but one important system among these core elements, as evidenced from all of our reviews, the stakeholder survey feedback, and input from the Expert Panel.

The process that was used to achieve the *Consensus Statement*, noted in the Methods section of this paper, is summarized for readers to provide the background for how we arrived at our conclusions and recommendations. This included the funding sources, reviews undertaken, the formation of the national Expert Panel, custom analyses, and stakeholder consultation process and tied release to the 2020 ParticipACTION Report Card. Finally, we note that the applicability of the *Consensus Statement* is meant to be broad and inclusive of children and youth of all gender/sex, ethnicities, and family socioeconomic status.

The *Consensus Statement* follows with a series of key evidence statements to stakeholders that broadly outline the importance of movement behaviours to child and youth health, and the role of the family, based on the evidence acquired through the process used to generate the statement. These points were not meant to be exhaustive; rather, they represent highlights of key takeaways from the reviews, analyses, and discussions of Expert Panel members that have the most evidence at present and also resonate with the *Consensus Statement* and recommendations to ensure cohesiveness of the document. These include:

- parental emotional support, physical activity modelling, parental knowledge/beliefs about physical activity, and various sociodemographic factors are related to children’s physical activity;
- parental modelling (physical activity and screen-time) and family expectations (rules) were important for limiting children and youth’s sedentary behaviour and screen-time;
- healthy expectations such as setting bedtime routines and having device-free bedrooms and good family functioning were important for helping children and youth acquire sufficient sleep; and,
- the entire family system is an important source of influence and subsequent promotion of healthy child and youth behaviours.

**Fig. 3:**
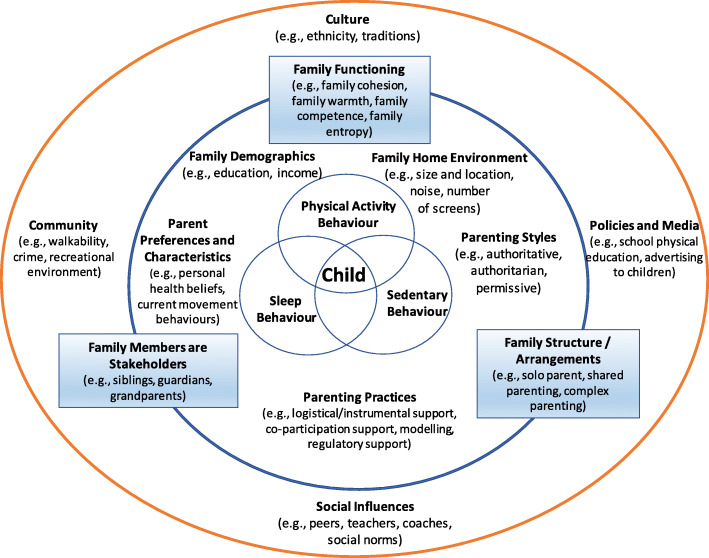
Conceptual model illustrating the relationships linking family and the physical activity, sedentary and sleep behaviours of children and youth, within a socioecological context

To relieve the word-heavy approach in the *Consensus Statement* and add visual representation of the findings from our process, the Expert Panel developed a conceptual diagram that highlights how we situate family influence on child and youth movement behaviours (Fig. 3). The figure was developed from the findings of all six reviews, the CHMS analyses, as well as input from the Expert Panel, and the stakeholder survey feedback. Through a Venn diagram, the illustration portrays physical activity, sedentary, and sleep behaviours as integrated, commensurate with the Canadian 24-Hour Movement Guidelines for Children and Youth [1], but also recognizing their unique variation that may require targeted attention. A series of concentric circles were used to situate family as a proximal source of influence, while allowing readers to quickly see that there are many sources and settings that influence child and youth movement behaviours beyond the family [209, 243, 244]. We positioned the types of influence (i.e., parenting practices, parent preferences and characteristics, parenting styles) as immediate to child and youth movement behaviours to denote the key mediators of action and then highlight core family systems constructs (i.e., family functioning, family structure, family members as stakeholders) as the likely sources of those types of influence. Finally, we acknowledge key additional influences (i.e., family demographics, community, policies and media, additional social influences) on family and subsequent child and youth movement behaviours in the outer concentric circle.

In the *Consensus Statement*, we dedicated considerable space to recommendations, subdivided by key stakeholders, because the ultimate purpose of the *Consensus Statement* is to act as an actionable document. Consideration was given to each potential group of stakeholders and recommendations were themed accordingly. These included family – broadly defined to incorporate the many structures and roles that encompass a family [245], educators, researchers, practitioners, service providers, and governments at all levels. Recommendations were evidence-based from the results of our reviews as well as discussion points among the Expert Panel and recommendations generated through the stakeholder survey. For example, for family, these included recommendations to model, facilitate, and encourage their children and youth to meet the Canadian 24-Hour Movement Guidelines [1] by creating routines, plans, priorities, and expectations. Co-activities were encouraged for physical activity and screen-time to monitor and socialize as a family. For all movement behaviours, we included a series of specific tactics that may be useful for families to assist in limiting screen viewing hours of children and youth based on the literature reviewed in our process of generating the *Consensus Statement*. A similar process for generating recommendations was followed for all other stakeholders.

The *Consensus Statement* was created to inform and activate stakeholders to promote healthy child and youth movement behaviours. In doing so, we were mindful of its length and the important balance between building an evidence-based document and an authoritative and thorough resource. Thus, to conclude the *Consensus Statement* we included a series of additional resource links and references for end-users to continue learning about the evidence used to build the document and other excellent sources of information related to the themes raised within the statement.

While the process we undertook to develop the *Consensus Statement* identified considerable evidence to substantiate the statement, it also demonstrated there are several aspects of family and child and youth movement behaviours where there are still gaps in the research literature. For example, a very recent systematic review (*n* = 23 studies, 1982–2019) which examined whether interventions with a parent/other adult caregiver component resulted in children being more physically active, reported little to no impact on children’s activity levels [246]. The most commonly reported intervention techniques were those aimed at shaping participants’ knowledge, such as providing instruction about how to perform a behaviour. However, the majority of included papers were of low or very low quality, and therefore, caution should be taken when interpreting the findings (uncertainty of the results). Accordingly, research priorities are noted below:

- Despite existing adaptations of Family Systems Theory for child and youth health behaviour in the form of schematics, only two trials [215, 216] focused on child physical activity and screen-time reduction, have been conducted. Clearly, this represents a paucity of research utilizing family systems to change behaviours of the 24-Hour Movement Guidelines for Children and Youth [1, 2].
- While parental practices in relation to child and youth movement behaviours has been linked to subsequent child and youth behaviour, current research highlights the paucity in our understanding of parental practices related to sleep and restriction of sedentary behaviour. More research on the antecedents of parental practices is needed.
- Parental practices and support of all movement behaviours would be better understood with a greater focus on social (e.g., norms, network, support) and environmental (e.g., home, community, policy) correlates of support in conjunction with individual factors. Such evidence is required.
- Almost all of the current evidence-base on family support of child movement behaviours is focused on parents, and much of this is exclusive to mothers. Family functioning (e.g., cohesion, entropy, chaos), structural arrangement supports (e.g., divorced shared parenting, single parenting) and overall family stakeholders (e.g., fathers, extended family, siblings) would assist in understanding the relative contributions of family influence.
- Research of family-based interventions for physical activity has improved over the last decade, yet family interventions on child and youth sleep and sedentary behaviour are limited. Primary research focussed on interventions to improve sedentary behaviours and sleep are still required in order to generate evidence-based recommendations.
- Interventions targeting a single movement behaviour are helpful, but we also recommend an examination of overflow effects on other non-targeted movement behaviours (e.g., does a family physical activity intervention result in better sleep practices?).
- Family intervention comparisons between integrated 24-h healthy movement behaviour approaches compared to isolated individual movement behaviour approaches will be a helpful future research direction to determine the best practice of (e.g., sequencing) behaviour change.
- Determining the best knowledge translation and mobilization techniques to increase stakeholder’s awareness, knowledge, understanding, and implementation of recommended amounts of physical activity, sedentary behaviour, screen-time and sleep is needed.

This initiative was undertaken by a large interdisciplinary team of researchers, clinicians, policy experts, public health professionals, and front-line family service providers. Though we sought international representation for the Expert Panel, our final composition lacked an international perspective. The undertaking of multiple literature reviews and custom national data analyses combined with ongoing expert consultation, was a significant strength of this project. The findings of this work have the potential to inform future contributions to the child health literature, care, and practice. With the pragmatic use of literature searches and scoping reviews rather than systematic reviews being carried out, data quality, full synthesis of findings, and risk of bias were not always considered. This choice was acknowledged as a limitation by the group, but all evidence for the development of the *Consensus Statement* was searched systematically and rigorously. The triangulation of evidence from multiple sources reinforced our confidence in the final *Consensus Statement*. Nevertheless, there are limitations to any large consensus building approach to develop similar public-facing documents. From this experience and others [1, 2, 65–69, 76] we recommend that special attention be given to inherent time and resource limitations that invariably constrain timelines and evidence quantity and quality. These realities can create conflict between research standards and knowledge translation needs and demands. In our case, these constraints precluded the lengthy preparation and publication process of systematic reviews (inclusive of review registration and quality assessment in some cases), as each one would have required 6 months or more to have this component achieved before the reviews were discussed among the Expert Panel. It also meant a much smaller window for the stakeholder survey than desirable. Our strengths, on the other hand, included a full series of consensus rounds for the final statement and the process manuscript for all panel members and a large amount of evidence (six reviews, national data, stakeholder survey, discussion time at two occasions among the panel) to make an evidence-based statement.

## Conclusions

In summary, there is broad triangulation support from the published literature, current nationally representative data, the Expert Panel, and the stakeholders consulted to support the *Consensus Statement on the Role of the Family in the Physical Activity, Sedentary, and Sleep Behaviours of Children and Youth.* This *Consensus Statement* is intended to facilitate a positive focus on the importance of key practices, policies, and societal actions to promote healthy child and youth growth and development through the integrated 24-h movement behaviours. The release of the *Consensus Statement* will benefit greatly from the proactive distribution, public relations and media strategy prepared to maximize the reach and impact of the release of the ParticipACTION Report Card. Through this, it is anticipated that the *Consensus Statement* will reach relevant government and non-government organizations whose work impacts child and youth movement behaviours across Canada and globally. Parents, educators, practitioners, service providers, lawmakers, and government will also be reached through public-facing communications channels (e.g., social media, blog, website) of ParticipACTION and collaborating organizations.

## Supplementary information

**Additional file 1 **Survey (PDF file of on-line survey). Full survey questions used to solicit assessments and comments from stakeholders on the draft *Consensus Statement*.

**Additional file 2.** Table S1 (docx). Inclusion and exclusion criteria for systematic literature searches on family and the physical activity (review #1), sedentary (review #2), and sleep (review #3) behaviours of children and youth.

**Additional file 3.** Review #1 (docx.). Search Process for Family and Physical Activity Literature Review (review #1). Themes from the physical activity literature review (review #1). References for the papers included in the family and physical activity review (review #1), organized by theme.

**Additional file 4.** Review #2 (docx.). Search Process for Family and Sedentary Behaviour Literature Review (review #2). Themes from the sedentary behaviour literature review (review #2). References for the papers included in the family and sedentary behaviour review (review #2), organized by theme.

**Additional file 5.** Review #3 (docx.). Search Process for Family and Sleep Behaviour Literature Review (review #3). Themes from the sleep literature review (review #3). References for the papers included in the family and sleep review (review #3), organized by theme.

**Additional file 6.** Table S2 (doc.). Inclusion and exclusion criteria for systematic literature searches on family systems theory (review #4), parental correlates (review #5), and family interventions (review #6) for movement behaviours of children and youth.

**Additional file 7.** Review #4 (docx.). Search Process for the Family Systems in the Context of Child Health Behaviour Change and Family-Based Interventions Scoping Review (review #4). References extracted from the Family Systems in the Context of Child Health Behaviour Change Scoping Review (review #4).

**Additional file 8.** Review #5 (docx.). Search Process for the Correlates of Parental Support of Child and Youth Physical Activity, Sedentary Behaviour, and Sleep Systematic Review (review #5). References Extracted from the Correlates of Parental Support of Child and Youth Physical Activity, Sedentary Behaviour, and Sleep Systematic Review (review #5).

**Additional file 9:.** Figure S1 (doc.). Search process of the Review of Reviews on Interventions Involving the Family to Change Child and Youth Physical Activity, Sedentary Behaviour, and Sleep (review #6).

## Data Availability

Material gathered and collated for the six reviews are included in this manuscript and its supplementary information files. The custom analyses from Statistics Canada in their entirety are available upon reasonable request, pending approval from Statistics Canada. Summaries of the stakeholder survey are provided in the manuscript and detailed results cannot be provided to assure respondent anonymity.
