# Supplementary material for: Development of a consensus statement on the role of the family in the physical activity, sedentary, and sleep behaviours of children and youth
Source: Int J Behav Nutr Phys Act. 2020 Jun 16;17:74. doi: 10.1186/s12966-020-00973-0 (PMC7296673; doi:10.1186/s12966-020-00973-0)
Supplement: Supplementary file 1 — Additional file 1 Survey (PDF file of on-line survey). Full survey questions used to solicit assessments and comments from stakeholders on the draft Consensus Statement. [file 12966_2020_973_MOESM1_ESM.pdf]

Consensus Statement

On the Role of the Family in the Physical Activity, Sedentary and Sleep Behaviours of Children and Youth

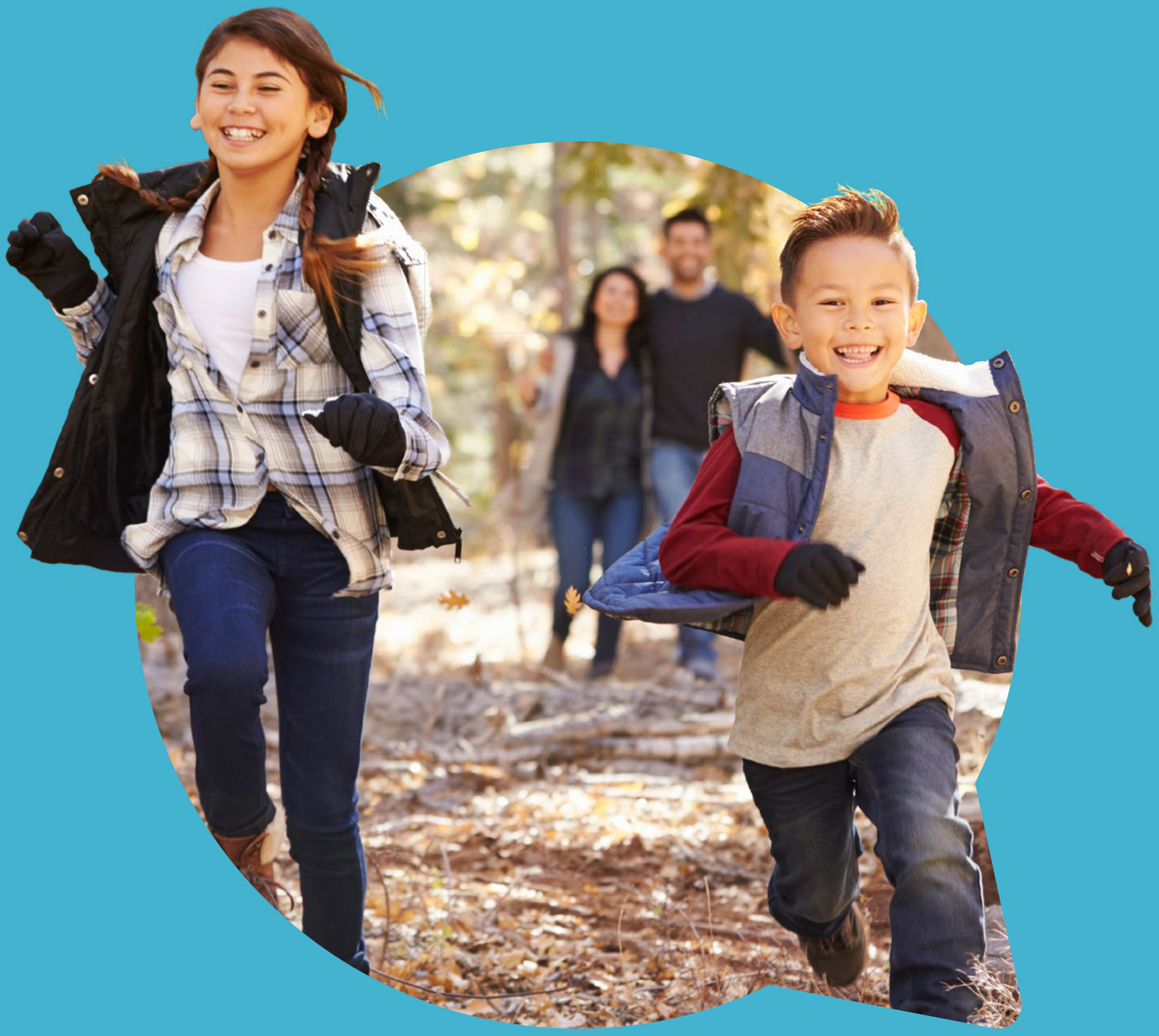

PARTICIPACTION

Families can support children and youth in achieving healthy physical activity, sedentary and sleep behaviours by encouraging, facilitating, modelling, setting expectations and engaging in healthy movement behaviours with them. Other sources of influence are important (e.g., child care, school, health care, community, governments) and can support families in these pursuits.

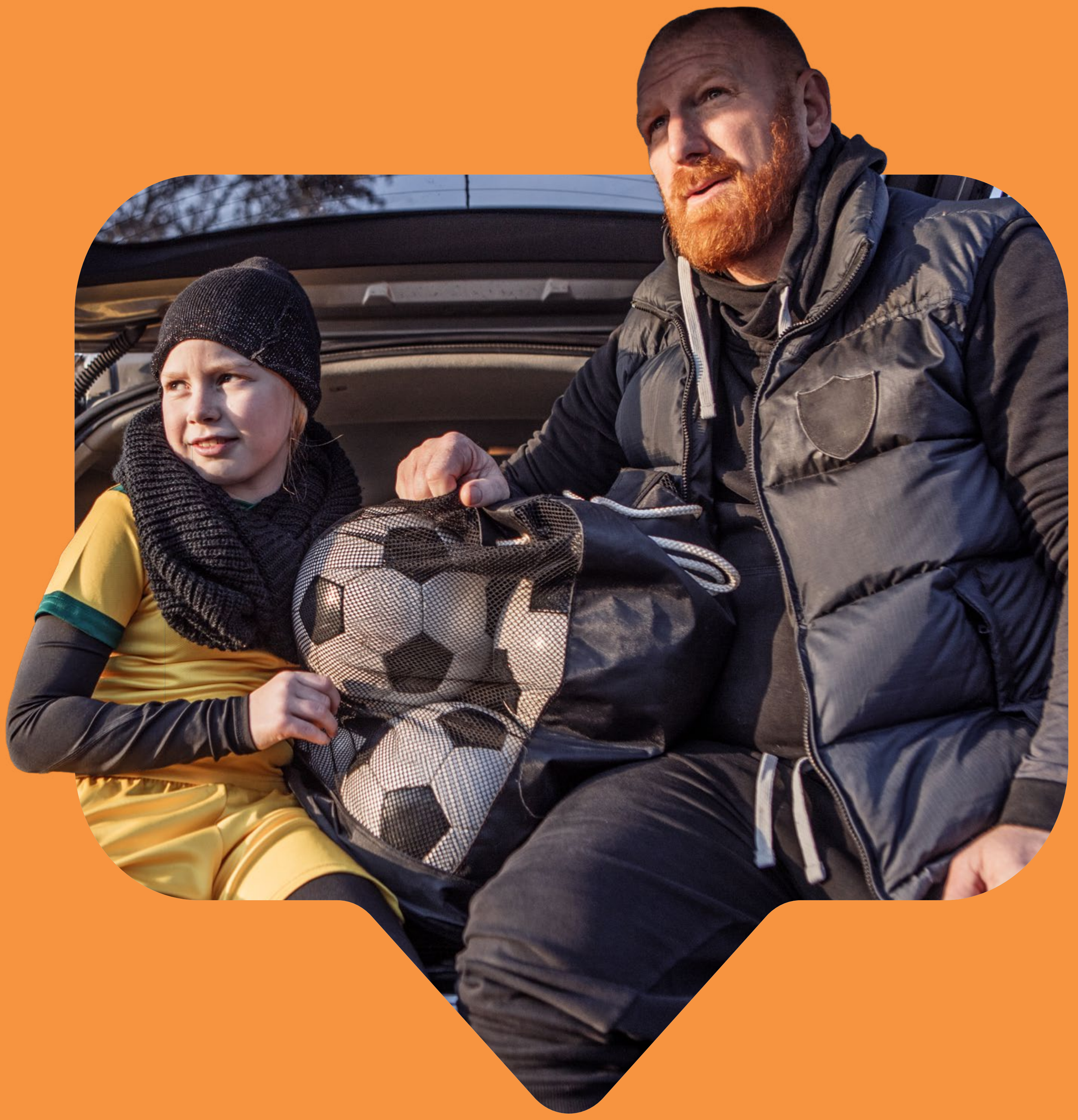

Background

Healthy physical activity, sedentary and sleep behaviours in childhood and adolescence are essential for healthy growth and development,[1–9] and this fact led to the development of the Canadian 24-Hour Movement Guidelines.[10,11] Unfortunately, few Canadian children and youth are meeting these guidelines.[12–14] There has been a dramatic decline in Canadian children's fitness over the past 35 years,[15–18] which has negatively affected their health.[19–23] The United Nations Convention on the Rights of the Child protects a child's right to rest and play in recreational and leisure activities appropriate to their age.[24] These rights are reinforced in the Canadian Children's Charter.[25]

Unfortunately, Canadians have not been successful in honouring these commitments. The 2020 ParticipACTION Report Card on Physical Activity for Children and Youth gives Canada a grade of "D+" for Overall Physical Activity, "D+" for Sedentary Behaviour, "B" for Sleep, and "F" for 24-Hour Movement Behaviours.[26] A recent UNICEF Canada report supports these findings, highlighting that only 21% of children aged 5-11 years engage in at least 1.5 hours per day of active play and unstructured physical activities.[27] Additional findings from Statistics Canada indicate that youth aged 12-17 years accumulate 4 hours per day of screen time outside of school.[28]

Movement behaviours are affected by the family, home, school, community, government and environment.[29–33] The family (e.g., parents, guardians, siblings) is typically the closest and most important influence, and its impact has received considerable research attention.[34–36] This Consensus Statement was developed by synthesizing and interpreting the research evidence, integrating expert input, and incorporating stakeholder and end-user feedback to provide guidance for families, and those who influence them.

Families vary in structure, function, roles and interactions among members. Families are constantly changing, creating both challenges and opportunities to promote healthy movement behaviours.

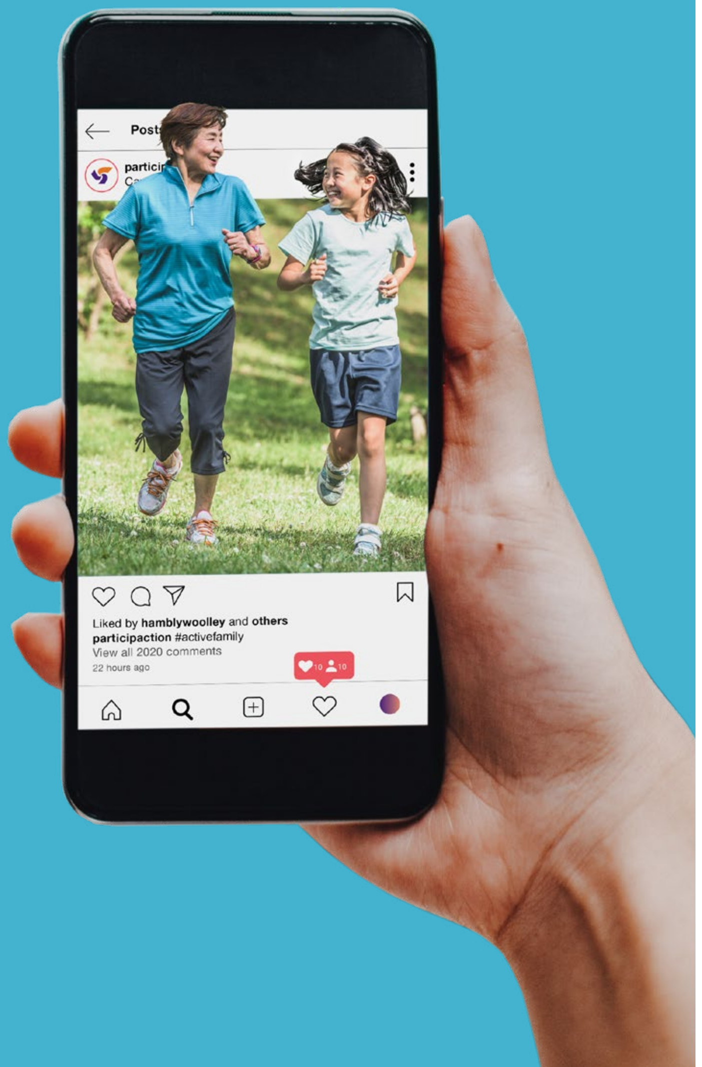

Process

The process to develop this Consensus Statement included completing six comprehensive literature reviews, establishing a national multidisciplinary Expert Panel, gathering custom data analyses from Statistics Canada's Canadian Health Measures Survey (CHMS), integrating related research identified by Expert Panel members, conducting a stakeholder consultation process, achieving Expert Panel consensus and releasing the Consensus Statement in conjunction with the 2020 ParticipACTION Report Card.[37]

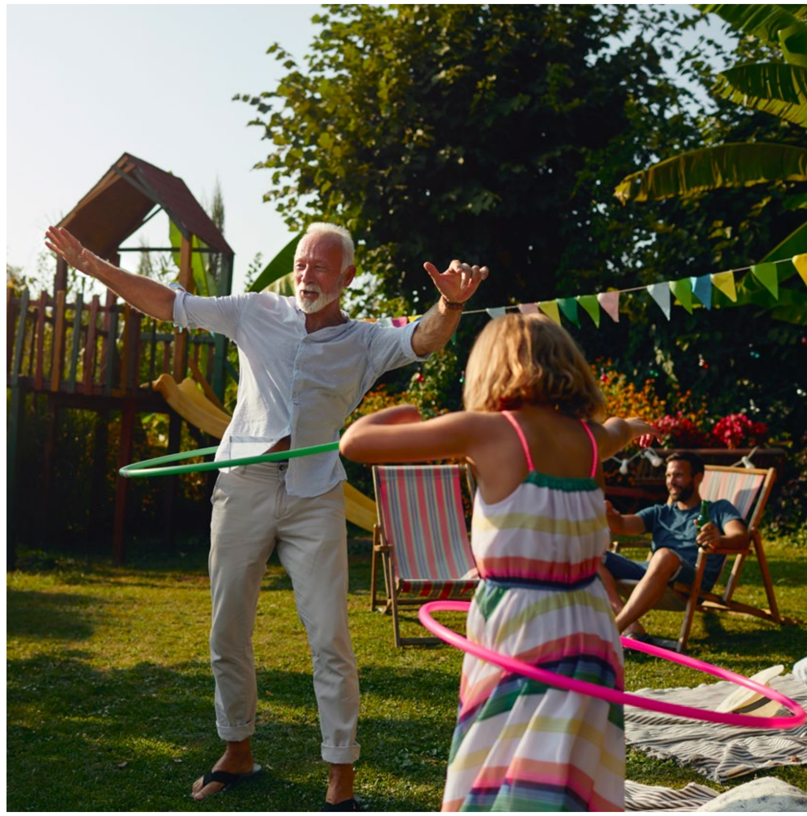

Summary of Supporting Evidence

This Consensus Statement applies to all families and is relevant for children and youth aged 0-17 years, including all genders/sexes, ethnicities and family socio-economic statuses. Families vary in structure, function, roles and interactions among members, and are constantly changing,[38,39] creating both challenges and opportunities for the promotion of healthy movement behaviours.

Overall Movement Behaviours

- Less than 1 in 5 children and youth in Canada meet national guidelines for physical activity, sedentary and sleep behaviours.[5,12,14,40]
- Positive parenting practices and attitudes, perceived control, perceived low risk of harm in activities, and intentions to provide support are critical; parents who make specific plans (how, when, where) are more likely to follow through and support their child's healthy movement behaviours.[36,41–48]
- As children age, their families provide less support for movement behaviours; however, parental support remains extremely important in supporting these behaviours in youth.[49,50]

Physical Activity

- Parents' physical activity is associated with that of their children, with each additional 20 minutes of parental moderate-to-vigorous physical activity associated with an extra 5 minutes for their child;[51] this finding is supported by other research.[35,52] Parental fitness characteristics are also related to those of their children.[53]
- Family social support is positively associated with children's physical activity levels.[54]
- There are generally no differences in the physical activity behaviours of children and youth according to the number of siblings in the household, or single- versus two-parent households.[53]
- Family-based interventions are generally effective at improving physical activity, while evidence about the efficacy of screen-time interventions to increase physical activity is less conclusive.[55–59] Providing families with educational materials about reducing screen time, without providing additional intervention components, may not be effective in changing child and youth physical activity behaviours. Current evidence supports using interventions that focus on self-regulatory approaches (e.g., planning, setting goals) and involve the whole family.[57]

Sedentary Behaviours

- The number of parents who reported that they spend too much time on their own mobile devices increased from 29% in 2016 to 52% in 2019.[60]
- The proportion of children and youth who thought their parent(s) were addicted to their mobile devices and wished their parent(s) would get off their device increased from 28% in 2016 to 39% in 2019.[60]
- 78% of parents believed they were good media and technology role models for their children,[61] even though the parents reported having high levels of screen viewing.
- Two-thirds of parents said that monitoring their children's media use was more important than respecting their privacy;[61] however, only 14% of youth with a phone or tablet said their parents tracked their device time.[62]
- Parents overwhelmingly have positive attitudes about the role of technology in their children's education and development of important skills.[61]
- Children spend less time engaged in screen-based behaviours if they live in households with screen time restrictions.[63–65]
- Higher parenting stress and lower household income are associated with a higher amount of daily screen time in infants and toddlers aged 7-18 months.[66]
- By age 11, the majority (53%) of children have their own smartphone, and this increases to 69% by age 12.[62]
- Children aged 9-12 years from higher-income homes accumulate an hour and 50 minutes less screen time per day than those from lower-income households.[62]
- Having screens in bedrooms is consistently linked with more screen viewing.[67–69]

Sleep

- Good sleep hygiene is associated with positive sleep outcomes, such as longer sleep duration and better quality of sleep. Good sleep hygiene includes regular bedtimes, consistent bedtime routines (e.g., bathing, brushing teeth, reading) and screen-free bedrooms.[70–73]
- One-third of youth keep their mobile devices in bed with them.[60]
- Having screens in bedrooms is consistently linked with less sleep.[67–69]

Conceptual Model

The Expert Panel developed the conceptual model below. It builds on earlier models that examined family systems in the context of child health behaviour change, and also incorporates new evidence.[74–76] The model illustrates the complexity of the family's role in influencing integrated movement behaviours, and provides a guide for future research and interventions.

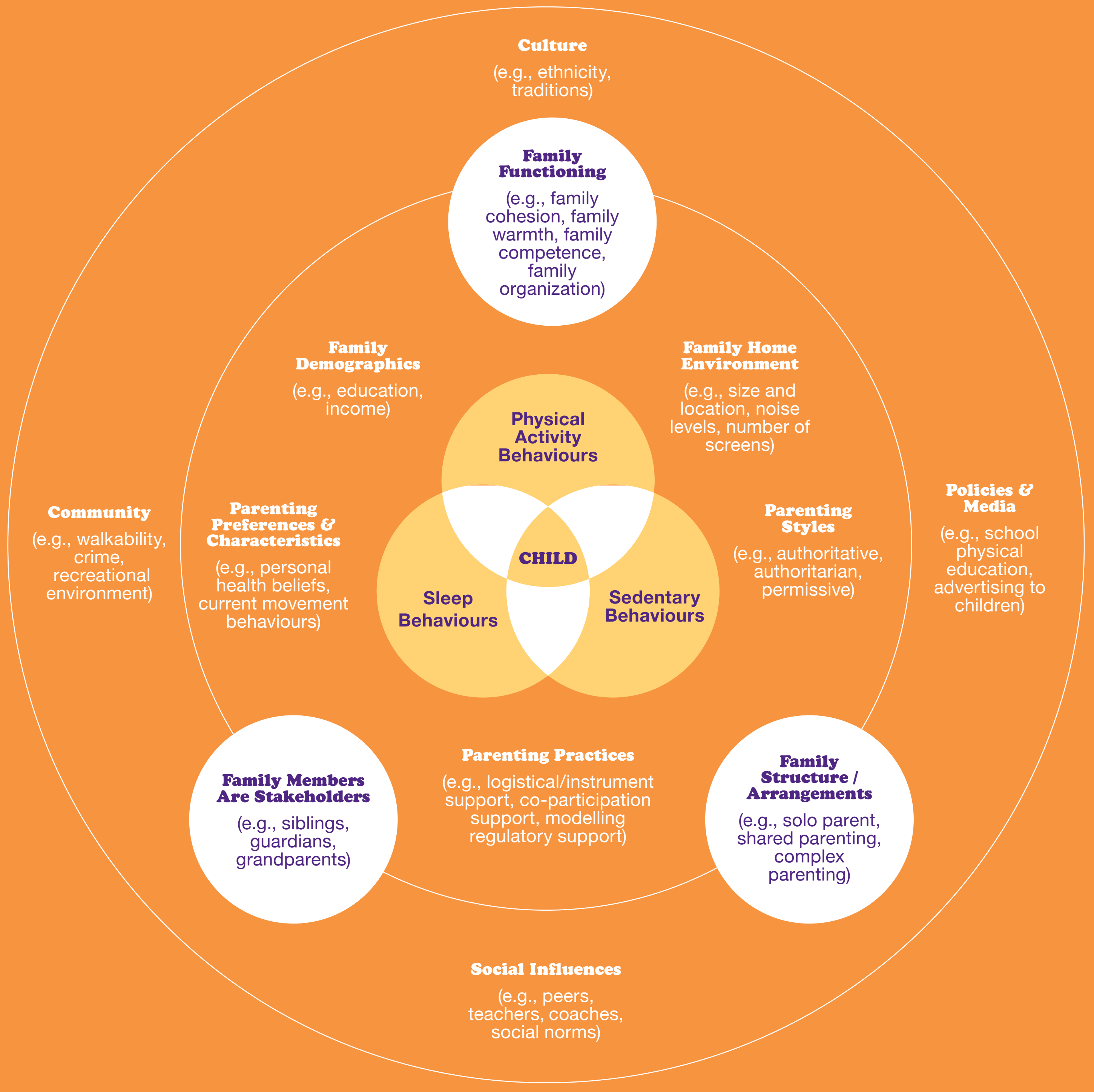

Recommendations

Families may find it challenging to support children and youth in achieving the 24-Hour Movement Guidelines. A list of recommendations for how families, and those who influence them, can improve the physical activity, sedentary and sleep behaviours of children and youth is provided below. These recommendations are based on the evidence reviewed for the Consensus Statement, and the expertise of the Expert Panel members.

Family

Including parents, elders, grandparents, siblings, legal guardians

Overall Movement Behaviours

- Know, understand and strive to adhere to the 24-Hour Movement Guidelines.[10,11]
- Establish an environment that supports healthy movement, and create routines and expectations for meeting the 24-Hour Movement Guidelines.[10,11]
- Recognize that engaging in recommended healthy movement behaviours reinforces family cohesiveness.
- Be a good role model by being physically active, limiting your own sedentary behaviour and screen time, and practising healthy sleep habits.
- Help children self-regulate their behaviours by setting expectations and making sure to involve them in deciding how they can meet the 24-Hour Movement Guidelines.[10,11]
- Encourage more outdoor time to increase physical activity, decrease sedentary behaviour and improve sleep.[77]

Physical Activity

- Facilitate physical activity by encouraging, watching, role modelling and attending physical activity events, and by co-participation.[52,54,78–91]
- Be active as a family – this encourages physical activity, social support, connectedness and attachment, which are all important for good mental health.[92]
- Promote and support opportunities for physical activity, active outdoor play and sport at home and school, and in the community.[26]
- Incorporate more opportunities for active transportation – walk, bike, wheel, skateboard, scooter or take transit to school, stores, parks and activities.[93]
- Allow your children to play and roam unsupervised, while ensuring that their range is safe.[94]
- Join with other families in enabling physical activity opportunities.

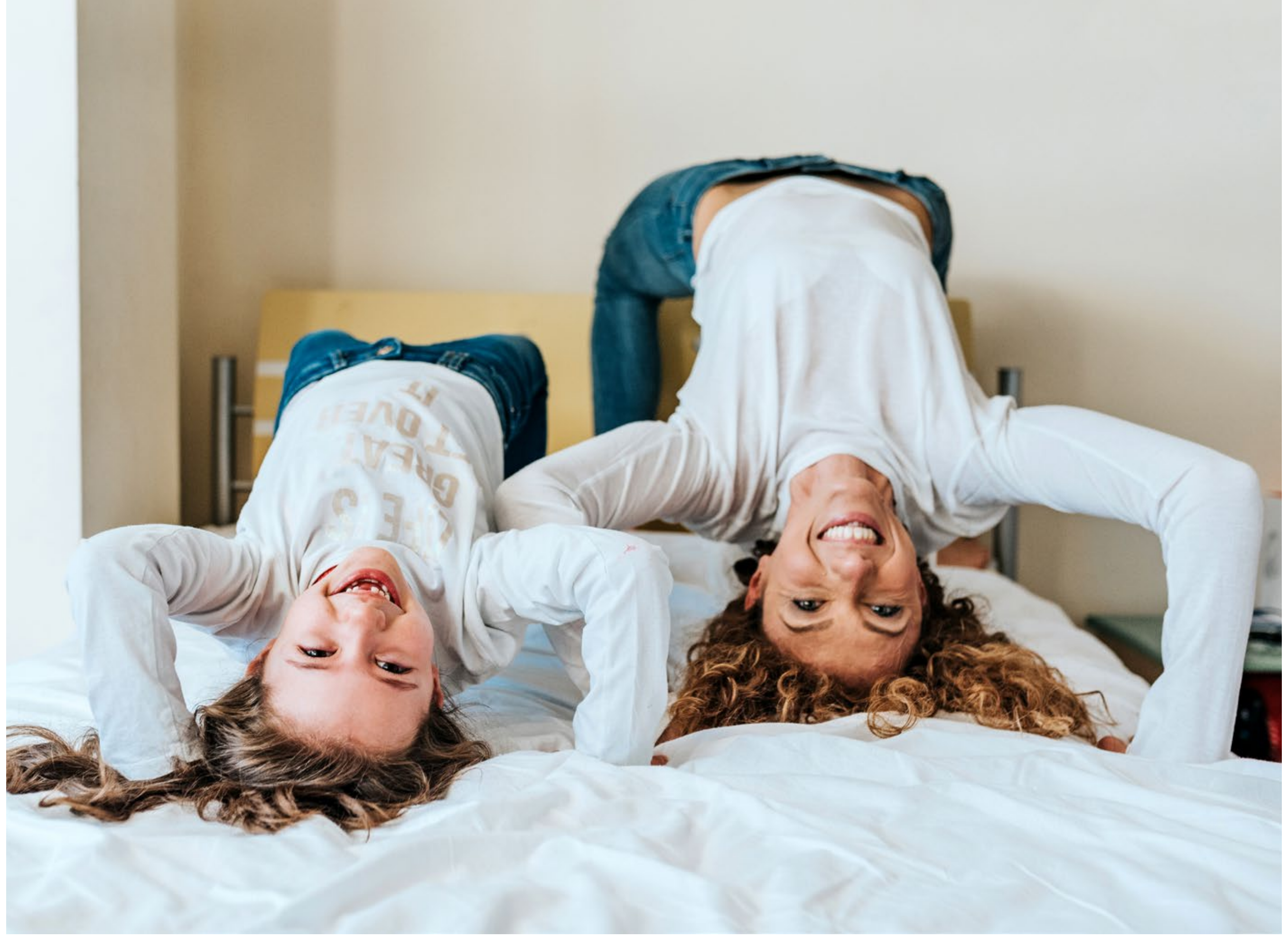

Sedentary Behaviours

- Create a family media plan that includes:
  - Setting limits around screen viewing consistent with established guidelines[10,11,63,64,69,95–108]
  - Prioritizing screen-free family time[97,109,110]
  - Prioritizing device-free time at home and using “device baskets” (bins for personal mobile devices) as an aid
  - Removing screens from children’s bedrooms[68,69,97,109–118]
  - Having screen-free family meals[97,104,109,110,116,119–121]
- Avoid using screen time as a reward.
- Be present and engaged when screens are used, and co-view when possible.[10,11,95–97,99,109,110]
- Break up children’s prolonged sitting with movement whenever possible – at home and during travel.[10,11,95–97,99]

Sleep

- Create and adhere to a consistent bedtime routine (e.g., bathing, brushing teeth, reading).[10,11,95–97,99,122–124]
- Schedule and encourage a regular bedtime that allows for sufficient sleep.[10,11,95,100,125,126]
- Support screen-free bedrooms for all family members.[97,104,110,122,127–134]
- Discourage screen viewing in the hour before bedtime.[96,109,110]
- Provide children with a comfortable and quiet sleeping environment.[122,124–126]

Educators & School Administrators

- Support families’ pursuit of healthy movement behaviours for their children and youth by:
  - Knowing and understanding the 24-Hour Movement Guidelines[10,11]
  - Embracing opportunities to incorporate healthy and inclusive movement messages, practices and policies into daily school routines and lessons by:
    - Respecting daily physical activity (DPA) policies
    - Ensuring all children have access to daily recess breaks that encourage physical activity
    - Reducing excessive safety restrictions on physical activity on school grounds (e.g., tag, tree climbing)
    - Minimizing long periods of sedentary behaviour and interrupting any long periods with active breaks
    - Scheduling classes to encourage changes in posture
    - Providing options for standing desks in classrooms and libraries[135]
    - Creating a culture that moderates the use of screens in schools
    - Integrating sleep health messages into the school curriculum
    - Supporting children and youth in self-regulating their habitual movement behaviours
    - Modelling healthy movement behaviours and discussing the importance of healthy, habitual movement behaviours
    - Assigning healthy movement behaviour homework – more physical activity, less sedentary behaviour and screen time, and a good night’s sleep
- Ensuring that before- and after-school programming incorporates healthy and inclusive movement messages, practices and policies.

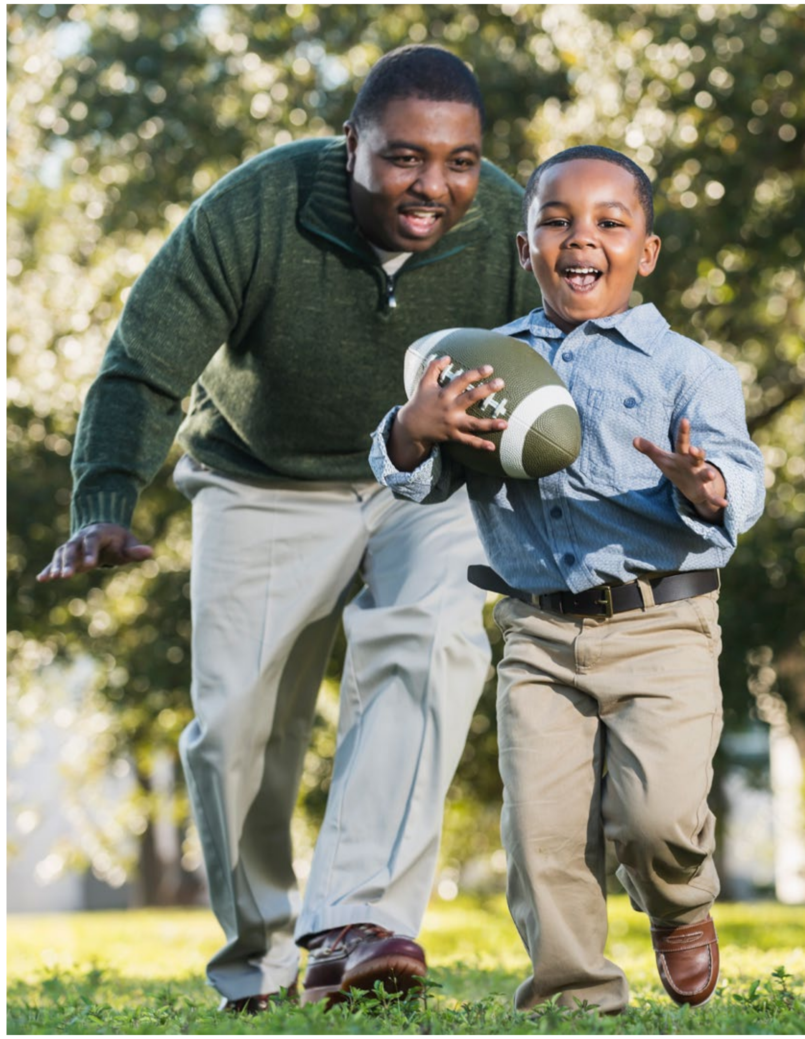

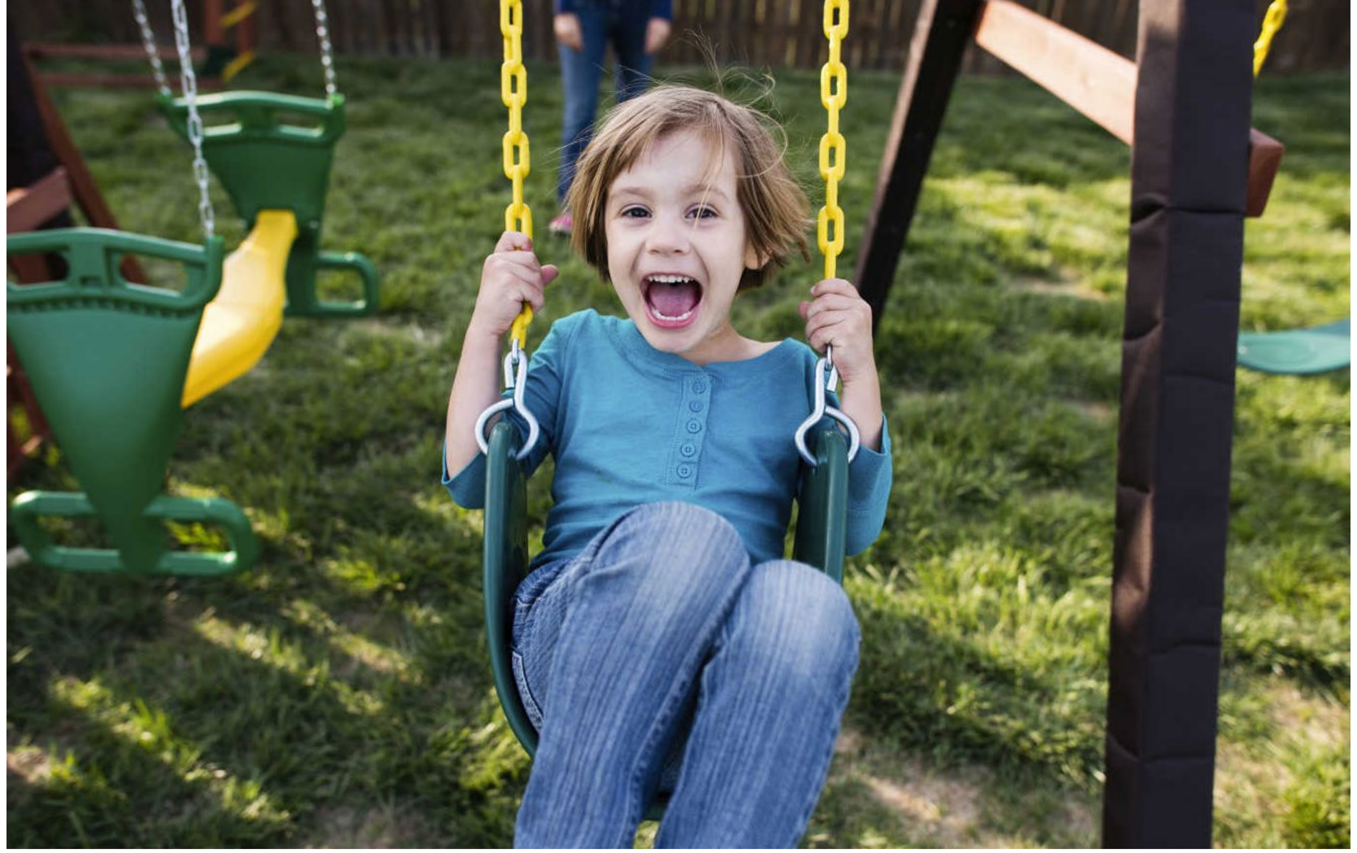

Academics & Researchers

- Help inform families' pursuit of healthy movement behaviours for their children and youth by:
  - Addressing evidence gaps in understanding family influence on healthy movement behaviours
  - Examining family-based interventions that use various behaviour modification techniques to address healthy physical activity, sedentary and sleep behaviours concurrently
  - Studying the external factors that mediate families' ability to adhere to the 24-Hour Movement Guidelines
  - Advocating for evidence-informed policies and practices related to the role of the family in healthy physical activity, sedentary and sleep behaviours
  - Engaging in knowledge translation efforts targeting families, the media, educators and policymakers

Health Care Practitioners

- Support families' pursuit of healthy movement behaviours for their children and youth by:
  - Promoting the 24-Hour Movement Guidelines[10,11] and recommendations from the Canadian Paediatric Society's Position Statement on Digital Media Use[109,110] during routine visits
  - Asking about the family's movement behaviours and emphasizing a whole family approach to improving these behaviours
  - Recommending that family members encourage, facilitate, model, set expectations, and participate in efforts that allow their children to achieve healthy movement behaviours
  - Becoming familiar with resources that enable families to support healthy movement behaviours
  - Advocating for preventive health-care tools such as the Rourke Baby Record and Greig Record to include an assessment of the family's role in physical activity, sedentary and sleep behaviours[109,110,136,137]

Allied professionals and community programming staff

- Support families' pursuit of healthy movement behaviours for their children and youth by:
  - Integrating key messages from the 24-Hour Movement Guidelines[10,11] into community-based programming
  - Modifying programs for parents by integrating healthy movement recommendations
  - Incorporating planning activities (e.g., developing sleep routines, screen time planning, physical activity scheduling) in family interventions/service provision
  - Encouraging and facilitating standing, stretching and quiet movement while clients are waiting or during events

Employers

- Support families' pursuit of healthy movement behaviours for their children and youth by:
  - Ensuring healthy 24-hour movement behaviours are part of a child and family health component in workplace wellness initiatives
  - Allowing flexibility in work hours to enable parents to promote and practise healthy family movement behaviours

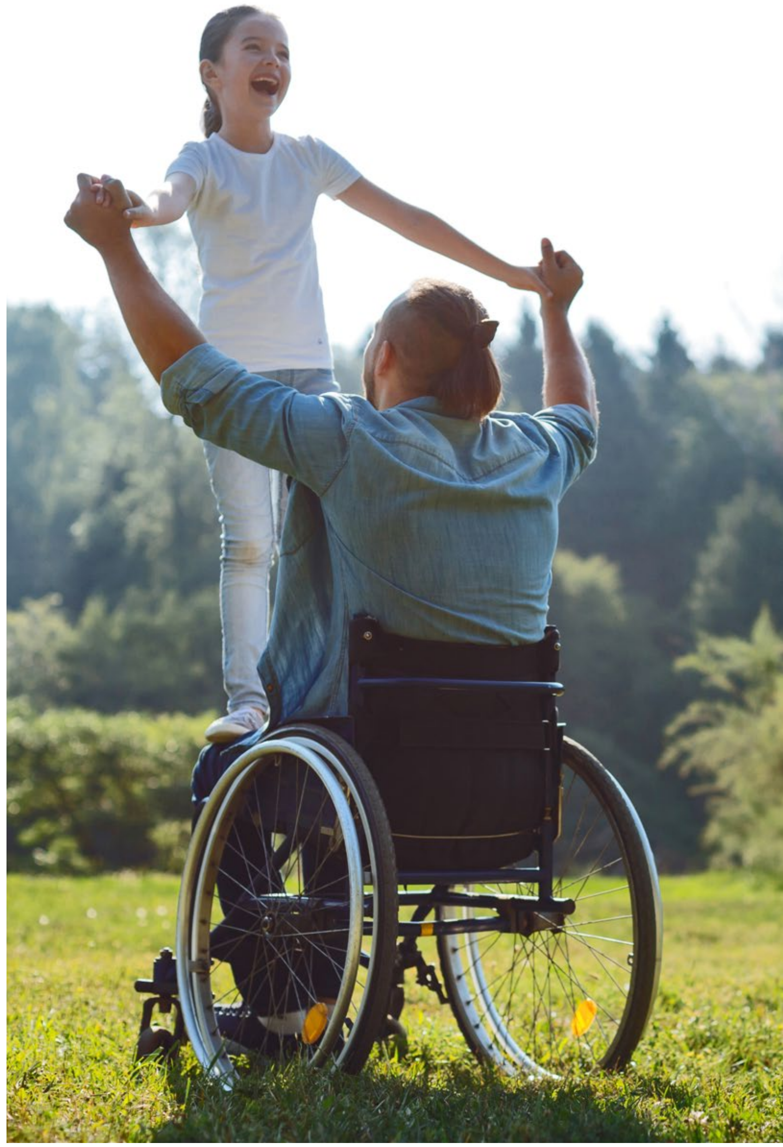

Federal, provincial, territorial, municipal as appropriate

Governments

- Support families' pursuit of healthy movement behaviours for their children and youth by:
  - Developing, implementing and evaluating strategies for healthy movement behaviours that draw from the guiding strategy "A Common Vision for Increasing Physical Activity and Reducing Sedentary Living in Canada: Let's Get Moving"[138]
  - Providing sustained resources and developing policies for the promotion and implementation of the 24-Hour Movement Guidelines in the context of the family[139]
  - Promoting healthy 24-hour movement behaviours by increasing access to schools, recreation centres and other public spaces outside of school hours
  - Supporting families' participation in sport and recreation activities[140]
  - Requiring manufacturers of digital screen devices to disclose on product labels the potential harms associated with excessive use
  - Investing in no-charge active play spaces (especially outdoors) for families with children of all ages
  - Providing infrastructure and social marketing to support families in adopting active transport practices
  - Updating educational curricula to better incorporate healthy physical activity, sedentary and sleep behaviours with a family-focused lens and through a whole-school approach
  - Supporting neighbourhood associations, recreation centres and schools in developing programs to encourage family physical activity
  - Reviewing and amending bylaws as needed to allow, promote and encourage outdoor play opportunities (e.g., climbing trees, playing street hockey and pick-up ball games, building tree forts, tobogganing)
  - Providing transit options that reach beaches, hiking trails, parks and other areas that promote healthy movement behaviours
  - Developing child protection policies that promote independent movement opportunities suited to the capacity of the child and their environment

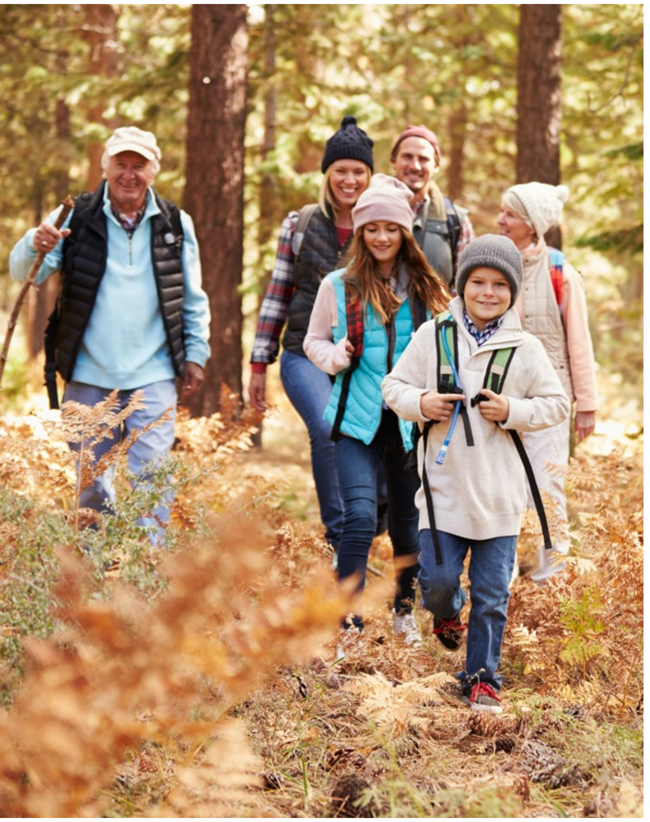

Expert Panel Members & Affiliations

Dr. Catherine S. Birken, SickKids Research Institute and the Department of Pediatrics, University of Toronto, Toronto, ON

Dr. Jean-Philippe Chaput, Healthy Active Living and Obesity Research Group, Children's Hospital of Eastern Ontario Research Institute, Ottawa, ON

Dr. Guy Faulkner, School of Kinesiology, University of British Columbia, Vancouver, BC

Dr. Michelle D. Guerrero, Healthy Active Living and Obesity Research Group, Children's Hospital of Eastern Ontario Research Institute, Ottawa, ON [member of Steering Committee]

Dr. Ian Janssen, School of Kinesiology and Health Studies, and Department of Public Health Sciences, Queen's University, Kingston, ON

Dr. Sheri Madigan, Department of Psychology, Faculty of Arts, University of Calgary, Calgary, AB

Dr. Louise C. Mâsse, BC Children's Hospital Research Institute / School of Population and Public Health, Faculty of Medicine, University of British Columbia, Vancouver, BC

Dr. Tara-Leigh McHugh, Faculty of Kinesiology, Sport and Recreation, University of Alberta, Edmonton, AB

Dr. Ryan E. Rhodes, Behavioural Medicine Laboratory, School of Exercise Science, Physical and Health Education, University of Victoria, Victoria, BC [member of Steering Committee]

Dr. Jacob Shelley, Faculty of Law & School of Health Studies, Faculty of Health Sciences, Western University, London, ON

Nora Spinks, The Vanier Institute of the Family, Ottawa, ON

Kelly Stone, Families Canada, Ottawa, ON

Dr. Katherine A. Tamminen, Faculty of Kinesiology and Physical Education, University of Toronto, Toronto, ON

Dr. Jennifer R. Tomasone, School of Kinesiology and Health Studies, Queen's University, Kingston, ON

Dr. Mark S. Tremblay, Healthy Active Living and Obesity Research Group, Children's Hospital of Eastern Ontario Research Institute, Ottawa, ON [Scientific Chair and member of Steering Committee]

Dr. Leigh M. Vanderloo, ParticipACTION, Toronto, ON [member of Steering Committee]

Helen Ward, Kids First Parents Association of Canada, Burnaby, BC

Dr. Frank Welsh, Canadian Public Health Association, Ottawa, ON

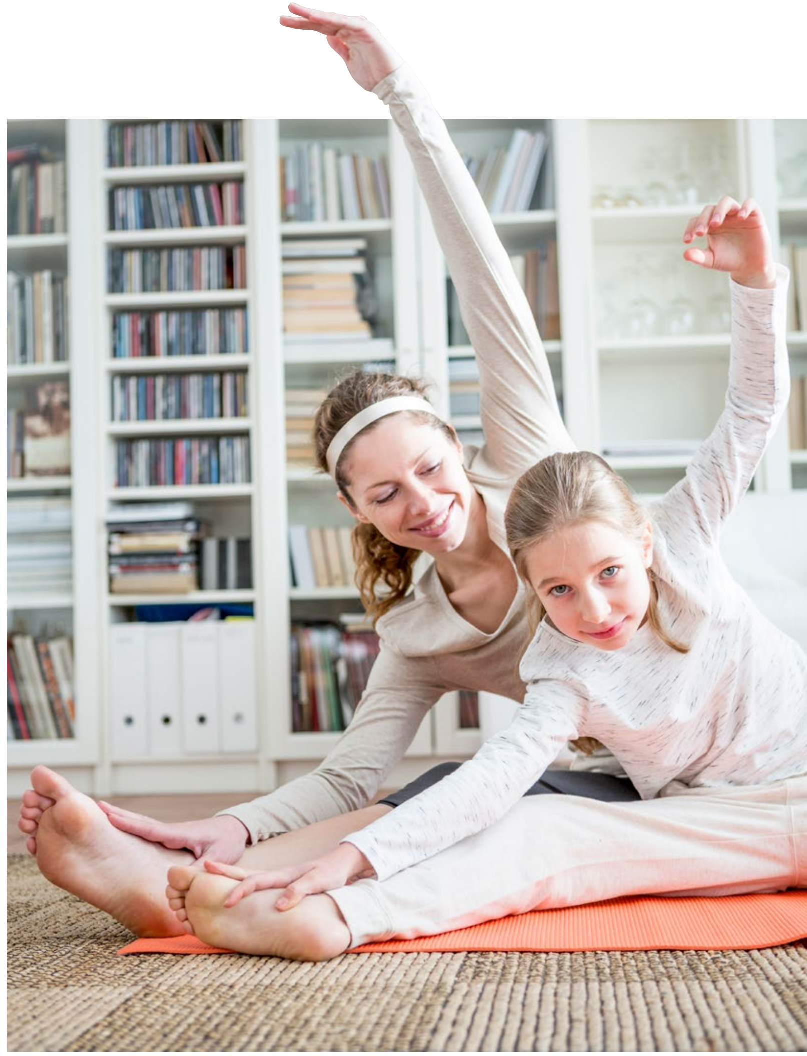

References

1. Carson V, et al. Systematic review of the relationships between physical activity and health indicators in the early years (0-4 years). BMC Public Health. 2017;17(S5):854.
2. Poitras VJ, et al. Systematic review of the relationships between sedentary behaviour and health indicators in the early years (0-4 years). BMC Public Health. 2017;17(S5):868.
3. Chaput JP, et al. Systematic review of the relationships between sleep duration and health indicators in school-aged children and youth. Appl Physiol Nutr Metab. 2016;41(S3):S266-S282.
4. Poitras VJ, et al. Systematic review of the relationships between objectively measured physical activity and health indicators in school-aged children and youth. Appl Physiol Nutr Metab. 2016;41(S3):S197-S239.
5. Carson V, et al. Cross-sectional associations between sleep duration, sedentary time, physical activity and adiposity indicators among Canadian preschool children using compositional analyses. BMC Public Health. 2017;17(S5):848.
6. Carson V, et al. Systematic review of sedentary behaviour and health indicators in school-aged children and youth: an update. Appl Physiol Nutr Metab. 2016;41(S3):S240-S265.
7. Saunders TJ, et al. Combinations of physical activity, sedentary behaviour and sleep: relationships with health indicators in school-aged children and youth. Appl Physiol Nutr Metab. 2016;41(S3):S283-S293.
8. Kuzik N, et al. Systematic review of the relationships between combinations of movement behaviors and health indicators in the early years (0 to 4 years). BMC Public Health. 2017;17(S5):849.
9. Carson V, et al. Associations between sleep duration, sedentary time, physical activity, and health indicators among Canadian children and youth using compositional analyses. Appl Physiol Nutr Metab. 2016;41(S3):S294-S302.
10. Tremblay MS, et al. Canadian 24-Hour Movement Guidelines for the Early Years (0-4 years): An Integration of Physical Activity, Sedentary Behaviour, and Sleep. BMC Public Health. 2017;17(S5):874.
11. Canadian Society for Exercise Physiology. Canadian 24-Hour Movement Guidelines for Children and Youth: An Integration of Physical Activity, Sedentary Behaviour, and Sleep. Ottawa; 2016. https://csepguidelines.ca/children-and-youth-5-17/.
12. Roberts KC, et al. Meeting the Canadian 24-Hour Movement Guidelines for Children and Youth. Health Rep. 2017;28(10):3-7.
13. Roman-Viñas B, et al. Proportion of children meeting recommendations for 24-hour movement guidelines and associations with adiposity in a 12-country study. Int J Behav Nutr Phys Act. 2016;13(1):123.
14. Janssen I, et al. Adherence to the 24-Hour Movement Guidelines among 10- to 17-year-old Canadians. Health Promot Chronic Dis Prev Canada. 2017;37(11):369-375.
15. Tremblay MS, et al. Fitness of Canadian children and youth: Results from the 2007-2009 Canadian Health Measures Survey. Health Rep. 2010;21(1):7-20.
16. Craig CL, et al. Trends in aerobic fitness among Canadian children and adults 1981 to 2007-2009: what can we conclude? Appl Physiol Nutr Metab. 2012;37:511-519.
17. Tomkinson GR, et al. Temporal trends in the cardiorespiratory fitness of children and adolescents representing 19 high-income and upper-middle-income countries between 1981 and 2014. Br J Sports Med. 2019;53:478-456.
18. Colley RC, et al. Trends in physical fitness among Canadian children and youth. Health Rep. 2019;30(10):3-13.
19. Lang JJ, et al. Making a case for cardiorespiratory fitness surveillance among children and youth. Exerc Sport Sci Rev. 2018;46(2):66-75.
20. Silva DAS, et al. Cardiorespiratory fitness in children: evidence for criterion-referenced cut-points. PLoS One. 2018;13(8):e0201048.
21. Lang JJ, et al. The association between physical fitness and health in a nationally representative sample of Canadian children and youth aged 6 to 17 years. Health Promot Chronic Dis Prev Canada. 2019;39(3):104-111.
22. Tomkinson GR, et al. The 20-m shuttle run: assessment and interpretation of data in relation to youth aerobic fitness and health. Pediatr Exerc Sci. 2019;31(2):152-163.
23. Lang JJ, et al. Review of criterion-referenced standards for cardiorespiratory fitness: what percentage of international children and youth are apparently healthy? Br J Sports Med. 2019;53:953-958.
24. United Nations. UN General Assembly, Convention on the Rights of the Child. United Nations, Treaty Series. 1989. https://www.unicef.org/child-rights-convention/convention-text-childrens-version.
25. Children First Canada. The Canadian Children's Charter: A Call to Action to Respect, Protect and Fulfil the Rights of Canada's Children. 2018. https://childrenfirstcanada.org/childrens-charter-english.
26. ParticipACTION. 2020 ParticipACTION Report Card on Physical Activity for Chilldren and Youth. 2020. https://www.participaction.com/en-ca/resources/report-card.
27. UNICEF Canada. Where Does Canada Stand? The Canadian Index of Child and Youth Well-Being Baseline Report. 2019. https://oneyouth.unicef.ca/sites/default/files/2019-08/2019_Baseline_Report_Canadian_Index_of_Child_and_Youth_Well-being.pdf
28. Janssen I, LeBlanc AG. Systematic review of the health benefits of physical activity and fitness in school-aged children and youth. Int J Behav Nutr Phys Act. 2010;7(1):40.
29. Sallis JF, et al. An ecological approach to creating more physically active communities. Ann Rev Pub Heal. 2006;27:297–322.
30. Owen N, et al. Adults' sedentary behavior - determinants and interventions. Am J Prev Med. 2011;41(2):189-196.
31. Grandner MA. Social-ecological model of sleep health. In: Grandner MA (Ed), Sleep and Health. London: Elsevier; 2019:45-53.
32. Stokols D. Establishing and maintaining healthy environments: toward a social ecology of health promotion. Am Psychol. 1992;47(1):6-22.
33. Bronfenbrenner U, Morris PA. The bioecological model of human development. In: Damon W (Series Ed.) and Lerner RM (Vol. Ed.), Handbook of Child Psychology. New York: Wiley; 2006:793-828.
34. Rhodes RE, Quinlan A. The family as a context for physical activity promotion. In: Beauchamp MR Eyes MA (Eds.), Group Dynamics in Exercise and Sport. 2nd ed. New York: Routledge; 2014:203–221.

35. Xu H, et al. Associations of parental influences with physical activity and screen time among young children: a systematic review. J Obes. 2015;2015:546925.
36. Pyper E, et al. Do parents' support behaviours predict whether or not their children get sufficient sleep? A cross-sectional study. BMC Public Health. 2017;17:432.
37. Rhodes R, et al. Development of a consensus statement on the role of the family in the physical activity, sedentary, and sleep behaviours of children and youth. Int J Behav Nutr Phys Act. 2020.
38. Cox, MJ, Paley B. Families as systems. Annu Rev Psychol. 1997;48:43–267.
39. Cox, MJ, Paley B. Understanding families as systems. Curr Dir Psychol Sci. 2003:193–196.
40. Chaput JP, et al. Proportion of preschool-aged children meeting the Canadian 24-Hour Movement Guidelines and associations with adiposity: results from the Canadian Health Measures Survey. BMC Public Health. 2017;17(S5):829.
41. Carver A, et al. Are children and adolescents less active if parents restrict their physical activity and active transport due to perceived risk? Soc Sci Med J. 2010;70(11):1799-1805.
42. Davison KK, et al. Associations among social capital, parenting for acive lifestyles, and youth physical activity in rural families living in upstate New York. Soc Sci Med J. 2012;75(8):1488-1496.
43. Huffman LE, et al. Associations between parenting factors, motivation, and physical activity in overweight African American adolescents. Ann Behav Med. 2018;52(2):93-105.
44. Lloyd AB, et al. Maternal and paternal parenting practices and their influence on children's adiposity, screen-time, diet and physical activity. Appetite. 2014;79:149-157.
45. Rhodes RE, et al. Predicting parental support and parental perceptions of child and youth movement behaviors. Psychol Sport Exerc. 2019;41:80-90.
46. Rhodes RE, et al. Application of the the Multi-Process Action Control Framework to understand parental support of child and youth physical activity, sleep, and screen time behaviors. Appl Psychol Heal Well-being. 2019;11(2):223-239.
47. Rhodes RE, et al. Understanding action control of parent support behavior for child physical activity. Heal Psychol. 2016;35:131-140.
48. Rhodes RE, et al. Predicting changes across 12 months in three types of parental support behaviors and mothers' perceptions of child physical activity. Ann Behav Med. 2015;49:853-864.
49. Edwardson CL, Gorely T. Parental influences on different types and intensities of physical activity in youth: A systematic review. Psychol Sport Exerc. 2010;11:522-535.
50. Rhodes RE, et al. Parental support of the Canadian 24-hour movement guidelines for children and youth: Prevalence and correlates. BMC Public Health. 2019;19(1):1385.
51. Garriguet D, et al. Parent-child association in physical activity and sedentary behaviour. Health Rep. 2017;28(5):3-11.
52. Neshteruk CD, et al. The influence of fathers on children's physical activity: A review of the literature from 2009 to 2015. Prev Med. 2017;102:12-19.
53. Canadian Health Measures Survey (CHMS) Custom Analysis.
54. Cislak A, et al. Family-related predictors of body weight and weight-related behaviours among children and adolescents: A systematic umbrella review. Child Care Health Dev. 2012;38:321-331.
55. Ash T, et al. Family-based childhood obesity prevention interventions a systematic review and quantitative content analysis. Int J Behav Nutr Phys Act. 2017:14:113.
56. Barr-Anderson DJ, et al. Family-focused physical activity, diet and obesity interventions in African-American girls: A systematic review. Obes Rev. 2013;14(1):29-51.
57. Brown HE, et al. Family-based interventions to increase physical activity in children: a systematic review, meta-analysis and realist synthesis. Pediatr Obes Prev. 2016:345–360.
58. Dellert JC, Johnson P. Interventions with children and parents to improve physical activity and body mass index: a meta-analysis. Am J Health Promot. 2014;28(4):259–267.
59. Verjans-Janssen SRB, et al. Effectiveness of school-based physical activity and nutrition interventions with direct parental involvement on children's BMI and energy balance-related behaviors – a systematic review. PLoS. 2018;13(9):1-24.
60. Robb MB. The new normal: parents, teens, screens, and sleep in the United States. San Fransisco: Common Sense Media; 2019. https://www.commonsensemedia.org/research/the-new-normal-parents-teens-and-devices-around-the-world.
61. Lauricella AR, et al. The common sense census: plugged-in parents of tweens and teens. San Francisco: Common Sense Media; 2016. https://www.commonsensemedia.org/research/the-common-sense-census-media-use-by-tweens-and-teens-2019.
62. Rideout V. The common sense census: media use in teens and teens. San Fransisco; 2019. https://www.commonsensemedia.org/sites/default/files/uploads/research/census_researchreport.pdf.
63. Sleddens EFC, et al. Bidirectional associations between activity-related parenting practices, and child physical activity, sedentary screen-based behavior and body mass index: a longitudinal analysis. Int J Behav Nutr Phys Act. 2017;14:89.
64. Thompson DA, et al. Parenting and preschooler TV viewing in low-income Mexican Americans: development of the parenting practices regarding TV viewing (PPRTV) scale. J Dev Behav Pediatr. 2016;37:465-474.
65. Kesten JM, et al. Associations between rule-based parenting practices and child screen viewing: A cross-sectional study. Prev Med Reports. 2015;2:84-89.
66. Tombeau Cost K, et al. Association of parental and contextual stressors with child screen exposure and child screen exposure combined with feeding. JAMA Netw Open. 2020;3(2):e1920557.
67. Robinson JL, et al. Relationship between parental estimate and an objective measure of child television watching. Int J Behav Nutr Phys Act. 2006;3:43.
68. Brindova D, et al. How parents can affect excessive spending of time on screen-based activities. BMC Public Health. 2014;14:1261.
69. Bounova A, et al. Home and neighborhood environment predictors of adolescents' screen viewing. J Phys Act Heal. 2016;13;1310-1316.

70. Billows M, et al. Family disorganization, sleep hygiene, and adolescent sleep disturbance. J Clin Child Adolesc Psychol. 2009;5:745-752.
71. Jones CHD, Ball H. Exploring socioeconomic differences in bedtime behaviours and sleep duration in english preschool children. Infant Child Dev. 2014;23:518-531.
72. Li S, et al. Risk factors associated with short sleep duration among Chinese school-aged children. Sleep Med. 2010;11(9):907-916.
73. Chen T, et al. Sleep duration in Chinese adolescents: biological, environmental, and behavioral predictors. Sleep Med. 2014;15:1345-1353.
74. Myoungok J, Whittemore R. The family management style framework for families of children with obesity. J Theory Constr Test. 2014;19:5-15.
75. Davison KK, et al. Reframing family-centred obesity prevention using the Family Ecological Model. Public Health Nutr. 2012;16:1861-1869.
76. Bronfenbrenner U. The Ecology of Human Development: Experiments by Nature and Design. Cambridge, MA: Harvard University Press; 1979.
77. Tremblay MS, et al. Position Statement on Active Outdoor Play. Int J Env Res Public Health. 2015;12(6):6475-6505.
78. Sterdt E, et al. Correlates of physical activity of children and adolescents: A systematic review of reviews. Heal Educ J. 2014;73:72–89.
79. Mendonça G, et al. Physical activity and social support in adolescents: A systematic review. Health Educ Res. 2014;29:822–839.
80. Laird Y, et al. The role of social support on physical activity behaviour in adolescent girls: A systematic review and meta-analysis. Int J Behav Nutr Phys Act. 2016;13:79.
81. Boxberger K, Reimers AK. Parental correlates of outdoor play in boys and girls aged 0 to 12: a systematic review. Int J Env Res Public Health. 2019;16:190.
82. Gordon-Larsen P, et al. Determinants of adolescent physical activity and inactivity patterns. Pediatrics. 2000;105:e83.
83. Rothman L, et al. The decline in active school transportation (AST): A systematic review of the factors related to AST and changes in school transport over time in North America. Prev Med. 2018;111:314-322.
84. Xu H, et al. Associations of parental influences with physical activity and screen time among young children: A systematic review. J Obes. 2015;2016:546925.
85. Cleland V, et al. A longitudinal study of the family hysical activity environment and physical activity among youth. Am J Health Promot. 2011;25:159-161.
86. Crawford D, et al. The longitudinal influence of home and neighbourhood environments on children's body mass index and physical activity over 5 years: The CLAN study. Int J Obes. 2010;34:1177–1187.
87. Carlson JA, et al. Built environment characteristics and parent active transportation are associated with active travel to school in youth age 12-15. Br J Sport Med. 2014;48:1634–1639.
88. Carson V, et al. Parents' perception of neighbourhood environment as a determinant of screen time, physical activity and active transport. Can J Public Health. 2010;101:124–127.
89. Cutumisu N, et al. Influence of area deprivation and perceived neighbourhood safety on active transport to school among urban Quebec preadolescents. Can J Public Health. 2014;105:e376-382.
90. Esteban-Cornejo I, et al. Parental and adolescent perceptions of neighborhood safety related to adolescents' physical activity in their neighborhood. Res Q Exerc Sport. 2006;87:191–199.
91. Ghekiere A, et al. Insights into children's independent mobility for transportation cycling—Which socio-ecological factors matter? J Sci Med Sport. 2017;20:267–272.
92. Blum RM, Rinehart Mann P. Reducing the Risk: Connections That Make a Difference in the Lives of Youth. Bethesda; 2012. https://files.eric.ed.gov/fulltext/ED412459.pdf.
93. Faulkner G, et al. School travel and children's physical activity: a cross-sectional study examining the influence of distance. BMC Public Health. 2013;13(1):1166.
94. Riazi NA, et al. Correlates of children's independent mobility in Canada: a multi-site study. Int J Environ Res Public Health. 2019;16(16):2862.
95. World Health Organization. WHO Guidelines on physical activity, sedentary behaviour and sleep for children under 5 years of age. 2019. https://apps.who.int/iris/handle/10665/311664.
96. Reilly JJ, et al. GRADE-ADOLOPMENT process to develop 24-hour movement behavior recommendations and physical activity guidelines for the under 5s in the UK, 2019. J Phys Act Health. 2020;17(1):101–108.
97. American Academy of Pediatrics – Council on Communications and Media. Media and young minds. Pediatrics. 2016;138(5):e20162591.
98. Australian Department of Health. Australian 24-Hour Movement Guidelines for Children and Young People (5-17 Years) – An Integration of Physical Activity, Sedentary Behaviour and Sleep. 2019. https://www1.health.gov.au/internet/main/publishing.nsf/Content/health-24-hours-phys-act-guidelines.
99. New Zealand Ministry of Health. Sit less, move more, sleep well: active play guidelines for under-fives. 2017. https://www.health.govt.nz/publication/sit-less-move-more-sleep-well-active-play-guidelines-under-fives.
100. Okely AD, et al. A collaborative approach to adopting/adapting guidelines - The Australian 24-Hour Movement Guidelines for the Early Years (birth to 5 years): An Integration of Physical Activity, Sedentary Behaviour, and Sleep. BMC Public Health. 2017;17(S5):869.
101. Kesten JM, et al. Associations between rule-based parenting practices and child screen viewing: A cross-sectional study. Prev Med Reports. 2015;2:84–89.
102. Saleem M, et al. Factors associated with excessive TV viewing in school children of Wah Cantt, Pakistan. Rawal Med J. 2014;39:323–326.
103. Goh SN, et al. Sociodemographic, home environment and parental influences on total and device-specific screen viewing in children aged 2 years and below: an observational study. BMJ Open. 2016;6:e009113.
104. Verloigne M, et al. Family- and school-based correlates of energy balance-related behaviours in 10-12-year-old children: a systematic review within the ENERGY (EuropeaN Energy balance Research to prevent excessive weight Gain among Youth) project. Public Heal Nutr. 2012;15:1380–1395.
105. van Zutphen M, et al. Association between the family environment and television viewing in Australian children. J Paediatr Child Health. 2007;43:458–463.
106. Cheng JK, et al. Parental guidance advised: associations between parental television limits and health behaviors among obese children. Acad Pediatr. 2015;15:204–209.
107. De Lepeleere S, et al. Do specific parenting practices and related parental self-efficacy associate with physical activity and screen time among primary schoolchildren? A cross-sectional study in Belgium. BMJ Open. 2005;5:e007209.
108. Downing KL, et al. Associations of Parental Rules and Socioeconomic Position With Preschool Children's Sedentary Behaviour and Screen Time. J Phys Act Health. 2015;12:515–521.
109. Ponti, M, et al. Digital Media: Promoting healthy screen use in school-aged children and dolescents. 2019. https://www.cps.ca/en/documents/position/digital-media.
110. Ponti M, et al. Screen time and young children: Promoting health and development in a digital world. Paediatr Child Health. 2017;22(8):461-468.
111. Cui Z, et al. Temporal trends and recent correlates in sedentary behaviours in Chinese children. Int J Behav Nutr Phys Act. 2011;8:93.
112. de Jong E, et al. Association between TV viewing, computer use and overweight, determinants and competing activities of screen time in 4- to 13-year-old children. Int J Obes. 2013;37:47–53.
113. Johnson L, et al. The association of parent's outcome expectations for child TV viewing with parenting practices and child TV viewing: an examination using path analysis. Int J Behav Nutr Phys Act. 2015;12:70.
114. Carson V, Janssen I. Associations between factors within the home setting and screen time among children aged 0-5 years: a cross-sectional study. BMC Public Health. 2012;12:539.
115. Hoyos Cillero I, Jago R. Sociodemographic and home environment predictors of screen viewing among Spanish school children. J Public Health. 2011;33:392–402.
116. Christakis DA, et al. Television, video, and computer game usage in children under 11 years of age. J Pediatr. 2004;145:652–656.
117. Robinson JL, et al. Relationship between parental estimate and an objective measure of child television watching. Int J Behav Nutr Phys Act. 2006;3:43.
118. Cameron AJ, et al. Television in the bedroom and increased body weight: potential explanations for their relationship among European schoolchildren. Pediatr Obes. 2013;8:130–141.
119. Hesketh K, et al. Mediators of the relationship between maternal education and children's TV viewing. Am J Prev Med. 2007;33:41–47.
120. Jiang XX, et al. Recreational screen-time among Chinese adolescents: a cross-sectional study. J Epidemiol. 2014;24:397–403.
121. Saelens BE, et al. Home environmental influences on children's television watching from early to middle childhood. J Dev Behav Pediatr. 2002;23:127–132.
122. Buxton OM, et al. Sleep in the modern family: Protective family routines for child and adolescent sleep. Sleep Health. 2015;1:15–27.
123. John B. Sleep-patterns, sleep hygiene behaviors and parental monitoring among Bahrain-based Indian adolescents. J Fam Med Prim Care. 2015;4:232–237.
124. Mindell JA, Williams A. Benefits of a bedtime routine in young children: Sleep, development, and beyond. Sleep Med Rev. 2018;40:93-108.
125. Hirshkowitz M, et al. National Sleep Foundation's updated sleep duration recommendations: final report. Sleep Heal. 2015;1(4):233-243.
126. Paruthi S, et al. Recommended amount of sleep for pediatric populations: a consensus statement of the American Academy of Sleep Medicine. J Clin Sleep Med. 2016;12:785–786.
127. Allen SL, et al. ABCs of SLEEPING: A review of the evidence behind pediatric sleep practice recommendations. Sleep Med Rev. 2016;29:1-14.
128. Brambilla P, et al. Sleep habits and pattern in 1-14 years old children and relationship with video devices use and evening and night child activities. Ital J Pediatr. 2017;43(1):7.
129. Plancoulaine S, et al. Gender-specific factors associated with shorter sleep duration at age 3 years. J Sleep Res. 2015;24:610-620.
130. Adam EK, et al. Sleep timing and quantity in ecological and family context: A nationally representative time-diary study. J Fam Psychol. 2007;21:4–19.
131. BaHammam A, et al. Sleep duration and its correlates in a sample of Saudi elementary school children. Singapore Med J. 2006;47:875.
132. Chahal H, et al. Availability and night-time use of electronic entertainment and communication devices are associated with short sleep duration and obesity among Canadian children. Pediatr Obes. 2013;8:42–51.
133. Nuutinen T, et al. Do computer use, TV viewing, and the presence of the media in the bedroom predict school-aged children's sleep habits in a longitudinal study? BMC Public Health. 2013;13:684.
134. Continente X, et al. Media devices, family relationships and sleep patterns among adolescents in an urban area. Sleep Med. 2017;32:28–35.
135. Minges KE, et al. Classroom standing desks and sedentary behavior: a systematic review. Pediatrics. 2016;137(2):e20153087.
136. Greig AA, et al. An update to the Greig Health Record: Position Statement. Paediatr Child Heal. 2016;21(5):265-268.
137. Rourke L. Rourke Baby Records. 2017. http://www.rourkebabyrecord.ca/default.
138. Public Health Agency of Canada. A Common Vision for Increasing Physical Activity and Reducing Sedentary Living in Canada: Let's Get Moving. 2018. https://www.canada.ca/en/public-health/services/publications/healthy-living/lets-get-moving.html.
139. Faulkner G, et al. Canadian 24-Hour Movement Guidelines for Children and Youth: Exploring the perceptions of stakeholders regarding their acceptability, barriers to uptake, and dissemination. Appl Physiol Nutr Metab. 2016;41(S3):S303-S310.
140. Jones LE, et al. Child cash benefits and family expenditures: evidence from the National Child Benefit. Can J Econ. 2019;52(4):1433-1463.
