# Supplementary material for: Development of a consensus statement on the role of the family in the physical activity, sedentary, and sleep behaviours of children and youth
Source: Int J Behav Nutr Phys Act. 2020 Jun 16;17:74. doi: 10.1186/s12966-020-00973-0 (PMC7296673; doi:10.1186/s12966-020-00973-0)
Supplement: Supplementary file 2 — Additional file 2. Table S1 (docx). Inclusion and exclusion criteria for systematic literature searches on family and the physical activity (review #1), sedentary (review #2), and sleep (review #3) behaviours of children and youth. [file 12966_2020_973_MOESM2_ESM.docx]

**Table S1. Inclusion and exclusion criteria for systematic literature searches on family and the physical activity (review #1), sedentary (review #2), and sleep (review #3) behaviours of children and youth.**

| **Criterion** | **Inclusion criteria** | **Exclusion criteria** |
| --- | --- | --- |
| Language | English | Non-English studies |
| Type of article | Original research, published in a peer review journal | Articles that were not peer-reviewed or original research. |
| Study design | All study types |  |
| Study focus | Studies that focus on modifiable (e.g., parent behaviours and parent-child relationships) and non-modifiable (e.g., family structure, socio-economic status) characteristics of the family.  Studies that examine the independent influence of family characteristics on children/youth’s physical activity/sedentary behaviour/sleep. | Articles that examine collective impact of various socializing agents (family, peers, and school/teachers) on children/youth’s physical activity/sedentary behaviour/sleep.  Articles that examine the impact of family characteristics on child/youth physical activity/sedentary behaviour/sleep during pregnancy.  Multicomponent interventions that actively target the family environment as well as other settings/environments (e.g., school-based interventions) |
| Literature focus | Articles where the focus is on the influence of family, which refers to those living in the same household as the child/youth. This includes, but is not limited to, the mother, father, siblings, caregiver, or other legal guardian. | Articles where the focus is on the influence of children/youth’s physical activity/sedentary behaviour/sleep on family members.  Articles that examine the total influence of socializing agents (family, peers, and school/teachers) on children/youth’s physical activity/sedentary behaviour/sleep |
| Population sample | Children and youth ($\leq$18 years). | Articles that target children/youth with a specific disease, illness, or condition, with the exception of children/youth with overweight/obesity. |
