# Supplementary material for: Development of a consensus statement on the role of the family in the physical activity, sedentary, and sleep behaviours of children and youth
Source: Int J Behav Nutr Phys Act. 2020 Jun 16;17:74. doi: 10.1186/s12966-020-00973-0 (PMC7296673; doi:10.1186/s12966-020-00973-0)
Supplement: Supplementary file 3 — Additional file 3. Review #1 (docx.). Search Process for Family and Physical Activity Literature Review (review #1). Themes from the physical activity literature review (review #1). References for the papers included in the family and physical activity review (review #1), organized by theme. [file 12966_2020_973_MOESM3_ESM.docx]

Records identified through databases searching
(n = 15,242)

Records after duplicates removed
(n = 10,381)

Records screened
(n = 739)

Identification

Eligibility

Screening

Records excluded

(n = 9,642)

**Search Process for Family and Physical Activity Literature Review (review #1).**

**Themes from the physical activity literature review (review #1).**

| **Theme** | **Topics Included** | **Number of studies** |
| --- | --- | --- |
| Physical activity modelling | Parental physical activity  Sibling physical activity  Perceptions of others’ physical activity  Co-participation in physical activity | 359 |
| Parental emotional support | Support/encouragement  Watching/attending physical activities Talking about children’s physical activity | 233 |
| Sociodemographic factors | Parental age  Parental education  Parental income  Parental employment status  Race/ethnicity | 172 |
| Parental beliefs, attitudes, knowledge | Perceived importance of physical activity  Attitudes towards physical activity/sport  Parental perceptions of child’s physical abilities  Perceived neighbourhood safety | 131 |
| Parental instrumental support | Transportation to physical activity events  Access to physical activity equipment (e.g., bicycle)  Paying for sport/active extracurricular activities | 60 |
| Parental monitoring | Monitoring of physical activity  Monitoring/supervision of outdoor time | 50 |
| Parental efficacy and motivation | Self-efficacy to support child’s physical activity  Motivation towards child’s engagement in physical activity | 43 |
| Family structure | Living in nuclear, reconstituted, or single-headed households  Living with biological parent vs non-resident parent  Living with other guardians, such as grandparents  Number of siblings  Age of siblings | 40 |
| Parenting style | Authoritative  Controlling/restrictive  Permissive  Uninvolved  Autonomy-supportive | 37 |
| Parental health | Maternal depression  Body mass index | 28 |
| Parent-child relationship | Strength/quality of relationship  Tension/conflictual | 21 |
| Family environment | Chaotic/disorganized family  Connection/cohesiveness  Family stress | 15 |

**References for the papers included in the family and physical activity review (review #1), organized by theme.**

**Physical activity modelling (n=359)**

(e.g., parental physical activity, sibling physical activity, co-participation in physical activity)

1. Aarnio M, Winter T, Kujala UM, Kaprio J. Familial aggregation of leisure-time physical activity - A three generation study. Int J Sports Med. 1997;18:549-556.
2. Abbott G, Hnatiuk J, Timperio A, Salmon J, Best K, Hesketh KD. Cross-sectional and longitudinal associations between parents’ and preschoolers’ physical activity and television viewing: The HAPPY study. J Phys Act Health. 2016;13:269-274.
3. Adkins S, Sherwood NE, Story M, Davis M. Physical activity among African-American girls: The role of parents and the home environment. Obes Res. 2004;12:38-45.
4. Alderman BL, Benham-Deal TB, Jenkins JM. Change in parental influence on children’s physical activity over time. J Phys Act Health. 2010;7:60-67.
5. Alhassan S, Nwaokelemeh O, Greever CJ, Burkart S, Ahmadi M, St Laurent CW, et al. Effect of a culturally-tailored mother-daughter physical activity intervention on pre-adolescent African-American girls’ physical activity levels. Prev Med reports. 2018;11:7-14.
6. Ali MM, Amialchuk A, Heiland FW. Weight-related behavior among adolescents: The role of peer effects. PLoS One. 2011;6:e21179.
7. Anderssen N, Wold B. Parental and peer influences on leisure-time physical activity in young adolescents. Res Q Exerc Sport. 1992;63:341-348.
8. Anderssen N, Wold B, Torsheim T. Are parental health habits transmitted to their children? An eight year longitudinal study of physical activity in adolescents and their parents. J Adolesc. 2006;29:513-524.
9. Ansari H, Farajzadegan Z, Hajigholami A, Paknahad Z. A randomized field trial for the primary prevention of osteoporosis among adolescent females: Comparison of two methods, mother centered and daughter centered. J Res Med Sci. 2014;19:746-752.
10. Anselma M, Maidy Chinapaw MJ, Altenburg TM. Determinants of child health behaviors in a disadvantaged area from a community perspective: A participatory needs assessment. Int J Environ Res Public Health. 2018;15:644.
11. Arauz Boudreau AD, Kurowski DS, Gonzalez WI, Dimond MA, Oreskovic NM. Latino families, primary care, and childhood obesity: A randomized controlled trial. Am J Prev Med. 2013;44:247-257.
12. Arredondo EM, Morello M, Holub C, Haughton J. Feasibility and preliminary findings of a church-based mother-daughter pilot study promoting physical activity among young Latinas. Fam Community Health. 2014;37:6-18.
13. Baker KM, Healy S, Rice DJ, Garcia JM. Adolescent weight and health behaviors and their associations with individual, social, and parental factors. J Phys Act Health. 2018;15: 912-917
14. Barkin SL, Lamichhane AP, Banda JA, JaKa MM, Buchowski MS, Evenson KR, et al. Parent’s physical activity associated with preschooler activity in underserved populations. Am J Prev Med. 2017;52:424-432.
15. Barnes AT, Plotnikoff RC, Collins CE, Morgan PJ. Maternal correlates of objectively measured physical activity in girls. Matern Child Health J. 2015;19:2348-2357.
16. Barr-Anderson DJ, Adams-Wynn AW, DiSantis KI, Kumanyika S. Family-focused physical activity, diet and obesity interventions in African-American girls: A systematic review. Obes Rev. 2013;14:29-51.
17. Barr-Anderson DJ, Adams-Wynn AW, Orekoya O, Alhassan S. Socio-cultural and environmental factors that influence weight-related behaviors: Focus group results from African-American girls and their mothers. Int J Environ Res Public Health. 2018;15:1354
18. Baskin ML, Dulin-Keita A, Thind H, Godsey E. Social and cultural environment factors influencing physical activity among african-american adolescents. J Adolesc Health. 2015;56:536-542.
19. Bastos JP, Araujo CLP, Hallal PC. Prevalence of insufficient physical activity and associated factors in Brazilian adolescents. J Phys Act Health. 2008;5:777-794.
20. Bauer KW, Neumark-Sztainer D, Hannan PJ, Fulkerson JA, Story M. Relationships between the family environment and school-based obesity prevention efforts: Can school programs help adolescents who are most in need?. Health Educ Res. 2011;26:675-688.
21. Bauer KW, Berge JM, Neumark-Sztainer D. The importance of families to adolescents’ physical activity and dietary intake. Adolesc Med State Art Rev. 2011;22:601-613.
22. Bauer KW, Neumark-Sztainer D, Fulkerson JA, Hannan PJ, Story M. Familial correlates of adolescent girls’ physical activity, television use, dietary intake, weight, and body composition. Int J Behav Nutr Phys Act. 2011;8:25.
23. Bautista-Castano I, Doreste J, Serra-Majem L. Effectiveness of interventions in the prevention of childhood obesity. Eur J Epidemiol. 2004;19:617-622.
24. Baxter-Jones ADG, Maffulli N, Group TS. Parental influence on sport participation in elite young athletes. J Sports Med Phys Fitness. 2003;43:250-255.
25. Beets MW, Foley JT. Association of father involvement and neighborhood quality with kindergartners’ physical activity: A multilevel structural equation model. Am J Health Promot. 2008;22:195-203.
26. Belanger-Gravel A, Gauvin L, Lagarde F, Laferte M. Correlates and moderators of physical activity in parent-tween dyads: A socio-ecological perspective. Public Health. 2015;129:1218-1223.
27. Bellows LL, McCloskey M, Clark L, Thompson DA, Bekelman TA, Chamberlin B, et al. HEROs: design of a mixed-methods formative research phase for an ecocultural intervention to promote healthy eating and activity behaviors in rural families with preschoolers. J Nutr Educ Behav. 2018;50:736-745.
28. Berge JM, Meyer C, MacLehose RF, Eisenberg ME, Neumark-Sztainer D. Nonresident parental influence on adolescent weight and weight-related behaviors: Similar or different from resident parental influence? Int J Behav Nutr Phys Act. 2014;11:131.
29. Berge JM, Saelens BE. Familial influences on adolescents’ eating and physical activity behaviors. Adolesc Med State Art Rev. 2012;23:424-439.
30. Bergmann GG, Bergmann ML de A, Marques AC, Hallal PC. Prevalence of physical inactivity and associated factors among adolescents from public schools in Uruguaiana, Rio Grande do Sul State, Brazil. Cad Saude Publica. 2013;29:2217-2229.
31. Bergmann K, Mestre Z, Strong D, Eichen DM, Rhee K, Crow S, et al. Comparison of two models of family-based treatment for childhood obesity: A pilot study. Child Obes. 2019;15:116-122.
32. Beutum MN, Cordier R, Bundy A. Comparing activity patterns, biological, and family factors in children with and without developmental coordination disorder. Phys Occup Ther Pediatr. 2013;33:174-185.
33. Bishop P, Donnelly JE. Home based activity program for obese children. Am Correct Ther J. 1987;41:12-19.
34. Bogaert N, Steinbeck KS, Baur LA, Brock K, Bermingham MA. Food, activity and family--environmental vs biochemical predictors of weight gain in children. Eur J Clin Nutr. 2003;57:1242-1249.
35. Boomsma DI, van den Bree MB, Orlebeke JF, Molenaar PC. Resemblances of parents and twins in sports participation and heart rate. Behav Genet. 1989;19:123-141.
36. Boutelle KN, Braden A, Douglas JM, Rhee KE, Strong D, Rock CL, et al. Design of the FRESH study: A randomized controlled trial of a parent-only and parent-child family-based treatment for childhood obesity. Contemp Clin Trials. 2015;45:364-370.
37. Boutelle KN, Rhee KE, Liang J, Braden A, Douglas J, Strong D, et al. Effect of attendance of the child on body weight, energy intake, and physical activity in childhood obesity treatment: A randomized clinical trial. JAMA Pediatr. 2017;171:622-628.
38. Bringolf-Isler B, Schindler C, Kayser B, Suggs LS, Probst-Hensch N, Group SS. Objectively measured physical activity in population-representative parent-child pairs: Parental modelling matters and is context-specific. BMC Public Health. 2018;18:1024.
39. Brouwer SI, Kupers LK, Kors L, Sijtsma A, Sauer PJJ, Renders CM, et al. Parental physical activity is associated with objectively measured physical activity in young children in a sex-specific manner: The GECKO Drenthe cohort. BMC Public Health. 2018;18:1033.
40. Brown NA, Smith KC, Thornton RLJ, Bowie JV, Surkan PJ, Thompson DA, et al. Gathering perspectives on extended family influence on African American children’s physical activity. J Health Dispar Res Pract. 2015;8:10-24.
41. Brunet J, Gaudet J, Wing W, Belanger M. Parents’ participation in physical activity predicts maintenance of some, but not all, types of physical activity in offspring during early adolescence: A prospective longitudinal study. J Sport Heal Sci. 2019.8:273-279
42. Brzek A, Strauss M, Przybylek B, Dworrak T, Dworrak B, Leischik R. How does the activity level of the parents influence their children’s activity? The contemporary life in a world ruled by electronic devices. Arch Med Sci. 2018;14:190-198.
43. Burchett HED, Sutcliffe K, Melendez-Torres GJ, Rees R, Thomas J. Lifestyle weight management programmes for children: A systematic review using qualitative comparative analysis to identify critical pathways to effectiveness. Prev Med (Baltim). 2018;106:1-12.
44. Burns RD. Enjoyment, self-efficacy, and physical activity within parent-adolescent dyads: Application of the actor-partner interdependence model. Prev Med (Baltim). 2019;126:105756.
45. Buscemi J, Berlin KS, Rybak TM, Schiffer LA, Kong A, Stolley MR, et al. Health behavior and weight changes among ethnic and racial minority preschoolers and their parents: Associations across 1 year. J Pediatr Psychol. 2016;41:777-785.
46. Cabanas-Sanchez V, Garcia-Cervantes L, Esteban-Gonzalo L, Girela-Rejon MJ, Castro-Pinero J, Veiga OL. Social correlates of sedentary behavior in young people: The UP&DOWN study. J Sport Heal Sci. 2020;9:189-196
47. Cameron AJ, Ball K, Hesketh KD, McNaughton SA, Salmon J, Crawford DA, et al. Variation in outcomes of the Melbourne infant, feeding, activity and nutrition trial (InFANT) program according to maternal education and age. Prev Med (Baltim). 2014;58:58-63.
48. Campbell KJ, Hesketh KD. Strategies which aim to positively impact on weight, physical activity, diet and sedentary behaviours in children from zero to five years: A systematic review of the literature. Obes Rev. 2007;8:327-338.
49. Campbell KJ, Hesketh KD, McNaughton SA, Ball K, McCallum Z, Lynch J, et al. The extended infant feeding, activity and nutrition trial (InFANT extend) program: A cluster-randomized controlled trial of an early intervention to prevent childhood obesity. BMC Public Health. 2016;16:166.
50. Campbell K, Hesketh K, Crawford D, Salmon J, Ball K, McCallum Z. The infant feeding activity and nutrition yrial (INFANT) an early intervention to prevent childhood obesity: Cluster-randomised controlled trial. BMC Public Health. 2008;8:103.
51. Cantell M, Crawford SG, Dewey D. Daily physical activity in young children and their parents: A descriptive study. Paediatr Child Health. 2012;17:e20-4.
52. Carbert NS, Brussoni M, Geller J, Masse LC. Familial environment and overweight/obese adolescents’ physical activity. Int J Environ Res Public Health. 2019;16:2558.
53. Carlson JA, Sallis JF, Kerr J, Conway TL, Cain K, Frank LD, et al. Built environment characteristics and parent active transportation are associated with active travel to school in youth age 12-15. Br J Sports Med. 2014;48:1634-1639.
54. Carson V. Cross-sectional and longitudinal associations between parental support and children’s physical activity in the early years. J Phys Act Health. 2016;13:611-616.
55. Cerin E, Baranowski T, Barnett A, Butte N, Hughes S, Lee RE, et al. Places where preschoolers are (in)active: An observational study on Latino preschoolers and their parents using objective measures. Int J Behav Nutr Phys Act. 2016;13:29.
56. Chen J-L, Guo J, Esquivel JH, Chesla CA. Like mother, like child: The influences of maternal attitudes and behaviors on weight-related health behaviors in their children. J Transcult Nurs Off J Transcult Nurs Soc. 2018;29:523-531.
57. Chen J-L, Weiss S, Heyman MB, Lustig RH. Efficacy of a child-centred and family-based program in promoting healthy weight and healthy behaviors in Chinese American children: A randomized controlled study. J Public Health (Oxf). 2010;32:219-229.
58. Cheng LA, Mendonca G, Farias Junior JC de. Physical activity in adolescents: Analysis of the social influence of parents and friends. J Pediatr (Rio J). 2014;90:35-41.
59. Chiarlitti NA, Kolen AM. Parental influences and the relationship to their children’s pysical activity levels. Int J Exerc Sci. 2017;10:205-212.
60. Christofaro DGD, Turi-Lynch BC, Lynch KR, Tebar WR, Fernandes RA, Tebar FG, et al. Parents’ lifestyle, sedentary behavior, and physical activity in their children: A cross-sectional study in Brazil. J Phys Act Health. 2019;16:631-636.
61. Christofaro DGD, Andersen LB, Andrade SM de, Barros MVG, Saraiva BTC, Fernandes RA, et al. Adolescents’ physical activity is associated with previous and current physical activity practice by their parents. J Pediatr (Rio J). 2018;94:48-55.
62. Cislak A, Safron M, Pratt M, Gaspar T, Luszczynska A. Family-related predictors of body weight and weight-related behaviours among children and adolescents: A systematic umbrella review. Child Care Health Dev. 2012;38:321-331.
63. Cleland V, Venn A, Fryer J, Dwyer T, Blizzard L. Parental exercise is associated with Australian children’s extracurricular sports participation and cardiorespiratory fitness: A cross-sectional study. Int J Behav Nutr Phys Act. 2005;2:3.
64. Cleland V, Timperio A, Salmon J, Hume C, Telford A, Crawford D. A longitudinal study of the family physical activity environment and physical activity among youth. Am J Health Promot. 2011;25:159-167.
65. Cliff DP, Okely AD, Morgan PJ, Steele JR, Jones RA, Colyvas K, et al. Movement skills and physical activity in obese children: Randomized controlled trial. Med Sci Sports Exerc. 2011;43:90-100.
66. Condessa LA, Chaves OC, Silva FM, Malta DC, Caiaffa WT. Sociocultural factors related to the physical activity in boys and girls: PeNSE 2012. Rev Saude Publica. 2019;53:25.
67. Cookson S, Heath A, Bertrand L. The heartsmart family fun pack: An evaluation of family-based intervention for cardiovascular risk reduction in children. Can J Public Health. 2000;91:256-259.
68. Coto J, Pulgaron ER, Graziano PA, Bagner DM, Villa M, Malik JA, et al. Parents as role models: Associations between parent and young children’s weight, dietary intake, and physical activity in a minority sample. Matern Child Health J. 2019;23:943-950.
69. Cottrell L, Spangler-Murphy E, Minor V, Downes A, Nicholson P, Neal WA. A kindergarten cardiovascular risk surveillance study: CARDIAC-kinder. Am J Health Behav. 2005;29:595-606.
70. Craig CL, Cameron C, Tudor-Locke C. Relationship between parent and child pedometer-determined physical activity: A sub-study of the CANPLAY surveillance study. Int J Behav Nutr Phys Act. 2013;10:8.
71. Craven MR, Keefer L, Rademaker A, Dykema-Engblade A, Sanchez-Johnsen L. Social support for exercise as a predictor of weight and physical activity status among Puerto Rican and Mexican men: Results from the Latino men’s health initiative. Am J Mens Health. 2018;12:766-778.
72. Crawford D, Cleland V, Timperio A, Salmon J, Andrianopoulos N, Roberts R, et al. The longitudinal influence of home and neighbourhood environments on children’s body mass index and physical activity over 5 years: The CLAN study. Int J Obes (Lond). 2010;34:1177-1187.
73. Crespo NC, Corder K, Marshall S, Norman GJ, Patrick K, Sallis JF, et al. An examination of multilevel factors that may explain gender differences in children’s physical activity. J Phys Act Health. 2013;10:982-992.
74. D’Haese S, Timperio A, Veitch J, Cardon G, Van Dyck D, Salmon J. Neighborhood perceptions moderate the association between the family environment and children’s objectively assessed physical activity. Health Place. 2013;24:203-209.
75. da Costa BGG, da Silva KS, da Silva JA, Minatto G, de Lima LRA, Petroski EL. Sociodemographic, biological, and psychosocial correlates of light- and moderate-to-vigorous-intensity physical activity during school time, recesses, and physical education classes. J Sport Heal Sci. 2019;8:177-182.
76. Dan SP, Mohd NMT, Zalilah MS. Determination of factors associated with physical activity levels among adolescents attending school in Kuantan, Malaysia. Malays J Nutr. 2011;17:175-187.
77. Davis AM, Daldalian MC, Mayfield CA, Dean K, Black WR, Sampilo ML, et al. Outcomes from an urban pediatric obesity program targeting minority youth: The Healthy Hawks program. Child Obes. 2013;9:492-500.
78. Davis AM, Sampilo M, Gallagher KS, Landrum Y, Malone B. Treating rural pediatric obesity through telemedicine: Outcomes from a small randomized controlled trial. J Pediatr Psychol. 2013;38:932-943.
79. Davison KK, Edmunds L, Wyker BA, Young LM, Sarfoh V. S, Sekhobo JP. Feasibility of increasing childhood outdoor play and decreasing television viewing through a family-based intervention in WIC, New York State, 2007-2008. Prev Chronic Dis. 2011;8:54.
80. Davison KK, Jurkowski JM, Li K, Kranz S, Lawson HA. A childhood obesity intervention developed by families for families: Results from a pilot study. Int J Behav Nutr Phys Act. 2013;10:3.
81. Davison KK, Cutting TM, Birch LL. Parents’ activity-related parenting practices predict girls’ physical activity. Med Sci Sports Exerc. 2003;35:1589-1595.
82. Davison KK, Jago R. Change in parent and peer support across ages 9 to 15 yr and adolescent girls’ physical activity. Med Sci Sports Exerc. 2009;41:1816-1825.
83. De Bock F, Genser B, Raat H, Fischer JE, Renz-Polster H. A participatory physical activity intervention in preschools: A cluster randomized controlled trial. Am J Prev Med. 2013;45:64-74.
84. De Lepeleere S, De Bourdeaudhuij I, Cardon G, Verloigne M. Do specific parenting practices and related parental self-efficacy associate with physical activity and screen time among primary schoolchildren? A cross-sectional study in Belgium. BMJ Open. 2015;5:e007209.
85. De Lepeleere S, De Bourdeaudhuij I, Cardon G, Verloigne M. The effect of an online video intervention “Movie Models” on specific parenting practices and parental self-efficacy related to children’s physical activity, screen-time and healthy diet: A quasi experimental study. BMC Public Health. 2017;17:366.
86. Dearth-Wesley T, Gordon-Larsen P, Adair LS, Zhang B, Popkin BM. Longitudinal, cross-cohort comparison of physical activity patterns in Chinese mothers and children. Int J Behav Nutr Phys Act. 2012;9:39.
87. Deflandre A, Lorant J, Gavarry O, Falgairette G. Determinants of physical activity and physical and sports activities in French school children. Percept Mot Skills. 2001;92:399-414.
88. Delaney C, Eck K, Byrd-Bredbenner C. Child physical activity propensity and parent physical activity cognitions behaviors and the home environment (P16-011-19). Curr Dev Nutr. 2019;3:nzz050
89. Dellert JC, Johnson P. Interventions with children and parents to improve physical activity and body mass index: A meta-analysis. Am J Health Promot. 2014;28:259-267.
90. Diep CS, Leung R, Thompson D, Gor BJ, Baranowski T. Physical activity behaviors and influences among Chinese-American children aged 9-13 years: A qualitative study. J Immigr Minor Heal. 2017;19:358-366.
91. DiLorenzo TM, Stucky-Ropp RC, Vander Wal JS, Gotham HJ. Determinants of exercise among children. II. A longitudinal analysis. Prev Med (Baltim). 1998;27:470-477.
92. Divyasree P, Kumar GD, Subitha L, Ramesh RS. Level, motivation and barriers to participate in physical activity among late adolescents in Puducherry. Int J Adolesc Med Health. 2018;0:pp.
93. Dlugonski D, DuBose KD, Rider P. Accelerometer-measured patterns of shared physical activity among mother-young child dyads. J Phys Act Health. 2017;14:808-814.
94. Dollman J. Changing associations of Australian parents’ physical activity with their children’s sport participation: 1985 to 2004. Aust N Z J Public Health. 2010;34:578-582.
95. Dong F, Howard AG, Herring AH, Thompson AL, Adair LS, Popkin BM, et al. Parent-child associations for changes in diet, screen time, and physical activity across two decades in modernizing China: China Health and Nutrition Survey 1991-2009. Int J Behav Nutr Phys Act. 2016;13:118.
96. Dowda M, Pfeiffer KA, Brown WH, Mitchell JA, Byun W, Pate RR. Parental and environmental correlates of physical activity of children attending preschool. Arch Pediatr Adolesc Med. 2011;165:939-944.
97. Downward P, Hallmann K, Pawlowski T. Assessing parental impact on the sports participation of children: A socio-economic analysis of the UK. Eur J Sport Sci. 2014;14:84-90.
98. Drenowatz C, Erkelenz N, Wartha O, Brandstetter S, Steinacker JM. Parental characteristics have a larger effect on children’s health behaviour than their body weight. Obes Facts. 2014;7:388-398.
99. Dumith SC, Gigante DP, Domingues MR, Hallal PC, Menezes AMB, Kohl HW 3rd. Predictors of physical activity change during adolescence: A 3.5-year follow-up. Public Health Nutr. 2012;15:2237-2245.
100. Duncan SC, Strycker LA, Chaumeton NR. Personal, family, and peer correlates of general and sport physical activity among African American, Latino, and White Girls. J Health Dispar Res Pract. 2015;8:12-28.
101. Dunton GF, Liao Y, Almanza E, Jerrett M, Spruijt-Metz D, Chou CP, et al. Joint physical activity and sedentary behavior in parent-child pairs. Med Sci Sports Exerc. 2012;44(8 PG-1473-80):1473-1480.
102. Edwardson CL, Gorely T, Pearson N, Atkin A. Sources of activity-related social support and adolescents’ objectively measured after-school and weekend physical activity: Gender and age differences. J Phys Act Heal. 2013;10:1153-1158.
103. Edwardson CL, Gorely T. Activity-related parenting practices and children’s objectively measured physical activity. Pediatr Exerc Sci. 2010;22:105-113.
104. Eime RM, Harvey JT, Craike MJ, Symons CM, Payne WR. Family support and ease of access link socio-economic status and sports club membership in adolescent girls: A mediation study. Int J Behav Nutr Phys Act. 2013;10:50.
105. Ek A, Delisle Nystrom C, Chirita-Emandi A, Tur JA, Nordin K, Bouzas C, et al. A randomized controlled trial for overweight and obesity in preschoolers: The More and Less Europe study - an intervention within the STOP project. BMC Public Health. 2019;19:945.
106. Eriksson M, Nordqvist T, Rasmussen F. Associations between parents’ and 12-year-old children’s sport and vigorous activity: The role of self-esteem and athletic competence. J Phys Act Health. 2008;5:359-373.
107. Erkelenz N, Kobel S, Kettner S, Drenowatz C, Steinacker JM. Parental Activity as influence on children`s BMI percentiles and physical activity. J Sports Sci Med. 2014;13:645-650.
108. Essery E V, DiMarco NM, Rich SS, Nichols DL. Mothers of preschoolers report using less pressure in child feeding situations following a newsletter intervention. J Nutr Educ Behav. 2008;40:110-115.
109. Fernandes RA, Christofaro DGD, Milanez VF, Casonatto J, Cardoso JR, Ronque ERV, et al. Physical activity: Rate, related factors, and association between parents and children. Rev Paul Pediatr. 2011;29:54-59.
110. Ferreira I, van der Horst K, Wendel-Vos W, Kremers S, van Lenthe FJ, Brug J. Environmental correlates of physical activity in youth - A review and update. Obes Rev. 2007;8:129-154.
111. Fiese BH. Playful relationships: A contextual analysis of mother-toddler interaction and symbolic play. Child Dev. 1990;61:1648-1656.
112. Finni T, Saakslahti A, Laukkanen A, Pesola A, Sipila S. A family based tailored counselling to increase non-exercise physical activity in adults with a sedentary job and physical activity in their young children: Design and methods of a year-long randomized controlled trial. BMC Public Health. 2011;11:944.
113. Fisher A, Saxton J, Hill C, Webber L, Purslow L, Wardle J. Psychosocial correlates of objectively measured physical activity in children. Eur J Public Health. 2011;21:145-150.
114. Fogelholm M, Nuutinen O, Pasanen M, Myohanen E, Saatela T. Parent-child relationship of physical activity patterns and obesity. Int J Obes Relat Metab Disord. 1999;23:1262-1268.
115. Forthofer M, Dowda M, O’Neill JR, Addy CL, McDonald S, Reid L, et al. Effect of child gender and psychosocial factors on physical activity from fifth to sixth grade. J Phys Act Health. 2017;14:953-958.
116. Fuemmeler BF, Anderson CB, Masse LC. Parent-child relationship of directly measured physical activity. Int J Behav Nutr Phys Act. 2011;8:17.
117. Garcia Bengoechea E, Ruiz Juan F, Bush PL. Delving into the social ecology of leisure-time physical activity among adolescents from south eastern Spain. J Phys Act Health. 2013;10:1136-1144.
118. Garriguet D, Colley R, Bushnik T. Parent-child association in physical activity and sedentary behaviour. Heal reports. 2017;28:3-11.
119. Geckova A., Pudelsky M, Van Dijk JP. Parenteral influence on smoking, alcohol consumption and sports activities of adolescents. Ces a Slov Psychiatr. 2000;96:406-410.
120. Gerards SMPL, Dagnelie PC, Gubbels JS, van Buuren S, Hamers FJ, Jansen MW, et al. The effectiveness of lifestyle triple P in the Netherlands: A randomized controlled trial. PLoS One. 2015;10:e0122240.
121. Ghekiere A, Carver A, Veitch J, Salmon J, Deforche B, Timperio A. Does parental accompaniment when walking or cycling moderate the association between physical neighbourhood environment and active transport among 10-12 year olds?. J Sci Med Sport. 2016;19:149-153.
122. Gottlieb NH, Chen MS. Sociocultural correlates of childhood sporting activities: their implications for heart health. Soc Sci Med. 1985;21:533-539.
123. Greenberg RS, Ariza AJ, Binns HJ. Activity and dietary habits of mothers and children: Close ties. Clin Pediatr (Phila). 2010;49:1026-1032.
124. Griffith JR, Clasey JL, King JT, Kryscio, H.S. B. Role of parents in determining children’s physical activity. World J Pediatr. 2007;3:265-270.
125. Gustafson SL, Rhodes RE. Parental correlates of physical activity in children and early adolescents. Sports Med. 2006;36:79-97.
126. HA A, Ng JYY, Lonsdale C, Lubans DR, Ng FF. Promoting physical activity in children through family-based intervention: Protocol of the “Active 1 + FUN” randomized controlled trial. BMC Public Health. 2019;19:218.
127. Haerens L, Deforche B, Maes L, Cardon G, Stevens V, De Bourdeaudhuij I. Evaluation of a 2-year physical activity and healthy eating intervention in middle school children. Health Educ Res. 2006;21:911-921.
128. Hammersley ML, Okely AD, Batterham MJ, Jones RA. An internet-based childhood obesity prevention program (Time2bHealthy) for parents of preschool-aged children: Randomized controlled trial. J Med Internet Res. 2019;21:e11964.
129. Hartson KR, Gance-Cleveland B, Amura CR, Schmiege S. Correlates of physical activity and sedentary behaviors among overweight Hispanic school-aged children. J Pediatr Nurs. 2018;40:1-6.
130. Hearst MO, Patnode CD, Sirard JR, Farbakhsh K, Lytle LA. Multilevel predictors of adolescent physical activity: A longitudinal analysis. Int J Behav Nutr Phys Act. 2012;9:8.
131. Heitzler CD, Martin SL, Duke J, Huhman M. Correlates of physical activity in a national sample of children aged 9-13 years. Prev Med (Baltim). 2006;42:254-260.
132. Hendrie GA, Coveney J, Cox DN. Defining the complexity of childhood obesity and related behaviours within the family environment using structural equation modelling. Public Health Nutr. 2012;15:48-57.
133. Henne HM, Tandon PS, Frank LD, Saelens BE. Parental factors in children’s active transport to school. Public Health. 2014;128:643-646.
134. Hennessy E, Hughes SO, Goldberg JP, Hyatt RR, Economos CD. Parent-child interactions and objectively measured child physical activity: A cross-sectional study. Int J Behav Nutr Phys Act. 2010;7:71.
135. Henriksen PW, Ingholt L, Rasmussen M, Holstein BE. Physical activity among adolescents: The role of various kinds of parental support. Scand J Med Sci Sports. 2016;26:927-932.
136. Heredia NI, Ranjit N, Warren JL, Evans AE. Association of parental social support with energy balance-related behaviors in low-income and ethnically diverse children: A cross-sectional study. BMC Public Health. 2016;16:1182.
137. Herman A, Nelson BB, Teutsch C, Chung PJ. “Eat Healthy, Stay Active!”: A coordinated intervention to improve nutrition and physical activity among Head Start parents, staff, and children. Am J Health Promot. 2012;27:e27-36.
138. Hesketh KR, Lakshman R, van Sluijs EMF. Barriers and facilitators to young children’s physical activity and sedentary behaviour: A systematic review and synthesis of qualitative literature. Obes Rev. 2017;18:987-1017.
139. Hesketh KR, Brage S, Cooper C, Godfrey KM, Harvey NC, Inskip NM, et al. The association between maternal-child physical activity levels at the transition to formal schooling: Cross-sectional and prospective data from the Southampton Women’s Survey. Int J Behav Nutr Phys Act. 2019;16:23.
140. Hesketh KR, Goodfellow L, Ekelund U, McMinn AM, Godfrey KM, Inskipet HM, al. Activity levels in mothers and their preschool children. Pediatrics. 2014;133:e973-80.
141. Hesketh KR, O’Malley C, Paes VM, Moore H, Summerbell C, Ong KK, et al. Determinants of change in physical activity in children 0-6 years of age: A systematic review of quantitative literature. Sports Med. 2017;47:1349-1374.
142. Hnatiuk JA, DeDecker E, Hesketh KD, Cardon G. Maternal-child co-participation in physical activity-related behaviours: Prevalence and cross-sectional associations with mothers and children’s objectively assessed physical activity levels. BMC Public Health. 2017;17:506.
143. Hnatiuk JA, Hesketh KR, van Sluijs EMF. Correlates of home and neighbourhood-based physical activity in UK 3-4-year-old children. Eur J Public Health. 2016;26:947-953.
144. Hnatiuk JA, Ridgers ND, Salmon J, Hesketh KD. Maternal correlates of young children’s physical activity across periods of the day. J Sci Med Sport. 2017;20:178-183.
145. Hnatiuk J, Salmon J, Campbell KJ, Ridgers ND, Hesketh KD. Early childhood predictors of toddlers’ physical activity: Longitudinal findings from the Melbourne InFANT program. Int J Behav Nutr Phys Act. 2013;10:123.
146. Holm K, Wyatt H, Murphy J, Hill J, Odgen L. Parental influence on child change in physical activity during a family-based intervention for child weight gain prevention. J Phys Act Heal. 2012;9:661-669.
147. Holt NL, Cunningham C-T, Sehn ZL, Spence JC, Newton AS, Ball GDC. Neighborhood physical activity opportunities for inner-city children and youth. Health Place. 2009;15:1022-1028.
148. Hopwood MJ, Farrow D, MacMahon C, Baker J. Sibling dynamics and sport expertise. Scand J Med Sci Sports. 2015;25:724-733.
149. Horimoto AR, Giolo SR, Oliveira CM, Alvim RO, Soler JP, de Andrade M, et al. Heritability of physical activity traits in Brazilian families: The Baependi heart study. BMC Med Genet. 2011;12:155.
150. Hosseini SV, Anoosheh M, Abbaszadeh A, Ehsani M. Qualitative Iranian study of parents’ roles in adolescent girls’ physical activity habit development. Nurs Health Sci. 2013;15:207-212.
151. Hoyos-Quintero AM, Garcia-Perdomo HA. Factors related to physical activity in early childhood: A systematic review. J Phys Act Health. 2019:1-12.
152. Hsu Y-W, Chou C-P, Nguyen-Rodriguez ST, McClain AD, Belcher BR, Spruijt-Metz D. Influences of social support, perceived barriers, and negative meanings of physical activity on physical activity in middle school students. J Phys Act Heal. 2011;8:210-219.
153. Huang WY, Wong SH, Salmon J. Correlates of physical activity and screen-based behaviors in Chinese children. J Sci Med Sport. 2013;16:509-514.
154. Huppertz C, Bartels M, Jansen IE, Boomsma DI, Willemsen G, de Moor MH, et al. A twin-sibling study on the relationship between exercise attitudes and exercise behavior. Behav Genet. 2014;44:45-55.
155. Hutchens A, Lee RE. Parenting practices and children’s physical activity: An integrative review. J Sch Nurs. 2018;34:68-85.
156. Ickes S, Mahoney E, Roberts A, Dolan C. Parental involvement in a school-based child physical activity and nutrition program in southeastern United States: A qualitative analysis of parenting capacities. Health Promot Pract. 2016;17:285-296.
157. Inskip H, Baird J, Barker M, Briley AL, D’Angelo S, Grote V, et al. Influences on adherence to diet and physical activity recommendations in women and children: Insights from six European studies. Ann Nutr Metab. 2014;64:332-339.
158. Isgor Z, Powell LM, Wang Y. Multivariable analysis of the association between fathers’ and youths’ physical activity in the United States. BMC Public Health. 2013;13:1075.
159. Jacobi D, Caille A, Borys J-M, Lommez A, Couet C, Charles MA, et al. Parent-offspring correlations in pedometer-assessed physical activity. PLoS One. 2011;6:e29195.
160. Jaeschke L, Steinbrecher A, Luzak A, Puggina A, Aleksovska K, Buck C, et al. Socio-cultural determinants of physical activity across the life course: A “Determinants of diet and physical activity” (DEDIPAC) umbrella systematic literature review. Int J Behav Nutr Phys Act. 2017;14:173.
161. Jago R, Sebire SJ, Turner KM, Bentley GF, Goodred JK, Fox KR et al. Feasibility trial evaluation of a physical activity and screen-viewing course for parents of 6 to 8 year-old children: Teamplay. Int J Behav Nutr Phys Act. 2013;10:31.
162. Jago R, Davison KK, Brockman R, Page AS, Thompson JL, Fox KR. Parenting styles, parenting practices, and physical activity in 10- to 11-year olds. Prev Med (Baltim). 2011;52:44-47.
163. Jago R, Fox KR, Page AS, Brockman R, Thompson JL. Parent and child physical activity and sedentary time: Do active parents foster active children?. BMC Public Health. 2010;10:194.
164. Jago R, Sebire SJ, Wood L, Pool L, Zahra J, Thompson JL, et al. Associations between objectively assessed child and parental physical activity: A cross-sectional study of families with 5-6 year old children. BMC Public Health. 2014;14:655.
165. Jago R, Solomon-Moore E, Macdonald-Wallis C, Thompson JL, Lawlor DA, Sebire SJ. Association of parents’ and children’s physical activity and sedentary time in year 4 (8-9) and change between year 1 (5-6) and year 4: A longitudinal study. Int J Behav Nutr Phys Act. 2017;14:110.
166. Jago R, Wood L, Sebire SJ, Edwards MJ, Davies B, Banfield K, et al. School travel mode, parenting practices and physical activity among UK Year 5 and 6 children. BMC Public Health. 2014;14:370.
167. Jalali MS, Sharafi-Avarzaman Z, Rahmandad H, Ammerman AS. Social influence in childhood obesity interventions: A systematic review. Obes Rev. 2016;17:820-832.
168. Janicke DM, Lim CS, Perri MG, Bobroff LB, Mathews AE, Brumback BA, et al. The extension family lifestyle intervention project (E-FLIP for Kids): Design and methods. Contemp Clin Trials. 2011;32:50-58.
169. Janssen I, Ferrao T, King N. Individual, family, and neighborhood correlates of independent mobility among 7 to 11-year-olds. Prev Med Reports. 2016;3:98-102.
170. Jimenez-Pavon D, Fernandez-Alvira JM, Te Velde SJ, Brug J, Bere E, Jan N, et al. Associations of parental education and parental physical activity (PA) with children’s PA: The ENERGY cross-sectional study. Prev Med (Baltim). 2012;55:310-314.
171. Johnson RC, Allen TD. Examining the links between employed mothers’ work characteristics, physical activity, and child health. J Appl Psychol. 2013;98:148-157.
172. Kalakanis LE, Goldfield GS, Paluch RA, Epstein LH. Parental activity as a determinant of activity level and patterns of activity in obese children. Res Q Exerc Sport. 2001;72:202-209.
173. Kalil A, Ryan R, Corey M. Diverging destinies: Maternal education and the developmental gradient in time with children. Demography. 2012;49:1361-1383.
174. Karppanen A-K, Ahonen S-M, Tammelin T, Vanhala M, Korpelainen R. Physical activity and fitness in 8-year-old overweight and normal weight children and their parents. Int J Circumpolar Health. 2012;71:17621.
175. Kaseva K, Hintsa T, Lipsanen J, Pulkki-Raback L, Hintsanen M, Yang X, et al. Parental physical activity associates with offspring’s physical activity until middle age: A 30-year study. J Phys Act Health. 2017;14:520-531.
176. Kelishadi R, Azizi-Soleiman F. Controlling childhood obesity: A systematic review on strategies and challenges. J Res Med Sci. 2014;19:993-1008.
177. Kerpan S, Humbert L. Playing together: The physical activity beliefs and behaviors of urban Aboriginal youth. J Phys Act Health. 2015;12:1409-1413.
178. Kimiecik JC, Horn TS. Parental beliefs and children’s moderate-to-vigorous physical activity. Res Q Exerc Sport. 1998;69:163-175.
179. King AC, Parkinson KN, Adamson AJ, Murray L, Besson H, Reilly JJ, et al. Correlates of objectively measured physical activity and sedentary behaviour in English children. Eur J Public Health. 2011;21:424-431.
180. Kitzman-Ulrich H, Wilson DK, St George SM, Lawman H, Segal M, Fairchild A. The integration of a family systems approach for understanding youth obesity, physical activity, and dietary programs. Clin Child Fam Psychol Rev. 2010;13:231-253.
181. Knowlden AP, Sharma M, Cottrell RR, Wilson BRA, Johnson ML. Impact evaluation of enabling mothers to prevent pediatric obesity through web-based education and reciprocal determinism (EMPOWER) randomized control trial. Health Educ Behav. 2015;42:171-184.
182. Knowlden A, Sharma M. A feasibility and efficacy randomized controlled trial of an online preventative program for childhood obesity: Protocol for the EMPOWER intervention. JMIR Res Protoc. 2012;1:e5.
183. Knowlden A, Sharma M. One-year efficacy testing of enabling mothers to prevent pediatric obesity through web-based education and reciprocal determinism (EMPOWER) randomized control trial. Health Educ Behav. 2016;43:94-106.
184. Knuth AG, Silva ICM, van Hees VT, Cordeira K, Matijasevich A, Barros AJD, et al. Objectively-measured physical activity in children is influenced by social indicators rather than biological lifecourse factors: Evidence from a Brazilian cohort. Prev Med (Baltim). 2017;97:40-44.
185. Kubik MY, Lytle L, Fulkerson JA. Fruits, vegetables, and football: Findings from focus groups with alternative high school students regarding eating and physical activity. J Adolesc Health. 2005;36:494-500.
186. Kwon S, Janz KF, Letuchy EM, Burns TL, Levy SM. Parental characteristic patterns associated with maintaining healthy physical activity behavior during childhood and adolescence. Int J Behav Nutr Phys Act. 2016;13:58.
187. Laird Y, Fawkner S, Kelly P, McNamee L, Niven A. The role of social support on physical activity behaviour in adolescent girls: A systematic review and meta-analysis. Int J Behav Nutr Phys Act. 2016;13:79.
188. Laird Y, Fawkner S, Niven A. A grounded theory of how social support influences physical activity in adolescent girls. Int J Qual Stud Health Well-being. 2018;13:1435099.
189. Lam CB, McHale SM. Developmental patterns and parental correlates of youth leisure-time physical activity. J Fam Psychol. 2015;29:100-107.
190. Larsen H, Dinkel D, Warehime S, Berg K. The relationship between parental and child physical activity in a rural community. Fam Community Health. 2017;40:331-337.
191. Laukkanen A, Niemisto D, Finni T, Cantell M, Korhonen E, Saakslahti A. Correlates of physical activity parenting: The skilled kids study. Scand J Med Sci Sports. 2018;28:2691-2701.
192. Laukkanen A, Pesola AJ, Heikkinen R, Saakslahti AK, Finni T. Family-based cluster randomized controlled trial enhancing physical activity and motor competence in 4-7-year-old children. PLoS One. 2015;10:e0141124.
193. Lawman HG, Wilson DK. A review of family and environmental correlates of health behaviors in high-risk youth. Obesity. 2012;20:1142-1157.
194. Leary JM, Lilly CL, Dino G, Loprinzi PD, Cottrell L. Parental influences on 7-9 year olds’ physical activity: A conceptual model. Prev Med (Baltim). 2013;56:341-344.
195. Lee E-Y, Hesketh KD, Rhodes RE, Rinaldi CM, Spence JC, Carson V. Role of parental and environmental characteristics in toddlers’ physical activity and screen time: Bayesian analysis of structural equation models. Int J Behav Nutr Phys Act. 2018;15:17.
196. Lee SM, Nihiser A, Strouse D, Das B, Michael S, Huhman M. Correlates of children and parents being physically active together. J Phys Act Health. 2010;7:776-783.
197. Leoni E, Beltrami P, Poletti G, Baldi E, Sacchetti R, Garulli A, et al. [Survey on sports practice and physical activity of primary school children living in the area of Bologna Local Health Unit in relation with some individual and environmental variables]. Ann Ig. 2008;20:441-453.
198. Lindsay AC, Wasserman M, Munoz MA, Wallington SF, Greaney ML. Examining influences of parenting styles and practices on physical activity and sedentary behaviors in Latino children in the United States: Integrative review. JMIR public Heal Surveill. 2018;4:e14.
199. Liszewska N, Scholz U, Radtke T, Horodyska K, Liszewski M, Luszczynska A. Association between children’s physical activity and parental practices enhancing children’s physical activity: The moderating effects of children’s BMI z-score. Front Psychol. 2017;8:2359.
200. Liu GC, Wiehe SE, Aalsma MC. Associations between child and sibling levels of vigorous physical activity in low-income minority families. Int J Pediatr Adolesc Med. 2014;1:61-68.
201. Liu Y, Zhang Y, Chen S, Zhang J, Guo Z, Chen P. Associations between parental support for physical activity and moderate-to-vigorous physical activity among Chinese school children: A cross-sectional study. J Sport Heal Sci. 2017;6:410-415.
202. Long DE, Gaetke LM, Perry SD, Abel MG, Clasey JL. The assessment of physical activity and nutrition in home schooled versus public schooled children. Pediatr Exerc Sci. 2010;22:44-59.
203. Loprinzi P., Cardinal BJ, Loprinzi KL, Lee H. Parenting practices as mediators of child physical activity and weight status. Obes Facts. 2012;5:420-430.
204. Loucaides CA, Tsangaridou N. Associations between parental and friend social support and children’s physical activity and time spent outside playing. Int J Pediatr. 2017;2017:7582398.
205. Maatta S, Ray C, Roos E. Associations of parental influence and 10-11-year-old children’s physical activity: Are they mediated by children’s perceived competence and attraction to physical activity? Scand J Public Health. 2014;42:45-51.
206. Madsen KA, McCulloch CE, Crawford PB. Parent modeling: Perceptions of parents’ physical activity predict girls’ activity throughout adolescence. J Pediatr. 2009;154:278-283.
207. Maia J, Gomes TN, Tregouet D-A, Katzmarzyk PT. Familial resemblance of physical activity levels in the Portuguese population. J Sci Med Sport. 2014;17:381-386.
208. Maltby AM, Vanderloo LM, Tucker P. Exploring mothers’ influence on preschoolers’ physical activity and sedentary time: A cross sectional study. Matern Child Health J. 2018;22:978-985.
209. Martin-Matillas M, Ortega FB, Ruiz JR, Martinez-Gomez D, Marcos A, Moliner-Urdiales D, et al. Adolescent’s physical activity levels and relatives’ physical activity engagement and encouragement: The HELENA study. Eur J Public Health. 2011;21:705-712.
210. Martin LJ, Burke SM, Shapiro S, Carron AV, Irwin JD, Petrella R, et al. The use of group dynamics strategies to enhance cohesion in a lifestyle intervention program for obese children. BMC Public Health. 2009;9:277.
211. Martin M, Dollman J, Norton K, Robertson I. A decrease in the association between the physical activity patterns of Australian parents and their children; 1985-1997. J Sci Med Sport. 2005;8:71-76.
212. McGarvey E, Keller A, Forrester M, Williams E, Seward D, Suttle DE. Feasibility and benefits of a parent-focused preschool child obesity intervention. *Am J Public Health*. 2004;94:1490-1495.
213. McKee MD, Deen D, Maher S, Fletcher J, Fornari A, Blank AE. Implementation of a pilot primary care lifestyle change intervention for families of pre-school children: Lessons learned. Patient Educ Couns. 2010;79:299-305.
214. McMinn AM, van Sluijs EMF, Nightingale CM, Griffi SJ, Cook DG, Owen CG, et al. Family and home correlates of children’s physical activity in a multi-ethnic population: The cross-sectional child heart and health study in England (CHASE). Int J Behav Nutr Phys Act. 2011;8:11.
215. McMurray RG, Bradley CB, Harrell JS, Bernthal PR, Frauman AC, Bangdiwala SI. Parental influences on childhood fitness and activity patterns. Res Q Exerc Sport. 1993;64:249-255.
216. McMurray RG, Berry DC, Schwartz TA, Hall EG, Neal MN, Li S, et al. Relationships of physical activity and sedentary time in obese parent-child dyads: A cross-sectional study. BMC Public Health. 2016;16:124.
217. Mendonca G, Junior JC. Physical activity and social support in adolescents: Analysis of different types and sources of social support. J Sports Sci. 2015;33:1942-1951.
218. Messing S, Rutten A, Abu-Omar K, Ungerer-Rohrich U, Goodwin L, Burlacu I, et al. How can physical activity be promoted among children and adolescents? A systematic review of reviews across settings. Front Public Heal. 2019;7:55.
219. Monge-Rojas R, Garita-Arce C, Sanchez-Lopez M, Colon-Ramos U. Barriers to and suggestions for a healthful, active lifestyle as perceived by rural and urban Costa Rican adolescents. J Nutr Educ Behav. 2009;41:152-160.
220. Moore LL, Lombardi DA, White MJ, Campbell JL, Oliveria SA, Ellison RC. Influence of parents’ physical activity levels on activity levels of young children. J Pediatr. 1991;118:215-219.
221. Morgan PJ, Collins CE, Plotnikoff RC, Callister R, Burrows T, Fletcher R, et al. The “Healthy Dads, Healthy Kids” community randomized controlled trial: A community-based healthy lifestyle program for fathers and their children. Prev Med. 2014;61:90-99.
222. Mulhall P, Reis J, Begum S. Early adolescent participation in physical activity: Correlates with individual and family characteristics. J Phys Act Heal. 2011;8:244-252.
223. Mutz M, Albrecht P. Parents’ social status and children’s daily physical activity: The role of familial socialization and support. J Child Fam Stud. 2017;26:3026-3035.
224. Nader PR, Sallis JF, Patterson TL, Abramson IS, Rupp JW, Senn KL, et al. A family approach to cardiovascular risk reduction: Results from the San Diego family health project. Health Educ Q. 1989;16:229-244.
225. Neshteruk CD, Nezami BT, Nino-Tapias G, Davison KK, Ward DS. The influence of fathers on children’s physical activity: A review of the literature from 2009 to 2015. Prev Med. 2017;102:12-19.
226. Niermann CYN, Gerards SMPL, Kremers SPJ. Conceptualizing family influences on children’s energy balance-related behaviors: Levels of interacting family environmental subsystems (The LIFES framework). Int J Environ Res Public Health. 2018;15:e2714.
227. Niermann CYN, Spengler S, Gubbels JS. Physical activity, screen time, and dietary intake in families: A cluster-analysis with mother-father-child triads. Front Public Heal. 2018;6:276.
228. Nikolaidis PT. Familial aggregation and maximal heritability of exercise participation: A cross-sectional study in schoolchildren and their nuclear families. Sci Sport. 2011;26:157-165.
229. O'Connor TM, Jago R, Baranowski T. Engaging parents to increase youth physical activity a systematic review. Am J Prev Med. 2009;37:141-149.
230. O'Dwyer MV, Fairclough SJ, Knowles Z, Stratton G. Effect of a family focused active play intervention on sedentary time and physical activity in preschool children. Int J Behav Nutr Phys Act. 2012;9:117.
231. Olivares PR, Cossio-Bolanos MA, Gomez-Campos R, Almonacid-Fierro A, Garcia-Rubio J. Influence of parents and physical education teachers in adolescent physical activity. Int J Clin Health Psychol. 2015;15:113-120.
232. Oliver M, Schluter PJ, Schofield GM, Paterson J. Factors related to accelerometer-derived physical activity in Pacific children aged 6 years. Asia Pac J Public Health. 2011;23:44-56.
233. Oliver M, Schofield GM, Schluter PJ. Parent influences on preschoolers' objectively assessed physical activity. J Sci Med Sport. 2010;13:403-409.
234. Olvera N, Smith DW, Lee C, Liu J, Lee J, Kim JH, et al. Comparing high and low acculturated mothers and physical activity in Hispanic children. J Phys Act Health. 2011;8 Suppl 2:s206-213.
235. Ornelas IJ, Perreira KM, Ayala GX. Parental influences on adolescent physical activity: A longitudinal study. Int J Behav Nutr Phys Act. 2007;4:3.
236. Ostbye T, Malhotra R, Stroo M, Lovelady C, Brouwer R, Zucker N, et al. The effect of the home environment on physical activity and dietary intake in preschool children. Int J Obes (Lond). 2013;37:1314-1321.
237. Ostbye T, Mann CM, Vaughn AE, Namenek Brouwer RJ, Benjamin Neelson SE, Hales D, et al. The keys to healthy family child care homes intervention: Study design and rationale. Contemp Clin Trials. 2015;40:81-89.
238. Ostbye T, Krause KM, Stroo M, Lovelady CA, Evenson KR, Peterson BL, et al. Parent-focused change to prevent obesity in preschoolers: Results from the KAN-DO study. Prev Med. 2012;55:188-195.
239. Pahkala K, Heinonen OJ, Lagstrom H, Hakala P, Sillanmaki L, Simell O. Leisure-time physical activity of 13-year-old adolescents. Scand J Med Sci Sports. 2007;17:324-330.
240. Park H, Kim N. Predicting factors of physical activity in adolescents: A systematic review. Asian Nurs Res. 2008;2:113-128.
241. Parker KE, Salmon J, Villanueva K, Mavoa S, Veitch J, Brown HL, et al. Ecological correlates of activity-related behavior typologies among adolescents. BMC Public Health*.* 2019;19:1041.
242. Pate RR, Trost SG, Felton GM, Ward DS, Dowda M, Saunders R. Correlates of physical activity behavior in rural youth. Res Q Exerc Sport. 1997;68:241-248.
243. Pearson N, Timperio A, Salmon J, Crawford D, Biddle SJH. Family influences on children's physical activity and fruit and vegetable consumption. Int J Behav Nutr Phys Act. 2009;6:34.
244. Pereira S, Katzmarzyk PT, Gomes TN, Souza M, Chaves RN, Santos FK, et al. Resemblance in physical activity levels: The Portuguese sibling study on growth, fitness, lifestyle, and health. Am J Hum Biol. 2018;30.
245. Pereira S, Katzmarzyk PT, Gomes TN, Elston R, Maia J. How consistent are genetic factors in explaining leisure-time physical activity and sport participation? The Portuguese healthy families study. Twin Res Hum Genet. 2018;21:369-377.
246. Perusse L, Tremblay A, Leblanc C, Bouchard C. Genetic and environmental influences on level of habitual physical activity and exercise participation. Am J Epidemiol. 1989;129:1012-1022.
247. Price SM, McDivitt J, Weber D, Wolff LS, Massett HA, Fulton JE. Correlates of weight-bearing physical activity among adolescent girls: Results from a national survey of girls and their parents. J Phys Act Health. 2008;5:132-145.
248. Pugliese J, Tinsley B. Parental socialization of child and adolescent physical activity: A meta-analysis. J Fam Psychol. 2007;21:331-343.
249. Rachele JN, Cuddihy TF, Washington TL, McPhail SM. Adolescent's perceptions of parental influences on physical activity. Int J Adolesc Med Health. 2016;29.
250. Ramanathan S, Crocker PRE. The influence of family and culture on physical activity among female adolescents from the Indian diaspora. Qual Health Res. 2009;19:492-503.
251. Rebold MJ, Lepp A, Kobak MS, McDaniel J, Barkley JE. The effect of parental involvement on children's physical activity. J Pediatr. 2016;170:206-210.
252. Remmers T, Broeren SML, Renders CM, Hirasing RA, van Grieken A, Raat H. A longitudinal study of children's outside play using family environment and perceived physical environment as predictors. Int J Behav Nutr Phys Act. 2014;11:76.
253. Rhodes RE, Nwachukwu N, Quinlan A. Family exergaming: Correlates and preferences. Games Health J. 2018;7:188-196.
254. Richards R, Poulton R, Reeder AI, Williams S. Childhood and contemporaneous correlates of adolescent leisure time physical inactivity: A longitudinal study. J Adolesc Health. 2009;44:260-267.
255. Robbins LB, Stommel M, Hamel LM. Social support for physical activity of middle school students. Public Health Nurs. 2008;25:451-460.
256. Robertson W, Fleming J, Kamal A, Hamborg T, Khan KA, Griffiths F, et al. Randomised controlled trial evaluating the effectiveness and cost-effectiveness of 'Families for Health', a family-based childhood obesity treatment intervention delivered in a community setting for ages 6 to 11 years. Health Technol Assess. 2017;21:1-180.
257. Rodrigues D, Padez C, Machado-Rodrigues AM. Active parents, active children: The importance of parental organized physical activity in children's extracurricular sport participation. J Child Health Care. 2018;22:159-170.
258. Rodrigues D, Padez C, Machado-Rodrigues AM. Child participation in sports is influenced by patterns of lifestyle-related behaviors. Am J Hum Biol. 2018;30:e23142.
259. Rodriguez-Lopez C, Villa-Gonzalez E, Perez-Lopez IJ, Delgado-Fernandez M, Ruiz JR, Chillon P. [Family factors influence active commuting to school in Spanish children]. Nutr Hosp. 2013;28:756-763.
260. Romanella NE, Wakat DK, Loyd BH, Kelly LE. Physical activity and attitudes in lean and obese children and their mothers. Int J Obes. 1991;15:407-414.
261. Rossow I, Rise J. Concordance of parental and adolescent health behaviors. Soc Sci Med. 1994;38:1299-1305.
262. Rothman L, Macpherson AK, Ross T, Buliung RN. The decline in active school transportation (AST): A systematic review of the factors related to AST and changes in school transport over time in North America. Prev Med. 2018;111:314-322.
263. Rutkowski EM, Connelly CD. Self-efficacy and physical activity in adolescent and parent dyads. J Spec Pediatr Nurs. 2012;17:51-60.
264. Rutten C, Boen F, Seghers J. The relation between environmental factors and pedometer-determined physical activity in children: The mediating role of autonomous motivation. Pediatr Exerc Sci. 2013;25:273-287.
265. Saavedra JM, Escalante Y, Dominguez AM, Garcia-Hermoso A, Hernandez-Mocholi MA. Prediction of correlates of daily physical activity in Spanish children aged 8-9 years. Scand J Med Sci Sports*.* 2014;24:e213-219.
266. Sadler LS, Cowlin A. Moving into parenthood: A program for new adolescent mothers combining parent education with creative physical activity. J Spec Pediatr Nurs. 2003;8:62-70.
267. Sallis JF, Alcaraz JE, McKenzie TL, Hovell MF, Kolody B, Nader PR. Parental behavior in relation to physical activity and fitness in 9-year-old children. Am J Dis Child. 1992;146:1383-1388.
268. Sallis JF, Patterson TL, Buono MJ, Atkins CJ, Nader PR. Aggregation of physical activity habits in Mexican-American and Anglo families. J Behav Med. 1988;11:31-41.
269. Sallis JF, Patterson TL, McKenzie TL, Nader PR. Family variables and physical activity in preschool children. J Dev Behav Pediatr. 1988;9:57-61.
270. Sallis JF, Prochaska JJ, Taylor WC. A review of correlates of physical activity of children and adolescents. Med Sci Sports Exerc. 2000;32:963-975.
271. Sanchez A, Norman GJ, Sallis JF, Calfas KJ, Cella J, Patrick K. Patterns and correlates of physical activity and nutrition behaviors in adolescents. Am J Prev Med. 2007;32:124-130.
272. Sanchez-Zamorano LM, Solano-Gonzalez M, Macias-Morales N, Flores-Sanchez G, Galvan-Portillo MV, Lazcano-Ponce EC. Perception of parents' physical activity as a positive model on physical activity of adolescents. Prev Med. 2019;127:105797.
273. Satija A, Khandpur N, Satija S, Mathur Gaiha S, Prabhakaran D, Reddy KS, et al. Physical activity among adolescents in India: A qualitative study of barriers and enablers. Health Educ Behav. 2018;45:926-934.
274. Schoeppe S, Liersch S, Robl M, Krauth C, Walter U. Mothers and fathers both matter: The positive influence of parental physical activity modelling on children's leisure-time physical activity. Pediatr Exerc Sci. 2016;28:466-472.
275. Schoeppe S, Trost SG. Maternal and paternal support for physical activity and healthy eating in preschool children: A cross-sectional study. BMC Public Health. 2015;15:971.
276. Seabra AC, Maia J, Seabra AF, Welk G, Brustad R, Fonseca AM. Evaluating the youth physical activity promotion model among Portuguese elementary schoolchildren. J Phys Act Health. 2013;10:1159-1165.
277. Seabra AF, Mendonca DM, Thomis MA, Malina RM, Maia JA. Correlates of physical activity in Portuguese adolescents from 10 to 18 years. Scand J Med Sci Sports. 2011;21:318-323.
278. Seabra AC, Seabra AF, Mendonca DM, Brustad R, Maia JA, Fonseca AM, et al. Psychosocial correlates of physical activity in school children aged 8-10 years. Eur J Public Health. 2013;23:794-798.
279. Sebire SJ, Jago R, Wood L, Thompson JL, Zahra J, Lawlor DA. Examining a conceptual model of parental nurturance, parenting practices and physical activity among 5-6 year olds. Soc Sci Med. 2016;148:18-24.
280. Siegel SR, Malina RM, Reyes MEP, Barahona EEC, Cumming SP. Correlates of physical activity and inactivity in urban Mexican youth. Am J Hum Biol. 2011;23:686-692.
281. Sigmund E, Sigmundova D, Badura P, Voracova J. Relationship between Czech parent and child pedometer-assessed weekday and weekend physical activity and screen time. Cent Eur J Public Health. 2015;23 Suppl:s83-90.
282. Sigmundova D, Badura P, Sigmund E, Bucksch J. Weekday-weekend variations in mother-/father-child physical activity and screen time relationship: A cross-sectional study in a random sample of Czech families with 5- to 12-year-old children. Eur J Sport Sci*.* 2018;18:1158-1167.
283. Sijtsma A, Sauer PJJ, Corpeleijn E. Parental correlations of physical activity and body mass index in young children- the GECKO Drenthe cohort. Int J Behav Nutr Phys Act*.* 2015;12:132.
284. Silva DR, Fernandes RA, Ohara D, Collings PJ, Souza MF, Tomeleri CM, et al. Correlates of sports practice, occupational and leisure-time physical activity in Brazilian adolescents. Am J Hum Biol. 2016;28:112-117.
285. Simonen RL, Perusse L, Rankinen T, Rice T, Rao DC, Bouchard C. Familial aggregation of physical activity levels in the Quebec Family Study. Med Sci Sports Exerc. 2002;34:1137-1142.
286. Singh GK, Kogan MD, Siahpush M, Van Dyck PC. Independent and joint effects of socioeconomic, behavioral, and neighborhood characteristics on physical inactivity and activity levels among US children and adolescents. J Community Health. 2008;33:206-216.
287. Singh GK, Kogan MD, Siahpush M, van Dyck PC. Prevalence and correlates of state and regional disparities in vigorous physical activity levels among US children and adolescents. J Phys Act Health*.* 2009;6:73-87.
288. Skouteris H, Hill B, McCabe M, Swinburn B, Busija L. A parent-based intervention to promote healthy eating and active behaviours in pre-school children: Evaluation of the MEND 2-4 randomized controlled trial. Pediatr Obes. 2016;11:4-10.
289. Sleddens EFC, Kremers SPJ, Hughes SO, Cross MB, Thijs C, De Vries NK, et al. Physical activity parenting: A systematic review of questionnaires and their associations with child activity levels. Obes Rev. 2012;13:1015-1033.
290. Smith JD, Berkel C, Jordan N, Atkins DC, Narayanan SS, Gallo C, et al. An individually tailored family-centered intervention for pediatric obesity in primary care: Study protocol of a randomized type II hybrid effectiveness-implementation trial (raising healthy children study). Implement Sci. 2018;13:11.
291. Sobko T, Tse M, Kaplan M. A randomized controlled trial for families with preschool children - Promoting healthy eating and active playtime by connecting to nature. BMC Public Health. 2016;16:505.
292. Song M, Lee CS, Lyons KS, Stoyles S, Winters-Stone KM. Assessing the feasibility of parent participation in a commercial weight loss program to improve child body mass index and weight-related health behaviors. SAGE Open Med. 2018;6:2050312118801220.
293. Standiford A. The secret struggle of the active girl: A qualitative synthesis of interpersonal factors that influence physical activity in adolescent girls. Health Care Women Int. 2013;34:860-877.
294. Stearns JA, Rhodes R, Ball GD, Boule N, Veugelers PJ, Cutumisu N, et al. A cross-sectional study of the relationship between parents' and children's physical activity. BMC Public Health. 2016;16:1129.
295. Steenbock B, Pischke CR, Schonbach J, Pottgen S, Brand T. [The effectiveness of primary prevention interventions promoting physical activity and healthy eating in preschool children: A review of reviews]. Bundesgesundheitsblatt, Gesundheitsforschung, Gesundheitsschutz. 2015;58:609-619.
296. Straker LM, Smith KL, Fenner AA, Kerr DA, McManus A, Davis MC, et al. Rationale, design and methods for a staggered-entry, waitlist controlled clinical trial of the impact of a community-based, family-centred, multidisciplinary program focussed on activity, food and attitude habits (Curtin University's activity, food and attitudes program--CAFAP) among overweight adolescents. BMC Public Health. 2012;12:471.
297. Strutz E, Browning R, Smith S, Lohse B, Cunningham-Sabo L. Accelerometry-derived physical activity correlations between parents and their fourth-grade child are specific to time of day and activity level. J Phys Act Health. 2018;15:440-447.
298. Stucky-Ropp RC, DiLorenzo TM. Determinants of exercise in children. Prev Med. 1993;22:880-889.
299. Sung-Chan P, Sung YW, Zhao X, Brownson RC. Family-based models for childhood-obesity intervention: A systematic review of randomized controlled trials. Obes Rev. 2013;14:265-278.
300. Swaminathan S, Thomas T, Yusuf S, Vaz M. Clustering of diet, physical activity and overweight in parents and offspring in South India. Eur J Clin Nutr. 2013;67:128-134.
301. Tabak I. [The role of parents in supporting teenage children undertaking physical activity]. Pediatria Polska. 2016;91:26-34.
302. Tabak I, Jodkowska M, Oblacinska A, Mikiel-Kostyra K. [Can family meals protect adolescents from obesity?]. Medycyna wieku rozwojowego*.* 2012;16:313-321.
303. Tanaka C, Okuda M, Tanaka M, Inoue S, Tanaka S. Associations of physical activity and sedentary time in primary school children with their parental behaviors and supports. Int J Environ Res Public Health. 2018;15:e1995.
304. Tandon PS, Zhou C, Christakis DA. Frequency of parent-supervised outdoor play of US preschool-aged children. Arch Pediatr Adolesc Med. 2012;166:707-712.
305. Tate EB, Shah A, Jones M, Pentz MA, Liao Y, Dunton G. Toward a better understanding of the link between parent and child physical activity levels: The moderating role of parental encouragement. J Phys Act Health. 2015;12:1238-1244.
306. Tate NH, Davis JE, Yarandi HN. Sociocultural influences on weight-related behaviors in African American adolescents. West J Nurs Res. 2015;37:1531-1547.
307. Taverno Ross SE, Barone Gibbs B, Documet PI, Pate RR. ANDALE Pittsburgh: Results of a promotora-led, home-based intervention to promote a healthy weight in Latino preschool children. BMC Public Health. 2018;18:360.
308. Taylor A, Wilson C, Slater A, Mohr P. Parent- and child-reported parenting. Associations with child weight-related outcomes. Appetite. 2011;57:700-706.
309. Taylor NJ, Sahota P, Sargent J, Barber S, Loach J, Louch G, et al. Using intervention mapping to develop a culturally appropriate intervention to prevent childhood obesity: The HAPPY (Healthy and active parenting programme for early years) study. Int J Behav Nutr Phys Act*.* 2013;10:142.
310. te Velde SJ, ChinAPaw MJ, De Bourdeaudhuij I, Bere E, Maes L, Moreno L, et al. Parents and friends both matter: Simultaneous and interactive influences of parents and friends on European schoolchildren's energy balance-related behaviours - The ENERGY cross-sectional study. Int J Behav Nutr Phys Act*.* 2014;11:82.
311. Thompson VJ, Baranowski T, Cullen KW, Rittenberry L, Baranowski J, Taylor WC, et al. Influences on diet and physical activity among middle-class African American 8- to 10-year-old girls at risk of becoming obese. J Nutr Educ Behav. 2003;35:115-123.
312. Timperio AF, van Stralen MM, Brug J, Bere E, Chinapaw MJ, De Bourdeaudhuji I, et al. Direct and indirect associations between the family physical activity environment and sports participation among 10-12 year-old European children: Testing the EnRG framework in the ENERGY project. Int J Behav Nutr Phys Act*.* 2013;10:15.
313. Timperio A, Ball K, Salmon J, Roberts R, Giles-Corti B, Simmons D, et al. Personal, family, social, and environmental correlates of active commuting to school. Am J Prev Med. 2006;30:45-51.
314. Toftegaard-Stockel J, Nielsen GA, Ibsen B, Andersen LB. Parental, socio and cultural factors associated with adolescents' sports participation in four Danish municipalities. Scand J Med Sci Sports. 2011;21:606-611.
315. Tomayko EJ, Prince RJ, Cronin KA, Kim K, Parker T, Adams AK. The healthy children, strong families 2 (HCSF2) randomized controlled trial improved healthy behaviors in American Indian families with young children. Curr Dev Nutr. 2019;3:53-62.
316. Trost SG, Pate RR, Saunders R, Ward DS, Dowda M, Felton G. A prospective study of the determinants of physical activity in rural fifth-grade children. Prev Med. 1997;26:257-263.
317. Trost SG, Pate RR, Ward DS, Saunders R, Riner W. Determinants of physical activity in active and low-active, sixth grade African-American youth. J Sch Health. 1999;69:29-34.
318. Trost SG, Pate RR, Ward DS, Saunders R, Riner W. Correlates of objectively measured physical activity in preadolescent youth. Am J Prev Med. 1999;17:120-126.
319. Trost SG, Sirard JR, Dowda M, Pfeiffer KA, Pate RR. Physical activity in overweight and nonoverweight preschool children. Int J Obes Relat Metab Disord. 2003;27:834-839.
320. Trost SG, Sallis JF, Pate RR, Freedson PS, Taylor WC, Dowda M. Evaluating a model of parental influence on youth physical activity. Am J Prev Med. 2003;25:277-282.
321. Tuominen PPA, Husu P, Raitanen J, Kujala UM, Luoto RM. The effect of a movement-to-music video program on the objectively measured sedentary time and physical activity of preschool-aged children and their mothers: A randomized controlled trial. PloS one. 2017;12:e0183317.
322. Van Der Horst K, Paw MJCA, Twisk JWR, Van Mechelen W. A brief review on correlates of physical activity and sedentariness in youth. Med Sci Sports Exerc. 2007;39:1241-1250.
323. Van Hecke L, Deforche B, Van Dyck D, De Bourdeaudhuij I, Veitch J, Van Cauwenberg J. Social and physical environmental factors influencing adolescents' physical activity in urban public open spaces: A qualitative study using walk-along interviews. PloS one. 2016;11:e0155686.
324. van Sluijs EMF, Kriemler S, McMinn AM. The effect of community and family interventions on young people's physical activity levels: A review of reviews and updated systematic review. Br J Sports Med. 2011;45:914-922.
325. Vander Ploeg KA, Maximova K, Kuhle S, Simen-Kapeu A, Veugelers PJ. The importance of parental beliefs and support for physical activity and body weights of children: A population-based analysis. Can J Public Health. 2012;103:e277-281.
326. Vanwolleghem G, Van Dyck D, De Meester F, De Bourdeaudhuij I, Cardon G, Gheysen F. Which socio-ecological factors associate with a switch to or maintenance of active and passive transport during the transition from primary to secondary school? PloS one*.* 2016;11:e0156531.
327. Verloigne M, Cardon G, De Craemer M, D'Haese S, De Bourdeaudhuij I. Mediating effects of self-efficacy, benefits and barriers on the association between peer and parental factors and physical activity among adolescent girls with a lower educational level. PloS one. 2016;11:e0157216.
328. Verloigne M, Van Lippevelde W, Maes L, Brug J, De Bourdeaudhuij I. Family- and school-based correlates of energy balance-related behaviours in 10-12-year-old children: A systematic review within the ENERGY (EuropeaN energy balance research to prevent excessive weight gain among youth) project. Public Health Nutr. 2012;15:1380-1395.
329. Viitasalo A, Eloranta A-M, Lintu N, Vaisto J, Venalainen T, Kiiskinen S, et al. The effects of a 2-year individualized and family-based lifestyle intervention on physical activity, sedentary behavior and diet in children. Prev Med. 2016;87:81-88.
330. Vilhjalmsson R, Thorlindsson T. Factors related to physical activity: A study of adolescents. Soc Sci Med. 1998;47:665-675.
331. Vollmer RL, Adamsons K, Gorin A, Foster JS, Mobley AR. Investigating the relationship of body mass index, diet quality, and physical activity level between fathers and their preschool-aged children. J Acad Nutr Diet. 2015;115:919-926.
332. Vukovic D, Zivkovic M, Bjegovic V. Physical activity among school children. Srpski arhiv za celokupno lekarstvo. 1998;126:101-106.
333. Wagner A, Klein-Platat C, Arveiler D, Haan MC, Schlienger JL, Simon C. Parent-child physical activity relationships in 12-year old French students do not depend on family socioeconomic status. Diabetes Metab. 2004;30:359-366.
334. Wang M, Druker S, Gapinski MA, Gellar L, Schneider K, Osganian S, et al. The role of social support vs. modeling on adolescents' diet and physical activity: Findings from a school-based weight management trial. J Child Adolesc Behav*.* 2014;2.
335. Ward DS, Vaughn AE, Bangdiwala KI, Campbell M, Jones DJ, Panter AT, et al. Integrating a family-focused approach into child obesity prevention: Rationale and design for the my parenting SOS study randomized control trial. BMC Public Health. 2011;11:431.
336. Waters E, de Silva-Sanigorski A, Hall BJ, Brown T, Campbell KJ, Gao Y, et al. Interventions for preventing obesity in children. Cochrane Database Syst Rev. 2011:CD001871.
337. Webber KJ, Loescher LJ. A systematic review of parent role modeling of healthy eating and physical activity for their young African American children. J Spec Pediatr Nurs. 2013;18:173-188.
338. Welch JD, Ellis EM, Green PA, Ferrer RA. Social support, loneliness, eating, and activity among parent-adolescent dyads. J Behav Med. 2019;42:1015-1028.
339. Welk GJ, Wood K, Morss G. Parental influences on physical activity in children: An exploration of potential mechanisms. Pediatr Exerc Sci. 2003;15:19-33.
340. Wen LM, Rissel C, Xu H, Taki S, Smith W, Bedford K, et al. Linking two randomised controlled trials for Healthy Beginnings©: Optimising early obesity prevention programs for children under 3 years. BMC Public Health*.* 2019;19:739.
341. Whitehead SH, Biddle SJH, O'Donovan TM, Nevill ME. Social-psychological and physical environmental factors in groups differing by levels of physical activity: A study of Scottish adolescent girls. Pediatr Exerc Sci. 2006;18:226-239.
342. Wiley AR, Flood TL, Andrade FCD, Aradillas C, Cerda EM. Family and individual predictors of physical activity for older Mexican adolescents. J Adolesc Health. 2011;49:222-224.
343. Wilk P, Clark AF, Maltby A, Tucker P, Gilliland JA. Exploring the effect of parental influence on children's physical activity: The mediating role of children's perceptions of parental support. Prev Med. 2018;106:79-85.
344. Williams SL, Mummery WK. Links between adolescent physical activity, body mass index, and adolescent and parent characteristics. Health Educ Behav. 2011;38:510-520.
345. Wilson DK, Lawman HG, Segal M, Chappell S. Neighborhood and parental supports for physical activity in minority adolescents. Am J Prev Med. 2011;41:399-406.
346. Wright MS, Wilson DK, Griffin S, Evans A. A qualitative study of parental modeling and social support for physical activity in underserved adolescents. Health Educ Res. 2010;25:224-232.
347. Wrotniak BH, Zimmer N, Dingle K, Dingle A, Miller A, Knoell A, et al. Physical activity, health, and dietary patterns of middle school children. Pediatr Phys Ther. 2007;19:203-210.
348. Wu TY, Pender N, Noureddine S. Gender differences in the psychosocial and cognitive correlates of physical activity among Taiwanese adolescents: A structural equation modeling approach. Int J Behav Med. 2003;10:93-105.
349. Xu C, Quan M, Zhang H, Zhou C, Chen P. Impact of parents' physical activity on preschool children's physical activity: A cross-sectional study. PeerJ. 2018;6:e4405.
350. Xu H, Wen LM, Rissel C. Associations of parental influences with physical activity and screen time among young children: A systematic review. J Obes. 2015;2015:546925.
351. Xu H, Wen LM, Hardy LL, Rissel C. A 5-year longitudinal analysis of modifiable predictors for outdoor play and screen-time of 2- to 5-year-olds. Int J Behav Nutr Phys Act. 2016;13:96.
352. Yao CA, Rhodes RE. Parental correlates in child and adolescent physical activity: A meta-analysis. Int J Behav Nutr Phys Act. 2015;12:10.
353. Ylitalo KR, Bridges CN, Gutierrez M, Sharkey JR, Meyer MRU. Sibship, physical activity, and sedentary behavior: A longitudinal, observational study among Mexican-heritage sibling dyads. BMC Public Health. 2019;19:191.
354. Yoon HJ, Lee SA, Ju YJ, Nam JY, Park E-C. The relationship between physical activity level of parents and that of their adolescent children. J Phys Act Health. 2018;15:613-619.
355. Zach S, Netz Y. Like mother like child: Three generations' patterns of exercise behavior. Families, Systems and Health*.* 2007;25:419-434.
356. Zahl-Thanem T, Steinsbekk S, Wichstrom L. Predictors of physical activity in middle childhood. A fixed-effects regression approach. Front Public Health. 2018;6:305.
357. Zarychta K, Mullan B, Luszczynska A. It doesn't matter what they say, it matters how they behave: Parental influences and changes in body mass among overweight and obese adolescents. Appetite. 2016;96:47-55.
358. Zhang M, Quick V, Jin Y, Martin-Biggers J. Associations of mother's behaviors and home/neighborhood environments with preschool children's physical activity behaviors. Am J Health Promot. 2020;34:83-86.
359. Ziviani J, Scott J, Wadley D. Walking to school: Incidental physical activity in the daily occupations of Australian children. Occup Ther Int. 2004;11:1-11.

**Parental emotional support (n=233)**

(i.e., support/encouragement, watching/attending physical activities, talking about children’s physical activity)

1. Abdelghaffar E-A, Hicham EK, Siham B, Samira EF, Youness EA. Perspectives of adolescents, parents, and teachers on barriers and facilitators of physical activity among school-age adolescents: A qualitative analysis. Environ Health Prev Med. 2019;24:21.
2. Ahmed J, Mehraj V, Jeswani GK, ur Rehman S, Shah SM, Hamadeh R. Parental and school influences on physical activity levels of high school students in Hyderabad, Pakistan. J Ayub Med Coll Abbottabad. 2016;28:110-115.
3. Allison KR, Dwyer JJM, Goldenberg E, Fein A, Yoshida KK, Boutilier M. Male adolescents’ reasons for participating in physical activity, barriers to participation, and suggestions for increasing participation. Adolescence. 2005;40:155-170.
4. Appelhans BM, Li H. Organized sports and unstructured active play as physical activity sources in children from low-income, Chicago households. Pediatr Exerc Sci. 2016;28:381-387.
5. Ardestani M, Niknami S, Hidarnia A, Hajizadeh E. Predictors of physical activity among adolescent girl students based on the social cognitive theory. J Res Health Sci. 2015;15:223-227.
6. Badura P, Madarasova Geckova A, Sigmundova D, Sigmund E, van Dijk JP, Reijneveld SA. Do family environment factors play a role in adolescents’ involvement in organized activities?. J Adolesc. 2017;59:59-66.
7. Baskin ML, Dulin-Keita A, Thind H, Godsey E. Social and cultural environment factors influencing physical activity among african-american adolescents. J Adolesc Health. 2015;56:536-542.
8. Bauer KW, Neumark-Sztainer D, Hannan PJ, Fulkerson JA, Story M. Relationships between the family environment and school-based obesity prevention efforts: can school programs help adolescents who are most in need?. Health Educ Res. 2011;26:675-688.
9. Bauer KW, Berge JM, Neumark-Sztainer D. The importance of families to adolescents’ physical activity and dietary intake. Adolesc Med State Art Rev. 2011;22:611-613.
10. Bauer KW, Nelson MC, Boutelle KN, Neumark-Sztainer D. Parental influences on adolescents’ physical activity and sedentary behavior: Longitudinal findings from project EAT-II. Int J Behav Nutr Phys Act. 2008;5:12.
11. Bauer KW, Neumark-Sztainer D, Fulkerson JA, Hannan PJ, Story M. Familial correlates of adolescent girls’ physical activity, television use, dietary intake, weight, and body composition. Int J Behav Nutr Phys Act. 2011;8:25.
12. Bauer KW, Neumark-Sztainer D, Fulkerson JA, Story M. Adolescent girls’ weight-related family environments, Minnesota. Prev Chronic Dis. 2011;8:68.
13. Beets MW, Cardinal BJ, Alderman BL. Parental social support and the physical activity-related behaviors of youth: A review. Health Educ Behav. 2010;37:621-644.
14. Beets MW, Pitetti KH, Forlaw L. The role of self-efficacy and referent specific social support in promoting rural adolescent girls’ physical activity. Am J Health Behav. 2007;31:227-237.
15. Benes D, Dowling J, Crawford S, Hayman LL. Social and environmental influences on physical activity levels in Latina adolescents. Public Health Nurs. 2017;34:101-111.
16. Berge JM, Saelens BE. Familial influences on adolescents’ eating and physical activity behaviors. Adolesc Med State Art Rev. 2012;23:424-439.
17. Best K, Ball K, Zarnowiecki D, Stanley R, Dollman J. In search of consistent predictors of children’s physical activity. Int J Environ Res Public Health. 2017;14:e1258.
18. Biddle S, Goudas M. Analysis of children’s physical activity and its association with adult encouragement and social cognitive variables. J Sch Health. 1996;66:75-78.
19. Boutelle KN, Braden A, Douglas JM, Rhee KE, Strong D, Rock CL, et al. Design of the FRESH study: A randomized controlled trial of a parent-only and parent-child family-based treatment for childhood obesity. Contemp Clin Trials. 2015;45:364-370.
20. Bradley RH, McRitchie S, Houts RM, Nader P, O’Brien M, Network NECCR. Parenting and the decline of physical activity from age 9 to 15. Int J Behav Nutr Phys Act. 2011;8:33.
21. Brown HE, Corder K, Atkin AJ, van Sluijs EMF. Childhood predictors of adolescent behaviour: The prospective association of familial factors with meeting physical activity guidelines. Prev Med reports. 2017;6:221-227.
22. Brunet J, Sabiston CM, O’Loughlin J, Mathieu ME, Tremblay A, Barnett TA, et al. Perceived parental social support and moderate-to-vigorous physical activity in children at risk of obesity. Res Q Exerc Sport. 2014;85:198-207.
23. Camacho-Minano MJ, LaVoi NM, Barr-Anderson DJ. Interventions to promote physical activity among young and adolescent girls: A systematic review. Health Educ Res. 2011;26:1025-1049.
24. Campbell KJ, Hesketh KD, McNaughton SA, Ball K, McCallum Z, Lynch J, et al. The extended infant feeding, activity and nutrition trial (InFANT extend) program: A cluster-randomized controlled trial of an early intervention to prevent childhood obesity. BMC Public Health. 2016;16:166. 0
25. Campbell K, Hesketh K, Crawford D, Salmon J, Ball K, McCallum Z. The Infant feeding activity and nutrition trial (INFANT) an early intervention to prevent childhood obesity: cluster-randomised controlled trial. BMC Public Health. 2008;8:103.
26. Carson V. Cross-sectional and longitudinal associations between parental support and children’s physical activity in the early years. J Phys Act Health. 2016;13:611-616.
27. Carver A, Panter JR, Jones AP, van Sluijs EMF. Independent mobility on the journey to school: A joint cross-sectional and prospective exploration of social and physical environmental influences. J Transp Heal. 2014;1:25-32.
28. Chen H, Dai J. Does gender moderate the direct and indirect relationships between different sources of social support and adolescents’ physical activity?. J Phys Act Health. 2016;13:874-881.
29. Cheng LA, Mendonca G, Lucena JMS, Rech CR, Farias JCJ. Is the association between sociodemographic variables and physical activity levels in adolescents mediated by social support and self-efficacy?. J Pediatr (Rio J*)*. 2020;96:46-52.
30. Chiarlitti NA, Kolen AM. Parental Influences and the Relationship to their Children’s Physical Activity Levels. Int J Exerc Sci. 2017;10:205-212.
31. Cleland V, Timperio A, Salmon J, Hume C, Telford A, Crawford D. A longitudinal study of the family physical activity environment and physical activity among youth. Am J Health Promot. 2011;25:159-167.
32. Colabianchi N, Clennin MN, Dowda M, McIver KL, Dishman RK, Porter DE, et al. Moderating effect of the neighbourhood physical activity environment on the relation between psychosocial factors and physical activity in children: A longitudinal study. J Epidemiol Community Health. 2019;73:598-604.
33. Coleman L, Cox L, Roker D. Girls and young women’s participation in physical activity: Psychological and social influences. Health Educ Res. 2008;23:633-647.
34. Cottrell L, Zatezalo J, Bonasso A, Lattin J, Shawley S, Murphy E, et al. The relationship between children’s physical activity and family income in rural settings: A cross-sectional study. Prev Med Reports. 2015;2:99-104.
35. Courtney JB, Moss HE, Butki BD, Li K. Parent support, perceptions, and child attributes affect child activity. Am J Health Behav. 2019;43:311-325.
36. Crespo NC, Corder K, Marshall S, Norman GJ, Patrick K, Sallis JF, et al. An examination of multilevel factors that may explain gender differences in children’s physical activity. J Phys Act Health. 2013;10:982-992.
37. Czaplicki G, Laurencelle L, Deslandes R, Rivard MC, Trudeau F. Parental practices and youth physical activity and fruit and vegetables consumption. Sci Sport. 2013;28:36-45.
38. D’Haese S, Gheysen F, De Bourdeaudhuij I, Deforche B, Van Dyck D, Cardon G. The moderating effect of psychosocial factors in the relation between neighborhood walkability and children’s physical activity. Int J Behav Nutr Phys Act. 2016;13:128.
39. Davison KK, Jurkowski JM, Li K, Kranz S, Lawson HA. A childhood obesity intervention developed by families for families: Results from a pilot study. Int J Behav Nutr Phys Act. 2013;10:3.
40. Davison KK, Schmalz DL. Youth at risk of physical inactivity may benefit more from activity-related support than youth not at risk. Int J Behav Nutr Phys Act. 2006;3:5.
41. Davison KK, Nishi A, Kranz S, Wyckoff L, May JJ, Earle-Richardson GB, et al. Associations among social capital, parenting for active lifestyles, and youth physical activity in rural families living in upstate New York. Soc Sci Med. 2012;75:1488-1496.
42. Davison KK, Downs DS, Birch LL. Pathways linking perceived athletic competence and parental support at age 9 years to girls’ physical activity at age 11 years. Res Q Exerc Sport. 2006;77:23-31.
43. DiLorenzo TM, Stucky-Ropp RC, Vander Wal JS, Gotham HJ. Determinants of exercise among children: II. A longitudinal analysis. Prev Med (Baltim). 1998;27:470-477.
44. Dollman J, Lewis NR. The impact of socioeconomic position on sport participation among South Australian youth. J Sci Med Sport. 2010;13:318-322.
45. Donnelly R, Springer A. Parental social support, ethnicity, and energy balance-related behaviors in ethnically diverse, low-income, urban elementary schoolchildren. J Nutr Educ Behav. 2015;47:10-18.
46. Dowda M, Dishman RK, Pfeiffer KA, Pate RR. Family support for physical activity in girls from 8th to 12th grade in South Carolina. Prev Med (Baltim). 2007;44:153-159.
47. Dowda M, Pfeiffer KA, Brown WH, Mitchell JA, Byun W, Pate RR. Parental and environmental correlates of physical activity of children attending preschool. Arch Pediatr Adolesc Med. 2011;165:939-944.
48. Draper CE, Grobler L, Micklesfield LK, Norris SA. Impact of social norms and social support on diet, physical activity and sedentary behaviour of adolescents: A scoping review. Child Care Health Dev. 2015;41:654-667.
49. Duncan SC, Duncan TE, Strycker LA. Sources and types of social support in youth physical activity. Health Psychol. 2005;24:3-10.
50. Duncan SC, Strycker LA, Chaumeton NR. Personal, family, and peer correlates of general and sport physical activity among African American, Latino, and White Girls. J Health Dispar Res Pract. 2015;8:12-28.
51. Dunton GF, Liao Y, Almanza E, Jerrett M, Spruijt-Metz D, Chou CP, et al. Joint physical activity and sedentary behavior in parent-child pairs. Med Sci Sports Exerc. 2012;44:1473-1480.
52. Edwardson CL, Gorely T, Pearson N, Atkin A. Sources of activity-related social support and adolescents’ objectively measured after-school and weekend physical activity: Gender and age differences. J Phys Act Heal. 2013;10:1153-1158.
53. Eichinger M, Schneider S, De Bock F. Subjectively and objectively assessed social and physical environmental correlates of preschoolers’ accelerometer-based physical activity. Int J Behav Nutr Phys Act. 2017;14:153.
54. Eime RM, Harvey JT, Craike MJ, Symons CM, Payne WR. Family support and ease of access link socio-economic status and sports club membership in adolescent girls: A mediation study. Int J Behav Nutr Phys Act. 2013;10:50.
55. Epstein LH, Paluch RA, Kilanowski CK, Raynor HA. The effect of reinforcement or stimulus control to reduce sedentary behavior in the treatment of pediatric obesity. Health Psychol. 2004;23:371-380.
56. Epstein LH, Roemmich JN, Stein RI, Paluch RA, Kilanowski CK. The challenge of identifying behavioral alternatives to food: Clinic and field studies. Ann Behav Med. 2005;30:201-209.
57. Farias Junior JC de, Reis RS, Hallal PC. Physical activity, psychosocial and perceived environmental factors in adolescents from Northeast Brazil. Cad Saude Publica. 2014;30:941-951.
58. Ferrao T, Janssen I. Parental encouragement is positively associated with outdoor active play outside of school hours among 7-12 year olds. PeerJ. 2015;3:e1463.
59. Fisher A, Saxton J, Hill C, Webber L, Purslow L, Wardle J. Psychosocial correlates of objectively measured physical activity in children. Eur J Public Health. 2011;21:145-150.
60. Forthofer M, Dowda M, McIver K, Barr-Anderson DJ, Pate R. Associations between maternal support and physical activity among 5th grade students. Matern Child Health J. 2016;20:720-729.
61. Forthofer M, Dowda M, O’Neill JR, Addy CL, McDonald S, Reid L, et al. Effect of child gender and psychosocial factors on physical activity from fifth to sixth grade. J Phys Act Health. 2017;14:953-958.
62. Frenn M, Malin S, Villarruel AM, Slaikeu K, McCarthy S, Freeman J, et al. Determinants of physical activity and low-fat diet among low income African American and Hispanic middle school students. Public Health Nurs. 2005;22:89-97.
63. Garcia JM, Sirard JR, Larsen R, Bruening M, Wall M, Neumark-Sztainer D. Social and psychological factors associated with adolescent physical activity. J Phys Act Health. 2016;13:957-963.
64. Garnham-Lee KP, Falconer CL, Sherar LB, Taylor IM. Evidence of moderation effects in predicting active transport to school. J Public Health (Oxf). 2017;39:153-162.
65. Gebremariam MK, H Bergh I, F Andersen L, Ommundsen Y, Bjelland M, Lien N. Stability and change in potential correlates of physical activity and association with pubertal status among Norwegian children in the transition between childhood and adolescence. Int J Behav Nutr Phys Act. 2012;9:56.
66. George AM, da Silva JA, Bandeira A da S, Filho VCB, Rohr LE, Lopes ADS, et al. Association between socio-economic status and physical activity is mediated by social support in Brazilian students. J Sports Sci. 2019;37:500-506.
67. Gill M, Chan-Golston AM, Rice LN, Roth SE, Crespi CM, Cole BL, et al. Correlates of social support and its association with physical activity among young adolescents. Health Educ Behav. 2018;45:207-216.
68. Glozah FN, Pevalin DJ. Perceived social support and parental education as determinants of adolescents’ physical activity and eating behaviour: A cross-sectional survey. Int J Adolesc Med Health. 2015;27:253-259.
69. Graham DJ, Wall MM, Larson N, Neumark-Sztainer D. Multicontextual correlates of adolescent leisure-time physical activity. Am J Prev Med. 2014;46:605-616.
70. Grigsby-Toussaint DS, Chi SH, Fiese BH, Group SKPW. Where they live, how they play: neighborhood greenness and outdoor physical activity among preschoolers*. Int J Health Geogr.* 2011;10:66.
71. Gunter KB, Rice KR, Ward DS, Trost SG. Factors associated with physical activity in children attending family child care homes. Prev Med (Baltim). 2012;54:131-133.
72. Gustafson SL, Rhodes RE. Parental correlates of physical activity in children and early adolescents. Sports Med. 2006;36:79-97.
73. Haidar A, Ranjit N, Archer N, Hoelscher DM. Parental and peer social support is associated with healthier physical activity behaviors in adolescents: A cross-sectional analysis of Texas School Physical Activity and Nutrition (TX SPAN) data. BMC Public Health. 2019;19:640.
74. Hallmann K, Breuer C. The influence of socio-demographic indicators economic determinants and social recognition on sport participation in Germany. Eur J Sport Sci. 2014;14:324-31.
75. Heitzler CD, Martin SL, Duke J, Huhman M. Correlates of physical activity in a national sample of children aged 9-13 years. Prev Med (Baltim). 2006;42:254-260.
76. Henriksen PW, Ingholt L, Rasmussen M, Holstein BE. Physical activity among adolescents: The role of various kinds of parental support. Scand J Med Sci Sports. 2016;26:927-932.
77. Heredia NI, Ranjit N, Warren JL, Evans AE. Association of parental social support with energy balance-related behaviors in low-income and ethnically diverse children: A cross-sectional study. BMC Public Health. 2016;16:1182.
78. Hesketh KR, O’Malley C, Paes VM, Moore H3, Summerbell C, Ong KK, et al. Determinants of change in physical activity in children 0-6 years of age: A systematic review of quantitative literature. Sports Med. 2017;47:1349-1374.
79. Hohepa M, Scragg R, Schofield G, Kolt GS, Schaaf D. Social support for youth physical activity: Importance of siblings, parents, friends and school support across a segmented school day. Int J Behav Nutr Phys Act. 2007;4:54.
80. Hong J, Choo J, Kim H-J, Jae SY. Gender-specific correlates of sufficient physical activity among vulnerable children. Jpn J Nurs Sci. 2020;17:e12278.
81. Hosseini SV, Anoosheh M, Abbaszadeh A, Ehsani M. Qualitative Iranian study of parents’ roles in adolescent girls’ physical activity habit development. Nurs Health Sci. 2013;15:207-212.
82. Howe CA, Casapulla S, Shubrook JH, Lopez P, Grijalva M, Berryman DE. Regional variations in physical fitness and activity in healthy and overweight Ecuadorian adolescents. Child (Basel, Switzerland). 2018;5:104.
83. Huang WY, Wong SH, Salmon J. Correlates of physical activity and screen-based behaviors in Chinese children. J Sci Med Sport. 2013;16:509-514.
84. Huffman LE, Wilson DK, Van Horn ML, Pate RR. Associations between parenting factors, motivation, and physical activity in overweight African American adolescents. Ann Behav Med. 2018;52:93-105.
85. Huppertz C, Bartels M, Jansen IE, Boomsma DI, Willemsen G, de Moor MH, et al. A twin-sibling study on the relationship between exercise attitudes and exercise behavior. Behav Genet. 2014;44:45-55.
86. Izaki T, Swaine I. Physical activity and obesity among year 7 children in Kent, UK: Gender, social background and implications for school health promotion*. Int J Heal Promot Educ. 2017;55:189-204.
87. Jabeen I, Zuberi R, Nanji K. Physical activity levels and their correlates among secondary school adolescents in a township of Karachi, Pakistan. J Pak Med Assoc. 2018;68:737-743.
88. Jaeschke L, Steinbrecher A, Luzak A, Puggina A, Aleksovska K, Buck C, et al. Socio-cultural determinants of physical activity across the life course: A “Determinants of diet and physical activity” (DEDIPAC) umbrella systematic literature review. Int J Behav Nutr Phys Act. 2017;14:173.
89. Jonsson L, Berg C, Larsson C, Korp P, Lindgren E-C. Facilitators of physical activity: voices of adolescents in a disadvantaged community. Int J Environ Res Public Health. 2017;14:839 .
90. Kader M, Sundblom E, Elinder LS. Effectiveness of universal parental support interventions addressing children’s dietary habits, physical activity and bodyweight: A systematic review. Prev Med (Baltim). 2015;77:52-67.
91. Kelishadi R, Ghatrehsamani S, Hosseini M, Mirmoghtadaee P, Mansouri S, Poursafa P. Barriers to physical activity in a population-based sample of children and adolescents in Isfahan, Iran. Int J Prev Med. 2010;1:131-137.
92. Keresztes N, Piko BF, Pluhar ZF, Page RM. Social influences in sports activity among adolescents. J R Soc Promot Health. 2008;128:21-25.
93. King KA, Tergerson JL, Wilson BR. Effect of social support on adolescents’ perceptions of and engagement in physical activity. J Phys Act Health. 2008;5:374-384.
94. Kirby J, Levin KA, Inchley J. Parental and peer influences on physical activity among scottish adolescents: A longitudinal study. *J Phys Act Heal*. 2011;8:785-793.
95. Kitzman-Ulrich H, Wilson DK, Van Horn ML, Lawman HG. Relationship of body mass index and psychosocial factors on physical activity in underserved adolescent boys and girls. Health Psychol. 2010;29:506-513.
96. Knowles A-M, Niven A, Fawkner S. A qualitative examination of factors related to the decrease in physical activity behavior in adolescent girls during the transition from primary to secondary school. J Phys Act Health. 2011;8:1084-1091.
97. Kubik MY, Lytle L, Fulkerson JA. Fruits, vegetables, and football: findings from focus groups with alternative high school students regarding eating and physical activity. J Adolesc Health. 2005;36:494-500.
98. Laird Y, Fawkner S, Kelly P, McNamee L, Niven A. The role of social support on physical activity behaviour in adolescent girls: A systematic review and meta-analysis. Int J Behav Nutr Phys Act. 2016;13:79.
99. Laird Y, Fawkner S, Niven A. A grounded theory of how social support influences physical activity in adolescent girls. Int J Qual Stud Health Well-being. 2018;13:1435099.
100. Langer SL, Crain AL, Senso MM, Levy RL, Sherwood NE. Predicting child physical activity and screen time: parental support for physical activity and general parenting styles. J Pediatr Psychol. 2014;39:633-642.
101. Larsen H, Dinkel D, Warehime S, Berg K. The relationship between parental and child physical activity in a rural community. Fam Community Health. 2017;40:331-337.
102. Lau EY, Faulkner G, Qian W, Leatherdale ST. Longitudinal associations of parental and peer influences with physical activity during adolescence: findings from the COMPASS study. Heal Promot chronic Dis Prev Canada Res policy Pract. 2016;36:235-242.
103. Lau EY, Barr-Anderson DJ, Dowda M, Forthofer M, Saunders RP, Pate RR. Associations between home environment and after-school physical activity and sedentary time among 6th grade children. Pediatr Exerc Sci. 2015;27:226-233.
104. Laukkanen A, Niemisto D, Finni T, Cantell M, Korhonen E, Saakslahti A. Correlates of physical activity parenting: The Skilled Kids study. Scand J Med Sci Sports. 2018;28:2691-2701.
105. Laukkanen A, Pesola AJ, Finni T, Saakslahti A. Parental support and objectively measured physical activity in children: A yearlong cluster-randomized controlled efficacy trial. Res Q Exerc Sport. 2017;88:293-306.
106. Lawman HG, Wilson DK. Associations of social and environmental supports with sedentary behavior, light and moderate-to-vigorous physical activity in obese underserved adolescents. Int J Behav Nutr Phys Act. 2014;11:92.
107. Lawman HG, Wilson DK, Van Horn ML, Zarrett N. The role of motivation in understanding social contextual influences on physical activity in underserved adolescents in the ACT Trial: A cross-sectional study. Child Obes. 2012;8:542-550.
108. Lee CG, Park S, Yoo S. The longitudinal effect of parental support during adolescence on the trajectory of sport participation from adolescence through young adulthood. J Sport Heal Sci. 2018;7:70-76.
109. Lee KS, Loprinzi PD, Trost SG. Determinants of physical activity in Singaporean adolescents. Int J Behav Med. 2010;17:279-286.
110. Leslie E, Kremer P, Toumbourou JW, Williams JW. Gender differences in personal, social and environmental influences on active travel to and from school for Australian adolescents. J Sci Med Sport. 2010;13:597-601.
111. Li K, Haynie D, Lipsky L, Iannotti RJ, Pratt C, Simons-Morton B. Changes in moderate-to-vigorous physical activity among older adolescents. Pediatrics. 2016;138:e20161372.
112. Licence K. Promoting and protecting the health of children and young people. Child Care Health Dev. 2004;30:623-635.
113. Lindsay AC, Wasserman M, Munoz MA, Wallington SF, Greaney ML. Examining influences of parenting styles and practices on physical activity and sedentary behaviors in Latino children in the United States: Integrative review. JMIR public Heal Surveill. 2018;4:e14.
114. Ling J, Robbins LB, McCarthy VL, Speck BJ. Psychosocial determinants of physical activity in children attending afterschool programs: A path analysis. Nurs Res. 2015;64:190-199.
115. Liszewska N, Scholz U, Radtke T, Horodyska K, Liszewski M, Luszczynska A. Association between children’s physical activity and parental practices enhancing children’s physical activity: The moderating effects of children’s BMI z-score. Front Psychol. 2017;8:2359.
116. Liu Y, Zhang Y, Chen S, Zhang J, Guo Z, Chen P. Associations between parental support for physical activity and moderate-to-vigorous physical activity among Chinese school children: A cross-sectional study. J Sport Heal Sci. 2017;6:410-415.
117. Long DE, Gaetke LM, Perry SD, Abel MG, Clasey JL. The assessment of physical activity and nutrition in home schooled versus public schooled children. Pediatr Exerc Sci. 2010;22:44-59.
118. Lopez N V, Yang C-H, Belcher BR, Margolin G, Dunton GF. Within-subject associations of maternal physical activity parenting practices on children’s objectively measured moderate-to-vigorous physical activity. J Pediatr Psychol. 2019;44:300-310.
119. Loprinzi P., Cardinal BJ, Loprinzi KL, Lee H. Parenting practices as mediators of child physical activity and weight status. Obes Facts. 2012;5:420-430.
120. Loprinzi PD, Trost SG. Parental influences on physical activity behavior in preschool children. Prev Med (Baltim). 2010;50:129-133.
121. Loucaides CA, Tsangaridou N. Associations between parental and friend social support and children’s physical activity and time spent outside playing. Int J Pediatr. 2017;2017:7582398.
122. Lowry R, Lee SM, Fulton JE, Demissie Z, Kann L. Obesity and other correlates of physical activity and sedentary behaviors among US high school students. J Obes. 2013;2013:276318.
123. Maatta S, Ray C, Roos E. Associations of parental influence and 10-11-year-old children’s physical activity: Are they mediated by children’s perceived competence and attraction to physical activity?. Scand J Public Health. 2014;42:45-51.
124. Mah SK, Nettlefold L, Macdonald HM, Winters M, Race D, Voss C, et al. Does parental support influence children’s active school travel?. Prev Med reports. 2017;6:346-351.
125. Maltby AM, Vanderloo LM, Tucker P. Exploring mothers’ influence on preschoolers’ physical activity and sedentary time: A cross sectional study. Matern Child Health J. 2018;22:978-985.
126. Martin-Matillas M, Ortega FB, Ruiz JR, Martínez-Gómez D, Marcos A, Moliner-Urdiales D, et al. Adolescent’s physical activity levels and relatives’ physical activity engagement and encouragement: The HELENA study. Eur J Public Health. 2011;21:705-712.
127. Martin JJ, McCaughtry N. Predicting physical activity in inner-city Hispanic American children. Hisp Heal Care Int. 2008;6:150-158.
128. Marzi I, Demetriou Y, Reimers AK. Social and physical environmental correlates of independent mobility in children: A systematic review taking sex/gender differences into account. Int J Health Geogr. 2018;17:24.
129. McDonald S, Dowda M, Colabianchi N, Porter D, Dishman RK, Pate RR. Perceptions of the neighborhood environment and children’s afterschool moderate-to-vigorous physical activity. Pediatr Exerc Sci. 2015;27:243-251.
130. McGuire MT, Hannan PJ, Neumark-Sztainer D, Cossrow NHF, Story M. Parental correlates of physical activity in a racially/ethnically diverse adolescent sample. J Adolesc Health. 2002;30:253-261.
131. McKenzie TL, Baquero B, Crespo NC, Arredondo EM, Campbell NR, Elder JP. Environmental correlates of physical activity in Mexican American children at home. J Phys Act Health. 2008;5:579-591.
132. McMinn AM, Griffin SJ, Jones AP, van Sluijs EMF. Family and home influences on children’s after-school and weekend physical activity. Eur J Public Health. 2013;23:805-810.
133. Mendonca G, Cheng LA, Melo EN, de Farias Junior JC. Physical activity and social support in adolescents: A systematic review. Health Educ Res. 2014;29:822-839.
134. Monge-Rojas R, Garita-Arce C, Sanchez-Lopez M, Colon-Ramos U. Barriers to and suggestions for a healthful, active lifestyle as perceived by rural and urban Costa Rican adolescents. J Nutr Educ Behav. 2009;41:152-160.
135. Monge-Rojas R, Nunez HP, Garita C, Chen-Mok M. Psychosocial aspects of Costa Rican adolescents’ eating and physical activity patterns. J Adolesc Health. 2002;31:212-219.
136. Moore LL, Lombardi DA, White MJ, Campbell JL, Oliveria SA, Ellison RC. Influence of parents’ physical activity levels on activity levels of young children. J Pediatr. 1991;118:215-219.
137. Morrissey JL, Janz KF, Letuchy EM, Francis SL, Levy SM. The effect of family and friend support on physical activity through adolescence: A longitudinal study. Int J Behav Nutr Phys Act. 2015;12:103.
138. Morrissey JL, Wenthe PJ, Letuchy EM, Levy SM, Janz KF. Specific types of family support and adolescent non-school physical activity levels. Pediatr Exerc Sci. 2012;24:333-346.
139. Motl RW, Dishman RK, Saunders RP, Dowda M, Pate RR. Perceptions of physical and social environment variables and self-efficacy as correlates of self-reported physical activity among adolescent girls. J Pediatr Psychol. 2007;32:6-12.
140. Mutz M, Albrecht P. Parents’ social status and children’s daily physical activity: The role of familial socialization and support. J Child Fam Stud. 2017;26:3026-3035.
141. Neumark-Sztainer D, Story M, Hannan PJ, Tharp T, Rex J. Factors associated with changes in physical activity: A cohort study of inactive adolescent girls. Arch Pediatr Adolesc Med. 2003;157:803-810.
142. Nicksic NE, Salahuddin M, Butte NF, Hoelscher DM. Associations between parent-perceived neighborhood safety and encouragement and child outdoor physical activity among low-income children. J Phys Act Health. 2018;15:317-324.
143. Niermann CYN, Gerards SMPL, Kremers SPJ. Conceptualizing family influences on children’s energy balance-related behaviors: Levels of interacting family environmental subsystems (the LIFES framework). Int J Environ Res Public Health. 2018;15:2714.
144. Noonan RJ, Boddy LM, Knowles ZR, Fairclough SJ. Predisposing, reinforcing and enabling factors for physical activity in boys and girls from socially disadvantaged communities. Health Educ J. 2019;78:149-162.
145. O'Dea JA. Why do kids eat healthful food? Perceived benefits of and barriers to healthful eating and physical activity among children and adolescents. J Am Diet Assoc. 2003;103:497-501.
146. O'Loughlin J, Paradis G, Kishchuk N, Barnett T, Renaud L. Prevalence and correlates of physical activity behaviors among elementary schoolchildren in multiethnic, low income, inner-city neighborhoods in Montreal, Canada. Ann Epidemiol*.* 1999;9:397-407.
147. Odusoga O, Sholeye O. Addressing risk factors for non-communicable diseases in adolescents: A study of physical activity among male students in Sagamu, Ogun State, Nigeria (P04-183-19). Current Developments in Nutrition. 2019;3.
148. Olivares PR, Cossio-Bolanos MA, Gomez-Campos R, Almonacid-Fierro A, Garcia-Rubio J. Influence of parents and physical education teachers in adolescent physical activity. Int J Clin Health Psychol. 2015;15:113-120.
149. Panter JR, Jones AP, van Sluijs EMF, Griffin SJ. Attitudes, social support and environmental perceptions as predictors of active commuting behaviour in school children. J Epidemiol Community Health. 2010;64:41-48.
150. Park H, Kim N. Predicting factors of physical activity in adolescents: A systematic review. Asian Nurs Res. 2008;2:113-128.
151. Parker KE, Salmon J, Villanueva K, Mavoa S, Veitch J, Brown HL, et al. Ecological correlates of activity-related behavior typologies among adolescents. BMC Public Health*.* 2019;19:1041.
152. Pate RR, Dowda M, Dishman RK, Colabianchi N, Saunders RP, McIver KL. Change in children's physical activity: Predictors in the transition from elementary to middle school. Am J Prev Med. 2019;56:e65-73.
153. Patnode CD, Lytle LA, Erickson DJ, Sirard JR, Barr-Anderson D, Story M. The relative influence of demographic, individual, social, and environmental factors on physical activity among boys and girls. Int J Behav Nutr Phys Act. 2010;7:79.
154. Pearson N, Timperio A, Salmon J, Crawford D, Biddle SJH. Family influences on children's physical activity and fruit and vegetable consumption. Int J Behav Nutr Phys Act. 2009;6:34.
155. Peeters C, Marchand H, Tulloch H, Sigal RJ, Goldfield GS, Hadjiyannakis S, et al. Perceived facilitators, barriers, and changes in a randomized exercise trial for obese youth: A qualitative inquiry. J Phys Act Health. 2012;9:650-660.
156. Pereira S, Katzmarzyk PT, Gomes TN, Souza M, Chaves RN, Santos FK, et al. Resemblance in physical activity levels: The Portuguese sibling study on growth, fitness, lifestyle, and health. Am J Hum Biol. 2018;30.
157. Peterson MS, Lawman HG, Wilson DK, Fairchild A, Van Horn ML. The association of self-efficacy and parent social support on physical activity in male and female adolescents. Health Psychol. 2013;32:666-674.
158. Pinquart M. Associations of general parenting and parent-child relationship with pediatric obesity: A meta-analysis. J Pediatr Psychol. 2014;39:381-393.
159. Prado CV, Lima AV, Fermino RC, Anez CRR, Reis RS. Social support and physical activity in adolescents from public schools: The importance of family and friends. Cad Saude Publica. 2014;30:827-838.
160. Prochaska JJ, Rodgers MW, Sallis JF. Association of parent and peer support with adolescent physical activity. Res Q Exerc Sport. 2002;73:206-210.
161. Pugliese J, Tinsley B. Parental socialization of child and adolescent physical activity: A meta-analysis. J Fam Psychol. 2007;21:331-343.
162. Rachele JN, Cuddihy TF, Washington TL, McPhail SM. Adolescent's perceptions of parental influences on physical activity. Int J Adolesc Med Health. 2016;29.
163. Raudsepp L. The relationship between socio-economic status, parental support and adolescent physical activity. Acta Paediatr. 2006;95:93-98.
164. Rebold MJ, Lepp A, Kobak MS, McDaniel J, Barkley JE. The effect of parental involvement on children's physical activity. J Pediatr. 2016;170:206-210.
165. Rhodes RE, Berry T, Craig CL, Faulkner G, Latimer-Cheung A, Spence JC, et al. Understanding parental support of child physical activity behavior. Am J Health Behav*.* 2013;37:469-477.
166. Rhodes RE, Spence JC, Berry T, Deshpande S, Faulkner G, Latimer-Cheung AE, et al. Predicting changes across 12 months in three types of parental support behaviors and mothers' perceptions of child physical activity. Ann Behav Med. 2015;49:853-864.
167. Ries AV, Voorhees CC, Gittelsohn J, Roche KM, Astone NM. Adolescents' perceptions of environmental influences on physical activity. Am J Health Behav. 2008;32:26-39.
168. Robbins LB, Ling J, Dalimonte-Merckling DM, Sharma DB, Bakhoya M, Pfeiffer KA. Sources and types of social support for physical activity perceived by fifth to eighth grade girls. J Nurs Scholarsh*.* 2018;50:172-180.
169. Robbins LB, Stommel M, Hamel LM. Social support for physical activity of middle school students. Public Health Nurs. 2008;25:451-460.
170. Robertson W, Fleming J, Kamal A, Hamborg T, Khan KA, Griffiths F, et al. Randomised controlled trial evaluating the effectiveness and cost-effectiveness of 'Families for Health', a family-based childhood obesity treatment intervention delivered in a community setting for ages 6 to 11 years. Health Technol Assess*.* 2017;21:1-180.
171. Robertson-Wilson JE, Leatherdale ST, Wong SL. Social-ecological correlates of active commuting to school among high school students. J Adolesc Health. 2008;42:486-495.
172. Roesch SC, Norman GJ, Adams MA, Kerr J, Sallis JF, Ryan S, et al. Latent growth curve modeling of adolescent physical activity: Testing parallel process and mediation models. J Health Psychol. 2009;14:313-325.
173. Rupp K, Taverno Ross SE, Gary-Webb TL, Akiva T, Jakicic JM. Household support for physical activity in adolescent girls living in primarily low socioeconomic status neighborhoods. Int J Exerc Sci. 2019;12:811-824.
174. Sallis JF, Alcaraz JE, McKenzie TL, Hovell MF, Kolody B, Nader PR. Parental behavior in relation to physical activity and fitness in 9-year-old children. Am J Dis Child. 1992;146:1383-1388.
175. Sallis JF, Prochaska JJ, Taylor WC. A review of correlates of physical activity of children and adolescents. Med Sci Sports Exerc*.* 2000;32:963-975.
176. Sallis JF, Prochaska JJ, Taylor WC, Hill JO, Geraci JC. Correlates of physical activity in a national sample of girls and boys in grades 4 through 12. Health Psychol. 1999;18:410-415.
177. Saunders RP, Motl RW, Dowda M, Dishman RK, Pate RR. Comparison of social variables for understanding physical activity in adolescent girls. Am J Health Behav. 2004;28:426-436.
178. Schaefer SE, Gomez-Camacho R, Martinez L, Sadeghi B, German JB, de la Torre A. Social and environmental determinants of child physical activity in a rural Mexican-origin community. J Community Health. 2016;41:409-416.
179. Schoeppe S, Trost SG. Maternal and paternal support for physical activity and healthy eating in preschool children: A cross-sectional study. BMC Public Health. 2015;15:971.
180. Sebire SJ, Jago R, Wood L, Thompson JL, Zahra J, Lawlor DA. Examining a conceptual model of parental nurturance, parenting practices and physical activity among 5-6 year olds. Soc Sci Med. 2016;148:18-24.
181. Sharma B, Chavez RC, Nam EW. Prevalence and correlates of insufficient physical activity in school adolescents in Peru. Rev Saude Publica. 2018;52:51.
182. Sharma SV, Hoelscher DM, Kelder SH, Diamond PM, Day RS, Hergenroeder AC. A path analysis to identify the psychosocial factors influencing physical activity and bone health in middle-school girls. J Phys Act Health. 2009;6:606-616.
183. Shokrvash B, Majlessi F, Montazeri A, Nedjat S, Rahimi A, Djazayeri A, et al. Correlates of physical activity in adolescence: A study from a developing country. Glob Health Action. 2013;6:20327.
184. Shuval K, Weissblueth E, Brezis M, Araida A, Dipietro L. Individual and socio-ecological correlates of physical activity among Arab and Jewish college students in Israel. J Phys Act Health*.* 2009;6:306-314.
185. Siceloff ER, Wilson DK, Van Horn L. A longitudinal study of the effects of instrumental and emotional social support on physical activity in underserved adolescents in the ACT trial. Ann Behav Med. 2014;48:71-79.
186. Silva KS, Pizarro AN, Garcia LMT, Mota J, Santos MP. Which social support and psychological factors are associated to active commuting to school? Prev Med. 2014;63:20-23.
187. Silva P, Lott R, Mota J, Welk G. Direct and indirect effects of social support on youth physical activity behavior. Pediatr Exerc Sci. 2014;26:86-94.
188. Simons D, Rosenberg M, Salmon J, Knuiman M, Granich J, Deforche B, et al. Psychosocial moderators of associations between life events and changes in physical activity after leaving high school. Prev Med. 2015;72:30-33.
189. Sleddens EFC, Kremers SPJ, Hughes SO, Cross MB, Thijs C, De Vries NK, et al. Physical activity parenting: A systematic review of questionnaires and their associations with child activity levels. Obes Rev. 2012;13:1015-1033.
190. Spurr S, Bally J, Trinder K. Predictors of physical activity in positive deviant adolescents. J Pediatr Nurs. 2016;31:311-318.
191. Standiford A. The secret struggle of the active girl: A qualitative synthesis of interpersonal factors that influence physical activity in adolescent girls. Health Care Women Int. 2013;34:860-877.
192. Stanley RM, Boshoff K, Dollman J. A qualitative exploration of the "critical window": Factors affecting Australian children's after-school physical activity. J Phys Act Health. 2013;10:33-41.
193. Sterdt E, Liersch S, Walter U. Correlates of physical activity of children and adolescents: A systematic review of reviews. Health Educ J. 2014;73:72-89.
194. Stucky-Ropp RC, DiLorenzo TM. Determinants of exercise in children. Prev Med. 1993;22:880-889.
195. Tabak I. The role of parents in supporting teenage children undertaking physical activity. Pediatria Polska. 2016;91:26-34.
196. Tandon P, Grow HM, Couch S, Glanz K, Sallis JF, Frank LD, et al. Physical and social home environment in relation to children's overall and home-based physical activity and sedentary time. Prev Med. 2014;66:39-44.
197. Tate EB, Shah A, Jones M, Pentz MA, Liao Y, Dunton G. Toward a better understanding of the link between parent and child physical activity levels: The moderating role of parental encouragement. J Phys Act Health. 2015;12:1238-1244.
198. Taylor WC, Sallis JF, Dowda M, Freedson PS, Eason K, Pate RR. Activity patterns and correlates among youth: Differences by weight status. Pediatr Exerc Sci. 2002;14:418-431.
199. Taymoori P, Rhodes RE, Berry TR. Application of a social cognitive model in explaining physical activity in Iranian female adolescents. Health Educ Res. 2010;25:257-267.
200. Thompson VJ, Baranowski T, Cullen KW, Rittenberry L, Baranowski J, Taylor WC, et al. Influences on diet and physical activity among middle-class African American 8- to 10-year-old girls at risk of becoming obese. J Nutr Educ Behav. 2003;35:115-123.
201. Timperio AF, van Stralen MM, Brug J, Bere E, Chinapaw MJ, De Bourdeaudhuij I, et al. Direct and indirect associations between the family physical activity environment and sports participation among 10-12 year-old European children: Testing the EnRG framework in the ENERGY project. Int J Behav Nutr Phys Act*.* 2013;10:15.
202. Trost SG, Sirard JR, Dowda M, Pfeiffer KA, Pate RR. Physical activity in overweight and nonoverweight preschool children. Int J Obes Relat Metab Disord. 2003;27:834-839.
203. Trost SG, Sallis JF, Pate RR, Freedson PS, Taylor WC, Dowda M. Evaluating a model of parental influence on youth physical activity. Am J Prev Med. 2003;25:277-282.
204. Umstattd Meyer MR, Walsh SM, Sharkey JR, Morgan GB, Nalty CC. Physical and social environmental characteristics of physical activity for Mexican-origin children: Examining differences between school year and summer perceptions. BMC Public Health. 2014;14:958.
205. Van Der Horst K, Paw MJCA, Twisk JWR, Van Mechelen W. A brief review on correlates of physical activity and sedentariness in youth. Med Sci Sports Exerc. 2007;39:1241-1250.
206. Vancampfort D, Van Damme T, Firth J, Smith J, Stubbs B, Rosenbaum S, et al. Correlates of physical activity among 142,118 adolescents aged 12-15 years from 48 low- and middle-income countries. Prev Med. 2019:105819.
207. Vander Ploeg KA, Kuhle S, Maximova K, McGavock J, Wu B, Veugelers PJ. The importance of parental beliefs and support for pedometer-measured physical activity on school days and weekend days among Canadian children. BMC Public Health. 2013;13:1132.
208. Vander Ploeg KA, Maximova K, Kuhle S, Simen-Kapeu A, Veugelers PJ. The importance of parental beliefs and support for physical activity and body weights of children: A population-based analysis. Can J Public Health. 2012;103:e277-281.
209. Vaughn AE, Hales D, Ward DS. Measuring the physical activity practices used by parents of preschool children. Med Sci Sports Exerc. 2013;45:2369-2377.
210. Verloigne M, Cardon G, De Craemer M, D'Haese S, De Bourdeaudhuij I. Mediating effects of self-efficacy, benefits and barriers on the association between peer and parental factors and physical activity among adolescent girls with a lower educational level. PloS one. 2016;11:e0157216.
211. Verloigne M, Van Lippevelde W, Maes L, Brug J, De Bourdeaudhuij I. Family- and school-based predictors of energy balance-related behaviours in children: A 6-year longitudinal study. Public Health Nutr. 2013;16:202-211.
212. Villard LC, Ryden L, Stahle A. Predictors of healthy behaviours in Swedish school children. Eur J Cardiovasc Prev Rehabil. 2007;14:366-372.
213. Wang M, Druker S, Gapinski MA, Gellar L, Schneider K, Osganian S, et al. The role of social support vs. modeling on adolescents' diet and physical activity: Findings from a school-based weight management trial. J Child Adolesc Behav. 2014;2.
214. Wang X, Hui Z, Terry PD, Ma M, Cheng L, Deng F, et al. Correlates of insufficient physical activity among junior high school students: A cross-sectional study in Xi'an, China. Int J Environ Res Public Health. 2016;13:397.
215. Wang X, Liu Q-M, Ren Y-J, Lv J, Li L-M. Family influences on physical activity and sedentary behaviours in Chinese junior high school students: A cross-sectional study. BMC Public Health. 2015;15:287.
216. Wang Y, Hager ER, Magder LS, Arbaiza R, Wilkes S, Black MM. A dyadic analysis on source discrepancy and a mediation analysis via self-efficacy in the parental support and physical activity relationship among black girls. Child Obes. 2019;15:123-130.
217. Waters E, de Silva-Sanigorski A, Hall BJ, Brown T, Campbell KJ, Gao Y, et al. Interventions for preventing obesity in children. Cochrane Database Syst Rev. 2011:CD001871.
218. Welch JD, Ellis EM, Green PA, Ferrer RA. Social support, loneliness, eating, and activity among parent-adolescent dyads. J Behav Med. 2019;42:1015-1028.
219. Welk GJ, Wood K, Morss G. Parental influences on physical activity in children: An exploration of potential mechanisms. Pediatr Exerc Sci*.* 2003;15:19-33.
220. Wenthe PJ, Janz KF, Levy SM. Gender similarities and differences in factors associated with adolescent moderate-vigorous physical activity. Pediatr Exerc Sci. 2009;21:291-304.
221. Wilk P, Clark AF, Maltby A, Smith C, Tucker P, Gilliland JA. Examining individual, interpersonal, and environmental influences on children's physical activity levels. SSM Popul Health. 2018;4:76-85.
222. Wilk P, Clark AF, Maltby A, Tucker P, Gilliland JA. Exploring the effect of parental influence on children's physical activity: The mediating role of children's perceptions of parental support. Prev Med*.* 2018;106:79-85.
223. Williams SL, Mummery WK. Links between adolescent physical activity, body mass index, and adolescent and parent characteristics. Health Educ Behav*.* 2011;38:510-520.
224. Wilson AN, Dollman J. Social influences on physical activity in Anglo- and Vietnamese-Australian adolescent males in a single sex school. J Sci Med Sport. 2007;10:147-155.
225. Wilson AN, Dollman J. Social influences on physical activity in Anglo-Australian and Vietnamese-Australian adolescent females in a single sex school. J Sci Med Sport. 2009;12:119-122.
226. Wright MS, Wilson DK, Griffin S, Evans A. A qualitative study of parental modeling and social support for physical activity in underserved adolescents. Health Educ Res. 2010;25:224-232.
227. Wu TY, Pender N, Noureddine S. Gender differences in the psychosocial and cognitive correlates of physical activity among Taiwanese adolescents: A structural equation modeling approach. Int J Behav Med. 2003;10:93-105.
228. Yao CA, Rhodes RE. Parental correlates in child and adolescent physical activity: A meta-analysis. Int J Behav Nutr Phys Act. 2015;12:10.
229. Zakarian JM, Hovell MF, Hofstetter CR, Sallis JF, Keating KJ. Correlates of vigorous exercise in a predominantly low SES and minority high school population. Prev Med*.* 1994;23:314-321.
230. Zecevic CA, Tremblay L, Lovsin T, Michel L. Parental influence on young children's physical activity. Int J Pediatr*.* 2010;2010:468526.
231. Zhao J, Gao Z, Settles BH. Determinants of parental perception and support on youth physical activity. Fam Community Health. 2013;36:77-88.
232. Zhao J, Settles BH. Environmental correlates of children's physical activity and obesity. Am J Health Behav. 2014;38:124-133.
233. Zook KR, Saksvig BI, Wu TT, Young DR. Physical activity trajectories and multilevel factors among adolescent girls. J Adolesc Health. 2014;54:74-80.

**Sociodemographic factors (n=172)**

(e.g., parental age, parental education, household income)

1. Aarnio M, Winter T, Kujala UM, Kaprio J. Familial aggregation of leisure-time physical activity -- A three generation study. Int J Sports Med. 1997;18:549-556.
2. Aarts MJ, de Vries SI, van Oers HAM, Schuit AJ. Outdoor play among children in relation to neighborhood characteristics: A cross-sectional neighborhood observation study. Int J Behav Nutr Phys Act. 2012;9:98.
3. Alharbi M. Influence of individual and family factors on physical activity among Saudi girls: A cross-sectional study. Ann Saudi Med. 2019;39:13-21.
4. Alves CF, Silva Rde C, Assis AM, Souza Cde O, Pinto Ede J, Frainer DE. Factors associated with physical inactivity in adolescents aged 10-14 years, enrolled in the public school network of the city of Salvador, Brazil. Rev Bras Epidemiol. 2012;15:858-870.
5. Aniza I, Fairuz MR. Factors influencing physical activity level among secondary school adolescents in Petaling District, Selangor. Med J Malaysia. 2009;64:228-232.
6. Arredondo EM, Elder JP, Ayala GX, Campbell N, Baquero B, Duerksen S. Is parenting style related to children’s healthy eating and physical activity in Latino families?. Health Educ Res. 2006;21:862-871.
7. Azeredo CM, Levy RB, Peres MF, Menezes PR, Araya R. Patterns of health-related behaviours among adolescents: A cross-sectional study based on the national survey of school health Brazil 2012. BMJ Open. 2016;6:e011571.
8. Babey SH, Hastert TA, Huang W, Brown ER. Sociodemographic, family, and environmental factors associated with active commuting to school among US adolescents. J Public Health Policy. 2009;30 Suppl 1:s203-220.
9. Bagordo F, De Donno A, Grassi T, Guido M, Devoti G, Ceretti E, et al. Lifestyles and socio-cultural factors among children aged 6-8 years from five Italian towns: The MAPEC_LIFE study cohort. BMC Public Health. 2017;17:233.
10. Ball K, Cleland VJ, Timperio AF, Salmon J, Crawford DA. Socioeconomic position and children’s physical activity and sedentary behaviors: Longitudinal findings from the CLAN study. J Phys Act Health. 2009;6:289-298.
11. Bastos JP, Araujo CLP, Hallal PC. Prevalence of insufficient physical activity and associated factors in Brazilian adolescents. J Phys Act Health. 2008;5:777-794.
12. Bauer KW, Neumark-Sztainer D, Fulkerson JA, Story M. Adolescent girls’ weight-related family environments, Minnesota. Prev Chronic Dis. 2011;8:a68.
13. Beech BM, Kumanyika SK, Baranowski T, Davis M, Robinson TN, Sherwoood NE, et al. Parental cultural perspectives in relation to weight-related behaviors and concerns of African-American girls. Obes Res. 2004;12 Suppl:s7-19.
14. Beets MW, Foley JT. Association of father involvement and neighborhood quality with kindergartners’ physical activity: A multilevel structural equation model. Am J Health Promot. 2008;22:195-203.
15. Belanger-Gravel A, Gauvin L, Lagarde F, Laferte M. Correlates and moderators of physical activity in parent-tween dyads: A socio-ecological perspective. Public Health. 2015;129:1218-1223.
16. Bharati DR, Deshmukh PR, Garg BS. Correlates of overweight & obesity among school going children of Wardha city, Central India. Indian J Med Res. 2008;127:539-543.
17. Boxberger K, Reimers AK. Parental correlates of outdoor play in boys and girls aged 0 to 12-A systematic review. Int J Environ Res Public Health. 2019;16:e190.
18. Breslin G, Shannon S, Rafferty R, Fitzpatrick B, Belton S, O’Brien W, et al. The effect of sport for LIFE: All island in children from low socio-economic status: A clustered randomized controlled trial. Health Qual Life Outcomes. 2019;17:66.
19. Brophy S, Cooksey R, Lyons RA, Thomas NE, Rodgers SE, Gravenor MB. Parental factors associated with walking to school and participation in organised activities at age 5: Analysis of the millennium cohort study. BMC Public Health. 2011;11:14.
20. Brown HE, Corder K, Atkin AJ, van Sluijs EMF. Childhood predictors of adolescent behaviour: The prospective association of familial factors with meeting physical activity guidelines. Prev Med reports. 2017;6:221-227.
21. Butte NF, Gregorich SE, Tschann JM, Penilla C, Pasch LA, De Groat CL, et al. Longitudinal effects of parental, child and neighborhood factors on moderate-vigorous physical activity and sedentary time in Latino children. Int J Behav Nutr Phys Act. 2014;11:108.
22. Cabrera NJ, Hofferth SL, Chae S. Patterns and predictors of father-infant engagement across race/ethnic groups. Early Child Res Q. 2011;26:365-375.
23. Cadogan SL, Keane E, Kearney PM. The effects of individual, family and environmental factors on physical activity levels in children: A cross-sectional study. BMC Pediatr. 2014;14:107.
24. Cameron AJ, Ball K, Hesketh KD, McNaughton SA, Salmon J, Crawford DA, et al. Variation in outcomes of the Melbourne infant, feeding, activity and nutrition trial (InFANT) program according to maternal education and age. Prev Med. 2014;58:58-63.
25. Chang SH, Kim K. A review of factors limiting physical activity among young children from low-income families. J Exerc Rehabil. 2017;13:375-377.
26. Cheung PPY. Children’s after-school physical activity participation in Hong Kong: Does family socioeconomic status matter? Health Educ J. 2017;76:221-230.
27. Chillon P, Ortega FB, Ruiz JR, Perez IJ, Martin-Matillas M, Valtuena J, et al. Socio-economic factors and active commuting to school in urban Spanish adolescents: The AVENA study. Eur J Public Health. 2009;19:470-476.
28. Christofaro DGD, Turi-Lynch BC, Lynch KR, Tebar WR, Fernandes RA, Tebar FG, et al. Parents’ lifestyle, sedentary behavior, and physical activity in their children: A cross-sectional study in Brazil. J Phys Act Health. 2019;16:631-636.
29. Cislak A, Safron M, Pratt M, Gaspar T, Luszczynska A. Family-related predictors of body weight and weight-related behaviours among children and adolescents: A systematic umbrella review. Child Care Health Dev. 2012;38:321-331.
30. Coledam DH, Ferraiol PF, Pires R Jr, Ribeiro EA, Ferreira MA, de Oliveira AR. [Agreement between two cutoff points for physical activity and associated factors in young individuals]. Rev Paul Pediatr. 2014;32:215-222.
31. Condessa LA, Chaves OC, Silva FM, Malta DC, Caiaffa WT. Sociocultural factors related to the physical activity in boys and girls: PeNSE 2012. Rev Saude Publica. 2019;53:25.
32. Cottrell L, Zatezalo J, Bonasso A, Lattin J, Shawley S, Murphy E, et al. The relationship between children’s physical activity and family income in rural settings: A cross-sectional study. Prev Med Reports. 2015;2:99-104.
33. Crawford D, Cleland V, Timperio A, Salmon J, Andrianopoulos N, Roberts R, et al. The longitudinal influence of home and neighbourhood environments on children’s body mass index and physical activity over 5 years: The CLAN study. Int J Obes (Lond). 2010;34:1177-1187.
34. da Costa BGG, da Silva KS, da Silva JA, Minatto G, de Lima LRA, Petroski EL. Sociodemographic, biological, and psychosocial correlates of light- and moderate-to-vigorous-intensity physical activity during school time, recesses, and physical education classes. J Sport Heal Sci. 2019;8:177-182.
35. Dan SP, Mohd NMT, Zalilah MS. Determination of factors associated with physical activity levels among adolescents attending school in Kuantan, Malaysia. Malays J Nutr. 2011;17:175-187.
36. Davis AM, Daldalian MC, Mayfield CA, Dean K, Black WR, Sampilo ML, et al. Outcomes from an urban pediatric obesity program targeting minority youth: The healthy hawks program. Child Obes. 2013;9:492-500.
37. Dawson-Hahn EE, Fesinmeyer MD, Mendoza JA. Correlates of physical activity in Latino preschool children attending head Start. Pediatr Exerc Sci. 2015;27:372-379.
38. De Cocker K, Artero EG, De Henauw S, Dietrich S, Gottrand F, Beghin L, et al. Can differences in physical activity by socio-economic status in European adolescents be explained by differences in psychosocial correlates? A mediation analysis within the HELENA (Healthy Lifestyle in Europe by Nutrition in Adolescence) study. Public Health Nutr. 2012;15:2100-2109.
39. Dearth-Wesley T, Gordon-Larsen P, Adair LS, Zhang B, Popkin BM. Longitudinal, cross-cohort comparison of physical activity patterns in Chinese mothers and children. Int J Behav Nutr Phys Act. 2012;9:39.
40. Diep CS, Leung R, Thompson D, Gor BJ, Baranowski T. Physical activity behaviors and influences among Chinese-American children aged 9-13 years: A qualitative study. J Immigr Minor Heal. 2017;19:358-366.
41. Dollman J, Lewis NR. The impact of socioeconomic position on sport participation among South Australian youth. J Sci Med Sport. 2010;13:318-322.
42. Dregval L, Petrauskiene A. Associations between physical activity of primary school first-graders during leisure time and family socioeconomic status. Medicina (Kaunas). 2009;45:549-556.
43. Dumith SC, Gigante DP, Domingues MR, Hallal PC, Menezes AMB, Kohl HW 3rd. Predictors of physical activity change during adolescence: A 3.5-year follow-up. Public Health Nutr. 2012;15:2237-2245.
44. Echeverria SE, Ohri-Vachaspati P, Yedidia MJ. The influence of parental nativity, neighborhood disadvantage and the built environment on physical activity behaviors in Latino youth. J Immigr Minor Heal. 2015;17:519-526.
45. Eime RM, Harvey JT, Craike MJ, Symons CM, Payne WR. Family support and ease of access link socio-economic status and sports club membership in adolescent girls: A mediation study. Int J Behav Nutr Phys Act. 2013;10:50.
46. Federico B, Falese L, Capelli G. Socio-economic inequalities in physical activity practice among Italian children and adolescents: A cross-sectional study. Z Gesundh Wiss. 2009;17:377-384.
47. Ferreira I, van der Horst K, Wendel-Vos W, Kremers S, van Lenthe FJ, Brug J. Environmental correlates of physical activity in youth - A review and update. Obes Rev. 2007;8:129-154.
48. Finger JD, Mensink GBM, Banzer W, Lampert T, Tylleskar T. Physical activity, aerobic fitness and parental socio-economic position among adolescents: The German health interview and examination survey for children and adolescents 2003-2006 (KiGGS). Int J Behav Nutr Phys Act. 2014;11:43.
49. Finn K, Johannsen N, Specker B. Factors associated with physical activity in preschool children. J Pediatr. 2002;140:81-85.
50. Frenn M, Malin S, Villarruel AM, Slaikeu K, McCarthy S, Freeman J, et al. Determinants of physical activity and low-fat diet among low income African American and Hispanic middle school students. Public Health Nurs. 2005;22:89-97.
51. Ghekiere A, Deforche B, Carver A, Mertens L, de Geus B, Clarys P, et al. Insights into children’s independent mobility for transportation cycling-Which socio-ecological factors matter?. J Sci Med Sport. 2017;20:267-272.
52. Ghekiere A, Van Cauwenberg J, Carver A, Mertens L, de Geus B, Clarys P, et al. Pyschosocial factors associated with children’s cycling for transport: A cross-sectional moderation study. Prev Med. 2016;86:141-146.
53. Glozah FN, Pevalin DJ. Perceived social support and parental education as determinants of adolescents’ physical activity and eating behaviour: A cross-sectional survey. Int J Adolesc Med Health. 2015;27:253-259.
54. Gomes TN, Dos Santos FK, Garganta RM, Kenny DA, Katzmarzyk PT, Maia JA. Multi-level modelling of physical activity in nuclear families. Ann Hum Biol. 2014;41:138-144.
55. Gomes TN, Hedeker D, Dos Santos FK, Souza M, Santos D, Pereira S, et al. Relationship between sedentariness and moderate-to-vigorous physical activity in youth: A multivariate multilevel study. Int J Environ Res Public Health. 2017;14:e148.
56. Gorely T, Atkin AJ, Biddle SJH, Marshall SJ. Family circumstance, sedentary behaviour and physical activity in adolescents living in England: Project STIL. Int J Behav Nutr Phys Act. 2009;6:33.
57. Gottlieb NH, Chen MS. Sociocultural correlates of childhood sporting activities: Their implications for heart health. Soc Sci Med. 1985;21:533-539.
58. Gregori D, Foltran F, Ghidina M, Zobec F, Berchialla P. Familial environment in high- and middle-low-income municipalities: A survey in Italy to understand the distribution of potentially obesogenic factors. Public Health. 2012;126:731-739.
59. Gubbels JS, Kremers SPJ, Stafleu A, Goldbohm RA, de Vries NK, Thijs C. Clustering of energy balance-related behaviors in 5-year-old children: Lifestyle patterns and their longitudinal association with weight status development in early childhood. Int J Behav Nutr Phys Act. 2012;9:77.
60. Gulati A, Hochdorn A, Paramesh H, Paramesh EC, Chiffi D, Kumar M, et al. Physical activity patterns among school children in India. Indian J Pediatr. 2014;81:47-54.
61. Gustafson SL, Rhodes RE. Parental correlates of physical activity in children and early adolescents. Sports Med. 2006;36:79-97.
62. Gwozdz W, Sousa-Poza A, Reisch LA, Ahrens W, Eiben G, M Fernandez-Alvira J, et al. Maternal employment and childhood obesity - A European perspective. J Health Econ. 2013;32:728-742.
63. Hallmann K, Breuer C. The influence of socio-demographic indicators economic determinants and social recognition on sport participation in Germany. Eur J Sport Sci. 2014;14 Suppl 1:s324-331.
64. Hashem R, Rey-Lopez JP, Hamer M, McMunn A, Whincup PH, Owen CG, et al. Physical activity and sedentary behaviors levels of Kuwaiti adolescents: The study of health and activity among adolescents in Kuwait. J Phys Act Health. 2018;15:255-262.
65. Hearst MO, Patnode CD, Sirard JR, Farbakhsh K, Lytle LA. Multilevel predictors of adolescent physical activity: A longitudinal analysis. Int J Behav Nutr Phys Act. 2012;9:8.
66. Hesketh KR, Brage S, Cooper C, Godfrey KM, Harvey NC, Inskip HM, et al. The association between maternal-child physical activity levels at the transition to formal schooling: Cross-sectional and prospective data from the Southampton women’s survey. Int J Behav Nutr Phys Act. 2019;16:23.
67. Hesketh KR, Goodfellow L, Ekelund U, McMinn AM, Godfrey KM, Inskip HM, et al. Activity levels in mothers and their preschool children. Pediatrics. 2014;133:e973-80.
68. Hesketh K, Graham M, Waters E. Children’s after school activity: Associations with weight status and family circumstance. Pediatr Exerc Sci. 2008;20:84-94.
69. Hnatiuk JA, Hesketh KR, van Sluijs EMF. Correlates of home and neighbourhood-based physical activity in UK 3-4-year-old children. Eur J Public Health. 2016;26:947-953.
70. Hung H, Lee CW. Relationship between ethnic backgrounds, family socioeconomic status, leisure participation, and psychological well-being. Percept Mot Skills. 2013;117:367-375.
71. Huppertz C, Bartels M, de Geus EJC, van Beijsterveldt CEM, Rose RJ, Kaprio J, et al. The effects of parental education on exercise behavior in childhood and youth: A study in Dutch and Finnish twins. Scand J Med Sci Sports. 2017;27:1143-1156.
72. Inskip H, Baird J, Barker M, Briley AL, D’Angelo S, Grote V, et al. Influences on adherence to diet and physical activity recommendations in women and children: Insights from six European studies. Ann Nutr Metab. 2014;64:332-339.
73. Izaki T, Swaine I. Physical activity and obesity among year 7 children in Kent, UK: Gender, social background and implications for school health promotion. Int J Heal Promot Educ. 2017;55:189-204.
74. Janssen I, Ferrao T, King N. Individual, family, and neighborhood correlates of independent mobility among 7 to 11-year-olds. Prev Med reports. 2016;3:98-102.
75. Janssen I, Levesque L, Xu F, First Nations Information Governance Centre. Correlates of physical activity among First Nations children residing in First Nations communities in Canada. Can J Public Health. 2014;105:e412-7.
76. Jimenez-Pavon D, Fernandez-Alvira JM, Te Velde SJ, Brug J, Bere E, Jan N, et al. Associations of parental education and parental physical activity (PA) with children’s PA: The ENERGY cross-sectional study. Prev Med. 2012;55:310-314.
77. Kader M, Sundblom E, Elinder LS. Effectiveness of universal parental support interventions addressing children’s dietary habits, physical activity and bodyweight: A systematic review. Prev Med. 2015;77:52-67.
78. Kaiser-Jovy S, Scheu A, Greier K. Media use, sports activities, and motor fitness in childhood and adolescence. Wien Klin Wochenschr. 2017;129:464-471.
79. Kalil A, Ryan R, Corey M. Diverging destinies: Maternal education and the developmental gradient in time with children. Demography. 2012;49:1361-1383.
80. Kantomaa MT, Tammelin TH, Nayha S, Taanila AM. Adolescents’ physical activity in relation to family income and parents’ education. Prev Med. 2007;44:410-415.
81. Kari JT, Tammelin TH, Viinikainen J, Hutri-Kahonen N, Raitakari OT, Pehkonen J. Childhood Physical Activity and Adulthood Earnings. Med Sci Sports Exerc. 2016;48:1340-1346.
82. Kelishadi R, Ghatrehsamani S, Hosseini M, Mirmoghtadaee P, Mansouri S, Poursafa P. Barriers to physical activity in a population-based sample of children and adolescents in Isfahan, Iran. Int J Prev Med. 2010;1:131-137.
83. King AC, Parkinson KN, Adamson AJ, Murray L, Besson H, Reilly JJ, et al. Correlates of objectively measured physical activity and sedentary behaviour in English children. Eur J Public Health. 2011;21:424-431.
84. Knuth AG, Silva ICM, van Hees VT, Cordeira K, Matijasevich A, Barros AJD, et al. Objectively-measured physical activity in children is influenced by social indicators rather than biological lifecourse factors: Evidence from a Brazilian cohort. Prev Med. 2017;97:40-44.
85. Kwon S, Janz KF, Letuchy EM, Burns TL, Levy SM. Parental characteristic patterns associated with maintaining healthy physical activity behavior during childhood and adolescence. Int J Behav Nutr Phys Act. 2016;13:58.
86. La Torre G, Masala D, De Vito E, Arzano I, Fargione V, Capelli G. Physical activity and socio-economic status: Results of a pilot study. Med dello Sport. 2003;56:175-183.
87. La Torre G, Masala D, De Vito E, Langiano E, Capelli G, Ricciardi W. Extra-curricular physical activity and socioeconomic status in Italian adolescents. BMC Public Health. 2006;6:22.
88. Lam CB, McHale SM. Developmental patterns and parental correlates of youth leisure-time physical activity. J Fam Psychol. 2015;29:100-107.
89. Langlois J, Omorou AY, Vuillemin A, Briancon S, Lecomte E, Group PT. Association of socioeconomic, school-related and family factors and physical activity and sedentary behaviour among adolescents: Multilevel analysis of the PRALIMAP trial inclusion data. BMC Public Health. 2017;17:175.
90. Latorre Roman PA, Mora Lopez D, Garcia Pinillos F. Feeding practices, physical activity, and fitness in Spanish preschoolers: Influence of sociodemographic outcome measures. Arch Argent Pediatr. 2016;114:441-447.
91. Laukkanen A, Niemisto D, Finni T, Cantell M, Korhonen E, Saakslahti A. Correlates of physical activity parenting: The skilled kids study. Scand J Med Sci Sports. 2018;28:2691-2701.
92. Lavielle-Sotomayor P, Pineda-Aquino V, Jauregui-Jimenez O, Castillo-Trejo M. [Physical activity and sedentary lifestyle: Family and socio-demographic determinants and their impact on adolescents’ health]. Rev Salud Publica. 2014;16:161-172.
93. Lee H, Cardinal BJ, Loprinzi PD. Effects of socioeconomic status and acculturation on accelerometer-measured moderate-to-vigorous physical activity among Mexican American adolescents: Findings from NHANES 2003-2004. J Phys Act Heal. 2012;9:1155-1162.
94. Lee SM, Nihiser A, Strouse D, Das B, Michael S, Huhman M. Correlates of children and parents being physically active together. J Phys Act Health. 2010;7:776-783.
95. Lindquist CH, Reynolds KD, Goran MI. Sociocultural determinants of physical activity among children. Prev Med. 1999;29:305-312.
96. Liu GC, Wiehe SE, Aalsma MC. Associations between child and sibling levels of vigorous physical activity in low-income minority families. Int J Pediatr Adolesc Med. 2014;1:61-68.
97. Long DE, Gaetke LM, Perry SD, Abel MG, Clasey JL. The assessment of physical activity and nutrition in home schooled versus public schooled children. Pediatr Exerc Sci. 2010;22:44-59.
98. Loprinzi PD, Davis RE. Recent temporal trends in parent-reported physical activity in children in the United States, 2009 to 2014. Mayo Clin Proc. 2016;91:477-481.
99. Loprinzi PD, Schary DP, Cardinal BJ. Adherence to active play and electronic media guidelines in preschool children: Gender and parental education considerations. Matern Child Health J. 2013;17:56-61.
100. Lu W, McKyer ELJ, Lee C, Ory MG, Goodson P, Wang S. Children’s active commuting to school: An interplay of self-efficacy, social economic disadvantage, and environmental characteristics. Int J Behav Nutr Phys Act. 2015;12:29.
101. Lundahl A, Nelson TD, Van Dyk TR, West T. Psychosocial stressors and health behaviors: Examining sleep, sedentary behaviors, and physical activity in a low-income pediatric sample. Clin Pediatr. 2013;52:721-729.
102. Maher JP, Ra C, O’Connor SG, Belcher BR, Leventhal A, Margolin G, et al. Associations between maternal mental health and well-being and physical activity and sedentary behavior in children. J Dev Behav Pediatr. 2017;38:385-394.
103. Martin MA, Lippert AM, Chandler KD, Lemmon M. Does mothers’ employment affect adolescents’ weight and activity levels? Improving our empirical estimates. SSM - Popul Heal. 2018;4:291-300.
104. Martins J, Marques A, Sarmento H, Carreiro da Costa F. Adolescents’ perspectives on the barriers and facilitators of physical activity: A systematic review of qualitative studies. Health Educ Res. 2015;30:742-755.
105. McMinn AM, van Sluijs EMF, Nightingale CM, Griffin SJ, Cook DG, Owen CG, et al. Family and home correlates of children’s physical activity in a multi-ethnic population: The cross-sectional child heart and health study in England (CHASE). Int J Behav Nutr Phys Act. 2011;8:11.
106. McVeigh JA, Norris SA, de Wet T. The relationship between socio-economic status and physical activity patterns in South African children. Acta Paediatr. 2004;93:982-988.
107. Monge-Rojas R, Nunez HP, Garita C, Chen-Mok M. Psychosocial aspects of Costa Rican adolescents’ eating and physical activity patterns. J Adolesc Health. 2002;31:212-219.
108. Moraeus L, Lissner L, Olsson L, Sjoberg A. Age and time effects on children’s lifestyle and overweight in Sweden. BMC Public Health. 2015;15:355.
109. Muhajarine N, Katapally TR, Fuller D, Stanley KG, Rainham D. Longitudinal active living research to address physical inactivity and sedentary behaviour in children in transition from preadolescence to adolescence. BMC Public Health. 2015;15:495.
110. Mulhall P, Reis J, Begum S. Early adolescent participation in physical activity: Correlates with individual and family characteristics. J Phys Act Heal. 2011;8:244-252.
111. Muthuri SK, Onywera VO, Tremblay MS, Broyles ST, Chaput JP, Fogelholm M, et al. Relationships between parental education and overweight with childhood overweight and physical activity in 9-11 year old children: Results from a 12-country study. PLoS One. 2016;11:e0147746.
112. Mutz M, Albrecht P. Parents’ social status and children’s daily physical activity: The role of familial socialization and support. J Child Fam Stud. 2017;26:3026-3035.
113. Nitzan Kaluski D, Demem Mazengia G, Shimony T, Goldsmith R, Berry EM. Prevalence and determinants of physical activity and lifestyle in relation to obesity among schoolchildren in Israel. Public Health Nutr. 2009;12:774-782.
114. O'Loughlin J, Paradis G, Kishchuk N, Barnett T, Renaud L. Prevalence and correlates of physical activity behaviors among elementary schoolchildren in multiethnic, low income, inner-city neighborhoods in Montreal, Canada. Ann Epidemiol. 1999;9:397-407.
115. Oosterhoff M, Joore MA, Bartelink NHM, Winkens B, Schayck OCP, Bosma H. Longitudinal analysis of health disparities in childhood. Arch Dis Child. 2019;104:781-788.
116. Ostbye T, Malhotra R, Stroo M, Lovelady C, Brouwer R, Zucker N, et al. The effect of the home environment on physical activity and dietary intake in preschool children. Int J Obes. 2013;37:1314-1321.
117. Park H, Kim N. Predicting factors of physical activity in adolescents: A systematic review. Asian Nurs Res. 2008;2:113-128.
118. Peltzer K, Pengpid S. Leisure time physical inactivity and sedentary behaviour and lifestyle correlates among students aged 13-15 in the association of Southeast Asian nations (ASEAN) member states, 2007-2013. Int J Environ Res Public Health. 2016;13:217.
119. Piko BF, Keresztes N. Sociodemographic and socioeconomic variations in leisure time physical activity in a sample of Hungarian youth. Int J Public Health. 2008;53:306-310.
120. Pitel L, Madarasova Geckova A, Reijneveld SA, van Dijk JP. Socioeconomic differences in adolescent health-related behavior differ by gender. J Epidemiol. 2013;23:211-218.
121. Poulain T, Vogel M, Sobek C, Hilbert A, Korner A, Kiess W. Associations between socio-economic status and child health: Findings of a large German cohort study. Int J Environ Res Public*.* 2019;16:e677.
122. Pouliou T, Sera F, Griffiths L, Joshi H, Geraci M, Cortina-Borja M, et al. Environmental influences on children's physical activity. J Epidemiol Community Health. 2015;69:77-85.
123. Price SM, McDivitt J, Weber D, Wolff LS, Massett HA, Fulton JE. Correlates of weight-bearing physical activity among adolescent girls: Results from a national survey of girls and their parents. J Phys Act Health. 2008;5:132-145.
124. Quon EC, McGrath JJ. Community, family, and subjective socioeconomic status: Relative status and adolescent health. Health Psychol. 2015;34:591-601.
125. Ramos CGC, Andrade RG, Andrade ACS, Fernandes AP, Costa DADS, Xavier CC, et al. Family context and the physical activity of adolescents: Comparing differences. Rev Bras Epidemiol. 2017;20:537-548.
126. Raudsepp L. The relationship between socio-economic status, parental support and adolescent physical activity. Acta Paediatr. 2006;95:93-98.
127. Remmers T, Van Kann D, Gubbels J, Schmidt S, de Vries S, Ettema D, et al. Moderators of the longitudinal relationship between the perceived physical environment and outside play in children: The KOALA birth cohort study. Int J Behav Nutr Phys Act. 2014;11:150.
128. Rhodes RE, Spence JC, Berry T, Deshpande S, Faulkner G, Latimer-Cheung AE, et al. Predicting changes across 12 months in three types of parental support behaviors and mothers' perceptions of child physical activity. Ann Behav Med. 2015;49:853-864.
129. Riazi NA, Blanchette S, Trudeau F, Larouche R, Tremblay MS, Faulkner G. Correlates of children's independent mobility in Canada: A multi-site study. Int J Environ Res Public Health*.* 2019;16:e2862.
130. Richards R, Poulton R, Reeder AI, Williams S. Childhood and contemporaneous correlates of adolescent leisure time physical inactivity: A longitudinal study. J Adolesc Health. 2009;44:260-267.
131. Riddoch CJ, Mattocks C, Deere K, Saunders J, Kirkby J, Tilling K, et al. Objective measurement of levels and patterns of physical activity. Arch Dis Child. 2007;92:963-969.
132. Rodrigues D, Padez C, Machado-Rodrigues AM. Child participation in sports is influenced by patterns of lifestyle-related behaviors. Am J Hum Biol. 2018;30:e23142.
133. Rodriguez-Lopez C, Villa-Gonzalez E, Perez-Lopez IJ, Delgado-Fernandez M, Ruiz JR, Chillon P. Family factors influence active commuting to school in Spanish children. Nutr Hosp. 2013;28:756-763.
134. Ross A, Kwon JY, Kulinna PH, Searle M. Active transportation: The role of parent attitude, the physical environment, and social capital. J Phys Act Health. 2018:1-8.
135. Rothman L, Macpherson AK, Ross T, Buliung RN. The decline in active school transportation (AST): A systematic review of the factors related to AST and changes in school transport over time in North America. Prev Med. 2018;111:314-322.
136. Santos ML, Simoes TB, Monteiro LA, Novaes JS, Fernandes HM. Longitudinal effects of biopsychosocial variables on physical activity after menarche. Minerva Pediatr. 2018.
137. Sanz-Arazuri E, Ponce-de-Leon-Elizondo A, Valdemoros-San-Emeterio MA. Parental predictors of physical inactivity in Spanish adolescents. J Sports Sci Med. 2012;11:95-101.
138. Saunders J, Hume C, Timperio A, Salmon J. Cross-sectional and longitudinal associations between parenting style and adolescent girls' physical activity. Int J Behav Nutr Phys Act. 2012;9:141.
139. Schaefer SE, Gomez-Camacho R, Martinez L, Sadeghi B, German JB, de la Torre A. Social and environmental determinants of child physical activity in a rural Mexican-origin community. J Community Health. 2016;41:409-416.
140. Schott K, Hunger M, Lampert T, Spengler S, Mess F, Mielck A. [Social differences in physical activity among adolescents in Germany: Analyses based on information concerning the metabolic equivalent of task (MET)]. Gesundheitswesen. 2016;78:630-636.
141. Seabra AF, Mendonca DM, Thomis MA, Malina RM, Maia JA. Correlates of physical activity in Portuguese adolescents from 10 to 18 years. Scand J Med Sci Sports. 2011;21:318-323.
142. Seabra AF, Mendonca DM, Thomis MA, Anjos LA, Maia JA. Biological and socio-cultural determinants of physical activity in adolescents. Cad Saude Publica. 2008;24:721-736.
143. Seabra AF, Mendonca DM, Thomis MA, Peters TJ, Maia JA. Associations between sport participation, demographic and socio-cultural factors in Portuguese children and adolescents. Eur J Public Health. 2008;18:25-30.
144. Sherar LB, Muhajarine N, Esliger DW, Baxter-Jones ADG. The relationship between girls' (8-14 years) physical activity and maternal education. Ann Hum Biol. 2009;36:573-583.
145. Siegel SR, Malina RM, Reyes MEP, Barahona EEC, Cumming SP. Correlates of physical activity and inactivity in urban Mexican youth. Am J Hum Biol. 2011;23:686-692.
146. Sigmund E, Badura P, Sigmundova D, Voracova J, Zacpal J, Kalman M, et al. Trends and correlates of overweight/obesity in Czech adolescents in relation to family socioeconomic status over a 12-year study period (2002-2014). BMC Public Health. 2018;18:122.
147. Sigmundova D, Sigmund E, Tesler R, Ng KW, Hamrik Z, Mathisen FKS, et al. Vigorous physical activity in relation to family affluence: Time trends in Europe and North America. Int J Public Health. 2019;64:1049-1058.
148. Silva AAPD, Fermino RC, Souza CA, Lima AV, Rodriguez-Anez CR, Reis RS. Socioeconomic status moderates the association between perceived environment and active commuting to school. Rev Saude Publica. 2018;52:93.
149. Simen-Kapeu A, Veugelers PJ. Socio-economic gradients in health behaviours and overweight among children in distinct economic settings. Can J Public Health. 2010;101 Suppl 3:s32-36.
150. Singh GK, Kogan MD, Siahpush M, Van Dyck PC. Independent and joint effects of socioeconomic, behavioral, and neighborhood characteristics on physical inactivity and activity levels among US children and adolescents. J Community Health. 2008;33:206-216.
151. Singh GK, Kogan MD, Siahpush M, van Dyck PC. Prevalence and correlates of state and regional disparities in vigorous physical activity levels among US children and adolescents. J Phys Act Health. 2009;6:73-87.
152. Smith BJ, Grunseit A, Hardy LL, King L, Wolfenden L, Milat A. Parental influences on child physical activity and screen viewing time: A population based study. BMC Public Health. 2010;10:593.
153. Soori H, Bhopal RS. Parental permission for children's independent outdoor activities: Implications for injury prevention. Eur J Public Health. 2002;12:104-109.
154. Sterdt E, Liersch S, Walter U. Correlates of physical activity of children and adolescents: A systematic review of reviews. Health Educ J. 2014;73:72-89.
155. Sullivan SO. The physical activity of children: A study of 1,602 Irish schoolchildren aged 11-12 years. Ir Med J. 2002;95:78-81.
156. Tandon PS, Zhou C, Sallis JF, Cain KL, Frank LD, Saelens BE. Home environment relationships with children's physical activity, sedentary time, and screen time by socioeconomic status. Int J Behav Nutr Phys Act. 2012;9:88.
157. Tandon PS, Zhou C, Christakis DA. Frequency of parent-supervised outdoor play of US preschool-aged children. Arch Pediatr Adolesc Med. 2012;166:707-712.
158. Toftegaard-Stockel J, Nielsen GA, Ibsen B, Andersen LB. Parental, socio and cultural factors associated with adolescents' sports participation in four Danish municipalities. Scand J Med Sci Sports. 2011;21:606-611.
159. Tuinstra J, Groothoff JW, van den Heuvel WJ, Post D. Socio-economic differences in health risk behavior in adolescence: Do they exist? Soc Sci Med. 1998;47:67-74.
160. Van Der Horst K, Paw MJCA, Twisk JWR, Van Mechelen W. A brief review on correlates of physical activity and sedentariness in youth. Med Sci Sports Exerc. 2007;39:1241-1250.
161. Vanwolleghem G, Van Dyck D, De Meester F, De Bourdeaudhuij I, Cardon G, Gheysen F. Which socio-ecological factors associate with a switch to or maintenance of active and passive transport during the transition from primary to secondary school? PloS one. 2016;11:e0156531.
162. Villagran Perez S, Novalbos-Ruiz JP, Rodriguez-Martin A, Martinez-Nieto JM, Lechuga-Sancho AM. Implications of family socioeconomic level on risk behaviors in child-youth obesity. Nutr Hosp. 2013;28:1951-1960.
163. Voss LD, Hosking J, Metcalf BS, Jeffery AN, Wilkin TJ. Children from low-income families have less access to sports facilities, but are no less physically active: Cross-sectional study (EarlyBird 35). Child Care Health Dev. 2008;34:470-474.
164. Wang X, Hui Z, Terry PD, Ma M, Cheng L, Deng F, et al. Correlates of insufficient physical activity among junior high school students: A cross-sectional study in Xi'an, China. Int J Environ Res Public Health. 2016;13:397.
165. Wiley AR, Flood TL, Andrade FCD, Aradillas C, Cerda EM. Family and individual predictors of physical activity for older Mexican adolescents. J Adolesc Health. 2011;49:222-224.
166. Wilk P, Clark AF, Maltby A, Smith C, Tucker P, Gilliland JA. Examining individual, interpersonal, and environmental influences on children's physical activity levels. SSM Popul Health. 2018;4:76-85.
167. Wilkie HJ, Standage M, Gillison FB, Cumming SP, Katzmarzyk PT. The home electronic media environment and parental safety concerns: Relationships with outdoor time after school and over the weekend among 9-11 year old children. BMC Public Health. 2018;18:456.
168. Wu S-s, Wang H-j, Li B-h, Li S-s, Ma J. [Association between socioeconomic status and physical activities in Chinese children]. Zhonghua Liu Xing Bing Xue Za Zhi. 2010;31:513-516.
169. Xu H, Wen LM, Hardy LL, Rissel C. A 5-year longitudinal analysis of modifiable predictors for outdoor play and screen-time of 2- to 5-year-olds. Int J Behav Nutr Phys Act. 2016;13:96.
170. Zaccagni L, Toselli S, Celenza F, Albertini A, Gualdi-Russo E. Sports activities in preschool children differed between those born to immigrants and native Italians. Acta Paediatr. 2017;106:1184-1191.
171. Zahl-Thanem T, Steinsbekk S, Wichstrom L. Predictors of physical activity in middle childhood. A fixed-effects regression approach. Front Public Health. 2018;6:305.
172. Ziviani J, Macdonald D, Ward H, Jenkins D, Rodger S. Physical activity of young children: A two-year follow-up. Phys Occup Ther Pediatr. 2008;28:25-39.

**Parental beliefs, attitudes, knowledge (n=131)**

(e.g., perceived importance of physical activity, attitudes towards physical activity/sport, perceived neighbourbood safety)

1. Anderson CB, Hughes SO, Fuemmeler BF. Parent-child attitude congruence on type and intensity of physical activity: Testing multiple mediators of sedentary behavior in older children. Health Psychol. 2009;28:428-438.
2. Anderssen N, Wold B. Parental and peer influences on leisure-time physical activity in young adolescents. Res Q Exerc Sport. 1992;63:341-348.
3. Ansari H, Farajzadegan Z, Hajigholami A, Paknahad Z. A randomized field trial for the primary prevention of osteoporosis among adolescent females: Comparison of two methods, mother centered and daughter centered. J Res Med Sci. 2014;19:746-752.
4. Barnes AT, Plotnikoff RC, Collins CE, Morgan PJ. Maternal correlates of objectively measured physical activity in girls. Matern Child Health J. 2015;19:2348-2357.
5. Bauer KW, Nelson MC, Boutelle KN, Neumark-Sztainer D. Parental influences on adolescents’ physical activity and sedentary behavior: Longitudinal findings from project EAT-II. Int J Behav Nutr Phys Act. 2008;5:12.
6. Belanger-Gravel A, Gauvin L, Lagarde F, Laferte M. Correlates and moderators of physical activity in parent-tween dyads: A socio-ecological perspective. Public Health. 2015;129:1218-1223.
7. Bergmann K, Mestre Z, Strong D, Eichen DM, Rhee K, Crow S, et al. Comparison of two models of family-based treatment for childhood obesity: A pilot study. Child Obes. 2019;15:116-122.
8. Borg A, Haughton CF, Sawyer M, Lemon SC, Kane K, Pbert L, et al. Design and methods of the healthy kids & families study: A parent-focused community health worker-delivered childhood obesity prevention intervention. BMC Obes. 2019;6:19.
9. Boshoff K, Dollman J, Magarey A. An investigation into the protective factors for overweight among low socio-economic status children. Health Promot J Austr. 2007;18:135-142.
10. Boxberger K, Reimers AK. Parental correlates of outdoor play in boys and girls aged 0 to 12 – a systematic review. Int J Environ Res Public Health. 2019;16:190.
11. Bringolf-Isler B, Grize L, Mader U, Ruch N, Sennhauser FH, Braun-Fahrlander C, et al. Built environment, parents’ perception, and children’s vigorous outdoor play. Prev Med (Baltim). 2010;50:251-256.
12. Bringolf-Isler B, Schindler C, de Hoogh K, Kayser B, Suggs LS, Dössegger A, et al. Association of objectively measured and perceived environment with accelerometer-based physical activity and cycling: A Swiss population-based cross-sectional study of children. Int J Public Health. 2019;64:499-510.
13. Budd EL, Aaron Hipp J, Geary N, Dodson EA. Racial differences in parental perceptions of the neighborhood as predictors of children’s physical activity and sedentary behavior. Prev Med Reports. 2015;2:397-402.
14. Burchett HED, Sutcliffe K, Melendez-Torres GJ, Rees R, Thomas J. Lifestyle weight management programmes for children: A systematic review using qualitative comparative analysis to identify critical pathways to effectiveness. Prev Med (Baltim). 2018;106:1-12.
15. Cadogan SL, Keane E, Kearney PM. The effects of individual, family and environmental factors on physical activity levels in children: A cross-sectional study. BMC Pediatr. 2014;14:107.
16. Cameron AJ, Ball K, Hesketh KD, McNaughton SA, Salmon J, Crawford DA, et al. Variation in outcomes of the Melbourne infant, feeding, activity and nutrition trial (InFANT) program according to maternal education and age. Prev Med (Baltim). 2014;58:58-63.
17. Campbell KJ, Hesketh KD, McNaughton SA, Ball K, McCallum Z, Lynch J, et al. The extended infant feeding, activity and nutrition trial (InFANT Extend) program: A cluster-randomized controlled trial of an early intervention to prevent childhood obesity. BMC Public Health. 2016;16:166.
18. Campbell KJ, Lioret S, McNaughton SA, Crawford DA, Salmon J, Ball K, et al. A parent-focused intervention to reduce infant obesity risk behaviors: A randomized trial. Pediatrics. 2013;131:652-660.
19. Campbell K, Hesketh K, Crawford D, Salmon J, Ball K, McCallum Z. The infant feeding activity and nutrition trial (INFANT) an early intervention to prevent childhood obesity: cluster-randomised controlled trial. BMC Public Health. 2008;8:103.
20. Carson V, Kuhle S, Spence JC, Veugelers PJ. Parents’ perception of neighbourhood environment as a determinant of screen time, physical activity and active transport. Can J Public Health. 2010;101:124-127.
21. Carver A, Timperio A, Hesketh K, Crawford D. Are children and adolescents less active if parents restrict their physical activity and active transport due to perceived risk*?.* Soc Sci Med. 2010;70:1799-1805.
22. Catenacci V, Barrett C, Odgen L, Browning R, Schaefer CA, Hill J, et al. Changes in physical activity and sedentary behavior in a randomized trial of an internet-based versus workbook-based family intervention study. J Phys Act Heal. 2014;11:348-358.
23. Cerin E, Baranowski T, Barnett A, Butte N, Hughes S, Lee RE, et al. Places where preschoolers are (in)active: An observational study on Latino preschoolers and their parents using objective measures. Int J Behav Nutr Phys Act. 2016;13:29.
24. Chen J-L, Guo J, Esquivel JH, Chesla CA. Like mother, like child: The influences of maternal attitudes and behaviors on weight-related health behaviors in their children. J Transcult Nurs. 2018;29:523-531.
25. Chillon P, Hales D, Vaughn A, Gizlice Z, Ni A, Ward DS. A cross-sectional study of demographic, environmental and parental barriers to active school travel among children in the United States. Int J Behav Nutr Phys Act. 2014;11:61.
26. Cottrell L, Spangler-Murphy E, Minor V, Downes A, Nicholson P, Neal WA. A kindergarten cardiovascular risk surveillance study: CARDIAC-Kinder. Am J Health Behav. 2005;29:595-606.
27. Cutumisu N, Belanger-Gravel A, Laferte M, Lagarde F, Lemay J-F, Gauvin L. Influence of area deprivation and perceived neighbourhood safety on active transport to school among urban Quebec preadolescents. Can J Public Health. 2014;105:e376-382.
28. D’Haese S, Gheysen F, De Bourdeaudhuij I, Deforche B, Van Dyck D, Cardon G. The moderating effect of psychosocial factors in the relation between neighborhood walkability and children’s physical activity. Int J Behav Nutr Phys Act. 2016;13:128.
29. D’Haese S, Van Dyck D, De Bourdeaudhuij I, Deforche B, Cardon G. The association between the parental perception of the physical neighborhood environment and children’s location-specific physical activity. BMC Public Health. 2015;15:565.
30. Datar A, Nicosia N, Wong E, Shier V. Neighborhood environment and children’s physical activity and body mass index: Evidence from military personnel installation assignments. Child Obes. 2015;11:130-138.
31. Davison KK, Jurkowski JM, Li K, Kranz S, Lawson HA. A childhood obesity intervention developed by families for families: Results from a pilot study. Int J Behav Nutr Phys Act. 2013;10:3.
32. Davison KK, Deane GD. The consequence of encouraging girls to be active for weight loss. Soc Sci Med. 2010;70:518-525.
33. Delamater AM, Pulgaron ER, Rarback S, Hernandez J, Carrillo A, Christiansen S, et al. Web-based family intervention for overweight children: A pilot study. Child Obes. 2013;9:57-63.
34. Delaney C, Eck K, Byrd-Bredbenner C. Child physical activity propensity and parent physical activity cognitions behaviors and the home environment (P16-011-19). Curr Dev Nutr. 2019;3:nzz050.
35. Dellert JC, Johnson P. Interventions with children and parents to improve physical activity and body mass index: A meta-analysis. Am J Health Promot. 2014;28:259-267.
36. Dowda M, Pfeiffer KA, Brown WH, Mitchell JA, Byun W, Pate RR. Parental and environmental correlates of physical activity of children attending preschool. Arch Pediatr Adolesc Med. 2011;165:939-944.
37. Ducheyne F, De Bourdeaudhuij I, Spittaels H, Cardon G. Individual, social and physical environmental correlates of “never” and “always” cycling to school among 10 to 12 year old children living within a 3.0 km distance from school. Int J Behav Nutr Phys Act. 2012;9:142.
38. Dumith SC, Gigante DP, Domingues MR, Hallal PC, Menezes AMB, Kohl HW 3rd. Predictors of physical activity change during adolescence: A 3.5-year follow-up. Public Health Nutr. 2012;15:2237-2245.
39. Eichinger M, Schneider S, De Bock F. Subjectively and objectively assessed social and physical environmental correlates of preschoolers’ accelerometer-based physical activity. Int J Behav Nutr Phys Act. 2017;14:153.
40. Esteban-Cornejo I, Carlson JA, Conway TL, Cain KL, Saelens BE, Frank LD, et al. Parental and adolescent perceptions of neighborhood safety related to adolescents’ physical activity in their neighborhood. Res Q Exerc Sport. 2016;87:191-199.
41. Gainforth HL, Jarvis JW, Berry TR, Chulak-Bozzer T, Deshpande S, Faulkner G, et al. Evaluating the ParticipACTION “Think Again” campaign. Health Educ Behav. 2016;43:434-441.
42. Gerards SMPL, Dagnelie PC, Gubbels JS, van Buuren S, Hamers FJ, Jansen MW, et al. The effectiveness of lifestyle triple P in the Netherlands: A randomized controlled trial. PLoS One. 2015;10:e0122240.
43. Gross RS, Mendelsohn AL, Yin HS, Tomopoulos S, Gross MB, Scheinmann R, et al. Randomized controlled trial of an early child obesity prevention intervention: Impacts on infant tummy time. Obesity (Silver Spring). 2017;25:920-927.
44. Gunter KB, Rice KR, Ward DS, Trost SG. Factors associated with physical activity in children attending family child care homes. Prev Med (Baltim). 2012;54:131-133.
45. Haerens L, Deforche B, Maes L, Cardon G, Stevens V, De Bourdeaudhuij I. Evaluation of a 2-year physical activity and healthy eating intervention in middle school children. Health Educ Res. 2006;21:911-921.
46. Hammersley ML, Okely AD, Batterham MJ, Jones RA. An internet-based childhood obesity prevention program (Time2bHealthy) for parents of preschool-aged children: Randomized controlled trial. J Med Internet Res. 2019;21:e11964.
47. Hearst MO, Patnode CD, Sirard JR, Farbakhsh K, Lytle LA. Multilevel predictors of adolescent physical activity: A longitudinal analysis. Int J Behav Nutr Phys Act. 2012;9:8.
48. Heitzler CD, Martin SL, Duke J, Huhman M. Correlates of physical activity in a national sample of children aged 9-13 years. Prev Med (Baltim). 2006;42:254-260.
49. Hendrie GA, Coveney J, Cox DN. Defining the complexity of childhood obesity and related behaviours within the family environment using structural equation modelling. Public Health Nutr. 2012;15:48-57.
50. Herman A, Nelson BB, Teutsch C, Chung PJ. “Eat healthy, stay active!”: A coordinated intervention to improve nutrition and physical activity among Head Start parents, staff, and children. Am J Health Promot. 2012;27:e27-36.
51. Hesketh KR, Lakshman R, van Sluijs EMF. Barriers and facilitators to young children’s physical activity and sedentary behaviour: a systematic review and synthesis of qualitative literature. Obes Rev. 2017;18:987-1017.
52. Hnatiuk J, Salmon J, Campbell KJ, Ridgers ND, Hesketh KD. Early childhood predictors of toddlers’ physical activity: Longitudinal findings from the Melbourne InFANT program. Int J Behav Nutr Phys Act. 2013;10:123.
53. Hosseini SV, Anoosheh M, Abbaszadeh A, Ehsani M. Qualitative Iranian study of parents’ roles in adolescent girls’ physical activity habit development. Nurs Health Sci. 2013;15:207-212.
54. Ikeda E, Hinckson E, Witten K, Smith M. Assessment of direct and indirect associations between children active school travel and environmental, household and child factors using structural equation modelling. Int J Behav Nutr Phys Act. 2019;16:32.
55. Jalali MS, Sharafi-Avarzaman Z, Rahmandad H, Ammerman AS. Social influence in childhood obesity interventions: A systematic review. Obes Rev. 2016;17:820-832.
56. Janicke DM, Lim CS, Perri MG, Bobroff LB, Mathews AE, Brumback BA, et al. The extension family lifestyle intervention project (E-FLIP for kids): Design and methods. Contemp Clin Trials. 2011;32:50-58.
57. Janssen I, Ferrao T, King N. Individual, family, and neighborhood correlates of independent mobility among 7 to 11-year-olds. Prev Med reports. 2016;3:98-102.
58. Kalish M, Banco L, Burke G, Lapidus G. Outdoor play: A survey of parent’s perceptions of their child’s safety. J Trauma. 2010;69:218-222.
59. Kimbro RT, Brooks-Gunn J, McLanahan S. Young children in urban areas: Links among neighborhood characteristics, weight status, outdoor play, and television watching. Soc Sci Med. 2011;72:668-676.
60. Kimiecik JC, Horn TS. Parental beliefs and children’s moderate-to-vigorous physical activity. Res Q Exerc Sport. 1998;69:163-175.
61. Kitzman-Ulrich H, Wilson DK, St George SM, Lawman H, Segal M, Fairchild A. The integration of a family systems approach for understanding youth obesity, physical activity, and dietary programs. Clin Child Fam Psychol Rev. 2010;13:231-253.
62. Knowlden AP, Sharma M, Cottrell RR, Wilson BRA, Johnson ML. Impact evaluation of enabling mothers to prevent pediatric obesity through web-based education and reciprocal determinism (EMPOWER) randomized control trial. Health Educ Behav. 2015;42:171-184.
63. Knowlden A, Sharma M. One-year efficacy testing of enabling mothers to prevent pediatric obesity through web-based education and reciprocal determinism (EMPOWER) randomized control trial. Health Educ Behav. 2016;43:94-106.
64. Knowlden A, Sharma M. A Feasibility and efficacy randomized controlled trial of an online preventative program for childhood obesity: Protocol for the EMPOWER intervention. JMIR Res Protoc. 2012;1:e5.
65. Larsen H, Dinkel D, Warehime S, Berg K. The relationship between parental and child physical activity in a rural community. Fam Community Health. 2017;40:331-337.
66. Lavin Fueyo J, Totaro Garcia LM, Mamondi V, Pereira Alencar G, Florindo AA, Berra S. Neighborhood and family perceived environments associated with children’s physical activity and body mass index. Prev Med (Baltim). 2016;82:35-41.
67. Leary JM, Lilly CL, Dino G, Loprinzi PD, Cottrell L. Parental influences on 7-9 year olds’ physical activity: A conceptual model. Prev Med (Baltim). 2013;56:341-344.
68. Lee C, Zhu X, Yoon J, Varni JW. Beyond distance: Children’s school travel mode choice. Ann Behav Med. 2013;45:55-67.
69. Lee H, Tamminen KA, Clark AM, Slater L, Spence JC, Holt NL. A meta-study of qualitative research examining determinants of children’s independent active free play. Int J Behav Nutr Phys Act. 2015;12:5.
70. Leslie E, Kremer P, Toumbourou JW, Williams JW. Gender differences in personal, social and environmental influences on active travel to and from school for Australian adolescents. J Sci Med Sport. 2010;13:597-601.
71. Long DE, Gaetke LM, Perry SD, Abel MG, Clasey JL. The assessment of physical activity and nutrition in home schooled versus public schooled children. Pediatr Exerc Sci. 2010;22:44-59.
72. Loprinzi P., Cardinal BJ, Loprinzi KL, Lee H. Parenting practices as mediators of child physical activity and weight status. Obes Facts. 2012;5:420-430.
73. Loprinzi PD, Trost SG. Parental influences on physical activity behavior in preschool children. Prev Med (Baltim). 2010;50:129-133.
74. Loucaides CA, Jago R, Theophanous M. Prevalence and correlates of active traveling to school among adolescents in Cyprus. Cent Eur J Public Health. 2010;18:151-156.
75. Luk JW, Miller JM, Gilman SE, Lipsky LM, Haynie DL, Simons-Morton BG. Sexual Minority status and adolescent eating behaviors, physical activity, and weight status. Am J Prev Med. 2018;55:839-847.
76. Mah SK, Nettlefold L, Macdonald HM, Winters M, Race D, Voss C, McKay HA. Does parental support influence children’s active school travel?. Prev Med reports. 2017;6:346-351.
77. Martin-Biggers J, Spaccarotella K, Hongu N, Alleman G, Worobey J, Byrd-Bredbenner C. Translating it into real life: A qualitative study of the cognitions, barriers and supports for key obesogenic behaviors of parents of preschoolers. BMC Public Health. 2015;15:189.
78. Marzi I, Demetriou Y, Reimers AK. Social and physical environmental correlates of independent mobility in children: A systematic review taking sex/gender differences into account. Int J Health Geogr. 2018;17:24.
79. Masoumi HE. Associations of built environment and children’s physical activity: a narrative review. Rev Environ Health. 2017;32:315-331.
80. McDonald S, Dowda M, Colabianchi N, Porter D, Dishman RK, Pate RR. Perceptions of the neighborhood environment and children’s afterschool moderate-to-vigorous physical activity. Pediatr Exerc Sci. 2015;27:243-251.
81. McGuire MT, Hannan PJ, Neumark-Sztainer D, Cossrow NHF, Story M. Parental correlates of physical activity in a racially/ethnically diverse adolescent sample. J Adolesc Health. 2002;30:253-261.
82. McMurray RG, Bradley CB, Harrell JS, Bernthal PR, Frauman AC, Bangdiwala SI. Parental influences on childhood fitness and activity patterns. Res Q Exerc Sport. 1993;64:249-255.
83. Mellin AE, Neumark-Sztainer D, Story M, Ireland M, Resnick MD. Unhealthy behaviors and psychosocial difficulties among overweight adolescents: the potential impact of familial factors. J Adolesc Health. 2002;31:145-153.
84. Mendonca G, Junior JC de F. Physical activity and social support in adolescents: analysis of different types and sources of social support. J Sports Sci. 2015;33:1942-1951.
85. Motl RW, Dishman RK, Saunders RP, Dowda M, Pate RR. Perceptions of physical and social environment variables and self-efficacy as correlates of self-reported physical activity among adolescent girls. J Pediatr Psychol. 2007;32:6-12.
86. Mulhall P, Reis J, Begum S. Early adolescent participation in physical activity: Correlates with individual and family characteristics. J Phys Act Heal. 2011;8:244-252.
87. Muthuri SK, Wachira L-J, Onywera VO, Tremblay MS. Associations between parental perceptions of the neighborhood environment and childhood physical activity: Results from ISCOLE-Kenya. J Phys Act Health. 2016;13:333-343.
88. Mutz M, Albrecht P. Parents’ social status and children’s daily physical activity: The role of familial socialization and support. J Child Fam Stud. 2017;26:3026-3035.
89. Nader PR, Sallis JF, Patterson TL, Abramson IS, Rupp JW, Senn KL, et al. A family approach to cardiovascular risk reduction: Results from the San Diego family health project. Health Educ Q. 1989;16:229-244.
90. Nader PR, Sellers DE, Johnson CC, Perry CL, Stone EJ, Cook KC, et al. The effect of adult participation in a school-based family intervention to improve children’s diet and physical activity: The child and adolescent trial for cardiovascular health. Prev Med (Baltim). 1996;25:455-464.
91. Newton RLJ, Marker AM, Allen HR, Machtmes R, Han H, Johnson WD, et al. Parent-targeted mobile phone intervention to increase physical activity in sedentary children: Randomized pilot trial. JMIR mHealth uHealth. 2014;2:e48.
92. Nichols-English GJ, Lemmon CR, Litaker MS, Cartee SG, Yin Z, Gutin B, et al. Relations of black mothers’ and daughters’ body fatness, physical activity beliefs and behavior. Ethn Dis. 2006;16:172-179.
93. Nicksic NE, Salahuddin M, Butte NF, Hoelscher DM. Associations between parent-perceived neighborhood safety and encouragement and child outdoor physical activity among low-income children. J Phys Act Health. 2018;15:317-324.
94. Noonan RJ, Boddy LM, Knowles ZR, Fairclough SJ. Predisposing, reinforcing and enabling factors for physical activity in boys and girls from socially disadvantaged communities. Health Educ J. 2019;78:149-162.
95. O'Connor TM, Jago R, Baranowski T. Engaging parents to increase youth physical activity a systematic review. Am J Prev Med. 2009;37:141-149.
96. Olvera N, Smith DW, Lee C, Liu J, Lee J, Kim JH, et al. Comparing high and low acculturated mothers and physical activity in Hispanic children. J Phys Act Health. 2011;8 Suppl 2:s206-213.
97. Ostbye T, Mann CM, Vaughn AE, Namenek Brouwer RJ, Benjamin Neelon SE, Hales D, et al. The keys to healthy family child care homes intervention: Study design and rationale. Contemp Clin Trials. 2015;40:81-89.
98. Panter JR, Jones AP, van Sluijs EMF, Griffin SJ. Attitudes, social support and environmental perceptions as predictors of active commuting behaviour in school children. J Epidemiol Community Health. 2010;64:41-48.
99. Parker KE, Salmon J, Villanueva K, Mavoa S, Veitch J, Brown HL, et al. Ecological correlates of activity-related behavior typologies among adolescents. BMC public health*.* 2019;19:1041.
100. Pfeiffer KA, Dowda M, McIver KL, Pate RR. Factors related to objectively measured physical activity in preschool children. Pediatr Exerc Sci. 2009;21:196-208.
101. Prioreschi A, Brage S, Hesketh KD, Hnatiuk J, Westgate K, Micklesfield LK. Describing objectively measured physical activity levels, patterns, and correlates in a cross sectional sample of infants and toddlers from South Africa. Int J Behav Nutr Phys Act. 2017;14:176.
102. Quinlan A, Rhodes RE, Blanchard CM, Naylor P-J, Warburton DER. Family planning to promote physical activity: A randomized controlled trial protocol. BMC Public Health. 2015;15:1011.
103. Remmers T, Van Kann D, Gubbels J, Schmidt S, de Vries S, Ettema D, et al. Moderators of the longitudinal relationship between the perceived physical environment and outside play in children: The KOALA birth cohort study. Int J Behav Nutr Phys Act. 2014;11:150.
104. Rhodes RE, Blanchard CM, Quinlan A, Naylor P-J, Warburton DER. Family physical activity planning and child physical activity outcomes: A randomized trial. Am J Prev Med. 2019;57:135-144.
105. Riazi NA, Blanchette S, Trudeau F, Larouche R, Tremblay MS, Faulkner G. Correlates of children's independent mobility in Canada: A multi-site study. Int J Environ Res Public Health. 2019;16:e2862.
106. Romanella NE, Wakat DK, Loyd BH, Kelly LE. Physical activity and attitudes in lean and obese children and their mothers. Int J Obes. 1991;15:407-414.
107. Ross A, Kwon JY, Kulinna PH, Searle M. Active transportation: The role of parent attitude, the physical environment, and social capital. J Phys Act Health. 2018:1-8.
108. Rothman L, Macpherson AK, Ross T, Buliung RN. The decline in active school transportation (AST): A systematic review of the factors related to AST and changes in school transport over time in North America. Prev Med. 2018;111:314-322.
109. Sadler LS, Cowlin A. Moving into parenthood: A program for new adolescent mothers combining parent education with creative physical activity. J Spec Pediatr Nurs. 2003;8:62-70.
110. Saimon R, Choo WY, Bulgiba A. "Feeling unsafe": A photovoice analysis of factors influencing physical activity behavior among Malaysian adolescents. Asia Pac J Public Health. 2015;27:nP2079-2092.
111. Sanz-Arazuri E, Ponce-de-Leon-Elizondo A, Valdemoros-San-Emeterio MA. Parental predictors of physical inactivity in Spanish adolescents. J Sports Sci Med. 2012;11:95-101.
112. Schmutz EA, Leeger-Aschmann CS, Radtke T, Muff S, Kakebeeke TH, Zysset AE, et al. Correlates of preschool children's objectively measured physical activity and sedentary behavior: A cross-sectional analysis of the SPLASHY study. Int J Behav Nutr Phys Act. 2017;14:1.
113. Shelton D, Le Gros K, Norton L, Stanton-Cook S, Morgan J, Masterman P. Randomised controlled trial: A parent-based group education programme for overweight children. J of Paediatr and Child Health. 2007;43:799-805.
114. Simons A, Koekemoer K, Niekerk Av, Govender R. Parental supervision and discomfort with children walking to school in low-income communities in Cape Town, South Africa. Traffic Inj Prev. 2018;19:391-398.
115. Stanley RM, Boshoff K, Dollman J. A qualitative exploration of the "critical window": Factors affecting Australian children's after-school physical activity. J Phys Act Health. 2013;10:33-41.
116. Stark LJ, Filigno SS, Bolling C, Ratcliff MB, Kichler JC, Robson SL et al. Learning about Activity and Understanding Nutrition for Child Health (LAUNCH): Rationale, design, and implementation of a randomized clinical trial of a family-based pediatric weight management program for preschoolers. Contemp Clinical Trials*.* 2017;52:10-19.
117. Taylor NJ, Sahota P, Sargent J, et al. Using intervention mapping to develop a culturally appropriate intervention to prevent childhood obesity: The HAPPY (Healthy and Active Parenting Programme for Early Years) study. Int J Behav Nutr Phys Act*.* 2013;10:142.
118. Timperio A, Ball K, Salmon J, et al. Personal, family, social, and environmental correlates of active commuting to school. Am J Prev Med. 2006;30:45-51.
119. Tung SEH, Ng XH, Chin YS, Mohd Taib MN. Associations between parents' perception of neighbourhood environments and safety with physical activity of primary school children in Klang, Selangor, Malaysia. Child Care Health Dev. 2016;42:478-485.
120. Umstattd Meyer MR, Sharkey JR, Patterson MS, Dean WR. Understanding contextual barriers, supports, and opportunities for physical activity among Mexican-origin children in Texas border colonias: a descriptive study. BMC Public Health. 2013;13:14.
121. Van Der Horst K, Paw MJCA, Twisk JWR, Van Mechelen W. A brief review on correlates of physical activity and sedentariness in youth. Med Sci Sorts Exerc. 2007;39:1241-1250.
122. Vanwolleghem G, Van Dyck D, De Meester F, De Bourdeaudhuij I, Cardon G, Gheysen F. Which socio-ecological factors associate with a switch to or maintenance of active and passive transport during the transition from primary to secondary school? PloS One. 2016;11:e0156531.
123. Verloigne M, Van Lippevelde W, Maes L, Brug J, De Bourdeaudhuij I. Family- and school-based predictors of energy balance-related behaviours in children: A 6-year longitudinal study. Public Health Nutr. 2013;16:202-211.
124. Wang ZH, Dong YH, Song Y, Yang ZP, Ma J. Analysis on prevalence of physical activity time <1 hour and related factors in students aged 9-22 years in China, 2014. Zhonghua Liu Xing Bing Xue Za Zhi. 2017;38:341-345.
125. Wenthe PJ, Janz KF, Levy SM. Gender similarities and differences in factors associated with adolescent moderate-vigorous physical activity. Pediatr Exer Sci. 2009;21:291-304.
126. Wilkie HJ, Standage M, Gillison FB, Cumming SP, Katzmarzyk PT. The home electronic media environment and parental safety concerns: relationships with outdoor time after school and over the weekend among 9-11 year old children. BMC Public Health. 2018;18:456.
127. Xu H, Wen LM, Hardy LL, Rissel C. A 5-year longitudinal analysis of modifiable predictors for outdoor play and screen-time of 2- to 5-year-olds. Int J Behav Nutr Phys Act. 2016;13:96.
128. Zecevic CA, Tremblay L, Lovsin T, Michel L. Parental Influence on Young Children's Physical Activity. Int J Pediatrs. 2010;2010:468526.
129. Zhang M, Quick V, Jin Y, Martin-Biggers J. Associations of mother's behaviors and home/neighborhood environments with preschool children's physical activity behaviors. Am J Health Promot. 2019;34:83-86.
130. Zhu X, Arch B, Lee C. Personal, social, and environmental correlates of walking to school behaviors: case study in Austin, Texas. ScientificWorldJournal. 2008;8:859-872.
131. Ziviani J, Scott J, Wadley D. Walking to school: Incidental physical activity in the daily occupations of Australian children. Occup Ther Int. 2004;11:1-11.

**Parenting style (n=37)**

(e.g., authoritative, controlling/restrictive, permissive)

1. Aaltonen S, Kaprio J, Kujala UM, Pulkkinen L, Rose RJ, Silventoinen K. The interplay between genes and psychosocial home environment on physical activity. Med Sci Sports Exerc. 2018;50:691-699.
2. Arredondo EM, Elder JP, Ayala GX, Campbell N, Baquero B, Duerksen S. Is parenting style related to children’s healthy eating and physical activity in Latino families?. Health Educ Res. 2006;21:862-871.
3. Baskin ML, Dulin-Keita A, Thind H, Godsey E. Social and cultural environment factors influencing physical activity among African-American adolescents. J Adolesc Health. 2015;56:536-542.
4. Berge JM. A review of familial correlates of child and adolescent obesity: What has the 21st century taught us so far? Int J Adolesc Med Health. 2009;21:457-483.
5. Berge JM, Wall M, Loth K, Neumark-Sztainer D. Parenting style as a predictor of adolescent weight and weight-related behaviors. J Adolesc Health. 2010;46:331-338.
6. Boutelle KN, Rhee KE, Liang J, Braden A, Douglas J, Strong D, et al. Effect of attendance of the child on body weight, energy intake, and physical activity in childhood obesity treatment: A randomized clinical trial. JAMA Pediatr. 2017;171:622-628.
7. Breslin G, Shannon S, Rafferty R, Fitzpatrick B, Belton S, O’Brien W, et al. The effect of sport for LIFE: All island in children from low socio-economic status: A clustered randomized controlled trial. Health Qual Life Outcomes. 2019;17:66.
8. Brotman LM, Dawson-McClure S, Huang K, Theise R, Kamboukos D, Wang J, et al. Early childhood family intervention and long-term obesity prevention among high-risk minority youth. Pediatrics. 2012;129:e621-628.
9. Cadogan SL, Keane E, Kearney PM. The effects of individual, family and environmental factors on physical activity levels in children: A cross-sectional study. BMC Pediatr. 2014;14:107.
10. Carbert NS, Brussoni M, Geller J, Masse LC. Familial environment and overweight/obese adolescents’ physical activity. Int J Environ Res Public Health. 2019;16:e2558.
11. Hendrie GA, Coveney J, Cox DN. Defining the complexity of childhood obesity and related behaviours within the family environment using structural equation modelling. Public Health Nutr. 2012;15:48-57.
12. Hennessy E, Hughes SO, Goldberg JP, Hyatt RR, Economos CD. Parent-child interactions and objectively measured child physical activity: A cross-sectional study. Int J Behav Nutr Phys Act. 2010;7:71.
13. Hesketh KR, O’Malley C, Paes VM, Moore H, Summerbell C, Ong KK, et al. Determinants of change in physical activity in children 0-6 years of age: A systematic review of quantitative literature. Sports Med. 2017;47:1349-1374.
14. Huffman LE, Wilson DK, Van Horn ML, Pate RR. Associations between parenting factors, motivation, and physical activity in overweight African American adolescents. Ann Behav Med. 2018;52:93-105.
15. Innella N, McNaughton D, Schoeny M, Tangney C, Breitenstein S, Reed M, et al. Child temperament, maternal feeding practices, and parenting styles and their influence on obesogenic behaviors in Hispanic preschool children. J Sch Nurs. 2019;35:287-298.
16. Jago R, Davison KK, Brockman R, Page AS, Thompson JL, Fox KR. Parenting styles, parenting practices, and physical activity in 10- to 11-year olds. Prev Med. 2011;52:44-47.
17. Janssen I. Hyper-parenting is negatively associated with physical activity among 7-12 year olds. Prev Med. 2015;73:55-59.
18. Kitzman-Ulrich H, Wilson DK, St George SM, Lawman H, Segal M, Fairchild A. The integration of a family systems approach for understanding youth obesity, physical activity, and dietary programs. Clin Child Fam Psychol Rev. 2010;13:231-253.
19. Langer SL, Crain AL, Senso MM, Levy RL, Sherwood NE. Predicting child physical activity and screen time: Parental support for physical activity and general parenting styles. J Pediatr Psychol. 2014;39:633-642.
20. Lloyd AB, Lubans DR, Plotnikoff RC, Collins CE, Morgan PJ. Maternal and paternal parenting practices and their influence on children’s adiposity, screen-time, diet and physical activity. Appetite. 2014;79:149-157.
21. Loprinzi P., Cardinal BJ, Loprinzi KL, Lee H. Parenting practices as mediators of child physical activity and weight status. Obes Facts. 2012;5:420-430.
22. Morton KL, Wilson AH, Perlmutter LS, Beauchamp MR. Family leadership styles and adolescent dietary and physical activity behaviors: A cross-sectional study. Int J Behav Nutr Phys Act. 2012;9:48.
23. Pinquart M. Associations of general parenting and parent-child relationship with pediatric obesity: A meta-analysis. J Pediatr Psychol. 2014;39:381-393.
24. Rutten C, Boen F, Seghers J. The relation between environmental factors and pedometer-determined physical activity in children: the mediating role of autonomous motivation. Pediatr Exerc Sci. 2013;25:273-287.
25. Sadler LS, Cowlin A. Moving into parenthood: A program for new adolescent mothers combining parent education with creative physical activity. J Spec Pediatr Nurs. 2003;8:62-70.
26. Saunders J, Hume C, Timperio A, Salmon J. Cross-sectional and longitudinal associations between parenting style and adolescent girls' physical activity. Int J Behav Nutr Phys Act. 2012;9:141.
27. Schmitz KH, Lytle LA, Phillips GA, Murray DM, Birnbaum AS, Kubik MY. Psychosocial correlates of physical activity and sedentary leisure habits in young adolescents: the Teens Eating for Energy and Nutrition at School study. Prev Med. 2002;34:266-278.
28. Sleddens EFC, Gerards SMPL, Thijs C, De Vries NK, Kremers SPJ. General parenting, childhood overweight and obesity-inducing behaviors: A review. Int J Pediatr Obes*.* 2011;6:e12-27.
29. Smith JD, Berkel C, Jordan N, Atkins DC, Narayanan SS, Gallo C,et al. An individually tailored family-centered intervention for pediatric obesity in primary care: Study protocol of a randomized type II hybrid effectiveness-implementation trial (Raising Healthy Children study). Implem Sci. 2018;13:11.
30. St George SM, Wilson DK. A qualitative study for understanding family and peer influences on obesity-related health behaviors in low-income African-American adolescents. Child Obes. 2012;8:466-476.
31. Sterrett EM, Williams J, Thompson K, et al. An exploratory study of 2 parenting styles and family health behaviors. Am J Health Behav. 2013;37:458-468.
32. Taylor A, Wilson C, Slater A, Mohr P. Parent- and child-reported parenting: Associations with child weight-related outcomes. Appetite. 2011;57:700-706.
33. Van der Geest KE, Merelle SYM, Rodenburg G, Van de Mheen D, Renders CM. Cross-sectional associations between maternal parenting styles, physical activity and screen sedentary time in children. BMC Public Health. 2017;17:753.
34. Ward DS, Vaughn AE, Bangdiwala KI, Campbell M, Jones DJ, Panter AT, Stevens J. Integrating a family-focused approach into child obesity prevention: Rationale and design for the My Parenting SOS study randomized control trial. BMC public health. 2011;11:431.
35. Xu H, Wen LM, Rissel C. Associations of parental influences with physical activity and screen time among young children: A systematic review. J Obes. 2015;2015:546925.
36. Yaffe Y. Physical activity among Israeli-Arab adolescent males: How do parenting styles matter? Am J Men Health. 2018;12:2037-2043.
37. Zhang Y, Davey C, Larson N, Reicks M. Influence of parenting styles in the context of adolescents' energy balance-related behaviors: Findings from the FLASHE study. Appetite. 2019;142:104364.

**Parental instrumental support (n=60)**

1. Anderssen N, Wold B. Parental and peer influences on leisure-time physical activity in young adolescents. Res Q Exerc Sport. 1992;63:341-348.
2. Barr-Anderson DJ, Adams-Wynn AW, Orekoya O, Alhassan S. Socio-cultural and environmental factors that influence weight-related behaviors: Focus group results from African-American girls and their mothers. Int J Environ Res Public Health. 2018;15:e1354.
3. Baskin ML, Dulin-Keita A, Thind H, Godsey E. Social and cultural environment factors influencing physical activity among African-American adolescents. J Adolesc Health. 2015;56:536-542.
4. Beets MW, Cardinal BJ, Alderman BL. Parental social support and the physical activity-related behaviors of youth: A review. Health Educ Behav. 2010;37:621-644.
5. Best K, Ball K, Zarnowiecki D, Stanley R, Dollman J. In search of consistent predictors of children’s physical activity. Int J Environ Res Public Health. 2017;14:e1258.
6. Brunet J, Sabiston CM, O’Loughlin J, Mathieu ME, Tremblay A, Barnett TA, et al. Perceived parental social support and moderate-to-vigorous physical activity in children at risk of obesity. Res Q Exerc Sport. 2014;85:198-207.
7. Corder K, Craggs C, Jones AP, Ekelund U, Griffin SJ, van Sluijs EMF. Predictors of change differ for moderate and vigorous intensity physical activity and for weekdays and weekends: A longitudinal analysis. Int J Behav Nutr Phys Act. 2013;10:69.
8. Cottrell L, Zatezalo J, Bonasso A, Lattin J, Shawley S, Murphy E, et al. The relationship between children’s physical activity and family income in rural settings: A cross-sectional study. Prev Med Reports. 2015;2:99-104.
9. Crespo NC, Corder K, Marshall S, Norman GJ, Patrick K, Sallis JF, et al. An examination of multilevel factors that may explain gender differences in children’s physical activity. J Phys Act Health. 2013;10:982-992.
10. Davison KK, Cutting TM, Birch LL. Parents’ activity-related parenting practices predict girls’ physical activity. Med Sci Sports Exerc. 2003;35:1589-1595.
11. Davison KK, Jago R. Change in parent and peer support across ages 9 to 15 yr and adolescent girls’ physical activity. Med Sci Sports Exerc. 2009;41:1816-1825.
12. Delaney C, Eck K, Byrd-Bredbenner C. Child physical activity propensity and parent physical activity cognitions behaviors and the home environment (P16-011-19). Curr Dev Nutr. 2019;3(Suppl 1):nzz050.
13. Dowda M, Dishman RK, Pfeiffer KA, Pate RR. Family support for physical activity in girls from 8th to 12th grade in South Carolina. Prev Med. 2007;44:153-159.
14. Edwardson CL, Gorely T. Activity-related parenting practices and children’s objectively measured physical activity. Pediatr Exerc Sci. 2010;22:105-113.
15. Eisenberg ME, Larson NI, Berge JM, Thul C, Neumark-Sztainer D. The home physical activity environment and adolescent BMI, physical activity and TV viewing: Disparities across a diverse sample. J Racial Ethn Heal Disparities. 2014;1:326-336.
16. Gunter KB, Rice KR, Ward DS, Trost SG. Factors associated with physical activity in children attending family child care homes. Prev Med (Baltim). 2012;54:131-133.
17. Hearst MO, Patnode CD, Sirard JR, Farbakhsh K, Lytle LA. Multilevel predictors of adolescent physical activity: A longitudinal analysis. Int J Behav Nutr Phys Act. 2012;9:8.
18. Heredia NI, Ranjit N, Warren JL, Evans AE. Association of parental social support with energy balance-related behaviors in low-income and ethnically diverse children: A cross-sectional study. BMC Public Health. 2016;16:1182.
19. Hesketh KR, Lakshman R, van Sluijs EMF. Barriers and facilitators to young children’s physical activity and sedentary behaviour: A systematic review and synthesis of qualitative literature. Obes Rev. 2017;18:987-1017.
20. Hesketh KR, O’Malley C, Paes VM, Moore H, Summerbell C, Ong KK, et al. Determinants of change in physical activity in children 0-6 years of age: A systematic review of quantitative literature. Sports Med. 2017;47:1349-1374.
21. Hoefer WR, McKenzie TL, Sallis JF, Marshall SJ, Conway TL. Parental provision of transportation for adolescent physical activity. Am J Prev Med. 2001;21:48-51.
22. Huffman LE, Wilson DK, Van Horn ML, Pate RR. Associations between parenting factors, motivation, and physical activity in overweight African American adolescents. Ann Behav Med. 2018;52:93-105.
23. Hutchens A, Lee RE. Parenting practices and children’s physical activity: An integrative review. J Sch Nurs. 2018;34:68-85.
24. Jago R, Davison KK, Brockman R, Page AS, Thompson JL, Fox KR. Parenting styles, parenting practices, and physical activity in 10- to 11-year olds. Prev Med. 2011;52:44-47.
25. Jago R, Wood L, Sebire SJ, Edwards MJ, Davies B, Banfield K, et al. School travel mode, parenting practices and physical activity among UK year 5 and 6 children. BMC Public Health. 2014;14:370.
26. Kirk D, Carlson T, O’Connor A, Burke P, Davis K, Glover S. The economic impact on families of children’s participation in junior sport. Aust J Sci Med Sport. 1997;29:27-33.
27. Kunin-Batson AS, Seburg EM, Crain AL, Jaka MM, Langer SL, Levy RL, et al. Household factors, family behavior patterns, and adherence to dietary and physical activity guidelines among children at risk for obesity. J Nutr Educ Behav. 2015;47:206-215.
28. Laird Y, Fawkner S, Kelly P, McNamee L, Niven A. The role of social support on physical activity behaviour in adolescent girls: A systematic review and meta-analysis. Int J Behav Nutr Phys Act. 2016;13:79.
29. Lau EY, Faulkner G, Qian W, Leatherdale ST. Longitudinal associations of parental and peer influences with physical activity during adolescence: Findings from the COMPASS study. Heal Promot Chronic Dis Prev Canada Res Policy Pract. 2016;36:235-242.
30. Lau EY, Barr-Anderson DJ, Dowda M, Forthofer M, Saunders RP, Pate RR. Associations between home environment and after-school physical activity and sedentary time among 6th grade children. Pediatr Exerc Sci. 2015;27:226-233.
31. Liu Y, Zhang Y, Chen S, Zhang J, Guo Z, Chen P. Associations between parental support for physical activity and moderate-to-vigorous physical activity among Chinese school children: A cross-sectional study. J Sport Heal Sci. 2017;6:410-415.
32. Long DE, Gaetke LM, Perry SD, Abel MG, Clasey JL. The assessment of physical activity and nutrition in home schooled versus public schooled children. Pediatr Exerc Sci. 2010;22:44-59.
33. Lopez N V, Yang C-H, Belcher BR, Margolin G, Dunton GF. Within-subject associations of maternal physical activity parenting practices on children’s objectively measured moderate-to-vigorous physical activity. J Pediatr Psychol. 2019;44:300-310.
34. Lu C, Huang G, Corpeleijn E. Environmental correlates of sedentary time and physical activity in preschool children living in a relatively rural setting in the Netherlands: A cross-sectional analysis of the GECKO Drenthe cohort. BMJ Open. 2019;9:e027468.
35. Marzi I, Demetriou Y, Reimers AK. Social and physical environmental correlates of independent mobility in children: A systematic review taking sex/gender differences into account. Int J Health Geogr. 2018;17:24.
36. Masoumi HE. Associations of built environment and children’s physical activity: A narrative review. Rev Environ Health. 2017;32:315-331.
37. Mendonca G, Junior JC. Physical activity and social support in adolescents: Analysis of different types and sources of social support. J Sports Sci. 2015;33:1942-1951.
38. Niermann CYN, Gerards SMPL, Kremers SPJ. Conceptualizing family influences on children’s energy balance-related behaviors: Levels of interacting family environmental subsystems (The LIFES framework). Int J Environ Res Public Health. 2018;15:e2714.
39. O'Connor TM, Chen T-A, Baranowski J, Thompson D, Baranowski T. Physical activity and screen-media-related parenting practices have different associations with children's objectively measured physical activity. Child Obes. 2013;9:446-453.
40. Pate RR, Trost SG, Felton GM, Ward DS, Dowda M, Saunders R. Correlates of physical activity behavior in rural youth. Res Q Exer Sport. 1997;68:241-248.
41. Patnode CD, Lytle LA, Erickson DJ, Sirard JR, Barr-Anderson D, Story M. The relative influence of demographic, individual, social, and environmental factors on physical activity among boys and girls. Inter J of Behav Nutr and Phys Ac. 2010;7:79.
42. Peeters C, Marchand H, Tulloch H, et al. Perceived facilitators, barriers, and changes in a randomized exercise trial for obese youth: A qualitative inquiry. J of Phys Act Health. 2012;9:650-660.
43. Peterson MS, Lawman HG, Wilson DK, Fairchild A, Van Horn ML.The association of self-efficacy and parent social support on physical activity in male and female adolescents. Health Psychol. 2013;32:666-674.
44. Price SM, McDivitt J, Weber D, Wolff LS, Massett HA, Fulton JE. Correlates of weight-bearing physical activity among adolescent girls: Results from a national survey of girls and their parents. J of Phys Act Health. 2008;5:132-145.
45. Prochaska JJ, Rodgers MW, Sallis JF. Association of parent and peer support with adolescent physical activity. Res Q Exer Sport. 2002;73:206-210.
46. Ries AV, Voorhees CC, Gittelsohn J, Roche KM, Astone NM. Adolescents' perceptions of environmental influences on physical activity. Am J Health Behav. 2008;32:26-39.
47. Robbins LB, Stommel M, Hamel LM. Social support for physical activity of middle school students. Public Health Nurs. 2008;25:451-460.
48. Rutten C, Boen F, Seghers J. Which school- and home-based factors in elementary school-age children predict physical activity and sedentary behavior in secondary school-age children? A prospective cohort study. J Phys Act Health. 2015;12:409-417.
49. Rutten C, Boen F, Seghers J. The relation between environmental factors and pedometer-determined physical activity in children: the mediating role of autonomous motivation. Pediatr Exer Sci. 2013;25:273-287.
50. Salas C, Petermann-Rocha F, Celis-Morales C, Martinez-Lopez EJ. Parental support for physical activity in schoolchildren and its influence on nutritional status and fitness. Rev Chil Pediatr. 2018;89:732-740.
51. Sebire SJ, Jago R, Wood L, Thompson JL, Zahra J, Lawlor DA. Examining a conceptual model of parental nurturance, parenting practices and physical activity among 5-6 year olds. Soc Sci Med*.* 2016;148:18-24.
52. Siceloff ER, Wilson DK, Van Horn L. A longitudinal study of the effects of instrumental and emotional social support on physical activity in underserved adolescents in the ACT trial. Ann Behav Med. 2014;48:71-79.
53. Stucky-Ropp RC, DiLorenzo TM. Determinants of exercise in children. Prev Med. 1993;22:880-889.
54. Trost SG, Pate RR, Ward DS, Saunders R, Riner W. Correlates of objectively measured physical activity in preadolescent youth. Am J Prev Med. 1999;17:120-126.
55. Trost SG, Sirard JR, Dowda M, Pfeiffer KA, Pate RR. Physical activity in overweight and non-overweight preschool children. Inter J Obes Relat Metab Disord.2003;27:834-839.
56. Umstattd Meyer MR, Sharkey JR, Patterson MS, Dean WR. Understanding contextual barriers, supports, and opportunities for physical activity among Mexican-origin children in Texas border Colonias: A descriptive study. BMC Public Health. 2013;13:14.
57. Verloigne M, Van Lippevelde W, Maes L, Brug J, De Bourdeaudhuij I. Family- and school-based correlates of energy balance-related behaviours in 10-12-year-old children: a systematic review within the ENERGY (EuropeaN Energy balance Research to prevent excessive weight Gain among Youth) project. Public Health Nutr. 2012;15:1380-1395.
58. Verloigne M, Veitch J, Carver A, Salmon J, Cardon G, De Bourdeaudhuij I, Timperio A. Exploring associations between parental and peer variables, personal variables and physical activity among adolescents: a mediation analysis. BMC Public Health. 2014;14:966.
59. Whitehead SH, Biddle SJH, O'Donovan TM, Nevill ME. Social-psychological and physical environmental factors in groups differing by levels of physical activity: A study of Scottish adolescent girls. Pediatr Exer Sci. 2006;18:226-239.
60. Zahl-Thanem T, Steinsbekk S, Wichstrom L. Predictors of physical activity in middle childhood. A fixed-effects regression approach. Front Public Health. 2018;6:305.

**Parental monitoring (n=50)**

(i.e., monitoring of physical activity, monitoring/supervision of outdoor time)

1. Ah Hong S, Peltzer K, Wimonpeerapattana W. Impact of self-efficacy and parenting practice on physical activity among school children. Nagoya J Med Sci. 2017;79:339-349.
2. Bauer KW, Berge JM, Neumark-Sztainer D. The importance of families to adolescents’ physical activity and dietary intake. Adolesc Med State Art Rev. 2011;22:601-613.
3. Bauer KW, Neumark-Sztainer D, Fulkerson JA, Hannan PJ, Story M. Familial correlates of adolescent girls’ physical activity, television use, dietary intake, weight, and body composition. Int J Behav Nutr Phys Act. 2011;8:25.
4. Bradley RH, McRitchie S, Houts RM, Nader P, O’Brien M, NICHD Early Child Care Resarch Network. Parenting and the decline of physical activity from age 9 to 15. Int J Behav Nutr Phys Act. 2011;8:33.
5. Campbell KJ, Hesketh KD, McNaughton SA, Ball K, McCallum Z, Lynch J, et al. The extended infant feeding, activity and nutrition trial (InFANT extend) program: A cluster-randomized controlled trial of an early intervention to prevent childhood obesity. BMC Public Health. 2016;16:166.
6. Campbell KJ, Lioret S, McNaughton SA, Crawford DA, Salmon J, Ball K, et al. A parent-focused intervention to reduce infant obesity risk behaviors: A randomized trial. Pediatrics. 2013;131:652-660.
7. Campbell K, Hesketh K, Crawford D, Salmon J, Ball K, McCallum Z. The infant feeding activity and nutrition trial (INFANT) an early intervention to prevent childhood obesity: Cluster-randomised controlled trial. BMC Public Health. 2008;8:103.
8. Carver A, Panter JR, Jones AP, van Sluijs EMF. Independent mobility on the journey to school: A joint cross-sectional and prospective exploration of social and physical environmental influences. J Transp Heal. 2014;1:25-32.
9. Carver A, Timperio A, Hesketh K, Crawford D. Are children and adolescents less active if parents restrict their physical activity and active transport due to perceived risk?. Soc Sci Med. 2010;70:1799-1805.
10. Chiarlitti NA, Kolen AM. Parental influences and the relationship to their children’s physical activity levels. Int J Exerc Sci. 2017;10:205-212.
11. Cislak A, Safron M, Pratt M, Gaspar T, Luszczynska A. Family-related predictors of body weight and weight-related behaviours among children and adolescents: A systematic umbrella review. Child Care Health Dev. 2012;38:321-331.
12. Cockrell Skinner A, Perrin EM, Steiner MJ. Healthy for now? A cross-sectional study of the comorbidities in obese preschool children in the United States. Clin Pediatr. 2010;49:648-655.
13. Condessa LA, Chaves OC, Silva FM, Malta DC, Caiaffa WT. Sociocultural factors related to the physical activity in boys and girls: PeNSE 2012. Rev Saude Publica. 2019;53:25.
14. Craven MR, Keefer L, Rademaker A, Dykema-Engblade A, Sanchez-Johnsen L. Social support for exercise as a predictor of weight and physical activity status among Puerto Rican and Mexican men: Results from the Latino men’s health initiative. Am J Mens Health. 2018;12:766-778.
15. Crawford D, Cleland V, Timperio A, Salmon J, Andrianopoulos N, Roberts R, et al. The longitudinal influence of home and neighbourhood environments on children’s body mass index and physical activity over 5 years: The CLAN study. Int J Obes (Lond). 2010;34:1177-1187.
16. Delamater AM, Pulgaron ER, Rarback S, Hernandez J, Carrillo A, Christiansen S, et al. Web-based family intervention for overweight children: A pilot study. Child Obes. 2013;9:57-63.
17. Edwardson CL, Gorely T. Activity-related parenting practices and children’s objectively measured physical activity. Pediatr Exerc Sci. 2010;22:105-113.
18. Epstein LH, Paluch RA, Kilanowski CK, Raynor HA. The effect of reinforcement or stimulus control to reduce sedentary behavior in the treatment of pediatric obesity. Health Psychol. 2004;23:371-380.
19. Epstein LH, Roemmich JN, Stein RI, Paluch RA, Kilanowski CK. The challenge of identifying behavioral alternatives to food: Clinic and field studies. Ann Behav Med. 2005;30:201-209.
20. Erika KA, Nurachmah E, Rustina Y, As’ad S, Nontji W. Effect of family empowerment modified model to a family’s ability in controlling life style and physical activity of children with overweight and obesity. Pakistan J Nutr. 2016;15:737-744.
21. Gerards SM, Dagnelie PC, Gubbels JS, van Buuren S, Hamers FJ, Jansen MW, et al. The effectiveness of lifestyle triple p in the Netherlands: A randomized controlled trial. PLoS One. 2015;10:e0122240.
22. Gubbels JS, Kremers SPJ, Stafleu A, de Vries SI, Goldbohm RA, Dagnelie PC, et al. Association between parenting practices and children’s dietary intake, activity behavior and development of body mass index: The KOALA birth cohort study. Int J Behav Nutr Phys Act. 2011;8:18.
23. Henne HM, Tandon PS, Frank LD, Saelens BE. Parental factors in children’s active transport to school. Public Health. 2014;128:643-646.
24. Heredia NI, Ranjit N, Warren JL, Evans AE. Association of parental social support with energy balance-related behaviors in low-income and ethnically diverse children: A cross-sectional study. BMC Public Health. 2016;16:1182.
25. Hesketh KR, O’Malley C, Paes VM, Moore H, Summerbell C, Ong KK, et al. Determinants of change in physical activity in children 0-6 years of age: A systematic review of quantitative literature. Sports Med. 2017;47:1349-1374.
26. Lawman HG, Wilson DK. A review of family and environmental correlates of health behaviors in high-risk youth. Obesity. 2012;20:1142-1157.
27. Lawman HG, Wilson DK. Associations of social and environmental supports with sedentary behavior, light and moderate-to-vigorous physical activity in obese underserved adolescents. Int J Behav Nutr Phys Act. 2014;11:92.
28. Lee E-Y, Hesketh KD, Rhodes RE, Rinaldi CM, Spence JC, Carson V. Role of parental and environmental characteristics in toddlers’ physical activity and screen time: Bayesian analysis of structural equation models. Int J Behav Nutr Phys Act. 2018;15:17.
29. Lee H, Tamminen KA, Clark AM, Slater L, Spence JC, Holt NL. A meta-study of qualitative research examining determinants of children’s independent active free play. Int J Behav Nutr Phys Act. 2015;12:5.
30. Lindsay AC, Wasserman M, Munoz MA, Wallington SF, Greaney ML. Examining influences of parenting styles and practices on physical activity and sedentary behaviors in Latino children in the United States: Integrative review. JMIR Public Heal Surveill. 2018;4:e14.
31. Lloyd AB, Lubans DR, Plotnikoff RC, Collins CE, Morgan PJ. Maternal and paternal parenting practices and their influence on children’s adiposity, screen-time, diet and physical activity. Appetite. 2014;79:149-157.
32. Loprinzi P., Cardinal BJ, Loprinzi KL, Lee H. Parenting practices as mediators of child physical activity and weight status. Obes Facts. 2012;5:420-430.
33. McClendon ME, Umstattd Meyer MR, Ylitalo KR, Sharkey JR. Physical activity of Mexican-heritage youth during the summer and school-year: The role of parenting strategies. J Community Health. 2017;42:1102-1110.
34. McMinn AM, Griffin SJ, Jones AP, van Sluijs EMF. Family and home influences on children’s after-school and weekend physical activity. Eur J Public Health. 2013;23:805-810.
35. Mellin AE, Neumark-Sztainer D, Story M, Ireland M, Resnick MD. Unhealthy behaviors and psychosocial difficulties among overweight adolescents: The potential impact of familial factors. J Adolesc Health. 2002;31:145-153.
36. Newton RLJ, Marker AM, Allen HR, Machtmes R, Han H, Johnson WD, et al. Parent-targeted mobile phone intervention to increase physical activity in sedentary children: Randomized pilot trial. JMIR Mhealth Uhealth. 2014;2:e48.
37. O'Connor TM, Chen T-A, Baranowski J, Thompson D, Baranowski T. Physical activity and screen-media-related parenting practices have different associations with children's objectively measured physical activity. Child Obes. 2013;9:446-453.
38. Ornelas IJ, Perreira KM, Ayala GX. Parental influences on adolescent physical activity: A longitudinal study. Int J Behav Nutr Phys Act. 2007;4:3.
39. Parker KE, Salmon J, Villanueva K, et al. Ecological correlates of activity-related behavior typologies among adolescents. BMC Public Health*.* 2019;19:1041.
40. Peltzer K. Health behavior and protective factors among school children in four African countries. Int J Behav Med. 2009;16:172-180.
41. Quinlan A, Rhodes RE, Blanchard CM, Naylor P-J, Warburton DER. Family planning to promote physical activity: A randomized controlled trial protocol. BMC Public Health. 2015;15:1011.
42. Remmers T, Broeren SML, Renders CM, Hirasing RA, van Grieken A, Raat H. A longitudinal study of children's outside play using family environment and perceived physical environment as predictors. Inter J Behav Nutr Phys Act. 2014;11:76.
43. Saimon R, Choo WY, Bulgiba A. "Feeling unsafe": A photovoice analysis of factors influencing physical activity behavior among Malaysian adolescents. Asia-Pac J Public Health. 2015;27:NP2079-2092.
44. Sharma B, Chavez RC, Nam EW. Prevalence and correlates of insufficient physical activity in school adolescents in Peru. Rev Saude Publica. 2018;52:51.
45. Sleddens EFC, Kremers SPJ, Hughes SO, Cross MB, Thijs C, De Vries NK, O’Connor TM. Physical activity parenting: A systematic review of questionnaires and their associations with child activity levels. Obes Rev. 2012;13:1015-1033.
46. Sleddens EFC, Gubbels JS, Kremers SPJ, van der Plas E, Thijs C. Bidirectional associations between activity-related parenting practices, and child physical activity, sedentary screen-based behavior and body mass index: a longitudinal analysis. Int J Behav Nutr Phys Act. 2017;14:89.
47. Vancampfort D, Van Damme T, Firth J, Smith L, Stubbs B, Rosenbaum S, Hallgren M, Hagemann N, Koyanagi A. Correlates of physical activity among 142,118 adolescents aged 12-15years from 48 low- and middle-income countries. Prev Med. 2019;127:105819.
48. Vaughn AE, Hales D, Ward DS. Measuring the physical activity practices used by parents of preschool children. Med Sci Sports Exer. 2013;45:2369-2377.
49. Verloigne M, Veitch J, Carver A, Salmon J, Cardon G, De Bourdeaudhuij I, Timperio A. Exploring associations between parental and peer variables, personal variables and physical activity among adolescents: A mediation analysis. BMC Public Health. 2014;14:966.
50. Ward DS, Vaughn AE, Bangdiwala KI, Campbell M, Jones DJ, Panter AT, Stevens J. Integrating a family-focused approach into child obesity prevention: rationale and design for the My Parenting SOS study randomized control trial. BMC Public Health. 2011;11:431.

**Parental efficacy and motivation (n=43)**

1. Adkins S, Sherwood NE, Story M, Davis M. Physical activity among African-American girls: The role of parents and the home environment. Obes Res. 2004;12 Suppl:s38-45.
2. Beutum MN, Cordier R, Bundy A. Comparing activity patterns, biological, and family factors in children with and without developmental coordination disorder. Phys Occup Ther Pediatr. 2013;33:174-185.
3. Borg A, Haughton CF, Sawyer M, Lemon SC, Kane K, Pbert L, et al. Design and methods of the healthy kids & families study: A parent-focused community health worker-delivered childhood obesity prevention intervention. BMC Obes. 2019;6:19.
4. Campbell KJ, Lioret S, McNaughton SA, Crawford DA, Salmon J, Ball K, et al. A parent-focused intervention to reduce infant obesity risk behaviors: A randomized trial. Pediatrics. 2013;131:652-660.
5. Chen J-L, Guo J, Esquivel JH, Chesla CA. Like mother, like child: The influences of maternal attitudes and behaviors on weight-related health behaviors in their children. J Transcult Nurs. 2018;29:523-531.
6. De Lepeleere S, De Bourdeaudhuij I, Cardon G, Verloigne M. Do specific parenting practices and related parental self-efficacy associate with physical activity and screen time among primary schoolchildren? A cross-sectional study in Belgium. BMJ Open. 2015;5:e007209.
7. De Lepeleere S, De Bourdeaudhuij I, Cardon G, Verloigne M. The effect of an online video intervention “Movie Models” on specific parenting practices and parental self-efficacy related to children’s physical activity, screen-time and healthy diet: A quasi experimental study. BMC Public Health. 2017;17:366.
8. DiLorenzo TM, Stucky-Ropp RC, Vander Wal JS, Gotham HJ. Determinants of exercise among children. II. A longitudinal analysis. Prev Med. 1998;27:470-477.
9. Gubbels JS, Kremers SP, Stafleu A, de Vries SI, Goldbohm RA, Dagnelie PC, et al. Association between parenting practices and children’s dietary intake, activity behavior and development of body mass index: The KOALA birth cohort study. Int J Behav Nutr Phys Act. 2011;8:18.
10. Gunter KB, Rice KR, Ward DS, Trost SG. Factors associated with physical activity in children attending family child care homes. Prev Med (Baltim). 2012;54:131-133.
11. HA A, Ng JYY, Lonsdale C, Lubans DR, Ng FF. Promoting physical activity in children through family-based intervention: Protocol of the “Active 1 + FUN” randomized controlled trial. BMC Public Health. 2019;19:218.
12. Hammersley ML, Okely AD, Batterham MJ, Jones RA. An internet-based childhood obesity prevention program (Time2bHealthy) for parents of preschool-aged children: Randomized controlled trial. J Med Internet Res. 2019;21:e11964.
13. Hesketh KR, O’Malley C, Paes VM, Moore H, Summerbell C, Ong KK, et al. Determinants of change in physical activity in children 0-6 years of age: A systematic review of quantitative literature. Sports Med. 2017;47:1349-1374.
14. Hovell MF, Nichols JF, Irvin VL, Schmitz KE, Rock CL, Hofstetter CR, et al. Parent/Child training to increase preteens’ calcium, physical activity, and bone density: A controlled trial. Am J Health Promot. 2009;24:118-128.
15. Jalali MS, Sharafi-Avarzaman Z, Rahmandad H, Ammerman AS. Social influence in childhood obesity interventions: A systematic review. Obes Rev. 2016;17:820-832.
16. Kepper M, Broyles S, Scribner R, Tseng TS, Zabaleta J, Griffiths L, et al. Parental perceptions of the social environment are inversely related to constraint of adolescents’ neighborhood physical activity. Int J Environ Res Public Health. 2016;13:e1266.
17. Kimbro RT, Brooks-Gunn J, McLanahan S. Young children in urban areas: Links among neighborhood characteristics, weight status, outdoor play, and television watching. Soc Sci Med. 2011;72:668-676.
18. Kitzman-Ulrich H, Wilson DK, St George SM, Lawman H, Segal M, Fairchild A. The integration of a family systems approach for understanding youth obesity, physical activity, and dietary programs. Clin Child Fam Psychol Rev. 2010;13:231-253.
19. Knowlden AP, Sharma M, Cottrell RR, Wilson BRA, Johnson ML. Impact evaluation of enabling mothers to prevent pediatric obesity through web-based education and reciprocal determinism (EMPOWER) randomized control trial. Health Educ Behav. 2015;42:171-184.
20. Knowlden A, Sharma M. One-year efficacy testing of enabling mothers to prevent pediatric obesity through web-based education and reciprocal determinism (EMPOWER) randomized control trial. Health Educ Behav. 2016;43:94-106.
21. Knowlden A, Sharma M. A feasibility and efficacy randomized controlled trial of an online preventative program for childhood obesity: Protocol for the EMPOWER intervention. JMIR Res Protoc. 2012;1:e5.
22. Kocken PL, Schonbeck Y, Henneman L, Janssens AC, Detmar SB. Ethnic differences and parental beliefs are important for overweight prevention and management in children: A cross-sectional study in the Netherlands. BMC Public Health. 2012;12:867.
23. Lee E-Y, Hesketh KD, Rhodes RE, Rinaldi CM, Spence JC, Carson V. Role of parental and environmental characteristics in toddlers’ physical activity and screen time: Bayesian analysis of structural equation models. Int J Behav Nutr Phys Act. 2018;15:17.
24. Lee SM, Nihiser A, Strouse D, Das B, Michael S, Huhman M. Correlates of children and parents being physically active together. J Phys Act Health. 2010;7:776-783.
25. Lloyd AB, Lubans DR, Plotnikoff RC, Collins CE, Morgan PJ. Maternal and paternal parenting practices and their influence on children’s adiposity, screen-time, diet and physical activity. Appetite. 2014;79:149-157.
26. Loprinzi P., Cardinal BJ, Loprinzi KL, Lee H. Parenting practices as mediators of child physical activity and weight status. Obes Facts. 2012;5:420-430.
27. Lu W, McKyer ELJ, Lee C, Ory MG, Goodson P, Wang S. Children’s active commuting to school: An interplay of self-efficacy, social economic disadvantage, and environmental characteristics. Int J Behav Nutr Phys Act. 2015;12:29.
28. McKee MD, Deen D, Maher S, Fletcher J, Fornari A, Blank AE. Implementation of a pilot primary care lifestyle change intervention for families of pre-school children: Lessons learned. Patient Educ Couns. 2010;79:299-305.
29. Price SM, McDivitt J, Weber D, Wolff LS, Massett HA, Fulton JE. Correlates of weight-bearing physical activity among adolescent girls: Results from a national survey of girls and their parents. J Phys Act Health. 2008;5:132-145.
30. Rhodes RE, Berry T, Craig CL, Faulkner G, Latimer-Cheung A, Spence JC, Tremblay MS. Understanding parental support of child physical activity behavior. Am J of Health Behav. 2013;37:469-477.
31. Rhodes RE, Spence JC, Berry T, et al. Predicting changes across 12 months in three types of parental support behaviors and mothers' perceptions of child physical activity. Ann of Behav Med. 2015;49:853-864.
32. Robertson W, Fleming J, Kamal A, et al. Randomised controlled trial evaluating the effectiveness and cost-effectiveness of 'Families for Health', a family-based childhood obesity treatment intervention delivered in a community setting for ages 6 to 11 years. Health Technol Assess*.* 2017;21:1-180.
33. Rohde JF, Bohman B, Berglind D, et al. Cross-sectional associations between maternal self-efficacy and dietary intake and physical activity in four-year-old children of first-time Swedish mothers. Appetite. 2018;125:131-138.
34. Rutkowski EM, Connelly CD. Self-efficacy and physical activity in adolescent and parent dyads. Journal for specialists in pediatric nursing. 2012;17:51-60.
35. Sabiston CM, Crocker PRE. Exploring self-perceptions and social influences as correlates of adolescent leisure-time physical activity. J Sport Exer Psychol. 2008;30:3-22.
36. Salas C, Petermann-Rocha F, Celis-Morales C, Martinez-Lopez EJ. Parental support for physical activity in schoolchildren and its influence on nutritional status and fitness. Rev Chil Pediatr. 2018;89:732-740.
37. Smith BJ, Grunseit A, Hardy LL, King L, Wolfenden L, Milat A. Parental influences on child physical activity and screen viewing time: a population based study. BMC Public Health. 2010;10:593.
38. Solomon-Moore E, Sebire SJ, Thompson JL, Zahra J, Lawlor DA, Jago R. Are parents' motivations to exercise and intention to engage in regular family-based activity associated with both adult and child physical activity? BMJ Open Sport Exerc Med. 2016;2:e000137.
39. Spencer PR, Sanders KA, Judge DS. Determinants of objectively measured physical activity in rural East Timorese children. Am J Hum Biol: 2019;31:e23247.
40. Stark LJ, Filigno SS, Bolling C, Ratcliff MB, Kichler JC, Robson SL et al. Learning about Activity and Understanding Nutrition for Child Health (LAUNCH): Rationale, design, and implementation of a randomized clinical trial of a family-based pediatric weight management program for preschoolers. Contemp Clinical Trials*.* 2017;52:10-19.
41. Ward DS, Vaughn AE, Bangdiwala KI, Campbell M, Jones DJ, Panter AT, Stevens J. Integrating a family-focused approach into child obesity prevention: rationale and design for the My Parenting SOS study randomized control trial. BMC public health. 2011;11:431.
42. Xu H, Wen LM, Rissel C. Associations of parental influences with physical activity and screen time among young children: A systematic review. J Obes. 2015;2015:546925.
43. Zach S, Netz Y. Like mother like child: Three generations' patterns of exercise behavior. Families, Systems and Health. 2007;25:419-434.

**Family structure (n=40)**

(e.g., living in nuclear, reconstituted, or single-headed households, number of siblings)

1. Ahmed J, Mehraj V, Jeswani GK, ur Rehman S, Shah SM, Hamadeh R. Parental and school influences on physical activity levels of high school students in Hyderabad, Pakistan. J Ayub Med Coll Abbottabad. 2016;28:110-115.
2. Berge JM, Wall M, Larson N, Loth KA, Neumark-Sztainer D. Family functioning: Associations with weight status, eating behaviors, and physical activity in adolescents. J Adolesc Health. 2013;52:351-357.
3. Burchinai LG, Eppright ES. Test of the psychogenic theory of obesity for a sample of rural girls. Am J Clin Nutr. 1959;7:288-294.
4. Cadogan SL, Keane E, Kearney PM. The effects of individual, family and environmental factors on physical activity levels in children: A cross-sectional study. BMC Pediatr. 2014;14:107.
5. Christian HE, Villanueva K, Klinker CD, Knuiman MW, Divitini M, Giles-Corti B. The effect of siblings and family dog ownership on children’s independent mobility to neighbourhood destinations. Aust N Z J Public Health. 2016;40:316-318.
6. Crawford D, Cleland V, Timperio A, Salmon J, Andrianopoulos N, Roberts R. The longitudinal influence of home and neighbourhood environments on children’s body mass index and physical activity over 5 years: The CLAN study. Int J Obes. 2010;34:1177-1187.
7. Duriancik DM, Goff CR. Children of single-parent households are at a higher risk of obesity: A systematic review. J Child Health Care. 2019;23:358-369.
8. Fernandes RA, Reichert FF, Monteiro HL, Freitas Junior IF, Cardoso JR, Ronque ER, et al. Characteristics of family nucleus as correlates of regular participation in sports among adolescents. Int J Public Health. 2012;57:431-435.
9. Gomes TN, Hedeker D, Dos Santos FK, Souza M, Santos D, Pereira S, et al. Relationship between sedentariness and moderate-to-vigorous physical activity in youth: A multivariate multilevel study. Int J Environ Res Public Health. 2017;14:e148.
10. Gorely T, Atkin AJ, Biddle SJH, Marshall SJ. Family circumstance, sedentary behaviour and physical activity in adolescents living in England: Project STIL. Int J Behav Nutr Phys Act. 2009;6:33.
11. Gustafson SL, Rhodes RE. Parental correlates of physical activity in children and early adolescents. Sports Med. 2006;36:79-97.
12. Hesketh K, Graham M, Waters E. Children’s after school activity: Associations with weight status and family circumstance. Pediatr Exerc Sci. 2008;20:84-94.
13. Hopwood MJ, Farrow D, MacMahon C, Baker J. Sibling dynamics and sport expertise. Scand J Med Sci Sports. 2015;25:724-733.
14. Jaeschke L, Steinbrecher A, Luzak A, Puggina A, Aleksovska K, Buck C, et al. Socio-cultural determinants of physical activity across the life course: A “Determinants of diet and physical activity” (DEDIPAC) umbrella systematic literature review. Int J Behav Nutr Phys Act. 2017;14:173.
15. Janssen I, Ferrao T, King N. Individual, family, and neighborhood correlates of independent mobility among 7 to 11-year-olds. Prev Med Rep. 2016;3:98-102.
16. Janssen I, Levesque L, Xu F, First Nations Information Governance Centre. Correlates of physical activity among First Nations children residing in First Nations communities in Canada. Can J Public Health. 2014;105:e412-7.
17. Kalish M, Banco L, Burke G, Lapidus G. Outdoor play: A survey of parent’s perceptions of their child’s safety. J Trauma. 2010;69:s218-22.
18. Kracht CL, Sisson SB. Sibling influence on children’s objectively measured physical activity: A meta-analysis and systematic review. BMJ Open Sport Exerc Med. 2018;4:e000405.
19. Kracht CL, Sisson SB, Guseman EH, Hubbs-Tait L, Arnold SH, Graef J, et al. Difference in objectively measured physical activity and obesity in children with and without siblings. Pediatr Exerc Sci. 2019;31:348-355.
20. Langoy A, Smith ORF, Wold B, Samdal O, Haug EM. Associations between family structure and young people’s physical activity and screen time behaviors. BMC Public Health. 2019;19:433.
21. Laukkanen A, Niemisto D, Finni T, Cantell M, Korhonen E, Saakslahti A. Correlates of physical activity parenting: The skilled kids study. Scand J Med Sci Sports. 2018;28:2691-2701.
22. Lavielle-Sotomayor P, Pineda-Aquino V, Jauregui-Jimenez O, Castillo-Trejo M. [Physical activity and sedentary lifestyle: Family and socio-demographic determinants and their impact on adolescents’ health]. Rev Salud Publica (Bogota). 2014;16:161-172.
23. Levesque L, Janssen I, Xu F. Correlates of physical activity in First Nations youth residing in First Nations and northern communities in Canada. Can J Public Health. 2015;106:e29-35.
24. Lindquist CH, Reynolds KD, Goran MI. Sociocultural determinants of physical activity among children. Prev Med (Baltim).1999;29:305-312.
25. Liu GC, Wiehe SE, Aalsma MC. Associations between child and sibling levels of vigorous physical activity in low-income minority families. Int J Pediatr Adolesc Med. 2014;1:61-68.
26. Long DE, Gaetke LM, Perry SD, Abel MG, Clasey JL. The assessment of physical activity and nutrition in home schooled versus public schooled children. Pediatr Exerc Sci. 2010;22:44-59.
27. Maher JP, Ra C, O’Connor SG, Belcher BR, Leventhal A. Associations between maternal mental health and well-being and physical activity and sedentary behavior in children. J Dev Behav Pediatr. 2017;38:385-394.
28. McMinn AM, van Sluijs EMF, Nightingale CM, Griffin SJ, Cook DG, Owen CG, et al. Family and home correlates of children’s physical activity in a multi-ethnic population: The cross-sectional child heart and health study in England (CHASE). Int J Behav Nutr Phys Act. 2011;8:11.
29. McMinn AM, Griffin SJ, Jones AP, van Sluijs EMF. Family and home influences on children’s after-school and weekend physical activity. Eur J Public Health. 2013;23:805-810.
30. McVeigh JA, Norris SA, de Wet T. The relationship between socio-economic status and physical activity patterns in South African children. Acta Paediatr. 2004;93:982-988.
31. Noonan RJ, Fairclough SJ, Knowles ZR, Boddy LM. One size does not fit all: Contextualising family physical activity using a write, draw, show and tell approach. Child. 2017;4:e59.
32. Noonan RJ, Fairclough SJ, Knowles ZR, Boddy LM. One size does not fit all: Contextualising family physical activity using a write, draw, show and tell approach. Children. 2017;4:e59.
33. Pereira S, Katzmarzyk PT, Gomes TN, Souza M, Chaves RN, Santos D, et al. Resemblance in physical activity levels: The Portuguese sibling study on growth, fitness, lifestyle, and health. Am J Hum Biol. 2018. In press.
34. Pouliou T, Sera F, Griffiths L, Joshi H, Geraci M, Cortina-Borja M, et al. Environmental influences on children's physical activity. J Epidemiol Community Health. 2015;69:77-85.
35. Ramos C, Andrade R, Andrade A, Fernandes A, Costa D, Xavier C, et al . Family context and the physical activity of adolescents: Comparing differences. Rev Bras Epidemiol. 2017;20:537-548.
36. Sallis JF, Broyles SL, Frank-Spohrer G, Berry CC, Davis TB, Nader PR. Child's home environment in relation to the mother's adiposity. Int J Obes Relat Metab Disord. 1995;19:190-197.
37. Scharte M, Bolte G. Children of single mothers: Health risks and environmental stress. Gesundheitswesen. 2012;74:123-131.
38. Silva DR, Fernandes RA, Ohara D, Collings PJ, Souza MF, Tomeleri CM, et al. Correlates of sports practice, occupational and leisure-time physical activity in Brazilian adolescents. Am J Hum Biol. 2016;28:112-117.
39. Tandon PS, Zhou C, Sallis JF, Cain KL, Frank LD, Saelens BE. Home environment relationships with children's physical activity, sedentary time, and screen time by socioeconomic status. Inter J Behav Nutr Phys Act. 2012;9:88.
40. Wang L, Qi J. Association between family structure and physical activity of Chinese adolescents. BioMed Res Inter. 2016;2016:4278682.

**Parental health (n=28)**

(i.e., body mass index, maternal depression)

1. Altenburg TM, Singh AS, Te Velde S, De Bourdeaudhuij I, Lien N, Bere E, et al. Actual and perceived weight status and its association with slimming and energy-balance related behaviours in 10- to 12-year-old European children: The ENERGY-project. Pediatr Obes. 2017;12:137-145.
2. Badura P, Madarasova Geckova A, Sigmundova D, Sigmund E, van Dijk JP, Reijneveld SA. Do family environment factors play a role in adolescents’ involvement in organized activities?. J Adolesc. 2017;59:59-66.
3. Benton PM, Skouteris H, Hayden M. Does maternal psychopathology increase the risk of pre-schooler obesity? A systematic review. Appetite. 2015;87:259-282.
4. Duarte CS, Shen S, Wu P, Must A. Maternal depression and child BMI: Longitudinal findings from a US sample. Pediatr Obes. 2012;7:124-133.
5. Harrison PA, Narayan G. Differences in behavior, psychological factors, and environmental factors associated with participation in school sports and other activities in adolescence. J Sch Health. 2003;73:113-120.
6. Hiolski K, Eisenberg ME, Shlafer RJ. Youth self-reported health and their experience of parental incarceration. Fam Syst Health. 2019;37:38-45.
7. Maher JP, Ra C, O’Connor SG, Belcher BR, Leventhal A, Margolin G, et al. Associations between maternal mental health and well-being and physical activity and sedentary behavior in children. J Dev Behav Pediatr. 2017;38:385-394.
8. Baker KM, Healy S, Rice DJ, Garcia JM. Adolescent weight and health behaviors and their associations with individual, social, and parental factors. J Phys Act Health. 2018:1-6.
9. Barnes AT, Plotnikoff RC, Collins CE, Morgan PJ. Maternal correlates of objectively measured physical activity in girls. Matern Child Health J. 2015;19:2348-2357.
10. Butte NF, Gregorich SE, Tschann JM, Penilla C, Pasch LA, De Groat CL, et al. Longitudinal effects of parental, child and neighborhood factors on moderate-vigorous physical activity and sedentary time in Latino children. Int J Behav Nutr Phys Act. 2014;11:108.
11. Cadogan SL, Keane E, Kearney PM. The effects of individual, family and environmental factors on physical activity levels in children: A cross-sectional study. BMC Pediatr. 2014;14:107.
12. Drenowatz C, Erkelenz N, Wartha O, Brandstetter S, Steinacker JM. Parental characteristics have a larger effect on children’s health behaviour than their body weight. Obes Facts. 2014;7:388-398.
13. Finn K, Johannsen N, Specker B. Factors associated with physical activity in preschool children. J Pediatr. 2002;140:81-85.
14. Grund A, Krause H, Siewers M, Rieckert H, Muller MJ. [Functional, behavioral and sociodemographic characteristics of Prepubertal children with obese and non-obese parents]. Aktuel Ernahrungsmed. 2001;26:1-7.
15. Klesges RC, Eck LH, Hanson CL, Haddock CK, Klesges LM. Effects of obesity, social interactions, and physical environment on physical activity in preschoolers. Health Psychol. 1990;9:435-449.
16. McMurray RG, Berry DC, Schwartz TA, Hall EG, Neal MN, Li S, et al. Relationships of physical activity and sedentary time in obese parent-child dyads: A cross-sectional study. BMC Public Health. 2016;16:124.
17. Nichols-English GJ, Lemmon CR, Litaker MS, Cartee SG, Yin Z, Gutin B, et al. Relations of black mothers’ and daughters’ body fatness, physical activity beliefs and behavior. Ethn Dis. 2006;16:172-179.
18. Ostbye T, Krause KM, Stroo M, Lovelady CA, Evenson KR, Peterson BL, et al. Parent-focused change to prevent obesity in preschoolers: Results from the KAN-DO study. Prev Med. 2012;55:188-195..
19. Pona AA, Carlson JA, Shook RP, Dreyer Gillette ML, Davis AM. Maternal BMI change linked to child activity change in family-based behavioral interventions for pediatric weight management. Child Obes. 2019;15:371-378.
20. Salbe AD, Fontvieille AM, Pettitt DJ, Ravussin E. Maternal diabetes status does not influence energy expenditure or physical activity in 5-year-old Pima Indian children. Diabetologia. 1998;41:1157-1162.
21. Sallis JF, Patterson TL, McKenzie TL, Nader PR. Family variables and physical activity in preschool children. J Develop Behav Pediatr. 1988;9:57-61.
22. Sallis JF, Prochaska JJ, Taylor WC. A review of correlates of physical activity of children and adolescents. Med Sci Sports Exer. 2000;32:963-975.
23. Sigmund E, Sigmundova D, Badura P, Madarasova Geckova A. Health-related parental indicators and their association with healthy weight and overweight/obese children's physical activity. BMC Public Health. 2018;18:676.
24. Trost SG, Sirard JR, Dowda M, Pfeiffer KA, Pate RR. Physical activity in overweight and nonoverweight preschool children. Inter J Obes Relat Metabol Disord. 2003;27:834-839.
25. Walton K, Simpson JR, Darlington G, Haines J. Parenting stress: a cross-sectional analysis of associations with childhood obesity, physical activity, and TV viewing. BMC Pediatr. 2014;14:244.
26. Watowicz RP, Taylor CA, Eneli IU. Lifestyle behaviors of obese children following parental weight loss surgery. Obes Surg. 2013;23:173-178.
27. Williams SL, Mummery WK. Links between adolescent physical activity, body mass index, and adolescent and parent characteristics. Health Educ Behav*.* 2011;38:510-520.
28. Zaccagni L, Toselli S, Celenza F, Albertini A, Gualdi-Russo E. Sports activities in preschool children differed between those born to immigrants and native Italians. Acta Paediatrica. 2017;106:1184-1191.

**Parent-child relationship (n=21)**

(i.e., strength/quality of relationship, tension/conflictual)

1. Aaltonen S, Kaprio J, Kujala UM, Pulkkinen L, Rose RJ, Silventoinen K. The interplay between genes and psychosocial home environment on physical activity. Med Sci Sports Exerc. 2018;50:691-699.
2. Ammouri AA, Kaur H, Neuberger GB, Gajewski B, Choi WS. Correlates of exercise participation in adolescents: Populations at risk across the lifespan: Empirical studies. Public Health Nurs. 2007; 24:111-120.
3. Beets MW, Foley JT. Association of father involvement and neighborhood quality with kindergartners’ physical activity: A multilevel structural equation model. Am J Health Promot. 2008;22:195-203.
4. Bungum TJ, Vincent ML. Determinants of physical activity among female adolescents. Am J Prev Med. 1997;13:115-122.
5. Carter M, McGee R, Taylor B, Williams S. Health outcomes in adolescence: Associations with family, friends and school engagement. J Adolesc. 2007;30:51-62.
6. Davison KK, Jurkowski JM, Li K, Kranz S, Lawson HA. A childhood obesity intervention developed by families for families: Results from a pilot study. Int J Behav Nutr Phys Act. 2013;10:3.
7. Field T, Diego M, Sanders CE. Exercise is positively related to adolescents’ relationships and academics. Adolescence. 2001;36:105-110.
8. Haines J, Rifas-Shiman SL, Horton NJ, Kleinman K, Bauer KW, Davison KK, et al. Family functioning and quality of parent-adolescent relationship: Cross-sectional associations with adolescent weight-related behaviors and weight status. Int J Behav Nutr Phys Act. 2016;13:68.
9. Hargreaves DS, McVey D, Nairn A, Viner RM. Relative importance of individual and social factors in improving adolescent health. Perspect Public Health. 2013;133:122-131.
10. Hesketh KR, Lakshman R, van Sluijs EMF. Barriers and facilitators to young children’s physical activity and sedentary behaviour: A systematic review and synthesis of qualitative literature*.* Obes Rev. 2017;18:987-1017.
11. Lawman HG, Wilson DK. Associations of social and environmental supports with sedentary behavior, light and moderate-to-vigorous physical activity in obese underserved adolescents. Int J Behav Nutr Phys Act. 2014;11:92.
12. Mata J, Munsch S. [Obesity in children and adolescents: Risks, causes, and therapy from a psychological perspective]. Bundesgesundheitsblatt Gesundheitsforschung Gesundheitsschutz. 2011;54:548-554.
13. Neshteruk CD, Nezami BT, Nino-Tapias G, Davison KK, Ward DS. The influence of fathers on children’s physical activity: A review of the literature from 2009 to 2015. Prev Med (Baltim). 2017;102:12-19.
14. Niermann CYN, Gerards SMPL, Kremers SPJ. Conceptualizing family influences on children’s energy balance-related behaviors: Levels of interacting family environmental subsystems (the LIFES framework). Int J Environ Res Public Health. 2018;15:e2714.
15. Ornelas IJ, Perreira KM, Ayala GX. Parental influences on adolescent physical activity: A longitudinal study. Inter J Behav Nutr Phys Act. 2007;4:3.
16. Peltzer K. Health behavior and protective factors among school children in four African countries. Inter J Behav Medicine. 2009;16:172-180.
17. Pinquart M. Associations of general parenting and parent-child relationship with pediatric obesity: A meta-analysis. J Pediatr Psychol. 2014;39:381-393.
18. Sebire SJ, Jago R, Wood L, Thompson JL, Zahra J, Lawlor DA. Examining a conceptual model of parental nurturance, parenting practices and physical activity among 5-6 year olds. Soc Sci Med. 2016;148:18-24.
19. Shennar-Golan V, Walter O. Physical activity intensity among adolescents and association with parent-adolescent relationship and well-being. Am J Mens Health*.* 2018;12:1530-1540.
20. Vander Ploeg KA, Kuhle S, Maximova K, McGavock J, Wu B, Veugelers PJ. The importance of parental beliefs and support for pedometer-measured physical activity on school days and weekend days among Canadian children. BMC public health. 2013;13:1132.
21. Vander Ploeg KA, Maximova K, Kuhle S, Simen-Kapeu A, Veugelers PJ. The importance of parental beliefs and support for physical activity and body weights of children: a population-based analysis. Can J Public Health. 2012;103:e277-281.

**Family environment (n=15)**

(i.e., chaotic/disorganized family, connection/cohesiveness, family stress)

1. Atkin AJ, Corder K, Goodyer I, Bamber I, Ekelund U, Brage S, et al. Perceived family functioning and friendship quality: Cross-sectional associations with physical activity and sedentary behaviours. Int J Behav Nutr Phys Act. 2015;12:23.
2. Baskind MJ, Taveras EM, Gerber MW, Fiechtner L, Horan C, Sharifi M. Parent-perceived stress and its association with children’s weight and obesity-related behaviors. Prev Chronic Dis. 2019;16:e39.
3. Bigman G, Rajesh V, Koehly LM, Strong LL, Oluyomi AO, Strom SS et al. Family cohesion and moderate-to-vigorous physical activity among Mexican origin adolescents: A longitudinal perspective. J Phys Act Health. 2015;12:1023-1030.
4. Cabrera NJ, Hofferth SL, Chae S. Patterns and predictors of father-infant engagement across race/ethnic groups. Early Child Res Q. 2011;26:365-375.
5. Carbert NS, Brussoni M, Geller J, Masse LC. Familial environment and overweight/obese adolescents’ physical activity. Int J Environ Res Public Health. 2019;16:e2558.
6. Haines J, Rifas-Shiman SL, Horton NJ, Kleinman K, Bauer KW, Davison KK, et al. Family functioning and quality of parent-adolescent relationship: Cross-sectional associations with adolescent weight-related behaviors and weight status. Int J Behav Nutr Phys Act. 2016;13:68.
7. Lavielle-Sotomayor P, Pineda-Aquino V, Jauregui-Jimenez O, Castillo-Trejo M. [Physical activity and sedentary lifestyle: Family and socio-demographic determinants and their impact on adolescents’ health]. Rev Salud Publica (Bogota). 2014;16:161-172.
8. Lebron CN, Lee TK, Park SE, St George SM, Messiah SE, Prado G. Effects of parent-adolescent reported family functioning discrepancy on physical activity and diet among Hispanic youth. J Fam Psychol. 2018;32:333-342.
9. Loprinzi PD. Association of family functioning on youth physical activity and sedentary behavior. J Phys Act Health. 2015;12:642-648
10. Mellin AE, Neumark-Sztainer D, Story M, Ireland M, Resnick MD. Unhealthy behaviors and psychosocial difficulties among overweight adolescents: The potential impact of familial factors. J Adolesc Health. 2002;31:145-153.
11. Niermann CYN, Gerards SMPL, Kremers SPJ. Conceptualizing family influences on children’s energy balance-related behaviors: Levels of interacting family environmental subsystems (the LIFES framework). Int J Environ Res Public Health. 2018;15:e2714.
12. Ornelas IJ, Perreira KM, Ayala GX. Parental influences on adolescent physical activity: A longitudinal study. Inter J of Behav Nutr Phys Act. 2007;4:3.
13. Ramanathan S, Crocker PRE. The influence of family and culture on physical activity among female adolescents from the Indian diaspora. Qual Health Res. 2009;19:492-503.
14. Salvy S-J, Miles JNV, Shih RA, Tucker JS, D'Amico EJ. Neighborhood, family and peer-level predictors of obesity-related health behaviors among young adolescents. J Pediatr Psychol. 2017;42:153-161.
15. Spink KS, Chad K, Muhajarine N, Humber L, Odnokon P, Gryba C et al. Intrapersonal correlates of sufficiently active youth and adolescents. Pediatr Exer Sci. 2005;17:124-135.
