# Supplementary material for: Development of a consensus statement on the role of the family in the physical activity, sedentary, and sleep behaviours of children and youth
Source: Int J Behav Nutr Phys Act. 2020 Jun 16;17:74. doi: 10.1186/s12966-020-00973-0 (PMC7296673; doi:10.1186/s12966-020-00973-0)
Supplement: Supplementary file 4 — Additional file 4. Review #2 (docx.). Search Process for Family and Sedentary Behaviour Literature Review (review #2). Themes from the sedentary behaviour literature review (review #2). References for the papers included in the family and sedentary behaviour review (review #2), organized by theme. [file 12966_2020_973_MOESM4_ESM.docx]

Records identified through databases searching
(n = 10,239)

Records after duplicates removed
(n = 6,808)

Records screened
(n = 313)

Identification

Eligibility

Screening

Records excluded

(n = 6,307)

**Search Process for Family and Sedentary Behaviour Literature Review (review #2).**

**Themes from the sedentary behaviour literature review (review #2).**

| **Theme** | **Topics Included** | **Number of studies** |
| --- | --- | --- |
| Parental behaviours | Parental sedentary behaviour/screen time  Co-viewing  Co-physical activity  Parental e-discipline (rewarding or punishing through screen time) | 104 |
| Sociodemographic factors | Parental age  Parental education  Parental income  Parental employment status  Race/ethnicity | 92 |
| Household practices | Sedentary behaviour/screen time limits  Number of TVs in household  Presence of screen in child’s bedroom  Watching TV during meals  Frequency of family dinners  Family routines | 84 |
| Parenting style | Authoritative  Controlling/restrictive  Permissive  Uninvolved | 55 |
| Parental beliefs, attitudes, knowledge | Self-efficacy to limit sedentary behaviour/screen time  Attitudes towards screen-based behaviours  Perceived neighborhood safety  Perceived distance from home to green space | 48 |
| Family environment | Chaotic/disorganized family  Familial connection/cohesiveness  Family stress  Stable/regular family routines | 27 |
| Parental support | Support/encouragement | 23 |
| Parental health | Mental health (e.g., depression, anxiety)  General health  Body mass index | 14 |
| Parent-child relationship | Strength/quality of relationship | 13 |
| Family structure | Single-parent home  Number of siblings  Age of siblings | 18 |

**References for the papers included in the family and sedentary behaviour review (review #2), organized by theme.**

**Parental behaviours (n=104)**

(e.g., parents’ sedentary time/screen time, co-viewing, co-physical activity)

1. Hammersley ML, Okely AD, Batterham MJ, Jones RA. An internet-based childhood obesity prevention program (time2bhealthy) for parents of preschool-aged children: randomized controlled trial. J Med Internet Re*s*. 2019;21:e11964.
2. Hesketh KR, Lakshman R, van Sluijs EMF. Barriers and facilitators to young children’s physical activity and sedentary behaviour: a systematic review and synthesis of qualitative literature. Obes Rev. 2017;18:987-1017.
3. Fernandez-Alvira JM, Te Velde SJ, Singh A, et al. Parental modeling, education and children’s sports and TV time: the ENERGY-project. Prev Med (Baltim). 2015;70:96-101.
4. Fuemmeler BF, Anderson CB, Masse LC. Parent-child relationship of directly measured physical activity. Int J Behav Nutr Phys Act. 2011;8:17.
5. Garriguet D, Colley R, Bushnik T. Parent-child association in physical activity and sedentary behaviour. Heal Reports. 2017;28:3-12.
6. Goh SN, Teh LH, Tay WR, et al. Sociodemographic, home environment and parental influences on total and device-specific screen viewing in children aged 2 years and below: an observational study. BMJ Open. 2016;6:e009113.
7. Goncalves WSF, Byrne R, Viana MT, Trost SG. Parental influences on screen time and weight status among preschool children from Brazil: a cross-sectional study. Int J Behav Nutr Phys Act. 2019;16:27.
8. Hawi NS, Rupert MS. Impact of e-discipline on children’s screen time. Cyberpsychol Behav Soc Netw. 2015;18:337-342.
9. He M, Harris S, Piche L, Beynon C. Understanding screen-related sedentary behavior and its contributing factors among school-aged children: a social-ecologic exploration. Am J Health Promot. 23;5:299-308.
10. He M, Piche L, Beynon C, Harris S. Screen-related sedentary behaviors: children’s and parents’ attitudes, motivations, and practices. J Nutr Educ Behav. 2010;42:17-25.
11. Hinkley T, Salmon J, Okely AD, Crawford D. The correlates of preschoolers’ compliance with screen recommendations exist across multiple domains. Prev Med (Baltim). 57;3:212-219.
12. Hnatiuk JA, Hesketh KR, van Sluijs EMF. Correlates of home and neighbourhood-based physical activity in UK 3-4-year-old children. Eur J Public Health. 2016;26:947-953.
13. Hoyos Cillero I, Jago R, Sebire S. Individual and social predictors of screen-viewing among Spanish school children. Eur J Pediatr. 170;1:93-102.
14. Huang WY, Wong SH, Salmon J. Correlates of physical activity and screen-based behaviors in Chinese children. J Sci Med Sport. 2013;16:509-514.
15. Hume C, van der Horst K, Brug J, Salmon J, Oenema A. Understanding the correlates of adolescents’ TV viewing: a social ecological approach. Int J Pediatr Obes. 2010;2:161-168.
16. Jago R, Edwards MJ, Urbanski CR, Sebire SJ. General and specific approaches to media parenting: a systematic review of current measures, associations with screen-viewing, and measurement implications. Child Obes. 2013;9:51-72.
17. Jago R, Fox KR, Page AS, Brockman R, Thompson JL. Parent and child physical activity and sedentary time: do active parents foster active children?. BMC Public Health. 2010;10:194.
18. Jago R, Sebire SJ, Edwards MJ, Thompson JL. Parental TV viewing, parental self-efficacy, media equipment and TV viewing among preschool children. Eur J Pediatr. 2013;172:1543-1545.
19. Jago R, Sebire SJ, Lucas PJ, et al. Parental modelling, media equipment and screen-viewing among young children: cross-sectional study. BMJ Open. 2013;3:e002593.
20. Jago R, Solomon-Moore E, Macdonald-Wallis C, Thompson JL, Lawlor DA, Sebire SJ. Association of parents’ and children’s physical activity and sedentary time in Year 4 (8-9) and change between Year 1 (5-6) and Year 4: a longitudinal study. Int J Behav Nutr Phys Act. 2017;14:110.
21. Jago R, Thompson JL, Sebire SJ, et al. Cross-sectional associations between the screen-time of parents and young children: differences by parent and child gender and day of the week. Int J Behav Nutr Phys Act. 2014;11:54.
22. Jiang X-X, Hardy LL, Ding D, Baur LA, Shi H-J. Recreational screen-time among Chinese adolescents: a cross-sectional study. J Epidemiol. 2014;24:397-403.
23. Johnson L, Chen T-A, Hughes SO, O’Connor TM. The association of parent’s outcome expectations for child TV viewing with parenting practices and child TV viewing: an examination using path analysis. Int J Behav Nutr Phys Act*.* 2015;12:70.
24. Kennedy CM. Television and young Hispanic children’s health behaviors. Pediatr Nurs. 2000;26:283-284.
25. Hardy LL, Baur LA, Garnett SP, et al. Family and home correlates of television viewing in 12-13 year old adolescents: The Nepean Study. Int J Behav Nutr Phys Act. 2006;3:24.
26. Langlois J, Omorou AY, Vuillemin A, Briancon S, Lecomte E, Group PT. Association of socioeconomic, school-related and family factors and physical activity and sedentary behaviour among adolescents: multilevel analysis of the PRALIMAP trial inclusion data. BMC Public Health. 2017;17:175.
27. Latomme J, Van Stappen V, Cardon G, et al. The association between children’s and parents’ co-TV viewing and their total screen time in six European countries: Cross-Sectional Data from the Feel4diabetes-Study. Int J Environ Res Public Health. 2018;15:2599.
28. Lee E-Y, Hesketh KD, Rhodes RE, Rinaldi CM, Spence JC, Carson V. Role of parental and environmental characteristics in toddlers’ physical activity and screen time: Bayesian analysis of structural equation models. Int J Behav Nutr Phys Act. 2018;15:17
29. Lee S-J, Chae Y-G. Children’s internet use in a family context: influence on family relationships and parental mediation. Cyberpsychol Behav. 2007;10(5):640-644
30. Saleem M, Hasaan A, Mahmood T, Mushtaq S, Bhatti A, Azam M. Factors associated with excessive TV viewing in school children of Wah Cantt, Pakistan. Rawal Med J. 2014;39:323-326.
31. Tu AW, Watts AW, Masse LC. Parent–adolescent patterns of physical activity, sedentary behaviors and sleep among a sample of overweight and obese adolescents. J Phys Act Heal. 2015;12:1469-1476.
32. Cabanas-Sánchez V, García-Cervantes L, Esteban-Gonzalo L, Girela-Rejón MJ, Castro-Piñero J, Veiga, ÓL. Social correlates of sedentary behavior in young people: The UP&DOWN study. J Sport Heal Sci. 2019;9:189-196.
33. van Ekris E, Solomon-Moore E, Chinapaw MJM, Jago R, Altenburg TM. Child- and parent-related correlates of total and prolonged sedentary time in 5- to 6-year-old children. Int J Environ Res Public Health. 2018;15:1817.
34. Van Lippevelde W, Bere E, Verloigne M, et al. The role of family-related factors in the effects of the UP4FUN school-based family-focused intervention targeting screen time in 10- to 12-year-old children: the ENERGY project. BMC Public Health. 2014;14:857.
35. Vaughn AE, Hales D, Ward DS. Measuring the physical activity practices used by parents of preschool children. Med Sci Sports Exerc. 2017;45:2369-2377.
36. Verloigne M, Van Lippevelde W, Bere E, et al. Individual and family environmental correlates of television and computer time in 10- to 12-year-old European children: the ENERGY-project. BMC Public Health. 2015;15:912.
37. Wagner A, Klein-Platat C, Arveiler D, Haan MC, Schlienger JL, Simon C. Parent-child physical activity relationships in 12-year old French students do not depend on family socioeconomic status. Diabetes Metab. 2004;30:359-366.
38. Wang X, Liu Q-M, Ren Y-J, Lv J, Li L-M. Family influences on physical activity and sedentary behaviours in Chinese junior high school students: a cross-sectional study. BMC Public Health. 2015;15:287.
39. Wickel E. Variables associated with active and inactive behavior during the after-school period. Pediatr Exerc Sci. 2013;25:288-299.
40. Wiseman N, Harris N, Downes M. Preschool children’s preferences for sedentary activity relates to parent’s restrictive rules around active outdoor play. BMC Public Health. 2019;19:946.
41. Xu C, Quan M, Zhang H, Zhou C, Chen P. Impact of parents’ physical activity on preschool children’s physical activity: a cross-sectional study. PeerJ. 2018;6:e4405.
42. Xu H, Wen LM, Rissel C. Associations of parental influences with physical activity and screen time among young children: a systematic review. J Obes. 2015. In press.
43. Xu H, Wen LM, Rissel C. Associations of maternal influences with outdoor play and screen time of two-year-olds: Findings from the Healthy Beginnings Trial. J Paediatr Child Health. 2014;50:680-686.
44. Yamada M, Sekine M, Tatsuse T. Parental Internet Use and Lifestyle Factors as Correlates of Prolonged Screen Time of Children in Japan: Results From the Super Shokuiku School Project. J Epidemiol. 2018;28:407-413.
45. Zhang M, Quick V, Jin Y, Martin-Biggers J. Associations of mother’s behaviors and home/neighborhood environments with preschool children’s physical activity behaviors. Am J Health Promot. 2019:34;83-86.
46. Bounova A, Michalopoulou M, Agelousis N, Kourtessis T, Gourgoulis V. The parental role in adolescent screen related sedentary behavior. Int J Adolesc Med Health. 2016;30:31.
47. Springer AE, Kelder SH, Hoelscher DM. Social support, physical activity and sedentary behavior among 6th-grade girls: A cross-sectional study. Int J Behav Nutr Phys Act. 2006;3:8.
48. Abbott G, Hnatiuk J, Timperio A, Salmon J, Best K, Hesketh KD. Cross-sectional and Longitudinal Associations Between Parents’ and Preschoolers’ Physical Activity and Television Viewing: The HAPPY Study. J Phys Act Health. 2016;13:269-274.
49. Asplund KM, Kair LR, Arain YH, Cervantes M, Oreskovic NM, Zuckerman KE. Early childhood screen time and parental attitudes toward child television viewing in a low-income latino population attending the special supplemental nutrition program for women, infants, and children. Child Obes. 2015;11:590-599.
50. Atkin AJ, Corder K, Ekelund U, Wijndaele K, Griffin SJ, van Sluijs EMF. Determinants of change in children’s sedentary time. PLoS One. 2013;8:e67627.
51. Barber SE, Kelly B, Collings PJ, Nagy L, Bywater T, Wright J. Prevalence, trajectories, and determinants of television viewing time in an ethnically diverse sample of young children from the UK. Int J Behav Nutr Phys Act. 2017;14:88.
52. Barradas DT, Fulton JE, Blanck HM, Huhman M. Parental influences on youth television viewing. *J Pediatr*. 2007;151:364-369.
53. Barr-Anderson DJ, Fulkerson JA, Smyth M, et al. Associations of American Indian children’s screen-time behavior with parental television behavior, parental perceptions of children’s screen time, and media-related resources in the home. Prev Chronic Dis. 2011;8:A105.
54. Bauer KW, Neumark-Sztainer D, Fulkerson JA, Hannan PJ, Story M. Familial correlates of adolescent girls’ physical activity, television use, dietary intake, weight, and body composition. Int J Behav Nutr Phys Act. 2011;8:25.
55. Bernard JY, Padmapriya N, Chen B, et al. Predictors of screen viewing time in young Singaporean children: the GUSTO cohort. Int J Behav Nutr Phys Act. 2017;14:112.
56. Bleakley A, Jordan AB, Hennessy M. The relationship between parents’ and children’s television viewing. Pediatrics. 2013;132:364-371
57. Brindova D, Pavelka J, Sevcikova A, et al. How parents can affect excessive spending of time on screen-based activities. BMC Public Health. 2014;14:1261.
58. Busschaert C, Ridgers ND, De Bourdeaudhuij I, Cardon G, Van Cauwenberg J, De Cocker K. Socio-Demographic, Social-Cognitive, Health-Related and Physical Environmental Variables Associated with Context-Specific Sitting Time in Belgian Adolescents: A One-Year Follow-Up Study. PLoS One. 2016;11:e0167553.
59. Cameron AJ, Crawford DA, Salmon J, et al. Clustering of obesity-related risk behaviors in children and their mothers. Ann Epidemiol. 2011;21:95-102.
60. Carson V, Janssen I. Associations between factors within the home setting and screen time among children aged 0-5 years: a cross-sectional study. BMC Public Health. 2012;12:539.
61. Carson V, Stearns J, Janssen I. The Relationship Between Parental Physical Activity and Screen Time Behaviors and the Behaviors of their Young Children. Pediatr Exerc Sci. 2015;27:390-395.
62. Chen J-L, Guo J, Esquivel JH, Chesla CA. Like Mother, Like Child: The Influences of Maternal Attitudes and Behaviors on Weight-Related Health Behaviors in Their Children. J Transcult Nurs. 2018:29:523-531.
63. Chiu Y-C, Li Y-F, Wu W-C, Chiang T-L. The amount of television that infants and their parents watched influenced children’s viewing habits when they got older. Acta Paediatr. 2017;106:984-990.
64. Christofaro DGD, Turi-Lynch BC, Lynch KR, et al. Parents’ Lifestyle, Sedentary Behavior, and Physical Activity in Their Children: A Cross-Sectional Study in Brazil. J Phys Act Health. 2019;16:631-636.
65. Davison KK, Francis LA, Birch LL. Links between parents’ and girls’ television viewing behaviors: a longitudinal examination. J Pediatr. 2005;147:436-442.
66. De Decker E, Hesketh K, De Craemer M, et al. Parental Influences on Preschoolers’ TV Viewing Time: Mediation Analyses on Australian and Belgian Data. J Phys Act Health. 12:1272-1279.
67. De Lepeleere S, De Bourdeaudhuij I, Cardon G, Verloigne M. Do specific parenting practices and related parental self-efficacy associate with physical activity and screen time among primary schoolchildren? A cross-sectional study in Belgium. BMJ Open. 2015;5:e007209.
68. De Lepeleere S, De Bourdeaudhuij I, Van Stappen V, et al. Parenting Practices as a Mediator in the Association Between Family Socio-Economic Status and Screen-Time in Primary Schoolchildren: A Feel4Diabetes Study. Int J Environ Res Public Health. 2018;15:2553.
69. Dearth-Wesley T, Gordon-Larsen P, Adair LS, Zhang B, Popkin BM. Longitudinal, cross-cohort comparison of physical activity patterns in Chinese mothers and children. Int J Behav Nutr Phys Act. 2012;9:39.
70. Drenowatz C, Erkelenz N, Wartha O, Brandstetter S, Steinacker JM, Group U-IS. Parental characteristics have a larger effect on children’s health behaviour than their body weight. Obes Facts. 2014;7:388-398.
71. Duch H, Fisher EM, Ensari I, Harrington A. Screen time use in children under 3 years old: a systematic review of correlates. Int J Behav Nutr Phys Act. 2013;10:102.
72. Dunton GF, Liao Y, Almanza E, et al. Joint physical activity and sedentary behavior in parent-child pairs. Med Sci Sports Exerc. 2012;44:1473-1480.
73. Eichinger M, Schneider S, De Bock F. Subjectively and Objectively Assessed Behavioral, Social, and Physical Environmental Correlates of Sedentary Behavior in Preschoolers. J Pediatr. 2018;199:71-78.
74. Epstein JA. The role of parents and related factors on adolescent computer use. J Public health Res. 2012;1:75-78.
75. Dong F, Howard AG, Herring AH, et al. Parent-child associations for changes in diet, screen time, and physical activity across two decades in modernizing China: China Health and Nutrition Survey 1991-2009. Int J Behav Nutr Phys Act. 2016;13:118.
76. Maatta S, Kaukonen R, Vepsalainen H, et al. The mediating role of the home environment in relation to parental educational level and preschool children’s screen time: a cross-sectional study. BMC Public Health. 2017;17:688.
77. Maatta S, Ray C, Vepsalainen H, et al. Parental Education and Pre-School Children’s Objectively Measured Sedentary Time: The Role of Co-Participation in Physical Activity. Int J Environ Res Public Health. 2018;15:366.
78. Marsh S, Foley LS, Wilks DC, Maddison R. Family-based interventions for reducing sedentary time in youth: a systematic review of randomized controlled trials. Obes Rev. 2014;15:117-133.
79. Matarma T, Koski P, Loyttyniemi E, Lagstrom H. The factors associated with toddlers’ screen time change in the STEPS Study: A two-year follow-up. Prev Med (Baltim). 2016;84:27-33.
80. McGuire MT, Hannan PJ, Neumark-Sztainer D, Cossrow NHF, Story M. Parental correlates of physical activity in a racially/ethnically diverse adolescent sample. J Adolesc Health. 2002;30:253-261.
81. McMurray RG, Berry DC, Schwartz TA, et al. Relationships of physical activity and sedentary time in obese parent-child dyads: a cross-sectional study. BMC Public Health. 2015;16:124.
82. Morowatisharifabad MA, Karimi M, Ghorbanzadeh F. Watching television by kids: How much and why?. J Educ Health Promot. 2015:4:36.
83. Pearson N, Biddle SJH, Griffiths P, Johnston JP, Haycraft E. Clustering and correlates of screen-time and eating behaviours among young children. BMC Public Health. 2018;18:753.
84. Raudsepp L, Riso E-M. Longitudinal Associations Between Sedentary Behavior of Adolescent Girls, Their Mothers, and Best Friends. Pediatr Exerc Sci. 2017;29:419-426.
85. Ravikiran SR, Baliga BS, Jain A, Kotian MS. Factors influencing the television viewing practices of Indian children. Indian J Pediatr. 2014;8:114-119.
86. Rosenkranz RR, Bauer A, Dzewaltowski DA. Mother-daughter resemblance in BMI and obesity-related behaviors. Int J Adolesc Med Health. 2010;22:477-489.
87. Ruiz R, Gesell SB, Buchowski MS, Lambert W, Barkin SL. The relationship between hispanic parents and their preschool-aged children’s physical activity. Pediatrics. 2011;127:888-895.
88. Rutten C, Boen F, Seghers J. Which school- and home-based factors in elementary school-age children predict physical activity and sedentary behavior in secondary school-age children? A prospective cohort study. J Phys Act Health. 2015;12:409-417.
89. Yalçin SS, TuĞrul B, Naçar N, Tuncer M, Yurdakök K. Factors that affect television viewing time in preschool and primary schoolchildren. Pediatr Int. 2002;44:622-627.
90. Salmon J, Tremblay MS, Marshall SJ, Hume C. Health risks, correlates, and interventions to reduce sedentary behavior in young people. Am J Prev Med. 2011;41:197-206.
91. Schoeppe S, Vandelanotte C, Bere E, et al. The influence of parental modelling on children’s physical activity and screen time: Does it differ by gender? Eur J Public Health. 2017;27:152-157.
92. Shaban LH, Vaccaro JA, Sukhram SD, Huffman FG. Do mothers affect daughter’s behaviors? Diet, physical activity, and sedentary behaviors in Kuwaiti mother-daughter dyads. Ecol Food Nutr. 2018;57:109-123.
93. Sigmund E, Badura P, Vokacova J, Sigmundova D. Parent-Child Relationship of Pedometer-Assessed Physical Activity and Proxy-Reported Screen Time in Czech Families with Preschoolers. Int J Environ Res Public Health. 2016;13:740.
94. Sigmundova D, Badura P, Sigmund E, Bucksch J. Weekday-weekend variations in mother-/father-child physical activity and screen time relationship: A cross-sectional study in a random sample of Czech families with 5- to 12-year-old children. Eur J Sport Sci. 2018;18:1158-1167.
95. Skouteris H, McCabe M, Swinburn B, Newgreen V, Sacher P, Chadwick P. Parental influence and obesity prevention in pre-schoolers: a systematic review of interventions. Obes Rev. 2011;12:315-328.
96. Sleddens EFC, Gubbels JS, Kremers SPJ, van der Plas E, Thijs C. Bidirectional associations between activity-related parenting practices, and child physical activity, sedentary screen-based behavior and body mass index: a longitudinal analysis. Int J Behav Nutr Phys Act. 2017;14:89.
97. Solomon-Moore E, Sebire SJ, Macdonald-Wallis C, Thompson JL, Lawlor DA, Jago R. Exploring parents’ screen-viewing behaviours and sedentary time in association with their attitudes toward their young child’s screen-viewing. Prev Med reports. 2017;7:198-205.
98. Sonneville KR, Rifas-Shiman SL, Kleinman KP, Gortmaker SL, Gillman MW, Taveras EM. Associations of obesogenic behaviors in mothers and obese children participating in a randomized trial. Obesity (Silver Spring). 2012;20:1449-1454.
99. Tanaka C, Okuda M, Tanaka M, Inoue S, Tanaka S. Associations of Physical Activity and Sedentary Time in Primary School Children with Their Parental Behaviors and Supports. Int J Environ Res Public Health. 2018;15:1995.
100. Te Velde SJ, ChinAPaw MJM, De Bourdeaudhuij I, et al. Parents and friends both matter: simultaneous and interactive influences of parents and friends on European schoolchildren’s energy balance-related behaviours - the ENERGY cross-sectional study. Int J Behav Nutr Phys Act. 2014;11:82.
101. Te Velde SJ, van der Horst K, Oenema A, Timperio A, Crawford D, Brug J. Parental and home influences on adolescents’ TV viewing: a mediation analysis. Int J Pediatr Obes. 2011;6:e364-e372.
102. Terras MM, Ramsay J. Family Digital Literacy Practices and Children’s Mobile Phone Use. Front Psychol. 2016;7:1957.
103. Thompson AL, Adair LS, Bentley ME. Maternal characteristics and perception of temperament associated with infant TV exposure. Pediatrics. 2013;131:e390-7.
104. Totland TH, Bjelland M, Lien N, et al. Adolescents’ prospective screen time by gender and parental education, the mediation of parental influences. Int J Behav Nutr Phys Act. 2013;10:89.

**Sociodemographic factors (n=92)**

(e.g., socioeconomic status, parental education, parental employment/profession)

1. Gorely T, Atkin AJ, Biddle SJH, Marshall SJ. Family circumstance, sedentary behaviour and physical activity in adolescents living in England: Project STIL. Int J Behav Nutr Phys Act. 2009;6:33.
2. Abedini Y, Zamani BE, Kheradmand A, Rajabizadeh G. Impacts of mothers’ occupation status and parenting styles on levels of self-control, addiction to computer games, and educational progress of adolescents. Addict Heal. 2012;4:102-110.
3. Aishworiya R, Cai S, Chen HY, et al. Television viewing and child cognition in a longitudinal birth cohort in Singapore: the role of maternal factors. BMC Pediatr. 2019;19:286.
4. Alamian A, Paradis G. Clustering of chronic disease behavioral risk factors in Canadian children and adolescents. Prev Med (Baltim). 2009;48:493-499.
5. Anand V, Downs SM, Bauer NS, Carroll AE. Prevalence of infant television viewing and maternal depression symptoms. J Dev Behav Pediatr. 2014;35:216-224.
6. Anastassea-Vlachou K, Fryssira-Kanioura H, Papathanasiou-Klontza D, Xipolita-Zachariadi A, Matsaniotis N. The effects of television viewing in Greece, and the role of the paediatrician: a familiar triangle revisited. Eur J Pediatr. 1996;155:1057-1060.
7. Anderson PM. Parental employment, family routines and childhood obesity. Econ Hum Biol. 2012;10:340-351.
8. Appelhans BM, Fitzpatrick SL, Li H, et al. The home environment and childhood obesity in low-income households: indirect effects via sleep duration and screen time. BMC Public Health. 2014;14:1160.
9. Asplund KM, Kair LR, Arain YH, Cervantes M, Oreskovic NM, Zuckerman KE. Early childhood screen time and parental attitudes toward child television viewing in a low-income Latino population attending the special supplemental nutrition program for women, infants, and children. Child Obes. 2015;11:590-599.
10. Augustine JM, Prickett KC, Kimbro R. Health-related parenting among U.S. families and young children’s physical health. J Marriage Fam. 2017;79:816-832.
11. Aznar S, Lara MT, Queralt A, Molina-Garcia J. Psychosocial and environmental correlates of sedentary behaviors in Spanish children. Biomed Res Int. 2017;2017:4728924.
12. Ford BS, McDonald TE, Owens AS, Robinson TN. Primary care interventions to reduce television viewing in African-American children. Am J Prev Med. 2002;22:106-109.
13. Babey SH, Hastert TA, Wolstein J. Adolescent sedentary behaviors: correlates differ for television viewing and computer use. J Adolesc Health. 2013;52:70-76.
14. Ball K, Cleland VJ, Timperio AF, Salmon J, Crawford DA. Socioeconomic position and children’s physical activity and sedentary behaviors: longitudinal findings from the CLAN study. J Phys Act Health. 2009;6:289-298.
15. Barber SE, Kelly B, Collings PJ, Nagy L, Bywater T, Wright J. Prevalence, trajectories, and determinants of television viewing time in an ethnically diverse sample of young children from the UK. Int J Behav Nutr Phys Act. 2017;14:88.
16. Berge JM, MacLehose RF, Loth KA, Eisenberg ME, Fulkerson JA, Neumark-Sztainer D. Parent-adolescent conversations about eating, physical activity and weight: prevalence across sociodemographic characteristics and associations with adolescent weight and weight-related behaviors. J Behav Med. 2015;38:122-135.
17. Bernard JY, Padmapriya N, Chen B, et al. Predictors of screen viewing time in young Singaporean children: the GUSTO cohort. Int J Behav Nutr Phys Act. 2017;14:112.
18. Brown JE, Broom DH, Nicholson JM, Bittman M. Do working mothers raise couch potato kids? Maternal employment and children’s lifestyle behaviours and weight in early childhood. Soc Sci Med. 2010;70:1816-1824.
19. Brug J, van Stralen MM, Chinapaw MJM, et al. Differences in weight status and energy-balance related behaviours according to ethnic background among adolescents in seven countries in Europe: the ENERGY-project. Pediatr Obes. 2012;7:399-411.
20. Brug J, Uijtdewilligen L, van Stralen MM, et al. Differences in beliefs and home environments regarding energy balance behaviors according to parental education and ethnicity among schoolchildren in Europe: the ENERGY cross sectional study. BMC Public Health. 2014;14:610.
21. Butte NF, Gregorich SE, Tschann JM, et al. Longitudinal effects of parental, child and neighborhood factors on moderate-vigorous physical activity and sedentary time in Latino children. Int J Behav Nutr Phys Act. 2014;11:108.
22. Vereecken CA, Maes L, De Bacquer D. The influence of parental occupation and the pupils’ educational level on lifestyle behaviors among adolescents in Belgium. J Adolesc Heal. 2004;34:330-338.
23. Carson V, Iannotti RJ, Pickett W, Janssen I. Urban and rural differences in sedentary behavior among American and Canadian youth. Health Place. 2011;17:920-928.
24. Carson V, Janssen I. Associations between factors within the home setting and screen time among children aged 0-5 years: a cross-sectional study. BMC Public Health. 2012;12:539.
25. Carson V, Kuzik N. Demographic correlates of screen time and objectively measured sedentary time and physical activity among toddlers: a cross-sectional study. BMC Public Health. 2017;17:187.
26. Cespedes EM, McDonald J, Haines J, Bottino CJ, Schmidt ME, Taveras EM. Obesity-related behaviors of US- and non-US-born parents and children in low-income households. J Dev Behav Pediatr. 2013;34:541-548.
27. Chiu Y-C, Li Y-F, Wu W-C, Chiang T-L. The amount of television that infants and their parents watched influenced children’s viewing habits when they got older. Acta Paediatr. 2017;106:984-990.
28. Christakis DA, Ebel BE, Rivara FP, Zimmerman FJ. Television, video, and computer game usage in children under 11 years of age. J Pediatr. 2004;145:652-656.
29. Coyne SM, Padilla-Walker LM, Holmgren HG. A Six-Year Longitudinal Study of Texting Trajectories During Adolescence. Child Dev. 2018;89:58-65.
30. De Craemer M, Verloigne M, Ghekiere A, et al. Changes in children’s television and computer time according to parental education, parental income and ethnicity: A 6-year longitudinal EYHS study. PLoS One. 2011;13:e0203592.
31. De Lepeleere S, De Bourdeaudhuij I, Van Stappen V, et al. Parenting Practices as a Mediator in the Association Between Family Socio-Economic Status and Screen-Time in Primary Schoolchildren: A Feel4Diabetes Study. Int J Environ Res Public Health. 2018;15:2553.
32. Downing KL, Hinkley T, Hesketh KD. Associations of Parental Rules and Socioeconomic Position With Preschool Children’s Sedentary Behaviour and Screen Time. J Phys Act Health. 2015;12:515-521.
33. Duch H, Fisher EM, Ensari I, Harrington A. Screen time use in children under 3 years old: a systematic review of correlates. Int J Behav Nutr Phys Act. 2013;10:102.
34. E. S. Parental education and living environmental influence on physical development, nutritional habits as well as level of physical activity in Polish children and adolescents. Anthropol Anzeiger. 2010;68:53-66.
35. Eisenberg ME, Larson NI, Berge JM, Thul C, Neumark-Sztainer D. The home physical activity environment and adolescent BMI, physical activity and TV viewing: Disparities across a diverse sample. J racial Ethn Heal disparities. 2014;1:326-336.
36. Fernandez-Alvira JM, De Bourdeaudhuij I, Singh AS, et al. Clustering of energy balance-related behaviors and parental education in European children: the ENERGY-project. Int J Behav Nutr Phys Act. 2013;10:5.
37. Maatta S, Kaukonen R, Vepsalainen H, et al. The mediating role of the home environment in relation to parental educational level and preschool children’s screen time: a cross-sectional study. BMC Public Health. 2017;17:688.
38. Maatta S, Ray C, Vepsalainen H, et al. Parental Education and Pre-School Children’s Objectively Measured Sedentary Time: The Role of Co-Participation in Physical Activity. Int J Environ Res Public Health. 2018;15:366.
39. MacLeod KE, Gee GC, Crawford P, Wang MC. Neighbourhood environment as a predictor of television watching among girls. J Epidemiol Community Health. 2008;62:288-292.
40. Martin MA, Lippert AM, Chandler KD, Lemmon M. Does mothers’ employment affect adolescents’ weight and activity levels? Improving our empirical estimates. SSM - Popul Heal. 2018;4:291-300.
41. Matarma T, Koski P, Loyttyniemi E, Lagstrom H. The factors associated with toddlers’ screen time change in the STEPS Study: A two-year follow-up. Prev Med (Baltim). 2016;84:27-33.
42. McGuire MT, Hannan PJ, Neumark-Sztainer D, Cossrow NHF, Story M. Parental correlates of physical activity in a racially/ethnically diverse adolescent sample. J Adolesc Health. 2002;30:253-261.
43. McVeigh JA, Norris SA, de Wet T. The relationship between socio-economic status and physical activity patterns in South African children. Acta Paediatr. 2004;93:982-988.
44. Min J, Xue H, Wang Y. Association between household poverty dynamics and childhood overweight risk and health behaviours in the United States: a 8-year nationally representative longitudinal study of 16 800 children. Pediatr Obes. 2018;13:590-597.
45. Morowatisharifabad MA, Karimi M, Ghorbanzadeh F. Watching television by kids: How much and why?. J Educ Health Promot. 2015;4:36.
46. Mullan K. Young people’s time use and maternal employment in the UK. Br J Sociol. 2009;60:741-762.
47. Munoz-Miralles R, Ortega-Gonzalez R, Lopez-Moron MR, et al. The problematic use of Information and Communication Technologies (ICT) in adolescents by the cross sectional JOITIC study. BMC Pediatr. 2016;16:140.
48. Mushtaq MU, Gull S, Mushtaq K, Shahid U, Shad MA, Akram J. Dietary behaviors, physical activity and sedentary lifestyle associated with overweight and obesity, and their socio-demographic correlates, among Pakistani primary school children. Int J Behav Nutr Phys Act. 2011;8:130.
49. Patriarca A, Di Giuseppe G, Albano L, Marinelli P, Angelillo IF. Use of television, videogames, and computer among children and adolescents in Italy. BMC Public Health. 2009;9:139.
50. Pearson N, Biddle SJH, Griffiths P, Johnston JP, Haycraft E. Clustering and correlates of screen-time and eating behaviours among young children. BMC Public Health. 2018;18:753.
51. Peltzer K, Pengpid S. Leisure Time Physical Inactivity and Sedentary Behaviour and Lifestyle Correlates among Students Aged 13-15 in the Association of Southeast Asian Nations (ASEAN) Member States, 2007-2013. Int J Environ Res Public Health. 2016;13:217.
52. Peneau S, Salanave B, Rolland-Cachera M-F, Hercberg S, Castetbon K. Correlates of sedentary behavior in 7 to 9-year-old French children are dependent on maternal weight status. Int J Obes (Lond). 2011;35:907-915.
53. Poulain T, Vogel M, Sobek C, Hilbert A, Korner A, Kiess W. Associations Between Socio-Economic Status and Child Health: Findings of a Large German Cohort Study. Int J Environ Res Public Health. 2019;16:677.
54. Ravikiran SR, Baliga BS, Jain A, Kotian MS. Factors influencing the television viewing practices of Indian children. Indian J Pediatr. 2014;81:114-119.
55. Rey-Lopez JP, Tomas C, Vicente-Rodriguez G, et al. Sedentary behaviours and socio-economic status in Spanish adolescents: the AVENA study. Eur J Public Health. 2011;21:151-157.
56. Ribner A, Fitzpatrick C, Blair C. Family Socioeconomic Status Moderates Associations Between Television Viewing and School Readiness Skills. J Dev Behav Pediatr. 2017;38:233-239
57. Richter M, Vereecken CA, Boyce W, Maes L, Gabhainn SN, Currie CE. Parental occupation, family affluence and adolescent health behaviour in 28 countries. Int J Public Health. 2009;54:203-212.
58. Rutten C, Boen F, Seghers J. Which school- and home-based factors in elementary school-age children predict physical activity and sedentary behavior in secondary school-age children? A prospective cohort study. J Phys Act Health. 2015;12:409-417.
59. Salmon J, Timperio A, Telford A, Carver A, Crawford D. Association of family environment with children’s television viewing and with low level of physical activity. Obes Res. 2005;13:1939-1951.
60. Salmon J, Tremblay MS, Marshall SJ, Hume C. Health risks, correlates, and interventions to reduce sedentary behavior in young people. Am J Prev Med. 2011;41:197-206.
61. Schmitz KH, Lytle LA, Phillips GA, Murray DM, Birnbaum AS, Kubik MY. Psychosocial correlates of physical activity and sedentary leisure habits in young adolescents: the Teens Eating for Energy and Nutrition at School study. Prev Med (Baltim). 2002;34:266-278.
62. Shahraki-Sanavi F, Rakhshani F, Ansari-Moghaddam A, Mohammadi M, Feizabad AK. Prevalence of health-risk behaviors among teen girls in Southeastern Iran. Electron physician. 2018;10:6988-6996.
63. Smith BJ, Grunseit A, Hardy LL, King L, Wolfenden L, Milat A. Parental influences on child physical activity and screen viewing time: a population based study. BMC Public Health. 2010;10:593.
64. Tandon PS, Zhou C, Sallis JF, Cain KL, Frank LD, Saelens BE. Home environment relationships with children’s physical activity, sedentary time, and screen time by socioeconomic status. Int J Behav Nutr Phys Act. 2012;9:88.
65. Thompson AL, Adair LS, Bentley ME. Maternal characteristics and perception of temperament associated with infant TV exposure. Pediatrics. 2013;131:e390-397.
66. Thompson DA, Matson PA, Ellen JM. Television viewing in low-income latino children: variation by ethnic subgroup and English proficiency. Child Obes. 2013;9:22-28.
67. Thompson DA, Sibinga EMS, Jennings JM, Bair-Merritt MH, Christakis DA. Television viewing by young Hispanic children: evidence of heterogeneity. Arch Pediatr Adolesc Med. 2010;164:174-179.
68. Fernandez-Alvira JM, Te Velde SJ, Singh A, et al. Parental modeling, education and children’s sports and TV time: the ENERGY-project. Prev Med (Baltim). 2015;70:96-101.
69. Mielke GI, Brown WJ, Ekelund U, et al. Socioeconomic position and sedentary behavior in Brazilian adolescents: A life-course approach. Prev Med (Baltim). 2018;107:29-35.
70. Gebremariam MK, Altenburg TM, Lakerveld J, et al. Associations between socioeconomic position and correlates of sedentary behaviour among youth: a systematic review. Obes Rev. 2015;16:988-1000.
71. Gomes TN, Hedeker D, Dos Santos FK, et al. Relationship between Sedentariness and Moderate-to-Vigorous Physical Activity in Youth: A Multivariate Multilevel Study. Int J Environ Res Public Health. 2017;14:148.
72. He M, Harris S, Piche L, Beynon C. Understanding screen-related sedentary behavior and its contributing factors among school-aged children: a social-ecologic exploration. Am J Health Promot. 2009;23:299-308.
73. Heshmat R, Qorbani M, Mozaffarian N, et al. Socioeconomic inequality in screen time frequency in children and adolescents: the weight disorders survey of the CASPIAN IV study. World J Pediatr. 2018;14:66-76.
74. Hesketh K, Ball K, Crawford D, Campbell K, Salmon J. Mediators of the relationship between maternal education and children’s TV viewing. Am J Prev Med. 2007;33:41-47.
75. Howe AS, Heath A-LM, Lawrence J, et al. Parenting style and family type, but not child temperament, are associated with television viewing time in children at two years of age. PLoS One. 2017;12:e0188558.
76. Hoyos Cillero I, Jago R. Sociodemographic and home environment predictors of screen viewing among Spanish school children. J Public Health (Oxf). 2011;33:392-402.
77. Hume C, van der Horst K, Brug J, Salmon J, Oenema A. Understanding the correlates of adolescents’ TV viewing: a social ecological approach. Int J Pediatr Obes. 2010;5:161-168.
78. Inyang I, Benke G, Dimitriadis C, Simpson P, McKenzie R, Abramson M. Predictors of mobile telephone use and exposure analysis in Australian adolescents. J Paediatr Child Health. 2010;46:226-233.
79. Jiang X-X, Hardy LL, Ding D, Baur LA, Shi H-J. Recreational screen-time among Chinese adolescents: a cross-sectional study. J Epidemiol. 2014;24:397-403.
80. Kim HS, Ham OK, Jang MN, Yun HJ, Park J. Economic differences in risk factors for obesity among overweight and obese children. J Sch Nurs. 2014;30:281-291.
81. Kumanyika S, Grier S. Targeting interventions for ethnic minority and low-income populations. Futur Child. 2006;16:187-207.
82. Lagerberg D, Magnusson M, Sundelin C. Child health and maternal stress: does neighbourhood status matter?. Int J Adolesc Med Health. 2011;23:19-25.
83. Langlois J, Omorou AY, Vuillemin A, Briancon S, Lecomte E, Group PT. Association of socioeconomic, school-related and family factors and physical activity and sedentary behaviour among adolescents: multilevel analysis of the PRALIMAP trial inclusion data. BMC Public Health. 2017;17:175.
84. Loprinzi PD, Schary DP, Cardinal BJ. Adherence to active play and electronic media guidelines in preschool children: gender and parental education considerations. Matern Child Health J. 2013;17:56-61.
85. Lowry R, Kann L, Collins JL, Kolbe LJ. The effect of socioeconomic status on chronic disease risk behaviors among US adolescents. JAMA. 1996;276:792-797.
86. Lundahl A, Nelson TD, Van Dyk TR, West T. Psychosocial stressors and health behaviors: examining sleep, sedentary behaviors, and physical activity in a low-income pediatric sample. Clin Pediatr (Phila). 2013;52:721-729.
87. Saleem M, Hasaan A, Mahmood T, Mushtaq S, Bhatti A, Azam M. Factors associated with excessive TV viewing in school children of Wah Cantt, Pakistan. Rawal Med J. 2014;39:323-326.
88. Van der Geest KE, Merelle SYM, Rodenburg G, Van de Mheen D, Renders CM. Cross-sectional associations between maternal parenting styles, physical activity and screen sedentary time in children. BMC Public Health. 2017;17:753.
89. Van Der Horst K, Paw MJCA, Twisk JWR, Van Mechelen W. A brief review on correlates of physical activity and sedentariness in youth. Med Sci Sports Exerc. 2007;39:1241-1250.
90. Villagran Perez S, Novalbos-Ruiz JP, Rodriguez-Martin A, Martinez-Nieto JM, Lechuga-Sancho AM. Implications of family socioeconomic level on risk behaviors in child-youth obesity. Nutr Hosp. 2013;28:1951-1960.
91. Whitley E, Gale CR, Deary IJ, Kivimaki M, Singh-Manoux A, Batty GD. Influence of maternal and paternal IQ on offspring health and health behaviours: evidence for some trans-generational associations using the 1958 British birth cohort study. Eur Psychiatry. 2013;28:219-224.
92. King AC, Parkinson KN, Adamson AJ, et al. Correlates of objectively measured physical activity and sedentary behaviour in English children. Eur J Public Health. 2011;21:424-431

**Household practices (n=84)**

(e.g., sedentary behaviour/screen time limits, number of televisions in household, presence of screen in bedroom)

1. Goh SN, Teh LH, Tay WR, et al. Sociodemographic, home environment and parental influences on total and device-specific screen viewing in children aged 2 years and below: an observational study. BMJ Open. 2016;6:e009113.
2. He M, Piche L, Beynon C, Harris S. Screen-related sedentary behaviors: children’s and parents’ attitudes, motivations, and practices. J Nutr Educ Behav. 2010;42:17-25.
3. Herman KM, Sabiston CM, Mathieu M-E, Tremblay A, Paradis G. Correlates of sedentary behaviour in 8- to 10-year-old children at elevated risk for obesity. Appl Physiol Nutr Metab. 2015;40:10-19.
4. Hesketh K, Ball K, Crawford D, Campbell K, Salmon J. Mediators of the relationship between maternal education and children’s TV viewing. Am J Prev Med. 2007;33:41-47.
5. Hohepa M, Scragg R, Schofield G, Kolt GS, Schaaf D. Associations between after-school physical activity, television use, and parental strategies in a sample of New Zealand adolescents. J Phys Act Health. 2009;6:299-305.
6. Howe AS, Heath A-LM, Lawrence J, et al. Parenting style and family type, but not child temperament, are associated with television viewing time in children at two years of age. PLoS One. 2017;12:e0188558.
7. Hoyos Cillero I, Jago R. Sociodemographic and home environment predictors of screen viewing among Spanish school children. J Public Health (Oxf). 2011;33:392-402.
8. Hoyos Cillero I, Jago R, Sebire S. Individual and social predictors of screen-viewing among Spanish school children. Eur J Pediatr. 2011;170:93-102.
9. Haines J, McDonald J, O’Brien A, et al. Healthy habits, happy homes: Randomized trial to improve household routines for obesity prevention among preschool-aged children. JAMA Pediatr. 2013;167:1072-1079.
10. Jago R, Davison KK, Thompson JL, Page AS, Brockman R, Fox KR. Parental sedentary restriction, maternal parenting style, and television viewing among 10- to 11-year-olds. Pediatrics. 2011;128:e572-e578.
11. Jago R, Edwards MJ, Urbanski CR, Sebire SJ. General and specific approaches to media parenting: a systematic review of current measures, associations with screen-viewing, and measurement implications. Child Obes. 2013;9:S51-72.
12. Jago R, Page A, Froberg K, Sardinha LB, Klasson-Heggebø L, Andersen LB. Screen-viewing and the home TV environment: The European Youth Heart Study. Prev Med (Baltim). 2008;47:525-529.
13. Jiang X-X, Hardy LL, Ding D, Baur LA, Shi H-J. Recreational screen-time among Chinese adolescents: a cross-sectional study. J Epidemiol. 2014;24:397-403.
14. Johnson L, Chen T-A, Hughes SO, O’Connor TM. The association of parent’s outcome expectations for child TV viewing with parenting practices and child TV viewing: an examination using path analysis. Int J Behav Nutr Phys Act. 2015;12:70.
15. Kesten JM, Sebire SJ, Turner KM, Stewart-Brown S, Bentley G, Jago R. Associations between rule-based parenting practices and child screen viewing: A cross-sectional study. Prev Med reports. 2015;2:84-89.
16. Kim KW, Wallander JL, Felt JM, Elliott MN, Schuster MA. Associations of Parental General Monitoring with Adolescent Weight-Related Behaviors and Weight Status. Obesity (Silver Spring). 2019;27:280-287.
17. Koning IM, Peeters M, Finkenauer C, van den Eijnden RJJM. Bidirectional effects of Internet-specific parenting practices and compulsive social media and Internet game use. J Behav Addict. 2018;7:624-632.
18. Hardy LL, Baur LA, Garnett SP, et al. Family and home correlates of television viewing in 12-13 year old adolescents: The Nepean Study. Int J Behav Nutr Phys Act. 2006;3:24.
19. Lampard AM, Jurkowski JM, Davison KK. The family context of low-income parents who restrict child screen time. Child Obes. 2013;9:386-392.
20. Lederer AM, King MH, Sovinski D, Kim N. The Impact of Family Rules on Children’s Eating Habits, Sedentary Behaviors, and Weight Status. Child Obes. 2015;11:421-429.
21. Lee E-Y, Hesketh KD, Rhodes RE, Rinaldi CM, Spence JC, Carson V. Role of parental and environmental characteristics in toddlers’ physical activity and screen time: Bayesian analysis of structural equation models. Int J Behav Nutr Phys Act. 2018;15:17.
22. Lee S-J, Bartolic S, Vandewater EA. Predicting children’s media use in the USA: differences in cross-sectional and longitudinal analysis. Br J Dev Psychol. 2009;27:123-143.
23. Lee S-J, Chae Y-G. Children’s Internet use in a family context: influence on family relationships and parental mediation. Cyberpsychol Behav. 2007;10:640-644.
24. Li M, Xue H, Wang W, Wang Y. Parental Expectations and Child Screen and Academic Sedentary Behaviors in China. Am J Prev Med. 2017;52:680-689.
25. Lloyd AB, Lubans DR, Plotnikoff RC, Collins CE, Morgan PJ. Maternal and paternal parenting practices and their influence on children’s adiposity, screen-time, diet and physical activity. Appetite. 2014;79:149-157.
26. Ran M, Wang H, Peng LL, He F, Yang JW. Study on recent sedentary behaviors and influencing factors among children and adolescents in Chongqing area. J Shanghai Jiaotong Univ (Medical Sci). 2018;38:95-100.
27. Saleem M, Hassan A, Mahmood T, Mushtaq S, Bhatti A, Azam M. Factors associated with excessive TV viewing in school children of Wah Cantt, Pakistan. Rawal Med J. 2014;39:323-326.
28. Van Lippevelde W, Bere E, Verloigne M, et al. The role of family-related factors in the effects of the UP4FUN school-based family-focused intervention targeting screen time in 10- to 12-year-old children: the ENERGY project. BMC Public Health. 2014;14:857.
29. van Zutphen M, Bell AC, Kremer PJ, Swinburn BA. Association between the family environment and television viewing in Australian children. J Paediatr Child Health. 2007;43:458-463.
30. Vaughn AE, Hales D, Ward DS. Measuring the physical activity practices used by parents of preschool children. Med Sci Sports Exerc. 2013;45:2369-2377.
31. Veldhuis L, van Grieken A, Renders CM, Hirasing RA, Raat H. Parenting style, the home environment, and screen time of 5-year-old children; the “be active, eat right” study. PLoS One. 9:e88486.
32. Verloigne M, Van Lippevelde W, Bere E, et al. Individual and family environmental correlates of television and computer time in 10- to 12-year-old European children: the ENERGY-project. BMC Public Health. 2015;15:912.
33. Verloigne M, Van Lippevelde W, Maes L, Brug J, De Bourdeaudhuij I. Family- and school-based correlates of energy balance-related behaviours in 10-12-year-old children: a systematic review within the ENERGY (EuropeaN Energy balance Research to prevent excessive weight Gain among Youth) project. Public Health Nutr. 2012;15:1380-1395.
34. Wiseman N, Harris N, Downes M. Preschool children’s preferences for sedentary activity relates to parent’s restrictive rules around active outdoor play. BMC Public Health. 2019;19:946.
35. Xu H, Wen LM, Rissel C. Associations of maternal influences with outdoor play and screen time of two-year-olds: Findings from the Healthy Beginnings Trial. J Paediatr Child Health. 2014;50:680-686.
36. Yamada M, Sekine M, Tatsuse T. Parental Internet Use and Lifestyle Factors as Correlates of Prolonged Screen Time of Children in Japan: Results From the Super Shokuiku School Project. J Epidemiol. 2018;28:407-413.
37. He M, Harris S, Piche L, Beynon C. Understanding screen-related sedentary behavior and its contributing factors among school-aged children: a social-ecologic exploration. Am J Health Promot. 2009;23:299-308.
38. Hesketh K, Ball K, Crawford D, Campbell K, Salmon J. Mediators of the relationship between maternal education and children’s TV viewing. Am J Prev Med. 2007;33:41-47.
39. Hoyos Cillero I, Jago R. Sociodemographic and home environment predictors of screen viewing among Spanish school children. J Public Health (Oxf). 2011;33:392-402.
40. Huang WY, Wong SH, Salmon J. Correlates of physical activity and screen-based behaviors in Chinese children. J Sci Med Sport. 2013;16:509-514.
41. Haines J, McDonald J, O’Brien A, et al. Healthy habits, happy homes: Randomized trial to improve household routines for obesity prevention among preschool-aged children. JAMA Pediatr. 2013;167:1072-1079.
42. Robinson JL, Winiewicz DD, Fuerch JH, Roemmich JN, Epstein LH. Relationship between parental estimate and an objective measure of child television watching. Int J Behav Nutr Phys Act. 2006;3:43.
43. Jago R, Sebire SJ, Lucas PJ, et al. Parental modelling, media equipment and screen-viewing among young children: cross-sectional study. BMJ Open. 2013;3:e002593.
44. Johnson L, Chen T-A, Hughes SO, O’Connor TM. The association of parent’s outcome expectations for child TV viewing with parenting practices and child TV viewing: an examination using path analysis. Int J Behav Nutr Phys Act. 2015;12:70.
45. Hardy LL, Baur LA, Garnett SP, et al. Family and home correlates of television viewing in 12-13 year old adolescents: The Nepean Study. Int J Behav Nutr Phys Act. 2006;3:24.
46. Lau EY, Barr-Anderson DJ, Dowda M, Forthofer M, Saunders RP, Pate RR. Associations Between Home Environment and After-School Physical Activity and Sedentary Time Among 6th Grade Children. Pediatr Exerc Sci. 2015;27:226-233.
47. LeBlanc AG, Katzmarzyk PT, Barreira T V, et al. Correlates of Total Sedentary Time and Screen Time in 9-11 Year-Old Children around the World: The International Study of Childhood Obesity, Lifestyle and the Environment. PLoS One. 2006;10:e0129622.
48. Lee E-Y, Hesketh KD, Rhodes RE, Rinaldi CM, Spence JC, Carson V. Role of parental and environmental characteristics in toddlers’ physical activity and screen time: Bayesian analysis of structural equation models. Int J Behav Nutr Phys Act. 2017;15:17.
49. Ran M, Wang H, Peng L-L, He F, Yang J-W. Study on recent sedentary behaviors and influencing factors among children and adolescents in Chongqing area. J Shanghai Jiaotong Univ (Medical Sci). 2018;38:95-100.
50. Van Lippevelde W, Bere E, Verloigne M, et al. The role of family-related factors in the effects of the UP4FUN school-based family-focused intervention targeting screen time in 10- to 12-year-old children: the ENERGY project. BMC Public Health. 2014;14:857.
51. van Zutphen M, Bell AC, Kremer PJ, Swinburn BA. Association between the family environment and television viewing in Australian children. J Paediatr Child Health. 2007;43:458-463.
52. Vaughn AE, Hales D, Ward DS. Measuring the physical activity practices used by parents of preschool children. Med Sci Sports Exerc. 2013;45:2369-2377.
53. Veldhuis L, van Grieken A, Renders CM, Hirasing RA, Raat H. Parenting style, the home environment, and screen time of 5-year-old children; the “be active, eat right” study. PLoS One. 2014;9:e88486.
54. Anastassea-Vlachou K, Fryssira-Kanioura H, Papathanasiou-Klontza D, Xipolita-Zachariadi A, Matsaniotis N. The effects of television viewing in Greece, and the role of the paediatrician: a familiar triangle revisited. Eur J Pediatr. 1996;155:1057-1060.
55. Anderson PM. Parental employment, family routines and childhood obesity. Econ Hum Biol. 2012;10:340-351.
56. Appelhans BM, Fitzpatrick SL, Li H, et al. The home environment and childhood obesity in low-income households: indirect effects via sleep duration and screen time. BMC Public Health. 2014;14:1160.
57. Atkin AJ, Corder K, Ekelund U, Wijndaele K, Griffin SJ, van Sluijs EMF. Determinants of change in children’s sedentary time. PLoS One. 2013;8:e67627.
58. Bounova A, Michalopoulou M, Agelousis N, Kourtessis T, Gourgoulis V. Home and Neighborhood Environment Predictors of Adolescents’ Screen Viewing. J Phys Act Health. 2016;13:1310-1316.
59. Christakis DA, Ebel BE, Rivara FP, Zimmerman FJ. Television, video, and computer game usage in children under 11 years of age. J Pediatr. 2004;145:652-656.
60. Conlon BA, Mcginn AP, Isasi CR, et al. Home Environment Factors and Health Behaviors of Low-income, Overweight, and Obese Youth. Am J Health Behav. 2019;43:420-436.
61. Eisenberg ME, Larson NI, Berge JM, Thul C, Neumark-Sztainer D. The home physical activity environment and adolescent BMI, physical activity and TV viewing: Disparities across a diverse sample. J Racial Ethn Health disparities. 2014;1:326-336.
62. Emond JA, Tantum LK, Gilbert-Diamond D, Kim SJ, Lansigan RK, Neelon SB. Household chaos and screen media use among preschool-aged children: a cross-sectional study. BMC Public Health. 2018;18:1210.
63. Patriarca A, Di Giuseppe G, Albano L, Marinelli P, Angelillo IF. Use of television, videogames, and computer among children and adolescents in Italy. BMC Public Health. 2009;9:139.
64. Pearson N, Biddle SJH, Griffiths P, Johnston JP, Haycraft E. Clustering and correlates of screen-time and eating behaviours among young children. BMC Public Health. 2018;18:753.
65. Pearson N, Salmon J, Crawford D, Campbell K, Timperio A. Are parental concerns for child TV viewing associated with child TV viewing and the home sedentary environment? Int J Behav Nutr Phys Act. 2011;8:102.
66. Saelens BE, Sallis JF, Nader PR, Broyles SL, Berry CC, Taras HL. Home environmental influences on children’s television watching from early to middle childhood. J Dev Behav Pediatr. 2002;23:127-132.
67. Salmon J, Timperio A, Telford A, Carver A, Crawford D. Association of family environment with children’s television viewing and with low level of physical activity. Obes Res. 2005;13:1939-1951.
68. Aggio D, Smith L, Fisher A, Hamer M. Mothers’ perceived proximity to green space is associated with TV viewing time in children: the Growing Up in Scotland study. Prev Med (Baltim). 2015;70:46-49.
69. Appelhans BM, Fitzpatrick SL, Li H, et al. The home environment and childhood obesity in low-income households: indirect effects via sleep duration and screen time. BMC Public Health. 2014;14:1160.
70. Aznar S, Lara MT, Queralt A, Molina-Garcia J. Psychosocial and environmental correlates of sedentary behaviors in Spanish children. Biomed Res Int. 2017;2017:4728924.
71. Barr-Anderson DJ, Fulkerson JA, Smyth M, et al. Associations of American Indian children’s screen-time behavior with parental television behavior, parental perceptions of children’s screen time, and media-related resources in the home. Prev Chronic Dis. 2011;8:A105.
72. Bringolf-Isler B, de Hoogh K, Schindler C, et al. Sedentary Behaviour in Swiss Children and Adolescents: Disentangling Associations with the Perceived and Objectively Measured Environment. Int J Environ Res Public Health. 2018;15:918.
73. Carson V, Kuhle S, Spence JC, Veugelers PJ. Parents’ perception of neighbourhood environment as a determinant of screen time, physical activity and active transport. Can J Public Health. 2010;101:124-127
74. Cecil-Karb R, Grogan-Kaylor A. Childhood body mass index in community context: neighborhood safety, television viewing, and growth trajectories of BMI. Health Soc Work. 2009;34:169-177.
75. Datar A, Nicosia N, Shier V. Parent perceptions of neighborhood safety and children’s physical activity, sedentary behavior, and obesity: evidence from a national longitudinal study. Am J Epidemiol. 2013;177:1065-1073.
76. MacLeod KE, Gee GC, Crawford P, Wang MC. Neighbourhood environment as a predictor of television watching among girls. J Epidemiol Community Health. 2008;62:288-292.
77. Morowatisharifabad MA, Karimi M, Ghorbanzadeh F. Watching television by kids: How much and why? J Educ Health Promot. 2015;4:36.
78. Norman GJ, Schmid BA, Sallis JF, Calfas KJ, Patrick K. Psychosocial and environmental correlates of adolescent sedentary behaviors. Pediatrics. 2005;116:908-916.
79. Pearson N, Biddle SJH, Griffiths P, Johnston JP, Haycraft E. Clustering and correlates of screen-time and eating behaviours among young children. BMC Public Health. 2018;18:753.
80. Pearson N, Salmon J, Crawford D, Campbell K, Timperio A. Are parental concerns for child TV viewing associated with child TV viewing and the home sedentary environment?. Int J Behav Nutr Phys Act. 2011;8:102.
81. Quick V, Martin-Biggers J, Povis GA, Worobey J, Hongu N, Byrd-Bredbenner C. Long-term follow-up effects of the HomeStyles randomized controlled trial in families with preschool children on social cognitive theory constructs associated with physical activity cognitions and behaviors. Contemp Clin Trials. 2018;68:79-89.
82. Roemmich JN, Epstein LH, Raja S, Yin L. The neighborhood and home environments: disparate relationships with physical activity and sedentary behaviors in youth. Ann Behav Med. 2007;33:29-38.
83. Saelens BE, Sallis JF, Nader PR, Broyles SL, Berry CC, Taras HL. Home environmental influences on children’s television watching from early to middle childhood. J Dev Behav Pediatr. 2002;23:127-132.
84. Salmon J, Veitch J, Abbott G, et al. Are associations between the perceived home and neighbourhood environment and children’s physical activity and sedentary behaviour moderated by urban/rural location? Health Place. 2013;24:44-53.

**Parenting style (n=55)**

(e.g., authoritative, controlling/restrictive, permissive)

1. Kim HH, Chun JS. Is the relationship between parental abuse and mobile phone dependency (MPD) contingent across neighborhood characteristics? A multilevel analysis of Korean Children and Youth Panel Survey. PLoS One. 2018;13;5:e0196824.
2. He M, Harris S, Piche L, Beynon C. Understanding screen-related sedentary behavior and its contributing factors among school-aged children: a social-ecologic exploration. Am J Health Promot. 2009;23:299-308.
3. Innella N, McNaughton D, Schoeny M, et al. Child temperament, maternal feeding practices, and parenting styles and their influence on obesogenic behaviors in hispanic preschool children. J Sch Nurs. 2019;35(4):287-298.
4. Jago R, Davison KK, Thompson JL, Page AS, Brockman R, Fox KR. Parental sedentary restriction, maternal parenting style, and television viewing among 10- to 11-year-olds. Pediatrics. 2011;128:e572-e578.
5. Jago R, Edwards MJ, Urbanski CR, Sebire SJ. General and specific approaches to media parenting: a systematic review of current measures, associations with screen-viewing, and measurement implications. Child Obes. 2013;9:S51-72.
6. Jago R, Wood L, Zahra J, Thompson JL, Sebire SJ. Parental control, nurturance, self-efficacy, and screen viewing among 5- to 6-year-old children: a cross-sectional mediation analysis to inform potential behavior change strategies. Child Obes. 2015;11:139-147.
7. Langer SL, Crain AL, Senso MM, Levy RL, Sherwood NE. Predicting child physical activity and screen time: parental support for physical activity and general parenting styles. J Pediatr Psychol. 2013;6:633-642.
8. Van der Geest KE, Merelle SYM, Rodenburg G, Van de Mheen D, Renders CM. Cross-sectional associations between maternal parenting styles, physical activity and screen sedentary time in children. BMC Public Health. 2017;17:753.
9. Veldhuis L, van Grieken A, Renders CM, Hirasing RA, Raat H. Parenting style, the home environment, and screen time of 5-year-old children; the “be active, eat right” study. PLoS One. 2014;2:e88486.
10. Xu H, Wen LM, Rissel C. Associations of parental influences with physical activity and screen time among young children: a systematic review. J Obes. 2015;2015:546925.
11. Xu H, Wen LM, Rissel C. Associations of maternal influences with outdoor play and screen time of two-year-olds: Findings from the Healthy Beginnings Trial. J Paediatr Child Health. 2014;50:680-686.
12. Abedini Y, Zamani BE, Kheradmand A, Rajabizadeh G. Impacts of mothers’ occupation status and parenting styles on levels of self-control, addiction to computer games, and educational progress of adolescents. Addict Heal. 2012;4:102-110.
13. Aftosmes-Tobio A, Ganter C, Gicevic S, et al. A systematic review of media parenting in the context of childhood obesity research. *BMC Public Health*. 2016;16:320.
14. Augustine JM, Prickett KC, Kimbro R. Health-Related Parenting among U.S. Families and Young Children’s Physical Health. J Marriage Fam. 2017;79:816-832.
15. Barradas DT, Fulton JE, Blanck HM, Huhman M. Parental influences on youth television viewing. J Pediatr. 2007;151:364-369.
16. Bjelland M, Soenens B, Bere E, et al. Associations between parental rules, style of communication and children’s screen time. BMC Public Health. 2015;15:1002.
17. Brindova D, Pavelka J, Sevcikova A, et al. How parents can affect excessive spending of time on screen-based activities. BMC Public Health. 2014;14:1261.
18. Carlson SA, Fulton JE, Lee SM, Foley JT, Heitzler C, Huhman M. Influence of limit-setting and participation in physical activity on youth screen time. Pediatrics. 2010;126:e89-96.
19. Cheng JK, Koziol RL, Taveras EM. Parental guidance advised: associations between parental television limits and health behaviors among obese children. Acad Pediatr. 2015;15:204-209.
20. Chiu Y-C, Li Y-F, Wu W-C, Chiang T-L. The amount of television that infants and their parents watched influenced children’s viewing habits when they got older. Acta Paediatr. 2017;106:984-990.
21. Collier KM, Coyne SM, Rasmussen EE, et al. Does parental mediation of media influence child outcomes? A meta-analysis on media time, aggression, substance use, and sexual behavior. Dev Psychol. 2016;52:798-812.
22. Conlon BA, Mcginn AP, Isasi CR, et al. Home Environment Factors and Health Behaviors of Low-income, Overweight, and Obese Youth. Am J Health Behav. 2019;43:420-436.
23. Conlon BA, McGinn AP, Lounsbury DW, et al. The Role of Parenting Practices in the Home Environment among Underserved Youth. Child Obes. 2015;11:394-405.
24. Cui Z, Hardy LL, Dibley MJ, Bauman A. Temporal trends and recent correlates in sedentary behaviours in Chinese children. Int J Behav Nutr Phys Act. 2011;8:93.
25. De Decker E, Hesketh K, De Craemer M, et al. Parental influences on preschoolers’ TV viewing time: mediation analyses on Australian and Belgian data. J Phys Act Health. 2015;12:1272-1279.
26. De Jong E, Visscher TLS, HiraSing RA, Heymans MW, Seidell JC, Renders CM. Association between TV viewing, computer use and overweight, determinants and competing activities of screen time in 4- to 13-year-old children. Int J Obes (Lond). 2011;37:47-53.
27. De Lepeleere S, De Bourdeaudhuij I, Cardon G, Verloigne M. The effect of an online video intervention “Movie Models” on specific parenting practices and parental self-efficacy related to children’s physical activity, screen-time and healthy diet: a quasi experimental study. BMC Public Health. 2017;17:366.
28. De Lepeleere S, De Bourdeaudhuij I, Van Stappen V, et al. Parenting Practices as a Mediator in the Association Between Family Socio-Economic Status and Screen-Time in Primary Schoolchildren: A Feel4Diabetes Study. Int J Environ Res Public Health. 2018;15:2553.
29. Domoff SE, Lumeng JC, Kaciroti N, Miller AL. Early childhood risk factors for mealtime TV exposure and engagement in low-income families. Acad Pediatr. 2017;17:411-415.
30. Downing KL, Hinkley T, Hesketh KD. Associations of Parental Rules and Socioeconomic Position With Preschool Children’s Sedentary Behaviour and Screen Time. J Phys Act Health. 2015;12:515-521.
31. Draper CE, Grobler L, Micklesfield LK, Norris SA. Impact of social norms and social support on diet, physical activity and sedentary behaviour of adolescents: a scoping review. Child Care Health Dev. 2015;41:654-667
32. Epstein JA. The role of parents and related factors on adolescent computer use. J Public health Res. 2002;1:75-78.
33. McClendon ME, Umstattd Meyer MR, Ylitalo KR, Sharkey JR. Physical activity of Mexican-heritage youth during the summer and school-year: the role of parenting strategies. J Community Health. 2017;42:1102-1110.
34. Nagy LC, Horne M, Faisal M, Mohammed MA, Barber SE. Ethnic differences in sedentary behaviour in 6-8-year-old children during school terms and school holidays: a mixed methods study. BMC Public Health. 2019;19:152.
35. Norman GJ, Schmid BA, Sallis JF, Calfas KJ, Patrick K. Psychosocial and environmental correlates of adolescent sedentary behaviors. Pediatrics. 2005;116:908-916.
36. O’Connor TM, Chen T-A, Baranowski J, Thompson D, Baranowski T. Physical activity and screen-media-related parenting practices have different associations with children’s objectively measured physical activity. Child Obes. 2015;9:446-453
37. Patriarca A, Di Giuseppe G, Albano L, Marinelli P, Angelillo IF. Use of television, videogames, and computer among children and adolescents in Italy. BMC Public Health. 2009;9:139.
38. Pearson N, Salmon J, Crawford D, Campbell K, Timperio A. Are parental concerns for child TV viewing associated with child TV viewing and the home sedentary environment?. Int J Behav Nutr Phys Act. 2011;8:102.
39. Philips N, Sioen I, Michels N, Sleddens E, De Henauw S. The influence of parenting style on health related behavior of children: findings from the ChiBS study. Int J Behav Nutr Phys Act. 2014;11:95.
40. Ramirez ER, Norman GJ, Rosenberg DE, et al. Adolescent screen time and rules to limit screen time in the home. J Adolesc Health. 2011;48:379-385.
41. Ravikiran SR, Baliga BS, Jain A, Kotian MS. Factors influencing the television viewing practices of Indian children. Indian J Pediatr. 2014;81:114-119.
42. Ray C, Roos E. Family characteristics predicting favourable changes in 10 and 11-year-old children’s lifestyle-related health behaviours during an 18-month follow-up. Appetite. 2012;58:326-332.
43. Sanders W, Parent J, Forehand R, Breslend NL. The roles of general and technology-related parenting in managing youth screen time. J Fam Psychol. 2016;30:641-646.
44. Sanders W, Parent J, Forehand R, Sullivan ADW, Jones DJ. Parental perceptions of technology and technology-focused parenting: Associations with youth screen time. J Appl Dev Psychol. 2016;44:28-38.
45. Schmitz KH, Lytle LA, Phillips GA, Murray DM, Birnbaum AS, Kubik MY. Psychosocial correlates of physical activity and sedentary leisure habits in young adolescents: the Teens Eating for Energy and Nutrition at School study. Prev Med (Baltim). 2002;34:266-278.
46. Sebire SJ, Jago R. Parenting quality and television viewing among 10 year old children. Prev Med (Baltim). 2013;56:348-350.
47. Sleddens EFC, Gubbels JS, Kremers SPJ, van der Plas E, Thijs C. Bidirectional associations between activity-related parenting practices, and child physical activity, sedentary screen-based behavior and body mass index: a longitudinal analysis. Int J Behav Nutr Phys Act. 2017;14:89.
48. Springer AE, Kelder SH, Barroso CS, et al. Parental influences on television watching among children living on the Texas-Mexico border. Prev Med. 2010;51:112-117.
49. St George SM, Wilson DK, Schneider EM, Alia KA. Project SHINE: effects of parent-adolescent communication on sedentary behavior in African American adolescents. J Pediatr Psychol. 2013;38:997-1009.
50. Tang L, Darlington G, Ma DWL, Haines J. Mothers’ and fathers’ media parenting practices associated with young children’s screen-time: a cross-sectional study. BMC Obes. 2018;5:37.
51. Te Velde SJ, van der Horst K, Oenema A, Timperio A, Crawford D, Brug J. Parental and home influences on adolescents’ TV viewing: a mediation analysis. Int J Pediatr Obes. 2011;6:e364-e372.
52. Thompson DA, Johnson SL, Vandewater EA, et al. Parenting and Preschooler TV Viewing in Low-Income Mexican Americans: Development of the Parenting Practices Regarding TV Viewing (PPRTV) Scale. J Dev Behav Pediatr. 2016;37:465-474.
53. Thompson DA, Schmiege SJ, Johnson SL, et al. Screen-related parenting practices in low-income Mexican American families. Acad Pediatr. 2018;18:820-827.
54. Thompson DA, Vandewater EA, Matson PA, Tschann JM. Young low-income ethnic minority children watch less television when their mothers regulate what they are viewing. Acta Paediatr. 2015;104:300-305.
55. Totland TH, Bjelland M, Lien N, et al. Adolescents’ prospective screen time by gender and parental education, the mediation of parental influences. Int J Behav Nutr Phys Act. 2013;10:89.

**Parental beliefs, attitudes, and knowledge (n=48)**

(e.g., self-effiacy to limit sedentary behaviour/screen time, attitudes towards screen-based behaviours, perceived neighborhood safety)

1. Goh SN, Teh LH, Tay WR, et al. Sociodemographic, home environment and parental influences on total and device-specific screen viewing in children aged 2 years and below: an observational study. BMJ Open. 2016;6:e009113.
2. Golan M, Fainaru M, Weizman A. Role of behaviour modification in the treatment of childhood obesity with the parents as the exclusive agents of change. Int J Obes. 1998;22:1217-1224.
3. Goncalves WSF, Byrne R, Viana MT, Trost SG. Parental influences on screen time and weight status among preschool children from Brazil: a cross-sectional study. Int J Behav Nutr Phys Act. 2019;16:27
4. Hamilton K, Spinks T, White KM, Kavanagh DJ, Walsh AM. A psychosocial analysis of parents’ decisions for limiting their young child’s screen time: An examination of attitudes, social norms and roles, and control perceptions. Br J Health Psychol. 2016;21:285-301.
5. Hammersley ML, Okely AD, Batterham MJ, Jones RA. An internet-based childhood obesity prevention program (Time2bHealthy) for parents of preschool-aged children: randomized controlled trial. J Med Internet Res. 2019;21:e11964.
6. He M, Piche L, Beynon C, Harris S. Screen-related sedentary behaviors: children’s and parents’ attitudes, motivations, and practices. J Nutr Educ Behav. 2010;42:17-25.
7. Herman KM, Sabiston CM, Mathieu M-E, Tremblay A, Paradis G. Correlates of sedentary behaviour in 8- to 10-year-old children at elevated risk for obesity. Appl Physiol Nutr Metab. 2015;40:10-19.
8. Hnatiuk JA, Salmon J, Campbell KJ, Ridgers ND, Hesketh KD. Tracking of maternal self-efficacy for limiting young children’s television viewing and associations with children’s television viewing time: a longitudinal analysis over 15-months. BMC Public Health. 2015;15:517.
9. Inyang I, Benke G, Dimitriadis C, Simpson P, McKenzie R, Abramson M. Predictors of mobile telephone use and exposure analysis in Australian adolescents. J Paediatr Child Health. 2010;46:226-233.
10. Jago R, Sebire SJ, Edwards MJ, Thompson JL. Parental TV viewing, parental self-efficacy, media equipment and TV viewing among preschool children. Eur J Pediatr. 2013;172:1543-1545.
11. Jago R, Sebire SJ, Lucas PJ, et al. Parental modelling, media equipment and screen-viewing among young children: cross-sectional study. BMJ Open. 2013;3:e002593.
12. Jago R, Wood L, Zahra J, Thompson JL, Sebire SJ. Parental control, nurturance, self-efficacy, and screen viewing among 5- to 6-year-old children: a cross-sectional mediation analysis to inform potential behavior change strategies. Child Obes. 2015;11:139-147.
13. Krossbakken E, Torsheim T, Mentzoni RA, et al. The effectiveness of a parental guide for prevention of problematic video gaming in children: A public health randomized controlled intervention study. J Behav Addict. 2018;7:52-61.
14. Lampard AM, Jurkowski JM, Davison KK. Social-cognitive predictors of low-income parents’ restriction of screen time among preschool-aged children. Health Educ Behav. 2013;40:526-530.
15. Li M, Xue H, Wang W, Wang Y. Parental expectations and child screen and academic sedentary behaviors in China. Am J Prev Med. 2017;52:680-689.
16. Tucker JM, DeFrang R, Orth J, Wakefield S, Howard K. Evaluation of a Primary Care Weight Management Program in Children Aged 2(-)5 years: Changes in Feeding Practices, Health Behaviors, and Body Mass Index. Nutrients. 2019;11:498.
17. van Grieken A, Vlasblom E, Wang L, et al. Personalized web-based advice in combination with well-child visits to prevent overweight in young children: cluster randomized controlled trial. J Med Internet Res. 2017;19:e268.
18. Xu H, Wen LM, Rissel C. Associations of parental influences with physical activity and screen time among young children: a systematic review. J Obes. 2015;2015:546925.
19. Xu H, Wen LM, Rissel C. Associations of maternal influences with outdoor play and screen time of two-year-olds: Findings from the Healthy Beginnings Trial. J Paediatr Child Health. 2014;50:680-686.
20. Yilmaz G, Demirli Caylan N, Karacan CD. An intervention to preschool children for reducing screen time: a randomized controlled trial. Child Care Health Dev. 2015;41:443-449.
21. Zhang M, Quick V, Jin Y, Martin-Biggers J. Associations of mother’s behaviors and home/neighborhood environments with preschool children’s physical activity behaviors. Am J Health Promot. 2019:34;83-86.
22. Borg A, Haughton CF, Sawyer M, et al. Design and methods of the Healthy Kids & Families study: A parent-focused community health worker-delivered childhood obesity prevention intervention. BMC Obes. 2019;6:19.
23. Adams EL, Marini ME, Stokes J, Birch LL, Paul IM, Savage JS. INSIGHT responsive parenting intervention reduces infant’s screen time and television exposure. Int J Behav Nutr Phys Act. 2018;15:24.
24. Anderson CB, Hughes SO, Fuemmeler BF. Parent-child attitude congruence on type and intensity of physical activity: testing multiple mediators of sedentary behavior in older children. Health Psychol. 2009;28:428-438.
25. Asplund KM, Kair LR, Arain YH, Cervantes M, Oreskovic NM, Zuckerman KE. Early childhood screen time and parental attitudes toward child television viewing in a low-income Latino population attending the special supplemental nutrition program for women, infants, and children. Child Obes. 2015;11:590-599.
26. Barber SE, Kelly B, Collings PJ, Nagy L, Bywater T, Wright J. Prevalence, trajectories, and determinants of television viewing time in an ethnically diverse sample of young children from the UK. Int J Behav Nutr Phys Act. 2017;14:88.
27. Barnes AT, Plotnikoff RC, Collins CE, Morgan PJ. Maternal correlates of objectively measured physical activity in girls. Matern Child Health J. 2015;19:2348-2357.
28. Barr-Anderson DJ, Fulkerson JA, Smyth M, et al. Associations of American Indian children’s screen-time behavior with parental television behavior, parental perceptions of children’s screen time, and media-related resources in the home. Prev Chronic Dis. 2011;8:A105.
29. Campbell KJ, Lioret S, McNaughton SA, et al. A parent-focused intervention to reduce infant obesity risk behaviors: a randomized trial. Pediatrics. 2013;131:652-660.
30. Campbell K, Hesketh K, Silverii A, Abbott G. Maternal self-efficacy regarding children’s eating and sedentary behaviours in the early years: associations with children’s food intake and sedentary behaviours. Int J Pediatr Obes. 2010;5:501-508.
31. Carson V, Janssen I. Associations between factors within the home setting and screen time among children aged 0-5 years: a cross-sectional study. BMC Public Health. 2012;12:539.
32. Cespedes EM, Horan CM, Gillman MW, et al. Participant characteristics and intervention processes associated with reductions in television viewing in the High Five for Kids study. Prev Med. 2014;62:64-70.
33. Chen J-L, Guo J, Esquivel JH, Chesla CA. Like mother, like child: the influences of maternal attitudes and behaviors on weight-related health behaviors in their children. J Transcult Nurs Off J Transcult Nurs Soc. 2018;29:523-531.
34. Cheng ER, Bauer NS, Downs SM, Sanders LM. Parent health literacy, depression, and risk for pediatric injury. Pediatrics. 2016;138:e20160025.
35. Downing KL, Salmon J, Hinkley T, Hnatiuk JA, Hesketh KD. Feasibility and efficacy of a parent-focused, text message-delivered intervention to reduce sedentary behavior in 2- to 4-year-old children (Mini Movers): pilot randomized Controlled Trial. JMIR mHealth uHealth. 2018;6:e39.
36. Maatta S, Kaukonen R, Vepsalainen H, et al. The mediating role of the home environment in relation to parental educational level and preschool children’s screen time: a cross-sectional study. BMC Public Health. 2017;17:688.
37. McGuire MT, Hannan PJ, Neumark-Sztainer D, Cossrow NHF, Story M. Parental correlates of physical activity in a racially/ethnically diverse adolescent sample. J Adolesc Health. 2002;30:253-261.
38. Moshki M, Delshad Noghabi A, Darabi F, Safari Palangi H, Bahri N. The effect of educational programs based on the theory of planned behavior on parental supervision in students’ television watching. Med J Islam Repub Iran. 2016;30:406.
39. Pinon MF, Huston AC, Wright JC. Family ecology and child characteristics that predict young children’s educational television viewing. Child Dev. 1989;60:846-856.
40. Kim R, Lee KJ, Choi YJ. Mobile Phone Overuse Among Elementary School Students in Korea: Factors Associated With Mobile Phone Use as a Behavior Addiction. J Addict Nurs. 2015;26:81-85.
41. Sanders W, Parent J, Forehand R, Sullivan ADW, Jones DJ. Parental perceptions of technology and technology-focused parenting: associations with youth screen time. J Appl Dev Psychol. 2016;44:28-38.
42. Skouteris H, McCabe M, Swinburn B, Newgreen V, Sacher P, Chadwick P. Parental influence and obesity prevention in pre-schoolers: a systematic review of interventions. Obes Rev. 2011;12:315-328.
43. Smith BJ, Grunseit A, Hardy LL, King L, Wolfenden L, Milat A. Parental influences on child physical activity and screen viewing time: a population based study. BMC Public Health. 2010;10:593.
44. Solomon-Moore E, Sebire SJ, Macdonald-Wallis C, Thompson JL, Lawlor DA, Jago R. Exploring parents’ screen-viewing behaviours and sedentary time in association with their attitudes toward their young child’s screen-viewing. Prev Med reports. 2017;7:198-205.
45. Taras HL, Sallis JF, Nader PR, Nelson J. Children’s television-viewing habits and the family environment. Am J Dis Child. 1990;144:357-359.
46. Thompson DA, Johnson SL, Schmiege SJ, et al. Beliefs About Child TV Viewing in Low-Income Mexican American Parents of Preschoolers: Development of the Beliefs About Child TV Viewing Scale (B-TV). Matern Child Health J. 2018;22:849-857.
47. Thompson DA, Schmiege SJ, Johnson SL, et al. Screen-related parenting practices in low-income Mexican American families. Acad Pediatr. 2018;18:820-827.
48. Tomayko EJ, Prince RJ, Cronin KA, Kim K, Parker T, Adams AK. The Healthy Children, Strong Families 2 (HCSF2) Randomized Controlled Trial Improved Healthy Behaviors in American Indian Families with Young Children. Curr Dev Nutr. 2019;3:53-62.

**Family environment (n=27)**

(e.g., chaotic/disorganized family, family stress, stable/regular family routines)

1. Haines J, Rifas-Shiman SL, Horton NJ, et al. Family functioning and quality of parent-adolescent relationship: cross-sectional associations with adolescent weight-related behaviors and weight status. Int J Behav Nutr Phys Act. 2016;13:68.
2. Lee S-J, Bartolic S, Vandewater EA. Predicting children’s media use in the USA: differences in cross-sectional and longitudinal analysis. Br J Dev Psychol. 2009;27:123-143.
3. Loprinzi PD. Association of family functioning on youth physical activity and sedentary behavior. J Phys Act Health. 2015;12:642-648.
4. Walton K, Simpson JR, Darlington G, Haines J. Parenting stress: a cross-sectional analysis of associations with childhood obesity, physical activity, and TV viewing. BMC Pediatr. 2014;14:244.
5. Wang H, Zhou X, Lu C, Wu J, Deng X, Hong L. Problematic internet use in high school students in Guangdong Province, China. PLoS One. 2011;6:e19660.
6. Wartberg L, Kriston L, Kammerl R, Petersen K-U, Thomasius R. Prevalence of pathological internet use in a representative German sample of adolescents: results of a latent profile analysis. Psychopathology. 2015;48:25-30.
7. Appelhans BM, Fitzpatrick SL, Li H, et al. The home environment and childhood obesity in low-income households: indirect effects via sleep duration and screen time. BMC Public Health. 2014;14:1160.
8. Asplund KM, Kair LR, Arain YH, Cervantes M, Oreskovic NM, Zuckerman KE. Early Childhood Screen Time and Parental Attitudes Toward Child Television Viewing in a Low-Income Latino Population Attending the Special Supplemental Nutrition Program for Women, Infants, and Children. Child Obes. 2015;11:590-599.
9. Bounova A, Michalopoulou M, Agelousis N, Kourtessis T, Gourgoulis V. Home and neighborhood environment predictors of adolescents’ screen viewing. J Phys Act Health. 2016;13:1310-1316.
10. Brindova D, Pavelka J, Sevcikova A, et al. How parents can affect excessive spending of time on screen-based activities. BMC Public Health. 2014;14:1261.
11. Cameron AJ, van Stralen MM, Brug J, et al. Television in the bedroom and increased body weight: potential explanations for their relationship among European schoolchildren. Pediatr Obes. 2013;8:130-141.
12. Carson V, Janssen I. Associations between factors within the home setting and screen time among children aged 0-5 years: a cross-sectional study. BMC Public Health. 2012;12:539.
13. Christakis DA, Ebel BE, Rivara FP, Zimmerman FJ. Television, video, and computer game usage in children under 11 years of age. J Pediatr. 2004;145:652-656.
14. Cui Z, Hardy LL, Dibley MJ, Bauman A. Temporal trends and recent correlates in sedentary behaviours in Chinese children. Int J Behav Nutr Phys Act. 2011;8:93.
15. de Jong E, Visscher TLS, HiraSing RA, Heymans MW, Seidell JC, Renders CM. Association between TV viewing, computer use and overweight, determinants and competing activities of screen time in 4- to 13-year-old children. Int J Obes (Lond). 2013;37:47-53.
16. Downing KL, Salmon J, Timperio A, et al. Sitting and Screen Time Outside School Hours: Correlates in 6- to 8-Year-Old Children. J Phys Act Health. 2019;16:1-13.
17. Emond JA, Tantum LK, Gilbert-Diamond D, Kim SJ, Lansigan RK, Neelon SB. Household chaos and screen media use among preschool-aged children: a cross-sectional study. BMC Public Health. 2018;18:1210.
18. O’Connor TM, Chen T-A, Baranowski J, Thompson D, Baranowski T. Physical activity and screen-media-related parenting practices have different associations with children’s objectively measured physical activity. Child Obes. 2013;9:446-453.
19. Patriarca A, Di Giuseppe G, Albano L, Marinelli P, Angelillo IF. Use of television, videogames, and computer among children and adolescents in Italy. BMC Public Health. 2009;9:139.
20. Ramirez ER, Norman GJ, Rosenberg DE, et al. Adolescent screen time and rules to limit screen time in the home. J Adolesc Health. 2011;48:379-385.
21. Ravikiran SR, Baliga BS, Jain A, Kotian MS. Factors influencing the television viewing practices of Indian children. Indian J Pediatr. 2014;81:114-119.
22. Salmon J, Tremblay MS, Marshall SJ, Hume C. Health risks, correlates, and interventions to reduce sedentary behavior in young people. Am J Prev Med. 2011;41:197-206.
23. Smith LJ, Gradisar M, King DL. Parental influences on adolescent video game play: a study of accessibility, rules, limit setting, monitoring, and cybersafety. Cyberpsychol Behav Soc Netw. 2015;18:273-279.
24. Tandon PS, Zhou C, Sallis JF, Cain KL, Frank LD, Saelens BE. Home environment relationships with children’s physical activity, sedentary time, and screen time by socioeconomic status. Int J Behav Nutr Phys Act. 2012;9:88.
25. Tandon P, Grow HM, Couch S, et al. Physical and social home environment in relation to children’s overall and home-based physical activity and sedentary time. Prev Med (Baltim). 2014;66:39-44.
26. Te Velde SJ, van der Horst K, Oenema A, Timperio A, Crawford D, Brug J. Parental and home influences on adolescents’ TV viewing: a mediation analysis. Int J Pediatr Obes. 2011;6:e364-e372.
27. Thompson DA, Schmiege SJ, Johnson SL, et al. Screen-related parenting practices in low-Income Mexican American families. Acad Pediatr. 2018;18:820-827.

**Parental support (n=23)**

(i.e., support/encouragement)

1. Hohepa M, Scragg R, Schofield G, Kolt GS, Schaaf D. Associations between after-school physical activity, television use, and parental strategies in a sample of New Zealand adolescents. J Phys Act Health. 2009;6:299-305.
2. Huang WY, Wong SH, Salmon J. Correlates of physical activity and screen-based behaviors in Chinese children. J Sci Med Sport. 2013;16:509-514.
3. Bauer KW, Nelson MC, Boutelle KN, Neumark-Sztainer D. Parental influences on adolescents’ physical activity and sedentary behavior: Longitudinal findings from Project EAT-II. Int J Behav Nutr Phys Act. 2008;5:12.
4. Lau EY, Barr-Anderson DJ, Dowda M, Forthofer M, Saunders RP, Pate RR. Associations Between Home Environment and After-School Physical Activity and Sedentary Time Among 6th Grade Children. Pediatr Exerc Sci. 2015;27:226-233.
5. Leatherdale ST, Faulkner G, Arbour-Nicitopoulos K. School and student characteristics associated with screen-time sedentary behavior among students in grades 5-8, Ontario, Canada, 2007-2008. Prev Chronic Dis. 2010;7:A128.
6. Lowry R, Lee SM, Fulton JE, Demissie Z, Kann L. Obesity and other correlates of physical activity and sedentary behaviors among US high school students. J Obes. 2013;2013:276318.
7. Wang X, Liu Q-M, Ren Y-J, Lv J, Li L-M. Family influences on physical activity and sedentary behaviours in Chinese junior high school students: a cross-sectional study. BMC Public Health. 2015;15:287.
8. Springer AE, Kelder SH, Hoelscher DM. Social support, physical activity and sedentary behavior among 6th-grade girls: A cross-sectional study. Int J Behav Nutr Phys Act. 2006;3:8
9. Maltby AM, Vanderloo LM, Tucker P. Exploring mothers’ influence on preschoolers’ physical activity and sedentary time: a cross sectional study. Matern Child Health J. 2018;22:978-985.
10. Pyper E, Harrington D, Manson H. The impact of different types of parental support behaviours on child physical activity, healthy eating, and screen time: a cross-sectional study. BMC Public Health. 2016;16:568.
11. Barnes AT, Plotnikoff RC, Collins CE, Morgan PJ. Maternal correlates of objectively measured physical activity in girls. Matern Child Health J. 2015;19:2348-2357.
12. Barr-Anderson DJ, Robinson-O’Brien R, Haines J, Hannan P, Neumark-Sztainer D. Parental report versus child perception of familial support: which is more associated with child physical activity and television use?. J Phys Act Health. 2010;7:364-368.
13. Downing KL, Salmon J, Timperio A, et al. Sitting and screen time outside school hours: correlates in 6- to 8-Year-Old Children. J Phys Act Health. 2019;1:1-13.
14. Maltby AM, Vanderloo LM, Tucker P. Exploring Mothers’ Influence on Preschoolers’ Physical Activity and Sedentary Time: A Cross Sectional Study. Matern Child Health J. 2018;22:978-985.
15. Norman A, Zeebari Z, Nyberg G, Elinder LS. Parental support in promoting children’s health behaviours and preventing overweight and obesity - a long-term follow-up of the cluster-randomised healthy school start study II trial. BMC Pediatr. 2019;19:104.
16. Norman GJ, Schmid BA, Sallis JF, Calfas KJ, Patrick K. Psychosocial and environmental correlates of adolescent sedentary behaviors. Pediatrics. 2005;116:908-916.
17. Orehek E, Ferrer R. Parent instrumentality for adolescent eating and activity. Ann Behav Med. 2019;53:652-664.
18. Peltzer K, Pengpid S. Leisure Time Physical Inactivity and Sedentary Behaviour and Lifestyle Correlates among Students Aged 13-15 in the Association of Southeast Asian Nations (ASEAN) Member States, 2007-2013. Int J Environ Res Public Health. 2016;13:217.
19. Pyper E, Harrington D, Manson H. The impact of different types of parental support behaviours on child physical activity, healthy eating, and screen time: a cross-sectional study. BMC Public Health. 2016;16:568
20. Swindle TM, Jarrett D, McKelvey LM, Whiteside-Mansell L, Conners Edge NA, Kraleti S. Test of a Conceptual Model to Explain Television Exposure of Head Start Children. Clin Pediatr (Phila). 2018;57:970-980.
21. Tanaka C, Okuda M, Tanaka M, Inoue S, Tanaka S. Associations of physical activity and sedentary time in primary school children with their parental behaviors and supports. Int J Environ Res Public Health. 2019;15:1995.
22. Tandon P, Grow HM, Couch S, et al. Physical and social home environment in relation to children’s overall and home-based physical activity and sedentary time. Prev Med (Baltim). 2014;66:39-44.
23. Lawman HG, Wilson DK. Associations of social and environmental supports with sedentary behavior, light and moderate-to-vigorous physical activity in obese underserved adolescents. Int J Behav Nutr Phys Act. 2014;11:92.

**Family structure (n=18)**

(i.e., single-parent home, number of siblings, age of siblings)

1. Gao Y, Li LP, Kim JH, Congdon N, Lau J, Griffiths S. The impact of parental migration on health status and health behaviours among left behind adolescent school children in China. BMC Public Health. 2010;10:56.
2. Gomes TN, Hedeker D, Dos Santos FK, et al. Relationship between Sedentariness and Moderate-to-Vigorous Physical Activity in Youth: A Multivariate Multilevel Study. Int J Environ Res Public Health. 2017;14:148.
3. Hnatiuk JA, Hesketh KR, van Sluijs EMF. Correlates of home and neighbourhood-based physical activity in UK 3-4-year-old children. Eur J Public Health. 2016;26:947-953.
4. Hoyos Cillero I, Jago R. Sociodemographic and home environment predictors of screen viewing among Spanish school children. J Public Health (Oxf). 2011;33:392-402.
5. Inyang I, Benke G, Dimitriadis C, Simpson P, McKenzie R, Abramson M. Predictors of mobile telephone use and exposure analysis in Australian adolescents. J Paediatr Child Health. 2010;46:226-233.
6. Kracht CL, Sisson SB. Sibling influence on children’s objectively measured physical activity: a meta-analysis and systematic review. BMJ open Sport Exerc Med. 2018;4:e000405.
7. Hardy LL, Baur LA, Garnett SP, et al. Family and home correlates of television viewing in 12-13 year old adolescents: The Nepean Study. Int J Behav Nutr Phys Act. 2006;3:24.
8. Langoy A, Smith ORF, Wold B, Samdal O, Haug EM. Associations between family structure and young people’s physical activity and screen time behaviors. BMC Public Health. 2019;19:433.
9. Ylitalo KR, Bridges CN, Gutierrez M, Sharkey JR, Meyer MRU. Sibship, physical activity, and sedentary behavior: a longitudinal, observational study among Mexican-heritage sibling dyads. BMC Public Health. 2019;19:191.
10. Berge JM, Meyer C, MacLehose RF, Crichlow R, Neumark-Sztainer D. All in the family: correlations between parents’ and adolescent siblings’ weight and weight-related behaviors. Obesity. 2015;23:833-839.
11. Maher JP, Ra C, O’Connor SG, et al. Associations Between Maternal Mental Health and Well-being and Physical Activity and Sedentary Behavior in Children. J Dev Behav Pediatr. 2005;38:385-394.
12. McMillan R, McIsaac M, Janssen I. Family structure as a predictor of screen time among youth. PeerJ. 2015;3:e1048.
13. McVeigh JA, Norris SA, de Wet T. The relationship between socio-economic status and physical activity patterns in South African children. Acta Paediatr. 2004;93:982-988.
14. Mushtaq MU, Gull S, Mushtaq K, Shahid U, Shad MA, Akram J. Dietary behaviors, physical activity and sedentary lifestyle associated with overweight and obesity, and their socio-demographic correlates, among Pakistani primary school children. Int J Behav Nutr Phys Act. 2011;8:130.
15. Pinon MF, Huston AC, Wright JC. Family ecology and child characteristics that predict young children’s educational television viewing. Child Dev. 1989;60:846-856.
16. Quarmby T, Dagkas S, Bridge M. Associations between children’s physical activities, sedentary behaviours and family structure: a sequential mixed methods approach. Health Educ Res. 2011;26:63-76.
17. Sisson SB, Sheffield-Morris A, Spicer P, Lora K, Latorre C. Influence of family structure on obesogenic behaviors and placement of bedroom TVs of American children: National Survey of Children’s Health 2007. Prev Med (Baltim). 2014;61:48-53.
18. Spilkova J, Chomynova P, Csemy L. Predictors of excessive use of social media and excessive online gaming in Czech teenagers. J Behav Addict. 2017;6:611-619.

**Parental health (n=14)**

(e.g., mental health [depression, anxiety], general health, body mass index)

1. Francis LA, Lee Y, Birch LL. Parental weight status and girls’ television viewing, snacking, and body mass indexes. Obes Res. 2003;11:143-151.
2. Gopinath B, Hardy LL, Baur LA, Teber E, Mitchell P. Influence of parental history of hypertension on screen time and physical activity in young offspring. J Hypertens. 2012;30:336-341.
3. Kelishadi R, Qorbani M, Motlagh ME, Ardalan G, Heshmat R, Hovsepian S. Socioeconomic disparities in dietary and physical activity habits of Iranian children and adolescents: the CASPIAN-IV study. Arch Iran Med. 2016;19:530-537.
4. Characteristic lifestyles in 6-year-old children with obese parents: Results of the Toyama birth cohort study. Environ Health Prev Med. 2001;6:104-108.
5. Alhassan BA, Liu Y, Slawson D, et al. The influence of maternal body mass index and physical activity on select cardiovascular risk factors of preadolescent Hispanic children. PeerJ. 2018;6:e6100.
6. Angoorani P, Heshmat R, Ejtahed H-S, et al. The association of parental obesity with physical activity and sedentary behaviors of their children: the CASPIAN-V study. J Pediatr (Rio J). 2018;94:410-418.
7. Berge JM, Meyer C, MacLehose RF, Crichlow R, Neumark-Sztainer D. All in the family: correlations between parents’ and adolescent siblings’ weight and weight-related behaviors. Obesity (Silver Spring). 2015;23:833-839.
8. Burdette HL, Whitaker RC, Kahn RS, Harvey-Berino J. Association of maternal obesity and depressive symptoms with television-viewing time in low-income preschool children. Arch Pediatr Adolesc Med. 2003;157:894-899.
9. Sekine M, Yamagami, T. Saito T, et al. Characteristic lifestyles in 6-year-old children with obese parents: Results of the Toyama birth cohort study. Environ Health Prev Med. 2001;6:104-108.
10. O’Connor SG, Maher JP, Belcher BR, et al. Associations of maternal stress with children’s weight-related behaviours: a systematic literature review. Obes Rev*.* 2017;18:514-525.
11. Peneau S, Salanave B, Rolland-Cachera M-F, Hercberg S, Castetbon K. Correlates of sedentary behavior in 7 to 9-year-old French children are dependent on maternal weight status. Int J Obes. 2011;35:907-915.
12. Pona AA, Carlson JA, Shook RP, Dreyer Gillette ML, Davis AM. Maternal BMI Change Linked to Child Activity Change in Family-Based Behavioral Interventions for Pediatric Weight Management. Child Obes. 2019;15:371-378.
13. Rosenkranz RR, Bauer A, Dzewaltowski DA. Mother-daughter resemblance in BMI and obesity-related behaviors. Int J Adolesc Med Health. 2010;22:477-489.
14. Thompson AL, Adair LS, Bentley ME. Maternal characteristics and perception of temperament associated with infant TV exposure. Pediatr. 2013;131:e390-e397.

**Parent-child relationship (n=13)**

(i.e., strength/quality of relationship)

1. Haines J, Rifas-Shiman SL, Horton NJ, et al. Family functioning and quality of parent-adolescent relationship: cross-sectional associations with adolescent weight-related behaviors and weight status. Int J Behav Nutr Phys Act. 2016;13:68.
2. Koning IM, Peeters M, Finkenauer C, van den Eijnden RJJM. Bidirectional effects of Internet-specific parenting practices and compulsive social media and Internet game use. J Behav Addict. 2018;7:624-632.
3. Lei L, Wu Y. Adolescents’ paternal attachment and Internet use. Cyberpsychol Behav. 2007;10:633-639.
4. Willoughby T. A short-term longitudinal study of Internet and computer game use by adolescent boys and girls: prevalence, frequency of use, and psychosocial predictors. Dev Psychol. 2008;44:195-204.
5. Atkin AJ, Corder K, Goodyer I, et al. Perceived family functioning and friendship quality: cross-sectional associations with physical activity and sedentary behaviours. Int J Behav Nutr Phys Act. 2015;12:23.
6. Ballarotto G, Volpi B, Marzilli E, Tambelli R. Adolescent internet abuse: A study on the role of attachment to parents and peers in a large community sample. Biomed Res Int. 2018;2018:5769250.
7. Domoff SE, Lumeng JC, Kaciroti N, Miller AL. Early childhood risk factors for mealtime TV exposure and engagement in low-income families. Acad Pediatr. 2017;17:411-415.
8. Munoz-Miralles R, Ortega-Gonzalez R, Lopez-Moron MR, et al. The problematic use of Information and Communication Technologies (ICT) in adolescents by the cross sectional JOITIC study. BMC Pediatr. 2016;16:140.
9. Punamäki, R. L., Wallenius, M., Hölttö, H., Nygård, C. H., & Rimpelä, A. The associations between information and communication technology (ICT) and peer and parent relations in early adolescence. Int J Behav Dev. 2009;33:556-564.
10. Rosenkranz RR, Bauer A, Dzewaltowski DA. Mother-daughter resemblance in BMI and obesity-related behaviors. Int J Adolesc Med Health. 2010;22:477-489.
11. Schneider LA, King DL, Delfabbro PH. Family factors in adolescent problematic Internet gaming: A systematic review. J Behav Addict. 2017;6:321-333.
12. Shahraki-Sanavi F, Rakhshani F, Ansari-Moghaddam A, Mohammadi M, Feizabad AK. Prevalence of health-risk behaviors among teen girls in Southeastern Iran. Electron physician. 2018;10:6988-6996.
13. Skaug S, Englund KT, Wichstrom L. Young children’s television viewing and the quality of their interactions with parents: A prospective community study. Scand J Psychol. 2018;59:503-510.
