# Supplementary material for: Development of a consensus statement on the role of the family in the physical activity, sedentary, and sleep behaviours of children and youth
Source: Int J Behav Nutr Phys Act. 2020 Jun 16;17:74. doi: 10.1186/s12966-020-00973-0 (PMC7296673; doi:10.1186/s12966-020-00973-0)
Supplement: Supplementary file 5 — Additional file 5. Review #3 (docx.). Search Process for Family and Sleep Behaviour Literature Review (review #3). Themes from the sleep literature review (review #3). References for the papers included in the family and sleep review (review #3), organized by theme. [file 12966_2020_973_MOESM5_ESM.docx]

Records identified through databases searching
(n = 3,793)

Records after duplicates removed
(n = 2,487)

Records screened
(n = 189)

Identification

Eligibility

Screening

Records excluded

(n = 2,276)

**Search Process for Family and Sleep Behaviour Literature Review (review #3).**

**Themes from the sleep literature review (review #3).**

| **Theme** | **Topic** | **Number of studies** |
| --- | --- | --- |
| Household practices | Sleep hygiene  Bed sharing  Bedroom sharing  Solitary sleeping  Presence of screens in child’s bedroom  Bedtime routines  Parental nighttime involvement (e.g., parental presence until sleep, soothing techniques for infants)  Screen time limits  Family dinner routines | 81 |
| Sociodemographic factors | Parental age  Parental education  Parental employment status  Race/ethnicity  Household income | 54 |
| Family environment | Chaotic/disorganized family  Family stress  Disruptive behaviours of family members  Stable/regular family life routines | 29 |
| Organisation of child daily activities | Diet, caffeine intake  Exercise | 25 |
| Parental sleep | Bed/wake preference  Sleep patterns  Sleep duration | 23 |
| Parental mental health | Depression  Anxiety  Posttraumatic stress disorder  Psychological status  Drinking problem  Mood | 23 |
| Parent-child relationship | Parent-child conflict at bedtime  Attachment to the primary caregiver  Emotional security/support | 17 |
| Parenting style | Quality of parenting  Parental warmth/responsiveness  Harsh parenting  Parental hardiness | 13 |
| Inter-parent/sibling relationship | Parental marital conflict  Marital instability  Parental relationship dissolution  Emotional security on parental marital relationship | 10 |
| Parental beliefs, attitudes, knowledge | Knowledge of good sleep hygiene practices  Knowledge about normal sleep patterns in infancy  Knowledge of optimal sleep duration for child age  Perceptions of sleep patterns | 9 |
| Family structure | Crowded/multi-family housing | 3 |

**References for the papers included in the family and sleep review (review #3), organized by theme.**

**Household practices (n=81)**

(e.g., sleep hygiene, presence of screens in child’s bedroom; bedtime routines)

1. Ahn Y, Williamson AA, Seo H-J, Sadeh A, Mindell JA. Sleep patterns among South Korean infants and toddlers: Global comparison. J Korean Med Sci. 2016;31:261-269.
2. Allen SL, Howlett MD, Coulombe JA, Corkum P V. ABCs of SLEEPING: A review of the evidence behind pediatric sleep practice recommendations. Sleep Med Rev. 2016;29:1-14.
3. Ball HL. Together or apart? A behavioural and physiological investigation of sleeping arrangements for twin babies. Midwifery. 2007;23:404-412.
4. Beijers R, Cassidy J, Lustermans H, de Weerth C. Parent-infant room sharing during the first months of life: Longitudinal links with behavior during middle childhood. Child Dev. 2019;90:1350-1367.
5. Brambilla P, Giussani M, Pasinato A, et al. Sleep habits and pattern in 1-14 years old children and relationship with video devices use and evening and night child activities. Ital J Pediatr. 2017;43:7.
6. Buxton OM, Chang A-M, Spilsbury JC, Bos T, Emsellem H, Knutson KL. Sleep in the modern family: protective family routines for child and adolescent sleep. Sleep Heal. 2015;1:15-27.
7. Chahal H, Fung C, Kuhle S, Veugelers PJ. Availability and night-time use of electronic entertainment and communication devices are associated with short sleep duration and obesity among Canadian children. Pediatr Obes. 2013;8:42-51.
8. Chen T, Wu Z, Shen Z, Zhang J, Shen X, Li S. Sleep duration in Chinese adolescents: Biological, environmental, and behavioral predictors. Sleep Med. 2014;15:1345-1353.
9. Continente X, Perez A, Espelt A, Lopez MJ. Media devices, family relationships and sleep patterns among adolescents in an urban area. Sleep Med. 2017;32:28-35.
10. Cortesi F, Giannotti F, Sebastiani T, Vagnoni C, Marioni P. Cosleeping versus solitary sleeping in children with bedtime problems: Child emotional problems and parental distress. Behav Sleep Med. 2008;6:89-105.
11. Curell N, Vinallonga X, Cubells JM, et al. Sleeping with the parents: Prevalence and associated factors among children of 6 to 36 months of age. Pediatr Catalana. 1999;59:73-78
12. DeLeon CW, Karraker KH. Intrinsic and extrinsic factors associated with night waking in 9-month-old infants. Infant Behav Dev. 2007;30:596-605.
13. Fisher A, van Jaarsveld CHM, Llewellyn CH, Wardle J. Genetic and environmental influences on infant sleep. Pediatr. 2012;129:1091-1096.
14. Godsell S, White J. Adolescent perceptions of sleep and influences on sleep behaviour: A qualitative study. J Adolesc. 2019;73:18-25.
15. Huang XN, Wang HS, Zhang LJ, Liu XC. Co-sleeping and children’s sleep in China. Biol Rhythm Res. 2010;41:169-181.
16. Iwata S, Iwata O, Matsuishi T. Sleep patterns of Japanese preschool children and their parents: implications for co-sleeping. Acta Paediatr. 2013;102:e257-262.
17. Jiang F, Yan C, Wu S, et al. An epidemiological study on sleep problems in children aged 1 to 23 months in Shanghai. Zhonghua Yu Fang Yi Xue Za Zhi. 2003;37:435-438
18. Jiang Y, Chen W, Spruyt K, et al. Bed-sharing and related factors in early adolescents. Sleep Med. 2016;17:75-80.
19. Julian MM, Leung CYY, Rosenblum KL, et al. Parenting and toddler self-regulation in low-income families: What does sleep have to do with it? Infant Ment Health J. 2019;40:479-495.
20. Kaczor M, Skalski M. Treatment of behavioral sleep problems in children and adolescents - literature review. Psychiatr Pol. 2016;50:571-584.
21. Kelmanson IA. Sleep disturbances in two-month-old infants sharing the bed with parent(s). Minerva Pediatr. 2010;62:161-169.
22. Lee S, Ha JH, Moon DS, et al. Effect of sleep environment of preschool children on children’s sleep problems and mothers’ mental health. Sleep Biol Rhythms. 2019;17:277-285.
23. Lindsay AC, Moura Arruda CA, Tavares Machado MM, De Andrade GP, Greaney ML. Exploring Brazilian immigrant mothers’ beliefs, attitudes, and practices related to their preschool-age children’s sleep and bedtime routines: A qualitative study conducted in the United States. Int J Environ Res Public Health. 2018;15:e1923.
24. Liu Z, Wang G, Geng L, Luo J, Li N, Owens J. Sleep patterns, sleep disturbances, and associated factors among Chinese urban kindergarten children. Behav Sleep Med. 2016;14:100-117.
25. Lozoff B, Askew GL, Wolf AW. Cosleeping and early childhood sleep problems: effects of ethnicity and socioeconomic status. J Dev Behav Pediatr. 1996;17:9-15.
26. Meijer AM, Habekothe RT, van den Wittenboer GL. Mental health, parental rules and sleep in pre-adolescents. J Sleep Res. 2001;10:297-302
27. Mindell JA, Leichman ES, Walters RM. Sleep location and parent-perceived sleep outcomes in older infants. Sleep Med. 2017;39:1-7.
28. Mindell JA, Sadeh A, Kohyama J, How TH. Parental behaviors and sleep outcomes in infants and toddlers: a cross-cultural comparison. Sleep Med. 2010;11:393-399.
29. Muller D, Signal L, Elder D, Gander P. Environmental and behavioural factors associated with school children’s sleep in Aotearoa/New Zealand. J Paediatr Child Health. 2017;53:68-74.
30. Nusrat M, Khan A, Hamid S, Hussain AA, Kadir MM. Bedtime and its correlates among secondary school children in Karachi, Pakistan. J Pak Med Assoc. 2012;62:1168-1173
31. Nuutinen T, Ray C, Roos E. Do computer use, TV viewing, and the presence of the media in the bedroom predict school-aged children’s sleep habits in a longitudinal study? BMC Public Health. 2013;13:684.
32. Quach J, Hiscock H, Wake M. Sleep problems and mental health in primary school new entrants: cross-sectional community-based study. J Paediatr Child Health. 2012;48:1076-1081.
33. Schwichtenberg AJ, Goodlin-Jones B. Causes and correlates of frequent night awakenings in early childhood. Int Rev Neurobiol. 2010;93:177-191.
34. Sletten TL, Rajaratnam SMW, Wright MJ, et al. Genetic and environmental contributions to sleep-wake behavior in 12-year-old twins. Sleep. 2013;36:1715-1722.
35. Street NW, McCormick MC, Austin SB, Slopen N, Habre R, Molnar BE. Examining family and neighborhood level predictors of sleep duration in urban youth. Fam Syst Health. 2018;36:439-450.
36. Tie LJ, Yu H, Huang SJ. Cultural influences on the bedtime behaviour of Chinese children. Biol Rhythm Res. 2010;41:183-202.
37. Yu X-T, Sadeh A, Lam HS, Mindell JA, Li AM. Parental behaviors and sleep/wake patterns of infants and toddlers in Hong Kong, China. World J Pediatr. 2017;13:496-502.
38. Bartel KA, Gradisar M, Williamson P. Protective and risk factors for adolescent sleep: a meta-analytic review. Sleep Med Rev. 2015;21:72-85.
39. Cheng JK, Koziol RL, Taveras EM. Parental guidance advised: associations between parental television limits and health behaviors among obese children. Acad Pediatr. 2015;15:204-209.
40. Gentile DA, Reimer RA, Nathanson AI, Walsh DA, Eisenmann JC. Protective effects of parental monitoring of children’s media use: a prospective study. JAMA Pediatr. 2014;168:479-484.
41. Gunn HE, O’Rourke F, Dahl RE, et al. Young adolescent sleep is associated with parental monitoring. Sleep Heal. 2019;5:58-63.
42. John B. Sleep-patterns, sleep hygiene behaviors and parental monitoring among Bahrain-based Indian adolescents. J Fam Med Prim care. 2015;4:232-237.
43. Maume DJ. Social relationships and the sleep-health nexus in adolescence: evidence from a comprehensive model with bi-directional effects. Sleep Heal. 2017;3:284-289.
44. Meijer AM, Habekothe RT, van den Wittenboer GL. Mental health, parental rules and sleep in pre-adolescents. J Sleep Res. 2001;10:297-302
45. Meijer AM, Reitz E, Dekovic M. Parenting matters: a longitudinal study into parenting and adolescent sleep. J Sleep Res. 2016;25:556-564.
46. Mindell JA, Sadeh A, Kohyama J, How TH. Parental behaviors and sleep outcomes in infants and toddlers: a cross-cultural comparison. Sleep Med. 2010;11:393-399.
47. Patrick KE, Millet G, Mindell JA. Sleep differences by race in preschool children: The roles of parenting behaviors and socioeconomic status. Behav Sleep Med. 2016;14:467-479.
48. Pyper E, Harrington D, Manson H. Do parents’ support behaviours predict whether or not their children get sufficient sleep? A cross-sectional study. BMC Public Health. 2017;17:432.
49. Randler C, Bilger S. Associations among sleep, chronotype, parental monitoring, and pubertal development among German adolescents. J Psychol. 2009;143:509-520.
50. Rea CJ, Smith RL, Taveras EM. Associations of parent health behaviors and parenting practices with sleep duration in overweight and obese children. J Clin Sleep Med. 2016;12:1493-1498.
51. Short MA, Gradisar M, Wright H, Lack LC, Dohnt H, Carskadon MA. Time for bed: Parent-set bedtimes associated with improved sleep and daytime functioning in adolescents. Sleep. 2011;34:797-800.
52. Jiang F, Yan C, Wu S, et al. An epidemiological study on sleep problems in children aged 1 to 23 months in Shanghai. Zhonghua Yu Fang Yi Xue Za Zhi. 2003;37:435-438
53. Hale L, Berger LM, LeBourgeois MK, Brooks-Gunn J. A longitudinal study of preschoolers’ language-based bedtime routines, sleep duration, and well-being. J Fam Psychol. 2011;25:423-433.
54. Tikotzky L, Sadeh A, Volkovich E, Manber R, Meiri G, Shahar G. Infant sleep development from 3 to 6 months postpartum: links with maternal sleep and paternal involvement. Monogr Soc Res Child Dev. 2015;80:107-124.
55. Schwichtenberg AJ, Goodlin-Jones B. Causes and correlates of frequent night awakenings in early childhood. Int Rev Neurobiol. 2010;93:177-191.
56. Haines J, McDonald J, O’Brien A, et al. Healthy habits, happy homes: Randomized trial to improve household routines for obesity prevention among preschool-aged children. JAMA Pediatr. 2013;167:1072-1079.
57. Allen SL, Howlett MD, Coulombe JA, Corkum P V. ABCs of SLEEPING: A review of the evidence behind pediatric sleep practice recommendations. Sleep Med Rev. 2016;29:1-14.
58. Nevarez MD, Rifas-Shiman SL, Kleinman KP, Gillman MW, Taveras EM. Associations of early life risk factors with infant sleep duration. Acad Pediatr. 2010;10:187-193.
59. Mindell JA, Williamson AA. Benefits of a bedtime routine in young children: Sleep, development, and beyond. Sleep Med Rev. 2018;40:93-108.
60. Philbrook LE, Teti DM. Bidirectional associations between bedtime parenting and infant sleep: Parenting quality, parenting practices, and their interaction. J Fam Psychol. 2016;30:431-441.
61. Schwichtenberg AJ, Goodlin-Jones B. Causes and correlates of frequent night awakenings in early childhood. Int Rev Neurobiol. 2010;93:177-191.
62. Tie LJ, Yu H, Huang SJ. Cultural influences on the bedtime behaviour of Chinese children. *Biol Rhythm Res*. 2010;41:183-202.
63. Zapata Roblyer MI, Grzywacz JG. Demographic and Parenting Correlates of Adolescent Sleep Functioning. J Child Fam Stud. 2015;24:3331-3340.
64. Ozturk Donmez R, Bayik Temel A. Effect of soothing techniques on infants’ self-regulation behaviors (sleeping, crying, feeding): A randomized controlled study. Jpn J Nurs Sci. 2019;16:407-409.
65. Jones CHD, Ball H. Exploring Socioeconomic Differences in Bedtime Behaviours and Sleep Duration in English Preschool Children. *Infant Child Dev*. 2014;23:518-531.
66. Touchette É, Petit D, Paquet J, et al. Factors associated with fragmented sleep at night across early childhood. Arch Pediatr Adolesc Med. 2005;159:242-249.
67. Plancoulaine S, Lioret S, Regnault N, Heude B, Charles M-A, Group EM-CCS. Gender-specific factors associated with shorter sleep duration at age 3 years. J Sleep Res. 2015;24:610-620.
68. Tikotzky L, Sadeh A, Glickman-Gavrieli T. Infant sleep and paternal involvement in infant caregiving during the first 6 months of life. J Pediatr Psychol. 2011;36:36-46. DeLeon CW, Karraker KH. Intrinsic and extrinsic factors associated with night waking in 9-month-old infants. Infant Behav Dev. 2007;30:596-605.
69. Simard V, Nielsen TA, Tremblay RE, Boivin M, Montplaisir JY. Longitudinal study of preschool sleep disturbance: the predictive role of maladaptive parental behaviors, early sleep problems, and child/mother psychological factors. Arch Pediatr Adolesc Med. 2008;162:360-367.
70. Teti DM, Kim B-R, Mayer G, Countermine M. Maternal emotional availability at bedtime predicts infant sleep quality. J Fam Psychol. 2010;24:307-315.
71. Mindell JA, Sadeh A, Kohyama J, How TH. Parental behaviors and sleep outcomes in infants and toddlers: a cross-cultural comparison. Sleep Med. 2010;11:393-399.
72. Cowie J, Palmer CA, Hussain H, Alfano CA. Parental Involvement in Infant Sleep Routines Predicts Differential Sleep Patterns in Children With and Without Anxiety Disorders. Child Psychiatry Hum Dev. 2016;47:636-646.
73. Sadeh A, Tikotzky L, Scher A. Parenting and infant sleep. Sleep Med Rev. 2010;14:89-96.
74. Tikotzky L. Parenting and sleep in early childhood. Curr Opin Psychol. 2017;15:118-124.
75. Brown SJ, Rhee KE, Gahagan S. Reading at Bedtime Associated with Longer Nighttime Sleep in Latino Preschoolers. Clin Pediatr. 2015;55:525-531.
76. Sadeh A, Mindell JA, Luedtke K, Wiegand B. Sleep and sleep ecology in the first 3 years: a web-based study. J Sleep Res. 2009;18:60-73.
77. Brambilla P, Giussani M, Pasinato A, Venturelli L, Privitera F, Miraglia Del Giudice E, et al. Sleep habits and pattern in 1-14 years old children and relationship with video devices use and evening and night child activities. Ital J Pediatr. 2017;43:7.
78. Liu Z, Wang G, Geng L, Luo J, Li N, Owens J. Sleep patterns, sleep disturbances, and associated factors among Chinese urban kindergarten children. Behav Sleep Med. 2016;14:100-117.
79. Giannotti F, Cortesi F, Sebastiani T, Vagnoni C. Sleeping habits in Italian children and adolescents. Sleep Biol Rhythms. 2005;3:15-21.
80. Schlarb AA, Achterberg K, Brocki S, Ziemann A, Wiater A, Lollies F. Sleep-related parental behavior and sleep of children: a review. Monatsschr Kinderheilkd. 2017;165:239-247.
81. Kempler L, Sharpe L, Miller CB, Bartlett DJ. Do psychosocial sleep interventions improve infant sleep or maternal mood in the postnatal period? A systematic review and meta-analysis of randomised controlled trials. Sleep Med Rev. 2016;29:15-22.

**Sociodemographic factors (n=54)**

(e.g., parental age, parental education, household income)

1. de Ruiter I, Olmedo-Requena R, Sanchez-Cruz J-J, Jimenez-Moleon J-J. Changes in sleep duration in Spanish children aged 2-14 years from 1987 to 2011. *Sleep Med*. 2016;21:145-150.
2. McLaughlin Crabtree V, Beal Korhonen J, Montgomery-Downs HE, Faye Jones V, O’Brien LM, Gozal D. Cultural influences on the bedtime behaviors of young children. *Sleep Med*. 2005;6:319-324.
3. El-Sheikh M, Bagley EJ, Keiley M, Elmore-Staton L, Chen E, Buckhalt JA. Economic adversity and children’s sleep problems: Multiple indicators and moderation of effects. Health Psychol. 2013;32:849-859.
4. Jones CHD, Ball H. Exploring Socioeconomic Differences in Bedtime Behaviours and Sleep Duration in English Preschool Children. Infant Child Dev. 2014;23:518-531
5. Schmeer KK, Tarrence J, Browning CR, Calder CA, Ford JL, Boettner B. Family contexts and sleep during adolescence. SSM - Popul Heal. 2019;7:4.
6. Bao Z, Chen C, Zhang W, Zhu J, Jiang Y, Lai X. Family economic hardship and Chinese adolescents’ sleep quality: A moderated mediation model involving perceived economic discrimination and coping strategy. J Adolesc. 2016;50:81-90.
7. Marco CA, Wolfson AR, Sparling M, Azuaje A. Family socioeconomic status and sleep patterns of young adolescents. Behav Sleep Med. 2011;10:70-80.
8. Na M, Eagleton S, Jomaa L, Lawton K, Savage J. Food insecurity is associated with suboptimal sleep quality among low-income head start preschool-aged children. Curr Dev Nutr. 2019;3:nzz051.
9. Muller D, Paine S-J, Wu LJ, Signal TL. How long do preschoolers in Aotearoa/New Zealand sleep? Associations with ethnicity and socioeconomic position. Sleep Heal. 2019;5:452-458.
10. Kelly RJ, El-Sheikh M. Marital conflict and children’s sleep: reciprocal relations and socioeconomic effects. J Fam Psychol. 2011;25:412-422.
11. de Jong DM, Cremone A, Kurdziel LBF, et al. Maternal depressive symptoms and household income in relation to sleep in early childhood. J Pediatr Psychol. 2016;41:961-970.
12. Nagy E, Moore S, Gruber R, Paquet C, Arora N, Dube L. Parental social capital and children’s sleep disturbances. Sleep Heal. 2016;2:330-334.
13. Zhang J, Li AM, Fok TF, Wing YK. Roles of parental sleep/wake patterns, socioeconomic status, and daytime activities in the sleep/wake patterns of children. J Pediatr. 2010;156:606-12.e5.
14. Boe T, Hysing M, Stormark KM, Lundervold AJ, Sivertsen B. Sleep problems as a mediator of the association between parental education levels, perceived family economy and poor mental health in children. J Psychosom Res. 2012;73:430-436.
15. Ahn Y, Williamson AA, Seo H-J, Sadeh A, Mindell JA. Sleep Patterns among South Korean Infants and Toddlers: Global Comparison. J Korean Med Sci. 2016;31:261-269.
16. BaHammam A, Bin Saeed A, Al-Faris E, Shaikh S. Sleep duration and its correlates in a sample of Saudi elementary school children. Singapore Med J. 2006;47:875-881
17. Boe T, Hysing M, Stormark KM, Lundervold AJ, Sivertsen B. Sleep problems as a mediator of the association between parental education levels, perceived family economy and poor mental health in children. J Psychosom Res. 2012;73:430-436.
18. Brambilla P, Giussani M, Pasinato A, et al. Sleep habits and pattern in 1-14 years old children and relationship with video devices use and evening and night child activities. Ital J Pediatr. 2017;43:7.
19. de Ruiter I, Olmedo-Requena R, Sanchez-Cruz J-J, Jimenez-Moleon J-J. Changes in sleep duration in Spanish children aged 2-14 years from 1987 to 2011. Sleep Med. 2016;21:145-150.
20. El-Sheikh M, Bagley EJ, Keiley M, Elmore-Staton L, Chen E, Buckhalt JA. Economic adversity and children’s sleep problems: multiple indicators and moderation of effects. Health Psychol. 2013;32:849-859.
21. Marco CA, Wolfson AR, Sparling M, Azuaje A. Family socioeconomic status and sleep patterns of young adolescents. Behav Sleep Med. 2011;10:70-80.
22. Matenchuk BA, Tamana SK, Lou WYW, et al. Prenatal depression and birth mode sequentially mediate maternal education’s influence on infant sleep duration. Sleep Med. 2019;59:24-32.
23. McDowall PS, Elder DE, Campbell AJ. Relationship between parent knowledge of child sleep, and child sleep practices and problems: A pilot study in a children’s hospital cohort. J Paediatr Child Health. 2017;53:788-793.
24. Palmstierna P, Sepa A, Ludvigsson J. Parent perceptions of child sleep: A study of 10,000 Swedish children. Acta Paediatr. 2008;97:1631-1639.
25. Schmeer KK, Tarrence J, Browning CR, Calder CA, Ford JL, Boettner B. Family contexts and sleep during adolescence. SSM - Popul Heal. 2019;7:4.
26. Zapata Roblyer MI, Grzywacz JG. Demographic and Parenting Correlates of Adolescent Sleep Functioning. J Child Fam Stud. 2015;24:3331-3340
27. Watanabe E, Lee JS, Kawakubo K. Associations of maternal employment and three-generation families with pre-school children’s overweight and obesity in Japan. Int J Obes. 2011;35:945-952.
28. Nusrat M, Khan A, Hamid S, Hussain AA, Kadir MM. Bedtime and its correlates among secondary school children in Karachi, Pakistan. J Pak Med Assoc. 2012;62:1168-1173
29. Lawson KM, Davis KD, McHale SM, Hammer LB, Buxton OM. Daily positive spillover and crossover from mothers’ work to youth health. *J Fam Psychol*. 2014;28:897-907.
30. Muller D, Signal L, Elder D, Gander P. Environmental and behavioural factors associated with school children’s sleep in Aotearoa/New Zealand. J Paediatr Child Health. 2017;53:68-74.
31. Martinez SM, Thompson-Lastad A. Latino parents’ insight on optimal sleep for their preschool-age child: Does context matter? Acad Pediatr. 2015;15:636-643.
32. Yu X-T, Sadeh A, Lam HS, Mindell JA, Li AM. Parental behaviors and sleep/wake patterns of infants and toddlers in Hong Kong, China. World J Pediatr. 2017;13:496-502.
33. Jackson DB, Vaughn MG. Parental incarceration and child sleep and eating behaviors. J Pediatr. 2017;185:211-217.
34. Ahn Y, Williamson AA, Seo H-J, Sadeh A, Mindell JA. Sleep patterns among South Korean infants and toddlers: Global comparison. J Korean Med Sci. 2016;31:261-269.
35. Speirs KE, Liechty JM, Wu C-F, Team SKR. Sleep, but not other daily routines, mediates the association between maternal employment and BMI for preschool children. Sleep Med. 2014;15:1590-1593.
36. Schlieber M, Han J. The sleeping patterns of Head Start children and the influence on developmental outcomes. Child Care Health Dev. 2018;44:462-469.
37. McLaughlin Crabtree V, Beal Korhonen J, Montgomery-Downs HE, Faye Jones V, O’Brien LM, Gozal D. Cultural influences on the bedtime behaviors of young children. Sleep Med. 2005;6:319-324.
38. Zapata Roblyer MI, Grzywacz JG. Demographic and Parenting Correlates of Adolescent Sleep Functioning. J Child Fam Stud. 2015;24:3331-3340
39. El-Sheikh M, Bagley EJ, Keiley M, Elmore-Staton L, Chen E, Buckhalt JA. Economic adversity and children’s sleep problems: multiple indicators and moderation of effects. Health Psychol. 2013;32:849-859.
40. Muller D, Paine S-J, Wu LJ, Signal TL. How long do preschoolers in Aotearoa/New Zealand sleep? Associations with ethnicity and socioeconomic position. Sleep Heal. 2019;5:452-458.
41. Kelly RJ, El-Sheikh M. Marital conflict and children’s sleep: reciprocal relations and socioeconomic effects. J Fam Psychol. 2011;25:412-422.
42. Troxel WM, Lee L, Hall M, Matthews KA. Single-parent family structure and sleep problems in black and white adolescents. Sleep Med. 2014;15:255-261.
43. Patrick KE, Millet G, Mindell JA. Sleep differences by race in preschool children: The roles of parenting behaviors and socioeconomic status. Behav Sleep Med. 2016;14:467-479.
44. Labree LJ, van de Mheen H, Rutten FF, Rodenburg G, Koopmans GT, Foets M. Sleep duration differences between children of migrant and native origins. Z Gesundh Wiss. 2015;23:149-156
45. Schlieber M, Han J. The sleeping patterns of Head Start children and the influence on developmental outcomes. Child Care Health Dev. 2018;44:462-469.
46. Nevarez MD, Rifas-Shiman SL, Kleinman KP, Gillman MW, Taveras EM. Associations of early life risk factors with infant sleep duration. Acad Pediatr. 2010;10:187-193.
47. Watanabe E, Lee JS, Kawakubo K. Associations of maternal employment and three-generation families with pre-school children’s overweight and obesity in Japan. Int J Obes. 2011;35:945-952.
48. Schmeer KK, Tarrence J, Browning CR, Calder CA, Ford JL, Boettner B. Family contexts and sleep during adolescence. SSM - Popul Heal. 2019;7:4.
49. Palmstierna P, Sepa A, Ludvigsson J. Parent perceptions of child sleep: a study of 10,000 Swedish children. Acta Paediatr. 2008;97:1631-1639.
50. Julian MM, Leung CYY, Rosenblum KL, et al. Parenting and toddler self-regulation in low-income families: What does sleep have to do with it? Infant Ment Health J. 2019;40:479-495.
51. Troxel WM, Lee L, Hall M, Matthews KA. Single-parent family structure and sleep problems in black and white adolescents. Sleep Med. 2014;15:255-261.
52. Maume DJ. Social relationships and the sleep-health nexus in adolescence: evidence from a comprehensive model with bi-directional effects. Sleep Heal. 2017;3:284-289.
53. Martins AL, Chaves P, Papoila AL, Loureiro HC. The family role in children’s sleep disturbances: Results from a cross-sectional study in a Portuguese Urban pediatric population. Sleep Sci. 2015;8:108-114.
54. Sun W, Chen W, Jiang Y, et al. The association of sleep hygiene and sleep quality among school-age children. Zhonghua Yu Fang Yi Xue Za Zhi. 2012;46:713-717

**Family environment (n=29)**

(e.g., chaotic/disorganized family, family stress, disruptive behaviours of others in the household)

1. Adam EK, Snell EK, Pendry P. Sleep timing and quantity in ecological and family context: a nationally representative time-diary study. J Fam Psychol. 2007;21:4-19
2. Allen SL, Howlett MD, Coulombe JA, Corkum P V. ABCs of SLEEPING: A review of the evidence behind pediatric sleep practice recommendations. Sleep Med Rev. 2016;29:1-14.
3. Appelhans BM, Fitzpatrick SL, Li H, et al. The home environment and childhood obesity in low-income households: indirect effects via sleep duration and screen time. BMC Public Health. 2014;14:1160.
4. Bates CR, Bohnert AM, Buscemi J, Vandell DL, Lee KT, Bryant FB. Family entropy: understanding the organization of the family home environment and impact on child health behaviors and weight. Transl Behav Med. 2019;9:413-421.
5. Berger RH, Diaz A, Valiente C, et al. The association between home chaos and academic achievement: The moderating role of sleep. J Fam Psychol. 2019.
6. Billon-Descarpentries J. Influences of parental educational practices on child quality of sleep and attentional performances. Arch Pediatr. 1997;4:181-185.
7. Billows M, Gradisar M, Dohnt H, Johnston A, McCappin S, Hudson J. Family disorganization, sleep hygiene, and adolescent sleep disturbance. J Clin Child Adolesc Psychol. 2009;38:745-752.
8. Boles RE, Halbower AC, Daniels S, Gunnarsdottir T, Whitesell N, Johnson SL. Family chaos and child functioning in relation to sleep problems among children at risk for o. Behav Sleep Med. 2017;15:114-128.
9. Breitenstein RS, Doane LD, Clifford S, Lemery-Chalfant K. Children’s sleep and daytime functioning: Increasing heritability and environmental associations with sibling conflict. Soc Dev. 2018;27:967-983.
10. Buxton OM, Chang A-M, Spilsbury JC, Bos T, Emsellem H, Knutson KL. Sleep in the modern family: protective family routines for child and adolescent sleep. Sleep Heal. 2015;1:15-27.
11. Chang L-Y, Wu C-C, Yen L-L, Chang H-Y. The effects of family dysfunction trajectories during childhood and early adolescence on sleep quality during late adolescence: Resilience as a mediator. Soc Sci Med. 2019;222:162-170.
12. Continente X, Perez A, Espelt A, Lopez MJ. Media devices, family relationships and sleep patterns among adolescents in an urban area. Sleep Med. 2017;32:28-35.
13. Golem D, Eck KM, Delaney CL, et al. “My stuffed animals help me”: the importance, barriers, and strategies for adequate sleep behaviors of school-age children and parents. Sleep Heal. 2019;5:152-160.
14. Haines J, McDonald J, O’Brien A, et al. Healthy habits, happy homes: Randomized trial to improve household routines for obesity prevention among preschool-aged children. JAMA Pediatr. 2013;167:1072-1079.
15. Kaczor M, Skalski M. Treatment of behavioral sleep problems in children and adolescents - literature review. Psychiatr Pol. 2016;50:571-584.
16. Koopman-Verhoeff ME, Serdarevic F, Kocevska D, et al. Preschool family irregularity and the development of sleep problems in childhood: a longitudinal study. J Child Psychol Psychiatry. 2019;60:857-865.
17. Li S, Zhu S, Jin X, et al. Risk factors associated with short sleep duration among Chinese school-aged children. Sleep Med. 2010;11:907-916.
18. Marsh S, Gerritsen S, Taylor R, Galland B, Parag V, Maddison R. Promotion of family routines and positive parent-child interactions for obesity prevention: Protocol for the 3 pillars study randomized controlled trial. JMIR Res Protoc. 2019;8:e12792.
19. Martinez SM, Tschann JM, Butte NF, Gregorich SE, Penilla C, Flores E, et al. Sleep duration in Mexican American children: Do mothers’ and fathers’ parenting and family practices play a role? J Sleep Res. 2019;28:e12784.
20. Mindell JA, Leichman ES, Lee C, Williamson AA, Walters RM. Implementation of a nightly bedtime routine: How quickly do things improve? Infant Behav Dev. 2017;49:220-227.
21. Mindell JA, Li AM, Sadeh A, Kwon R, Goh DYT. Bedtime routines for young children: a dose-dependent association with sleep outcomes. Sleep. 2015;38:717-722.
22. Montgomery E, Foldspang A. Traumatic experience and sleep disturbance in refugee children from the Middle East. Eur J Public Health. 2001;11:18-22.
23. Peltz JS, Rogge RD, O’Connor TG. Adolescent sleep quality mediates family chaos and adolescent mental health: A daily diary-based study. J Fam Psychol. 2019;33:259-269.
24. Spilsbury JC, Patel SR, Morris N, Ehayaei A, Intille SS. Household chaos and sleep-disturbing behavior of family members: results of a pilot study of African American early adolescents. Sleep Heal. 2017;3:84-89.
25. Staples AD, Bates JE, Petersen IT. Bedtime routines in early childhood: prevalence, consistency, and associations with nighttime sleep. Monogr Soc Res Child Dev. 2015;80:141-159.
26. Tsai KM, Dahl RE, Irwin MR, Bower JE, McCreath H, Seeman TE, et al. The roles of parental support and family stress in adolescent sleep. Child Dev. 2018;89:1577-1588.
27. Tynjala J, Kannas L, Levalahti E, Valimaa R. Perceived sleep quality and its precursors in adolescents. Health Promot Int. 1999;14:155-166.
28. Whitesell CJ, Crosby B, Anders TF, Teti DM. Household chaos and family sleep during infants’ first year. J Fam Psychol. 2018;32:622-631.
29. Yoong SL, Grady A, Stacey F, Polimeni M, Clayton O, Jones J, et al. A pilot randomized controlled trial examining the impact of a sleep intervention targeting home routines on young children’s (3-6 years) physical activity. Pediatr Obes. 2019;14:e12481.

**Organization of child daily activities (n=25)**

(i.e., diet and caffeine intake, exercise)

1. Adam EK, Snell EK, Pendry P. Sleep timing and quantity in ecological and family context: a nationally representative time-diary study. J Fam Psychol. 2007;21:4-19
2. Allen SL, Howlett MD, Coulombe JA, Corkum P V. ABCs of SLEEPING: A review of the evidence behind pediatric sleep practice recommendations. Sleep Med Rev. 2016;29:1-14.
3. BaHammam A, Bin Saeed A, Al-Faris E, Shaikh S. Sleep duration and its correlates in a sample of Saudi elementary school children. Singapore Med J. 2006;47:875-881
4. Bartel KA, Gradisar M, Williamson P. Protective and risk factors for adolescent sleep: a meta-analytic review. Sleep Med Rev. 2015;21:72-85.
5. Brambilla P, Giussani M, Pasinato A, Venturelli L, Privitera F, Miraglia Del Giudice et al. Sleep habits and pattern in 1-14 years old children and relationship with video devices use and evening and night child activities. Ital J Pediatr. 2017;43:7.
6. Chen B, van Dam RM, Tan CS, et al. Screen viewing behavior and sleep duration among children aged 2 and below. BMC Public Health. 2019;19:59.
7. Chen T, Wu Z, Shen Z, Zhang J, Shen X, Li S. Sleep duration in Chinese adolescents: biological, environmental, and behavioral predictors. Sleep Med. 2014;15:1345-1353.
8. de Jong E, Stocks T, Visscher TLS, HiraSing RA, Seidell JC, Renders CM. Association between sleep duration and overweight: the importance of parenting. Int J Obes. 2012;36:1278-1284.
9. de Jong E, Visscher TLS, HiraSing RA, Heymans MW, Seidell JC, Renders CM. Association between TV viewing, computer use and overweight, determinants and competing activities of screen time in 4- to 13-year-old children. Int J Obes. 2013;37:47-53.
10. Jones CHD, Pollard TM, Summerbell CD, Ball H. Could parental rules play a role in the association between short sleep and obesity in young children?. J Biosoc Sci. 2014;46:405-418.
11. Kaczor M, Skalski M. Treatment of behavioral sleep problems in children and adolescents - literature review. Psychiatr Pol. 2016;50:571-584.
12. Koulouglioti C, Cole R, Moskow M, McQuillan B, Carno M-A, Grape A. The longitudinal association of young children’s everyday routines to sleep duration. J Pediatr Health Care. 2014;28:80-87.
13. Li S, Zhu S, Jin X, et al. Risk factors associated with short sleep duration among Chinese school-aged children. Sleep Med. 2010;11:907-916.
14. Maume DJ. Social relationships and the sleep-health nexus in adolescence: evidence from a comprehensive model with bi-directional effects. Sleep Heal. 2017;3:284-289.
15. Mindell JA, Williamson AA. Benefits of a bedtime routine in young children: Sleep, development, and beyond. Sleep Med Rev. 2018;40:93-108.
16. Nevarez MD, Rifas-Shiman SL, Kleinman KP, Gillman MW, Taveras EM. Associations of early life risk factors with infant sleep duration. Acad Pediatr. 2010;10:187-193.
17. Nusrat M, Khan A, Hamid S, Hussain AA, Kadir MM. Bedtime and its correlates among secondary school children in Karachi, Pakistan. J Pak Med Assoc. 2012;62:1168-1173
18. Nuutinen T, Ray C, Roos E. Do computer use, TV viewing, and the presence of the media in the bedroom predict school-aged children’s sleep habits in a longitudinal study? *BMC Public Health*. 2013;13:684.
19. Ogunleye AA, Voss C, Sandercock GR. Delayed bedtime due to screen time in schoolchildren: importance of area deprivation. Pediatr Int. 2015;57:137-142.
20. Pieters D, De Valck E, Vandekerckhove M, Pirrera S, Wuyts J, Exadktylos V, et al. Effects of pre-sleep media use on sleep/wake patterns and daytime functioning among adolescents: the moderating role of parental control. Behav Sleep Med. 2014;12:427-443.
21. Plancoulaine S, Lioret S, Regnault N, Heude B, Charles M-A, Group EM-CCS. Gender-specific factors associated with shorter sleep duration at age 3 years. J Sleep Res. 2015;24:610-620.
22. Quach J, Hiscock H, Wake M. Sleep problems and mental health in primary school new entrants: cross-sectional community-based study. J Paediatr Child Health. 2012;48:1076-1081.
23. Ray C, Roos E. Family characteristics predicting favourable changes in 10 and 11-year-old children’s lifestyle-related health behaviours during an 18-month follow-up. Appetite. 2012;58:326-332.
24. Schmeer KK, Tarrence J, Browning CR, Calder CA, Ford JL, Boettner B. Family contexts and sleep during adolescence. SSM - Popul Heal. 2019;7:4.
25. Shinkoda H, Matsumoto K, Asami E, Suetsugu Y, Kato N, Uchimura N, et al. Analysis of late bedtime and influencing factors for it with respect to infants’ development age and sleep behavior of parents and children. Fukuoka Igaku Zasshi. 2008;99:246-261

**Parental sleep (n=23)**

(i.e., bed/wake-up time preference, sleep patterns, sleep duration)

1. Jiang F, Yan C, Wu S, et al. An epidemiological study on sleep problems in children aged 1 to 23 months in Shanghai. Zhonghua Yu Fang Yi Xue Za Zhi. 2003;37:435-438
2. Mindell JA, Williamson AA. Benefits of a bedtime routine in young children: Sleep, development, and beyond. Sleep Med Rev. 2018;40:93-108.
3. Rea CJ, Smith RL, Taveras EM. Associations of parent health behaviors and parenting practices with sleep duration in overweight and obese children. J Clin Sleep Med. 2016;12:1493-1498.
4. Roberts CM, Harper KL, Bistricky SL, Short MB. Bedtime behaviours: Parental mental health, parental sleep, parental accommodation, and children’s sleep disturbance. Child Heal Care. 2019. In press.
5. Schmeer KK, Tarrence J, Browning CR, Calder CA, Ford JL, Boettner B. Family contexts and sleep during adolescence*.* SSM - Popul Heal. 2019;7:4.
6. Shinkoda H, Matsumoto K, Asami E, et al. Analysis of late bedtime and influencing factors for it with respect to infants’ development age and sleep behavior of parents and children. Fukuoka Igaku Zasshi. 2008;99:246-261
7. Street NW, McCormick MC, Austin SB, Slopen N, Habre R, Molnar BE. Examining family and neighborhood level predictors of sleep duration in urban youth. Fam Syst Health. 2018;36:439-450.
8. Fuligni AJ, Tsai KM, Krull JL, Gonzales NA. Daily concordance between parent and adolescent sleep habits. J Adolesc Health. 2015;56:244-250.
9. Brand S, Gerber M, Hatzinger M, Beck J, Holsboer-Trachsler E. Evidence for similarities between adolescents and parents in sleep patterns. Sleep Med. 2009;10:1124-1131.
10. Street NW, McCormick MC, Austin SB, Slopen N, Habre R, Molnar BE. Examining family and neighborhood level predictors of sleep duration in urban youth. Fam Syst Health. 2018;36:439-450.
11. Tikotzky L, Sadeh A, Volkovich E, Manber R, Meiri G, Shahar G. Infant sleep development from 3 to 6 months postpartum: links with maternal sleep and paternal involvement. Monogr Soc Res Child Dev. 2015;80:107-124.
12. Jones BL, Fiese BH, Team SK. Parent routines, child routines, and family demographics associated with obesity in parents and preschool-aged children. Front Psychol. 2014;5:374.
13. Tu AW, Watts AW, Masse LC. Parent–Adolescent Patterns of Physical Activity, Sedentary Behaviors and Sleep among a Sample of Overweight and Obese Adolescents. J Phys Act Heal. 2015.
14. Li S, Zhu S, Jin X, et al. Risk factors associated with short sleep duration among Chinese school-aged children. Sleep Med. 2010;11:907-916.
15. Zhang J, Li AM, Fok TF, Wing YK. Roles of parental sleep/wake patterns, socioeconomic status, and daytime activities in the sleep/wake patterns of children. J Pediatr. 2010;156:606-12.e5.
16. Komada Y, Abe T, Okajima I, et al. Short sleep duration and irregular bedtime are associated with increased behavioral problems among Japanese preschool-age children. Tohoku J Exp Med. 2011;224:127-136
17. Bajoghli H, Alipouri A, Holsboer-Trachsler E, Brand S. Sleep patterns and psychological functioning in families in northeastern Iran; evidence for similarities between adolescent children and their parents. J Adolesc. 2013;36:1103-1113.
18. Chen T, Wu Z, Shen Z, Zhang J, Shen X, Li S. Sleep duration in Chinese adolescents: biological, environmental, and behavioral predictors. Sleep Med. 2014;15:1345-1353.
19. Iwata S, Iwata O, Matsuishi T. Sleep patterns of Japanese preschool children and their parents: implications for co-sleeping. Acta Paediatr. 2013;102:e257-262.
20. Kalak N, Gerber M, Kirov R, et al. The relation of objective sleep patterns, depressive symptoms, and sleep disturbances in adolescent children and their parents: a sleep-EEG study with 47 families. J Psychiatr Res. 2012;46:1374-1382.
21. Van Tassel EB. The relative influence of child and environmental characteristics on sleep disturbances in the first and second years of life. J Dev Behav Pediatr. 1985;6:81-86
22. Morales-Munoz I, Partonen T, Saarenpaa-Heikkila O, et al. The role of parental circadian preference in the onset of sleep difficulties in early childhood. Sleep Med. 2019;54:223-230.
23. Kouros CD, El-Sheikh M. Within-family Relations in Objective Sleep Duration, Quality, and Schedule. Child Dev. 2017;88:1983-2000.

**Parental mental health (n=23)**

(e.g., depression, anxiety, posttraumatic stress disorder)

1. Jiang F, Yan C, Wu S, et al. An epidemiological study on sleep problems in children aged 1 to 23 months in Shanghai. Zhonghua Yu Fang Yi Xue Za Zhi. 2003;37:435-438
2. Nevarez MD, Rifas-Shiman SL, Kleinman KP, Gillman MW, Taveras EM. Associations of early life risk factors with infant sleep duration. *Acad Pediatr*. 2010;10:187-193.
3. Roberts CM, Harper KL, Bistricky SL, Short MB. Bedtime behaviors: Parental mental health, parental sleep, parental accommodation, and children’s sleep disturbance. Child Heal Care. 2019. In press.
4. Lawson KM, Davis KD, McHale SM, Hammer LB, Buxton OM. Daily positive spillover and crossover from mothers’ work to youth health. J Fam Psychol. 2014;28:897-907.
5. Armitage R, Flynn H, Hoffmann R, Vazquez D, Lopez J, Marcus S. Early developmental changes in sleep in infants: the impact of maternal depression. Sleep. 2009;32:693-696
6. Miadich SA, Doane LD, Davis MC, Lemery-Chalfant K. Early parental positive personality and stress: Longitudinal associations with children’s sleep. Br J Health Psychol. 2019;24:629-650.
7. Dubois-Comtois K, Pennestri M-H, Bernier A, Cyr C, Godbout R. Family environment and preschoolers’ sleep: the complementary role of both parents. Sleep Med. 2019;58:114-122.
8. Schmeer KK, Tarrence J, Browning CR, Calder CA, Ford JL, Boettner B. Family contexts and sleep during adolescence*.* SSM - Popul Heal. 2019;7:4.
9. Keller PS, Kouros CD, Erath SA, Dahl RE, El-Sheikh M. Longitudinal relations between maternal depressive symptoms and child sleep problems: The role of parasympathetic nervous system reactivity. J Child Psychol Psychiatry. 2014;55:172-179.
10. Gross RS, Velazco NK, Briggs RD, Racine AD. Maternal depressive symptoms and child obesity in low-income urban families. Acad Pediatr. 2013;13:356-363.
11. de Jong DM, Cremone A, Kurdziel LBF, et al. Maternal depressive symptoms and household income in relation to sleep in early childhood. J Pediatr Psychol. 2016;41:961-970.
12. Caldwell BA, Redeker NS. Maternal stress and psychological status and sleep in minority preschool children. Public Health Nurs. 2015;32:101-111.
13. Ystrom E, Hysing M, Torgersen L, Ystrom H, Reichborn-Kjennerud T, Sivertsen B. Maternal Symptoms of Anxiety and Depression and Child Nocturnal Awakenings at 6 and 18 Months. J Pediatr Psychol. 2017;42:1156-1164.
14. El-Sheikh M, Kelly RJ, Bagley EJ, Wetter EK. Parental depressive symptoms and children’s sleep: the role of family conflict. J Child Psychol Psychiatry. 2012;53:806-814.
15. Kelly RJ, El-Sheikh M. Parental problem drinking and children’s sleep: The role of ethnicity and socioeconomic status. J Fam Psychol. 2016;30:708-719.
16. Sadeh A, Tikotzky L, Scher A. Parenting and infant sleep. Sleep Med Rev. 2010;14:89-96.
17. Tikotzky L. Parenting and sleep in early childhood. Curr Opin Psychol. 2017;15:118-124.
18. Heerman WJ, Taylor JL, Wallston KA, Barkin SL. Parenting self-efficacy, parent depression, and healthy childhood behaviors in a low-income minority population: A cross-sectional analysis. Matern Child Health J. 2017;21:1156-1165.
19. Gress-Smith JL, Luecken LJ, Lemery-Chalfant K, Howe R. Postpartum depression prevalence and impact on infant health, weight, and sleep in low-income and ethnic minority women and infants. Matern Child Health J. 2012;16:887-893.
20. Kelly RJ, El-Sheikh M. Reciprocal relations between parental problem drinking and children’s sleep: The role of socioeconomic adversity. Child Dev. 2019.90:1987-2000.
21. Michaeli Manee F, Alizadeh S, Hassan Pour E, Sadighie Z. Relationship between mothers’ depression and sleep problems in 3-6 year old preschool children. J Maz Univ Med Sci. 2015;25:102-117.
22. Quach J, Hiscock H, Wake M. Sleep problems and mental health in primary school new entrants: cross-sectional community-based study. J Paediatr Child Health. 2012;48:1076-1081.
23. Garthus-Niegel S, Horsch A, Bickle Graz M, et al. The prospective relationship between postpartum PTSD and child sleep: A 2-year follow-up study. J Affect Disord. 2018;241:71-79.

**Parent-child relationship (n=17)**

(i.e., parent-child conflict at bedtime, attachment to the primary caregiver)

1. Alfano CA, Smith VC, Reynolds KC, Reddy R, Dougherty LR. The Parent-Child Sleep Interactions Scale (PSIS) for preschoolers: factor structure and initial psychometric properties. J Clin Sleep Med. 2013;9:1153-1160.
2. Allen SL, Howlett MD, Coulombe JA, Corkum P V. ABCs of SLEEPING: A review of the evidence behind pediatric sleep practice recommendations. Sleep Med Rev. 2016;29:1-14.
3. Barrios CS, Jay SY, Smith VC, Alfano CA, Dougherty LR. Stability and predictive validity of the parent-child sleep interactions scale: A longitudinal study among preschoolers. J Clin Child Adolesc Psychol. 2018;47:382-396.
4. Beijers R, Jansen J, Riksen-Walraven M, de Weerth C. Attachment and infant night waking: a longitudinal study from birth through the first year of life. J Dev Behav Pediatr. 2011;32:635-643.
5. Belanger M-E, Bernier A, Simard V, Bordeleau S, Carrier J. Viii. Attachment and sleep among toddlers: disentangling attachment security and dependency. Monogr Soc Res Child Dev. 2015;80:125-140.
6. Bernier A, Tetreault E, Belanger ME, Carrier J. Paternal involvement and child sleep: A look beyond infancy. Int J Behav Dev. 2017;41:714-722.
7. Bordeleau S, Bernier A, Carrier J. Longitudinal associations between the quality of parent-child interactions and children’s sleep at preschool age. J Fam Psychol. 2012;12:254-262.
8. Dubois-Comtois K, Pennestri M-H, Bernier A, Cyr C, Godbout R. Family environment and preschoolers’ sleep: The complementary role of both parents. Sleep Med. 2019;58:114-122.
9. El-Sheikh M, Kelly RJ, Bagley EJ, Wetter EK. Parental depressive symptoms and children’s sleep: the role of family conflict. J Child Psychol Psychiatry. 2012;53:806-814.
10. Johnson N, McMahon C. Preschoolers’ sleep behaviour: associations with parental hardiness, sleep-related cognitions and bedtime interactions. J Child Psychol Psychiatry. 2008;49:765-773.
11. Keller P, El-Sheikh M. Children’s emotional security and sleep: longitudinal relations and directions of effects. J Child Psychol Psychiatry. 2011;52:64-71.
12. Kelly RJ, Marks BT, El-Sheikh M. Longitudinal relations between parent-child conflict and children’s adjustment: the role of children’s sleep. J Abnorm Child Psychol. 2014;42:1175-1185.
13. Meijer AM, Reitz E, Dekovic M. Parenting matters: a longitudinal study into parenting and adolescent sleep. J Sleep Res. 2016;25:556-564.
14. Peltz JS, Rogge RD. The moderating role of parents’ dysfunctional sleep-related beliefs among associations between adolescents’ pre-bedtime conflict, sleep quality, and their mental halth. J Clin Sleep Med. 2019;15:265-274.
15. Schwichtenberg AJ, Goodlin-Jones B. Causes and correlates of frequent night awakenings in early childhood. Int Rev Neurobiol. 2010;93:177-191.
16. Tikotzky L. Parenting and sleep in early childhood. Curr Opin Psychol. 2017;15:118-124.
17. Zapata Roblyer MI, Grzywacz JG. Demographic and parenting correlates of adolescent sleep functioning. J Child Fam Stud. 2015;24:3331-3340

**Parenting style (n=13)**

(i.e., quality of parenting, parental warmth/responsiveness, harsh parenting)

1. Ray C, Kalland M, Lehto R, Roos E. Does parental warmth and responsiveness moderate the associations between parenting practices and children’s health-related behaviors? J Nutr Educ Behav. 2013;45:602-610.
2. Spilsbury JC, Storfer-Isser A, Drotar D, Rosen CL, Kirchner HL, Redline S. Effects of the home environment on school-aged children’s sleep. Sleep. 2005;28:1419-1427
3. Brand S, Gerber M, Hatzinger M, Beck J, Holsboer-Trachsler E. Evidence for similarities between adolescents and parents in sleep patterns. Sleep Med. 2009;10:1124-1131.
4. El-Sheikh M, Tu KM, Erath SA, Buckhalt JA. Family stress and adolescents’ cognitive functioning: sleep as a protective factor. J Fam Psychol. 2014;28:887-896.
5. Bordeleau S, Bernier A, Carrier J. Maternal sensitivity and children’s behavior problems: examining the moderating role of infant sleep duration. J Clin Child Adolesc Psychol. 2012;41:471-481.
6. Vazsonyi AT, Harris C, Terveer AM, Pagava K, Phagava H, Michaud P-A. Parallel mediation effects by sleep on the parental warmth-problem behavior links: evidence from national probability samples of Georgian and Swiss adolescents. J Youth Adolesc. 2015;44:331-345.
7. Brand S, Hatzinger M, Beck J, Holsboer-Trachsler E. Perceived parenting styles, personality traits and sleep patterns in adolescents. J Adolesc. 2009;32:1189-1207.
8. Johnson N, McMahon C. Preschoolers’ sleep behaviour: Associations with parental hardiness, sleep-related cognitions and bedtime interactions. J Child Psychol Psychiatry. 2008;49:765-773.
9. Labree LJ, van de Mheen H, Rutten FF, Rodenburg G, Koopmans GT, Foets M. Sleep duration differences between children of migrant and native origins. Z Gesundh Wiss. 2015;23:149-156.
10. Martinez SM, Tschann JM, Butte NF, et al. Sleep duration in Mexican American children: Do mothers’ and fathers’ parenting and family practices play a role?. J Sleep Res. 2019;28(4 PG-e12784):e12784.
11. Meltzer LJ, Montgomery-Downs HE. Sleep in the family. Pediatr Clin North Am. 2011;58(3 PG-765-74):765-774.
12. Adam EK, Snell EK, Pendry P. Sleep timing and quantity in ecological and family context: a nationally representative time-diary study. J Fam Psychol. 2007;21:4-19
13. Philips N, Sioen I, Michels N, Sleddens E, De Henauw S. The influence of parenting style on health related behavior of children: findings from the ChiBS study. Int J Behav Nutr Phys Act. 2014;11:95.

**Inter-parent/sibling relationship (n=10)**

(e.g., parental marital conflict, marital instability, parental relationship dissolution)

1. Keller P, El-Sheikh M. Children’s emotional security and sleep: longitudinal relations and directions of effects. J Child Psychol Psychiatry. 2011;52:64-71.
2. El-Sheikh M, Tu KM, Erath SA, Buckhalt JA. Family stress and adolescents’ cognitive functioning: sleep as a protective factor. J Fam Psychol. 2014;28:887-896.
3. Mannering AM, Harold GT, Leve LD, et al. Longitudinal associations between marital instability and child sleep problems across infancy and toddlerhood in adoptive families. Child Dev. 2011;82:1252-1266.
4. Kelly RJ, El-Sheikh M. Marital conflict and children’s sleep: reciprocal relations and socioeconomic effects. J Fam Psychol. 2011;25:412-422.
5. El-Sheikh M, Kelly RJ, Bagley EJ, Wetter EK. Parental depressive symptoms and children’s sleep: the role of family conflict. J Child Psychol Psychiatry. 2012;53:806-814.
6. Rudd BN, Holtzworth-Munroe A, D’Onofrio BM, Waldron M. Parental relationship dissolution and child development: The role of child sleep quality. Sleep. 2019;42:zsy224.
7. Tikotzky L. Parenting and sleep in early childhood. Curr Opin Psychol. 2017;15:118-124.
8. El-Sheikh M, Buckhalt JA, Mark Cummings E, Keller P. Sleep disruptions and emotional insecurity are pathways of risk for children. J Child Psychol Psychiatry. 2007;48:88-96
9. Meltzer LJ, Montgomery-Downs HE. Sleep in the family. Pediatr Clin North Am. 2011;58:765-774.
10. Breitenstein RS, Doane LD, Clifford S, Lemery-Chalfant K. Children’s sleep and daytime functioning: Increasing heritability and environmental associations with sibling conflict. Soc Dev. 2018;27:967-983.

**Parental beliefs, attitudes, and knowledge (n=8)**

(e.g., knowledge of good sleep hygiene practices, knowledge about normal sleep patterns in infancy, perceptions of sleep patterns)

1. Rhodes RE, Berry T, Faulkner G, Latimer-Cheung AE, O’Reilly N, Tremblay MS, et al. Application of the multi-process action control framework to understand parental support of child and youth physical activity, sleep, and screen time behaviours. Appl Psychol Heal Well-Being. 2019;11:223-239.
2. Cook F, Bayer J, Le HND, Mensah F, Cann W, Hiscock H. Baby Business: A randomised controlled trial of a universal parenting program that aims to prevent early infant sleep and cry problems and associated parental depression. BMC Pediatr. 2012;12:13.
3. Crichton GE, Symon B. Behavioral management of sleep problems in infants under 6 months: What works? J Dev Behav Pediatr. 2016;37:164-171.
4. Bathory E, Tomopoulos S, Rothman R, et al. Infant sleep and parent health literacy. Acad Pediatr. 2016;16:550-557.
5. Martinez SM, Thompson-Lastad A. Latino Parents’ Insight on Optimal Sleep for Their Preschool-Age Child: Does Context Matter? Acad Pediatr. 2015;15:636-643.
6. McDowall PS, Elder DE, Campbell AJ. Relationship between parent knowledge of child sleep, and child sleep practices and problems: A pilot study in a children’s hospital cohort. J Paediatr Child Health. 2017;53:788-793.
7. Gunn HE, O’Rourke F, Dahl RE, et al. Young adolescent sleep is associated with parental monitoring. Sleep Heal. 2019;5:58-63.
8. Lindsay AC, Moura Arruda CA, Tavares Machado MM, De Andrade GP, Greaney ML. Exploring Brazilian immigrant mothers’ beliefs, attitudes, and practices related to their preschool-age children’s sleep and bedtime routines: A qualitative study conducted in the United States. Int J Environ Res Public Health. 2018;15:e1923.

**Family structure (n=3)**

(i.e. crowded/multi-housing)

1. Lindsay AC, Moura Arruda CA, Tavares Machado MM, De Andrade GP, Greaney ML. Exploring Brazilian immigrant mothers’ beliefs, attitudes, and practices related to their preschool-age children’s sleep and bedtime routines: A qualitative study conducted in the United States. Int J Environ Res Public Health. 2018;15:e1923.
2. Martinez SM, Thompson-Lastad A. Latino parents’ insight on optimal sleep for their preschool-age child: Does context matter? Acad Pediatr. 2015;15:636-643.
3. Julian MM, Leung CYY, Rosenblum KL, et al. Parenting and toddler self-regulation in low-income families: What does sleep have to do with it? Infant Ment Health J. 2019;40:479-495.
