# Supplementary material for: Development of a consensus statement on the role of the family in the physical activity, sedentary, and sleep behaviours of children and youth
Source: Int J Behav Nutr Phys Act. 2020 Jun 16;17:74. doi: 10.1186/s12966-020-00973-0 (PMC7296673; doi:10.1186/s12966-020-00973-0)
Supplement: Supplementary file 6 — Additional file 6. Table S2 (doc.). Inclusion and exclusion criteria for systematic literature searches on family systems theory (review #4), parental correlates (review #5), and family interventions (review #6) for movement behaviours of children and youth. [file 12966_2020_973_MOESM6_ESM.docx]

**Table S2. Inclusion and exclusion criteria for systematic literature searches on family systems theory (review #4), parental correlates (review #5), and family interventions (review #6) for movement behaviours of children and youth.**

| **Criterion** | **Inclusion criteria** | **Exclusion criteria** |
| --- | --- | --- |
| Language | English | Non-English studies |
| Type of article | Original research, published in a peer reviewed journal; review articles (literature, umbrella, critical, scoping or systematic reviews) | Articles that were not peer-reviewed |
| Study design | All study types |  |
| Study focus | Family theory applied to interventions targeting child movement behaviours; family theory developed for predicting and explaining child and youth movement behaviours.  Studies that investigate the impact of parental support for physical activity on 24-hour movement behaviours for children and youth; parental support included encouragement, transportation, facilitating one of the movement behaviours, coactivity or other supportive behaviors.  Studies that investigate the efficacy of family-based interventions and subsequent mediators and moderators of changes in children and youth movement behaviours | Studies investigating behavioural theories for child health behaviour changes not involving the family or family systems.  Studies that primarily examine child perceived parental support of movement behaviours.  Studies that investigate the influence of peers, school or the neighborhood environment on child movement behaviours. |
| Literature focus | Articles where the focus is on child and youth 24-hour movement behaviours and the influence of parental support and family functioning (i.e., family cohesion, family dynamics).  Articles that focus on family systems or family-based theory for the purpose of addressing child health behaviour change in the intervention setting. | Articles examining children’s perceptions of parental support.  Articles analyzing the influence of children/youth’s 24-hour movement behaviours on family members. |
| Population sample | Children and youth (5-17 years old); families with children and/or youth; parents/guardians/caregivers. | Articles that target parents or families with children under 5 years of age; articles that address children with a specific disease, illness, or condition, with the exception of children/youth with overweight/obesity. |
