# Supplementary material for: Development of a consensus statement on the role of the family in the physical activity, sedentary, and sleep behaviours of children and youth
Source: Int J Behav Nutr Phys Act. 2020 Jun 16;17:74. doi: 10.1186/s12966-020-00973-0 (PMC7296673; doi:10.1186/s12966-020-00973-0)
Supplement: Supplementary file 7 — Additional file 7. Review #4 (docx.). Search Process for the Family Systems in the Context of Child Health Behaviour Change and Family-Based Interventions Scoping Review (review #4). References extracted from the Family Systems in the Context of Child Health Behaviour Change Scoping Review (review #4). [file 12966_2020_973_MOESM7_ESM.docx]

Records identified through database searching
(n = 646)

Additional records identified through other sources
(n = 27)

Records after duplicates removed
(n = 453)

Records screened
(n = 453)

Records excluded
(n = 413)

Full-text articles assessed for eligibility
(n = 40)

Full-text articles excluded, with reasons (n = 20)

ut of targeted age range = 6

No theory specified = 4

Socio-Ecological Model = 4

Missing at least one child 24-hour movement behaviour = 5

Missing family theory = 1

Studies included in qualitative synthesis
(n = 20)

**Search Process for the Family Systems in the Context of Child Health Behaviour Change**

**and Family-Based Interventions Scoping Review (review #4).**

**References extracted from the Family Systems in the Context of Child Health Behaviour Change Scoping Review (review #4).**

1. Barnhill LR. Healthy Family Systems. Fam Coord. 1979;28:94–100.

2. Bowlby J, Ainsworth M, Bretherton I, Ainsworth M. The Origins of Attachment Theory: Dev Psychol. 1992;759–75.

3. Bronfenbrenner U. Ecology of the Family as a Context for Human Development: Research Perspectives. Dev Psychol. 1986;22:723–42.

4. Cox MJ, Paley B. Family as systems. Annu Rev Psychol. 1997;48:243–67.

5. Cox MJ, Paley B. Understanding families as systems. Curr Dir Psychol Sci. 2003;193–6.

6. Davison KK, Jurkowski JM, Lawson HA. Reframing family-centred obesity prevention using the Family Ecological Model. Public Health Nutr. 2012;16:1861–9.

7. Davison, K CK. Opportunities to Prevent Obesity in Children within Families: an Ecological Approach. Obes Prev Public Heal. 2005;207–30.

8. Davison KK, Lawson HA, Coatsworth JD. The Family-Centered Action Model of Intervention Layout and Implementation (FAMILI): The Example of Childhood Obesity. Health Promot Pract. 2012;13:454–61.

9. El-Sheikh M, Kelly RJ. Family Functioning and Children’s Sleep. Child Dev Perspect. 2017;11:264–9.

10. Jang M, Whittemore R. The Family Management Style Framework for Families of Children with Obesity. J Theory Constr Test. 2014;19:5–15.

11. Gunn HE, Eberhardt KR. Family Dynamics in Sleep Health and Hypertension. Curr Hypertens Rep. Current Hypertension Reports. 2019;21.

12. Kitzman-Ulrich, Heather, Wilson D, St George, Sara, Lawman Hannah, Segal Michelle, Fairchild A. The Integration of a Family Systems Approach for Understanding Youth Obesity, Physical Activity, and Dietary Programs. Clin Child Fam Psychol Rev. 2010;13:231–53.

13. Kitzman-ulrich H, Hampson R, Wilson DK, Presnell K, Brown A, Boyle MO. An Adolescent Weight-Loss Program Integrating Family Variables Reduces Energy Intake. American Dietetic Association. 2009;109:491–6.

14. Minuchin S. Families and Individual Development: Provocations from the Field of Family Therapy. Child Dev. 1985;56:289–302.

15. Ng K, Smith SD. The Relationships Between Attachment Theory and Intergenerational Family Systems Theory. Fam J. 2006;14:430–40.

16. Niermann CYN, Gerards SMPL, Kremers SPJ. Conceptualizing family influences on children’s energy balance-related behaviors: Levels of interacting family environmental subsystems (The LIFES framework). Int J Environ Res Public Health. 2018;15.

17. Nowicka P, Flodmark CE. Family therapy as a model for treating childhood obesity: Useful tools for clinicians. Clin Child Psychol Psychiatry. 2011;16:129–45.

18. Palombi M. From Gestalt Therapy to Family Systems: How Theoretical Frameworks Inform Clinical Applications. 2018;514–27.

19. Pietrobelli A, Lissau I, Nowicka P, Ho P. Family Weight School treatment: 1-year results in obese adolescents. Int J Pediatr Obes. 2008;1–7.

20. Pratt KJ, Skelton JA. Family Functioning and Childhood Obesity Treatment: A Family Systems Theory-Informed Approach. Ideas Innov. 2018;18.

21. Rothbaum F, Rosen K, Ujiie T, Uchida N. Family Systems Theory, Attachment Theory, and Culture. 2002;41:328–50.

22. Skelton, J A, Buehler C, Irby MB, Grzywacz J. Where are family theories in family-based obesity treatment? Conceptualizing the study of families in pediatric weight management. Int J Obes. 2014;36:891–900.

23. Sung-Chan P, Sung YW, Zhao X, Brownson RC. Family-based models for childhood-obesity intervention: A systematic review of randomized controlled trials. Obes Rev. 2013;14:265–78.
