# Supplementary material for: Development of a consensus statement on the role of the family in the physical activity, sedentary, and sleep behaviours of children and youth
Source: Int J Behav Nutr Phys Act. 2020 Jun 16;17:74. doi: 10.1186/s12966-020-00973-0 (PMC7296673; doi:10.1186/s12966-020-00973-0)
Supplement: Supplementary file 8 — Additional file 8. Review #5 (docx.). Search Process for the Correlates of Parental Support of Child and Youth Physical Activity, Sedentary Behaviour, and Sleep Systematic Review (review #5). References Extracted from the Correlates of Parental Support of Child and Youth Physical Activity, Sedentary Behaviour, and Sleep Systematic Review (review #5). [file 12966_2020_973_MOESM8_ESM.docx]

Additional records identified through other sources
(n = 22)

Records identified through database searching

(n = 2,917)

## Identification

Records after duplicates removed
(n = 2,881)

## Screening

Records screened
(n = 2,881)

## Eligibility

Full-text articles excluded, with reasons (n = 33)

Child level measures only = 11

No measures of support = 8

No correlates provided for support = 9

Child age out of scope = 2

Qualitative study = 2

Experimental study = 1

Full-text articles assessed for eligibility
(n = 58)

## Included

Studies included in qualitative synthesis
(n = 25)

*22 unique datasets

**Search Process for the Correlates of Parental Support of Child and Youth Physical Activity, Sedentary Behaviour, and Sleep Systematic Review (review #5).**

**References Extracted from the Correlates of Parental Support of Child and Youth Physical Activity, Sedentary Behaviour, and Sleep Systematic Review (review #5).**

1. Belanger-Gravel A, Gauvin L, Lagarde F, Laferte M. Correlates and moderators of physical activity in parent-tween dyads: a socio-ecological perspective. Public Health. 2015;129:1218–23.

2. Carver A, Timperio A, Hesketh K, Crawford D. Are children and adolescents less active if parents restrict their physical activity and active transport due to perceived risk? Soc Sci Med J. 2010;70:1799–805.

3. Davison, Kirsten, Cutting, Tanja, Birch L. Parents’ Activity-Related Parenting Pracctices Predict Girls’ Physical Activity. Med Sci Sport Exerc. 2003;35:1–7.

4. Davison KK, Nishi A, Kranz S, Wyckoff L, May JJ, Earle-Richardson GB, et al. Associations among social capital, parenting for active lifestyles, and youth physical activity in rural families living in upstate New York. Soc Sci Med. 2012;75:1488–96.

5. Forthofer, Melinda, Dowda, Marsha, Mciver, Kerry, Barr-Anderson, Daheia, Pate R. Associations between Maternal Support and Physical Activity Among 5th Grade Students Melinda. Matern Child Heal J. 2016;20:720–9.

6. Greguool M, Gobbi E, Carraro A. Physical activity practice among children and adolescents with visual impairment-influence of parental support and perceived barriers. Disabil Rehabil. 2015;37:327–30.

7. Gunn HE, O’Rourke F, Dahl RE, Goldstein TR, Rofey DL, Forbes EE, et al. Young adolescent sleep is associated with parental monitoring. Sleep Heal. 2019;5:58–63.

8. de la Haye K, de Heer HD, Wilkinson A V, Koehly LM. Predictors of parent–child relationships that support physical activity in Mexican–American families. J Behav Med. 2014;37:234–44.

9. Hoefer WR, McKenzie TL, Sallis JF, Marshall SJ, Conway TL. Parental provision of transportation for adolescent physical activity. Am J Prev Med. 2001;21:48–51.

10. Huffman LE, Wilson DK, Horn ML, Pate RR. Associations between parenting factors, motivation, and physical activity in overweight African American adolescents. Ann Behav Med. 2018;52:93–105.

11. Ice CL, Neal WA, Cottrell L. Parental Efficacy and Role Responsibility for Assisting in Child’s Healthful Behaviors. Educ Urban Soc. 2014;46:699–715.

12. Langer SL, Crain AL, Senso MM, Levy RL, Sherwood NE. Predicting child physical activity and screen time: parental support for physical activity and general parenting styles. J Pediatr Psychol. 2014;39:633–42.

13. Liszewska N, Scholz U, Radtke T, Horodyska K, Liszewski M, Luszczynska A. Association between children’s physical activity and parental practices enhancing children’s physical activity: The moderating effects of children’s BMI z-score. Front Psychol. 2018;8.

14. Lloyd AB, Lubans DR, Plotnikoff RC, Collins CE, Morgan PJ. Maternal and paternal parenting practices and their influence on children’s adiposity, screen-time, diet and physical activity. Appetite. 2014;79:149–57.

15. Rhodes RE, Benoit C, Blanchard CM, Symons Downs D, Levy Milne R, Naylor PJ, et al. Predicting regular physical activity continuation after the onset of first-time parenthood. Ann Behav Med. 2010;39:31.

16. Rhodes RE, Berry T, Faulkner G, Latimer-Cheung AE, O’Reilly N, Tremblay MS, et al. Application of the Multi-Process Action Control Framework to Understand Parental Support of Child and Youth Physical Activity, Sleep, and Screen Time Behaviours. Appl Psychol Heal Well-Being. 2019;11:223–39.

17. Pyper E, Harrington D, Manson H. Do parents’ support behaviours predict whether or not their children get sufficient sleep? A cross-sectional study. BMC Public Health. 2017;17:432.

18. Rhodes RE, Spence JC, Berry T, Deshpande S, Faulkner G, Latimer-Cheung A, et al. Understanding action control of parent support behavior for child physical activity. Heal Psychol. 2016;35:131–40.

19. Rhodes RE, Spence JC, Berry T, Deshpande S, Faulkner G, Latimer-Cheung AE, et al. Predicting changes across 12 months in three types of parental support behaviors and mothers’ perceptions of child physical activity. Ann Behav Med. 2015;49:853–64.

20. Richards R, Poulton R, Reeder AI, Williams S. Childhood and contemporaneous correlates of adolescent leisure time physical inactivity: a longitudinal study. J Adolesc Heal. 2009;44:260–7.

21. Tate, Eleanor, Shah, Anuja, Jones, Malia, Pentz, Mary Ann, Liao, Yue, Dunton G. Toward a Better Understanding of the Link between Parent and Child Physical Activity Levels: The Moderating Role of Parental Encouragement Eleanor. J Phys Act Heal. 2016;112:1238–44.

22. Totland TH, Bjelland M, Lien N, Bergh IH, Gebremariam MK, Grydeland M, et al. Adolescents’ prospective screen time by gender and parental education, the mediation of parental influences. Int J Behav Nutr Phys Act. 2013;10:1–10.

23. Wilson DK, Lawman HG, Segal M, Chappell S. Neighborhood and parental supports for physical activity in minority adolescents. Am J Prev Med. 2011;41:399–406.

24. Zhao J, Gao Z, Settles BH. Determinants of parental perception and support on youth physical activity. Fam Community Heal. 2013;36:77–88.
