# Supplementary material for: Development of a consensus statement on the role of the family in the physical activity, sedentary, and sleep behaviours of children and youth
Source: Int J Behav Nutr Phys Act. 2020 Jun 16;17:74. doi: 10.1186/s12966-020-00973-0 (PMC7296673; doi:10.1186/s12966-020-00973-0)
Supplement: Supplementary file 9 — Additional file 9:. Figure S1 (doc.). Search process of the Review of Reviews on Interventions Involving the Family to Change Child and Youth Physical Activity, Sedentary Behaviour, and Sleep (review #6). [file 12966_2020_973_MOESM9_ESM.docx]

Records excluded
(n = 1494)

Records screened
(n = 1510)

Records after duplicates removed
(n = 1,510)

## Identification

## Eligibility

## Included

## Screening

Records identified through database searching

(n = 1540)

Additional records identified through other sources
(n = 8)

Full-text articles assessed for eligibility (n = 16)

Full-text articles excluded, with reasons (n =5)

Not a systematic review = 1

School-based intervention only = 1

Weight-loss interventions = 1

Review of reviews = 2

No behavioral outcomes reported = 1

Studies included in qualitative synthesis (n = 11)

**Figure S1. Search process of the Review of Reviews on Interventions Involving the Family to Change Child and Youth Physical Activity, Sedentary Behaviour, and Sleep (review #6).**
